# Tumor endothelial cell autophagy is a key vascular‐immune checkpoint in melanoma

**DOI:** 10.15252/emmm.202318028

**Published:** 2023-11-27

**Authors:** Jelle Verhoeven, Kathryn A Jacobs, Francesca Rizzollo, Francesca Lodi, Yichao Hua, Joanna Poźniak, Adhithya Narayanan Srinivasan, Diede Houbaert, Gautam Shankar, Sanket More, Marco B Schaaf, Nikolina Dubroja Lakic, Maarten Ganne, Jochen Lamote, Johan Van Weyenbergh, Louis Boon, Oliver Bechter, Francesca Bosisio, Yasuo Uchiyama, Mathieu JM Bertrand, Jean Christophe Marine, Diether Lambrechts, Gabriele Bergers, Madhur Agrawal, Patrizia Agostinis

**Affiliations:** ^1^ Cell Death Research and Therapy Laboratory Center for Cancer Biology, VIB Leuven Belgium; ^2^ Department of Cellular and Molecular Medicine KU Leuven Leuven Belgium; ^3^ Laboratory of Translational Genetics Center for Cancer Biology, VIB Leuven Belgium; ^4^ Department of Human Genetics KU Leuven Leuven Belgium; ^5^ Laboratory of Tumor Microenvironment and Therapeutic Resistance Center for Cancer Biology, VIB Leuven Belgium; ^6^ Department of Oncology KU Leuven Leuven Belgium; ^7^ Laboratory for Molecular Cancer Biology Center for Cancer Biology, VIB Leuven Belgium; ^8^ Laboratory of Translational Cell and Tissue Research, Department of Pathology KULeuven and UZ Leuven Leuven Belgium; ^9^ Department of Pathology UZLeuven Leuven Belgium; ^10^ Laboratory of Clinical and Epidemiological Virology, Department of Microbiology, Immunology and Transplantation, Rega Institute for Medical Research KU Leuven Leuven Belgium; ^11^ Polpharma Biologics Utrecht The Netherlands; ^12^ Department of General Medical Oncology UZ Leuven Leuven Belgium; ^13^ Department of Cellular and Molecular Neuropathology Juntendo University Graduate School of Medicine Tokyo Japan; ^14^ VIB Center for Inflammation Research Ghent University Ghent Belgium; ^15^ Department of Biomedical Molecular Biology Ghent University Ghent Belgium

**Keywords:** autophagy, cancer, immunotherapy, inflammation, tumor endothelial cells, Autophagy & Cell Death, Cancer, Vascular Biology & Angiogenesis

## Abstract

Tumor endothelial cells (TECs) actively repress inflammatory responses and maintain an immune‐excluded tumor phenotype. However, the molecular mechanisms that sustain TEC‐mediated immunosuppression remain largely elusive. Here, we show that autophagy ablation in TECs boosts antitumor immunity by supporting infiltration and effector function of T‐cells, thereby restricting melanoma growth. In melanoma‐bearing mice, loss of TEC autophagy leads to the transcriptional expression of an immunostimulatory/inflammatory TEC phenotype driven by heightened NF‐kB and STING signaling. In line, single‐cell transcriptomic datasets from melanoma patients disclose an enriched Inflammatory^High^/Autophagy^Low^ TEC phenotype in correlation with clinical responses to immunotherapy, and responders exhibit an increased presence of inflamed vessels interfacing with infiltrating CD8^+^ T‐cells. Mechanistically, STING‐dependent immunity in TECs is not critical for the immunomodulatory effects of autophagy ablation, since NF‐kB‐driven inflammation remains functional in STING/ATG5 double knockout TECs. Hence, our study identifies autophagy as a principal tumor vascular anti‐inflammatory mechanism dampening melanoma antitumor immunity.

## Graphical abstract

Tumor endothelial cell (EC)‐autophagy was identified as a major barrier to anti‐tumor immune responses against melanoma.

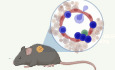

**The paper explained**

**Problem**

In advanced cancers, vessel remodeling through unproductive angiogenesis generates a barrier to T‐cells, by reducing blood flow and by preventing cytotoxic T‐cells from entering and attacking the tumor. Hence, harnessing the tumor vasculature could be crucial in order to fully unleash antitumor immunity and the therapeutic potential of immunotherapy. The mechanisms controlling the immunosuppressive phenotype of tumor endothelial cells are largely unknown. Gaining insights into these mechanisms may provide novel and actionable tumor vessel‐targeted strategies that can improve patient response to immunotherapy.

**Results**

Our preclinical data demonstrate that endothelial cell (EC)‐specific deletion of autophagy genes endorses an inflammatory tumor ECs (TECs) phenotype, by the concomitant activation of STING and NF‐KB pathways, which promotes immunosurveillance and controls melanoma tumor burden. We identify a TEC‐inflammatory gene signature that inversely correlates with autophagy genes in mice and in different treatment‐naïve human cancers. This Inflammatory^High^/Autophagy^Low^ signature is positively associated with immunotherapy response in melanoma patients; with responders showing an increased fraction of CD8^+^ T‐cells in close contact with inflamed vessels.

**Impact**

We provide unappreciated insights into the immunosuppressive role of autophagy in tumor endothelial cells and the antitumor immunity benefits of autophagy inhibition in tumor vessels. We show that in several cancers, a heightened TEC‐autophagy status inversely correlates with an inflamed EC signature, which is predictive of poor immunotherapy response in melanoma. Thus, the Inflammatory^High^/Autophagy^Low^ TEC status may aid in patient‐based selection. Our data advocate for the development of vascular‐homed autophagy inhibitors for their use in combination with immune checkpoint blockers.

## Introduction

The success of immunotherapy using immune checkpoint blockers (ICBs) has been remarkable in boosting CD8^+^ T‐cell‐mediated antitumor immunity in the treatment of various solid tumors, including melanoma (Robert *et al*, 2015; Huang & Zappasodi, 2022). However, a substantial number of melanoma patients still fail to respond to immunotherapy (Jerby‐Arnon *et al*, 2018). Melanoma is a prototypical immunogenic tumor that can maintain a profound immunosuppressive tumor microenvironment (TME) by coopting multiple cancer cell‐intrinsic and extrinsic mechanisms (Fischer *et al*, 2018; Huang & Zappasodi, 2022). Together, these mechanisms limit the therapeutic efficacy of ICBs. Therefore, unraveling the molecular mechanisms that facilitate the immunosuppressive TME is of primary clinical importance to overcome resistance to immunotherapy in melanoma.

Infiltration of CD8^+^ T‐cells into the tumor parenchyma is associated with a favorable prognosis and improved response to ICBs (Yang *et al*, 2019; Huang & Zappasodi, 2022). In particular, recent studies indicate that the density, distribution, and activation pattern of tumor‐infiltrating lymphocytes (TILs) in primary melanoma has prognostic significance (Rohaan *et al*, 2022). In a recent phase 3 trial of patients with advanced melanoma, expanding the number of tumor‐reactive T‐lymphocytes through autologous TIL therapy showed superior efficacy compared to anti‐PD1 therapy (Rohaan *et al*, 2022). This suggests that facilitating the infiltration of tumor‐reactive T‐lymphocytes within the tumor parenchyma may be critical in overcoming the immunosuppressive TME in melanoma.

To recognize cancer cells and eliminate them, cytotoxic CD8^+^ T‐cells need to: extravasate the vascular lumen, transmigrate the endothelial lining to infiltrate the tumor, survive within the TME, and evade immunosuppressive signals. Compared with the mostly quiescent endothelial cells (ECs) of healthy tissues, TECs are constantly exposed to angiogenic cues driving functional and structural abnormalities including: loose intercellular junctions, reduced pericyte coverage, and vascular leakiness (Jain, 2014; Schaaf *et al*, 2018; García‐Caballero *et al*, 2022). In advanced tumors, vessel remodeling through aberrant angiogenesis generates a barrier to T‐cell infiltration by heightening the immunosuppressive features of TECs. TECs impair lymphocyte recruitment and transmigration (diapedesis) by reducing the secretion of immune‐attracting chemokines (e.g., CXCL10, CXCL1), expression of adhesion molecules (e.g., vascular cell adhesion protein 1 [VCAM1], intercellular adhesion molecule 1 [ICAM1], E‐ and P‐selectins), and the antigen presentation machinery (e.g., MHC class I) on their surface (Griffioen *et al*, 1996; Huang *et al*, 2015; Amersfoort *et al*, 2022). Through these immunosuppressive processes TECs impair antitumor immunity (Hua *et al*, 2022; Sun & Hornung, 2022) and represent a bottleneck for immunotherapies. Unraveling key mechanisms capable of counteracting the immunosuppressive features of TECs, or endorsing their inflamed status, could be crucial to fully unleash anti‐tumor immune responses.

Macroautophagy (hereafter called autophagy) is a vital regulator of the immune and inflammatory microenvironment in tumors and other inflammatory conditions (Levine *et al*, 2011). Autophagy is the primary lysosomal degradation pathway responsible for the degradation of intracellular material to maintain cell survival and energy homeostasis (Singh & Cuervo, 2011). In advanced tumors, autophagy supports the heightened metabolic needs of cancer cells and exerts cell nonautonomous functions through the unconventional secretion of inflammatory mediators (Katheder *et al*, 2017; Debnath *et al*, 2023). Recent studies have shown that loss of autophagy in the host tissues induces systemic and tissue‐specific metabolic reprogramming that favors antitumor immunity. Full‐body or liver‐specific deletion of the canonical autophagy genes *Atg5* or *Atg7* impairs tumor immunotolerance and the growth of tumors with high mutational burdens, while remaining ineffective against tumors with a low mutational load (Poillet‐Perez *et al*, 2020). T‐cells from *Atg5* ^−/−^ mice display greater T‐cell‐intrinsic effector function resulting from enhanced glycolytic metabolism and transcriptional upregulation of both metabolic and effector target genes (DeVorkin *et al*, 2019).

While these studies support the immunosuppressive nature of host autophagy, it is not well understood how autophagy regulates immunomodulatory functions of blood vessels at the interface with immune cells, under physiological or pathological conditions. Recently, in a model of acute physiological inflammation, genetic loss of autophagy in venular ECs of cremaster muscles exacerbated neutrophil transendothelial migration and tissue damage by controlling the degradation of adhesion molecules at the sites of EC contacts (Reglero‐Real *et al*, 2021). Yet, it remains unknown to which extent TEC‐associated autophagy shapes inflammation, immunosurveillance, and responses to ICBs.

In this study, we combined multiplexed analysis and single‐cell transcriptomic profiling of TECs from human melanoma, with *in vivo* functional/mechanistic studies in melanoma‐bearing mice in which different autophagy genes were conditionally deleted in ECs. We demonstrate that autophagy in TECs exerts specific and persuasive anti‐inflammatory and immunosuppressive functions, which impede antitumor immunity and responses to immunotherapy.

## Results

### Loss of autophagy in endothelial cells improves melanoma immunosurveillance

**Figure EV1:**
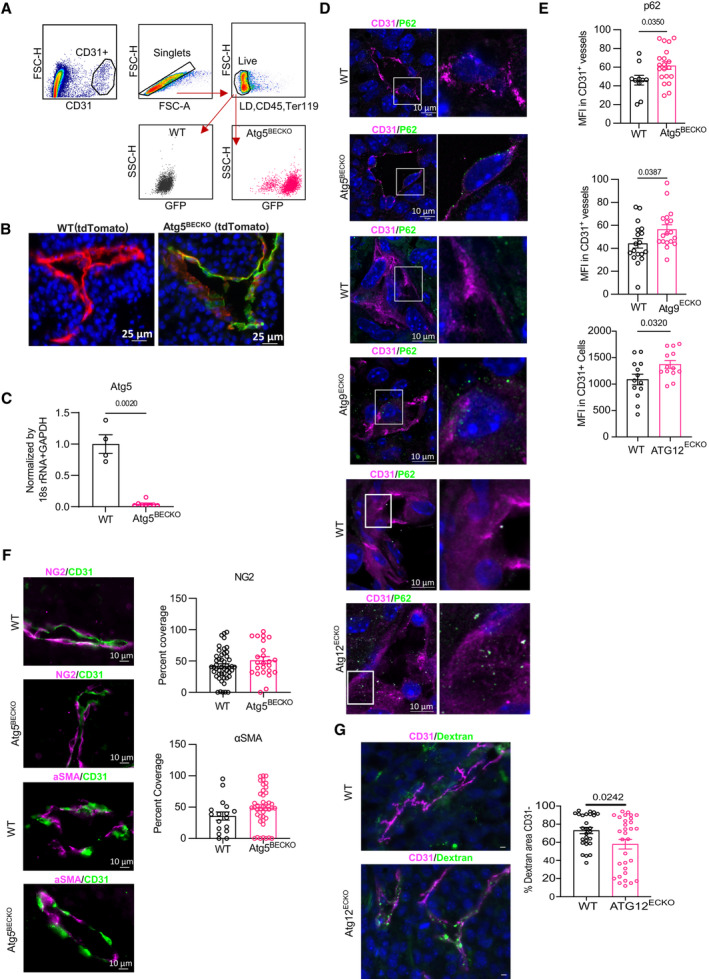
Validation of genetic loss of *Atg5* in tumor endothelial cells AGating strategy for quantifying GFP signal in CD31^+^ tumor endothelial cells in subcutaneous B16‐F10 tumors from WT and Atg5^BECKO^ mice.BRepresentative immunofluorescence images showing tdtomato and GFP in CD31^+^ tumor endothelial cells in subcutaneous B16‐F10 tumors from WT and Atg5^BECKO^ mice with subcutaneous B16‐F10 tumors.CGene expression of *Atg5* normalized to 18 s rRNA + GAPDH in sorted CD31^+^ tumor endothelial cells in subcutaneous B16‐F10 tumors from WT and Atg5^BECKO^ mice. Forward primer was designed to bind in Exon 3 region and reverse primer was designed to bind in Exon 4 region of *Atg5* gene.D, ERepresentative immunofluorescence images and quantification for p62 (MFI), in CD31^+^ tumor endothelial cells from subcutaneous B16‐F10 tumors from WT and Atg5^BECKO^, ATG9a^ECKO^, And ATG12^ECKO^ mice.FRepresentative immunofluorescence images and quantification for NG2 (% coverage) and αSMA (% coverage) in CD31^+^ tumor endothelial cells from subcutaneous B16‐F10 tumors from WT and Atg5^BECKO^ mice.GRepresentative immunofluorescence images and quantification for dextran outside of CD31^+^ vessels from subcutaneous B16‐F10 tumors from WT and Atg12^ECKO^ mice. Scale bars represent 10 μm. Data Information: For immunofluorescence staining, at least 3 WT and 3 Atg5^BECKO^ mice were used for the quantification. All data represent mean ± s.e.m. Statistical differences were determined using two‐sided Student's *t*‐test.

To uncover the contribution of EC‐autophagy in regulating immunosurveillance in melanoma, we crossed the inducible *PDGFb‐cre* ^*ERT2*^ *Rosa26* ^*tdTomato*/*tdTomato*^ *line* with *Atg5* ^fl/fl^ mice to delete the essential autophagy gene *Atg5* from blood ECs (i.e., Atg5^BECKO^) upon tamoxifen administration. Loss *Atg5* exon3 was verified using qPCR on sorted murine TECs from B16‐F10 melanoma‐bearing mice and by reporter protein expression (switch from dTomato to GFP after deletion) by flow cytometry (FACs) and microscopy (Fig EV1A–C). Genetic loss of *Atg5* in TECs increased the presence of p62 puncta in CD31^+^ vessels, indicating blockade in autophagy flux (Fig EV1D and E).

**Figure 1:**
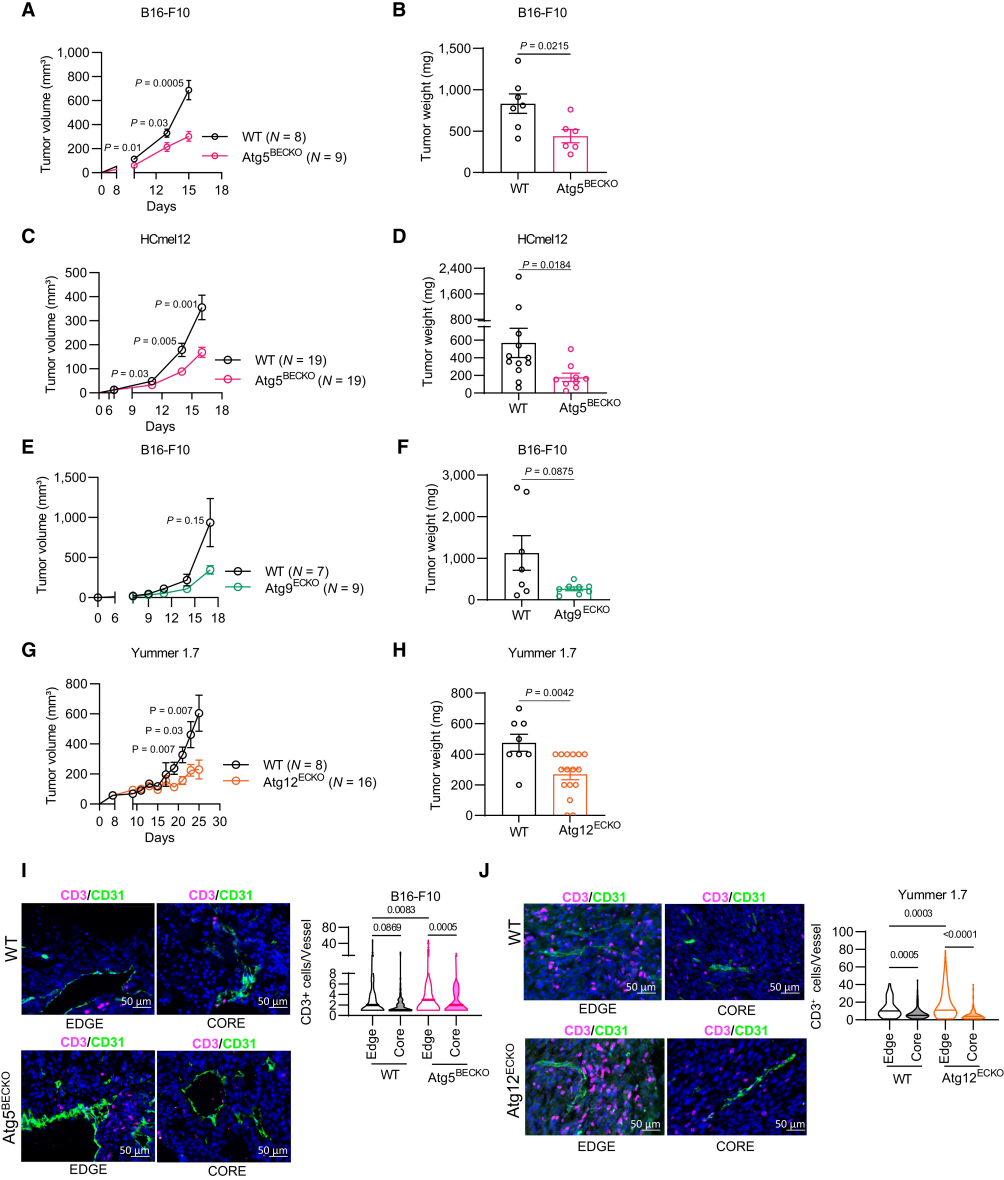
Genetic loss of autophagy in endothelial cells decreases growth of melanoma A–HTumor volume (A, C, E, G) and end‐point tumor weights (B, D, F, H) of WT and autophagy KO mice. Each point represents an individual tumor, *n* = at least 7.A, BWT and ATG5^BECKO^ mice subcutaneously injected with B16‐F10.C, DWT and ATG5^BECKO^ mice subcutaneously injected with HcMel12‐mcherry^+^ melanoma cells.E, FWT and Atg9a^ECKO^ mice subcutaneously injected with B16‐F10 melanoma.G, HWT and Atg12^ECKO^ mice subcutaneously injected with Yummer 1.7 melanoma cells.I, JRepresentative images and quantification of immunofluorescence staining for CD3^+^ T‐cells in the edge and the rim of tumor sections of subcutaneous B16‐F10 from WT and Atg5^BECKO^ mice (I) or Yummer 1.7 (J) tumors from WT and Atg12^BECKO^ mice. Scale bar represents 50 μm. For immunofluorescence staining, at least 3 WT and 3 Atg5^BECKO^ mice and 2 WT and 3 Atg12^ECKO^ mice were used for the quantification. Data Information: All data represent mean ± s.e.m. Statistical differences were determined using a two‐sided Student's *t*‐test (A–H) or one‐way Anova with Holm–Sidak correction for multiple comparisons (I, J). Source data are available online for this figure.

We then compared the growth of subcutaneously implanted murine B16‐F10 melanoma, a prototypical poorly immunogenic tumor, or the more immunogenic Hcmel12 (mCherry^+^) melanoma, derived from a serial transplant of a UV‐induced primary melanoma in *Hgf‐Cdk4*_*R24C*_ mice (Bald *et al*, 2014), in WT and Atg5^BECKO^ mice. In both syngeneic models, deletion of *Atg5* in blood ECs yielded a similar reduction in tumor burden (Fig 1A–D). To exclude the possibility that differences were caused by possible nonautophagic functions of Atg5 (Peña‐Martinez *et al*, 2022), we included Atg12^ECKO^ or Atg9^ECKO^ mice which were generated by crossing *Atg12* ^*f*l/fl^ or *Atg9* ^*f*l/fl^ mice with the *VeCadh‐cre* ^*ERT2*^ line (Sörensen *et al*, 2009; Yamaguchi *et al*, 2018). Similar to what was observed upon deletion of Atg5, loss of Atg12 or Atg9 in ECs increased p62 granularity in CD31^+^ tumor vessels indicating reduced autophagy flux (Fig EV1D and E).

As the canonical autophagy Atg5/Atg12 proteins are part of the same multimeric ubiquitin‐like conjugation complex that regulates the developing autophagosome (Hanada *et al*, 2007), we reasoned that *Atg*12 deletion should be functional epistatic to the loss of *Atg5* in TECs. Atg9 is a transmembrane protein that is phosphorylated by ULK1 and recruited to the PI3KC3 autophagy initiation machinery complex, which governs the first steps of autophagosome formation (Nishimura & Tooze, 2020). Moreover, while both Atg5 and Atg12 are involved in LC3‐associated phagocytosis, components of the ULK1 complex, including Atg9, do not seem to be implicated (Peña‐Martinez *et al*, 2022). We observed similar phenotypic differences in tumor burden when comparing WT and Atg12^ECKO^ or Atg9^ECKO^ mice inoculated with B16‐F10 or the immunogenic YUMMER 1.7 melanoma cells, carrying the *Braf* ^V600E^/*Pten* ^−/−^/*Cdkn2a* ^−/−^ mutations, respectively (Fig 1E–H).

Vessel maturation as measured by double staining for CD31 and the mural cell markers αSMA and NG2 showed a trend—albeit nonsignificant—toward increased pericyte coverage in the vessels from autophagy‐deficient mice (Fig EV1F). Injecting tumor‐bearing WT and Atg12^ECKO^ mice with dextran‐FITC to measure the fraction of extravasating leaky vessels, followed by staining for CD31, showed that loss of autophagy reduced dextran‐FITC leakage (Fig EV1G). This suggests that genetic loss of autophagy, in these models, partially improves tumor vessel functionality, which could be associated with an improved interface with immune cells (Schaaf *et al*, 2018).

In line with this, in both B16‐F10 and YUMMER 1.7 tumors, loss of TEC‐autophagy increased the presence of CD3^+^ T‐lymphocytes around CD31^+^ TECs, particularly at the more vascularized tumor edge (Fig 1I and J), suggesting an increased ability to attract and recruit T‐cells, irrespective of the immunogenicity of the melanoma models used.

**Figure 2:**
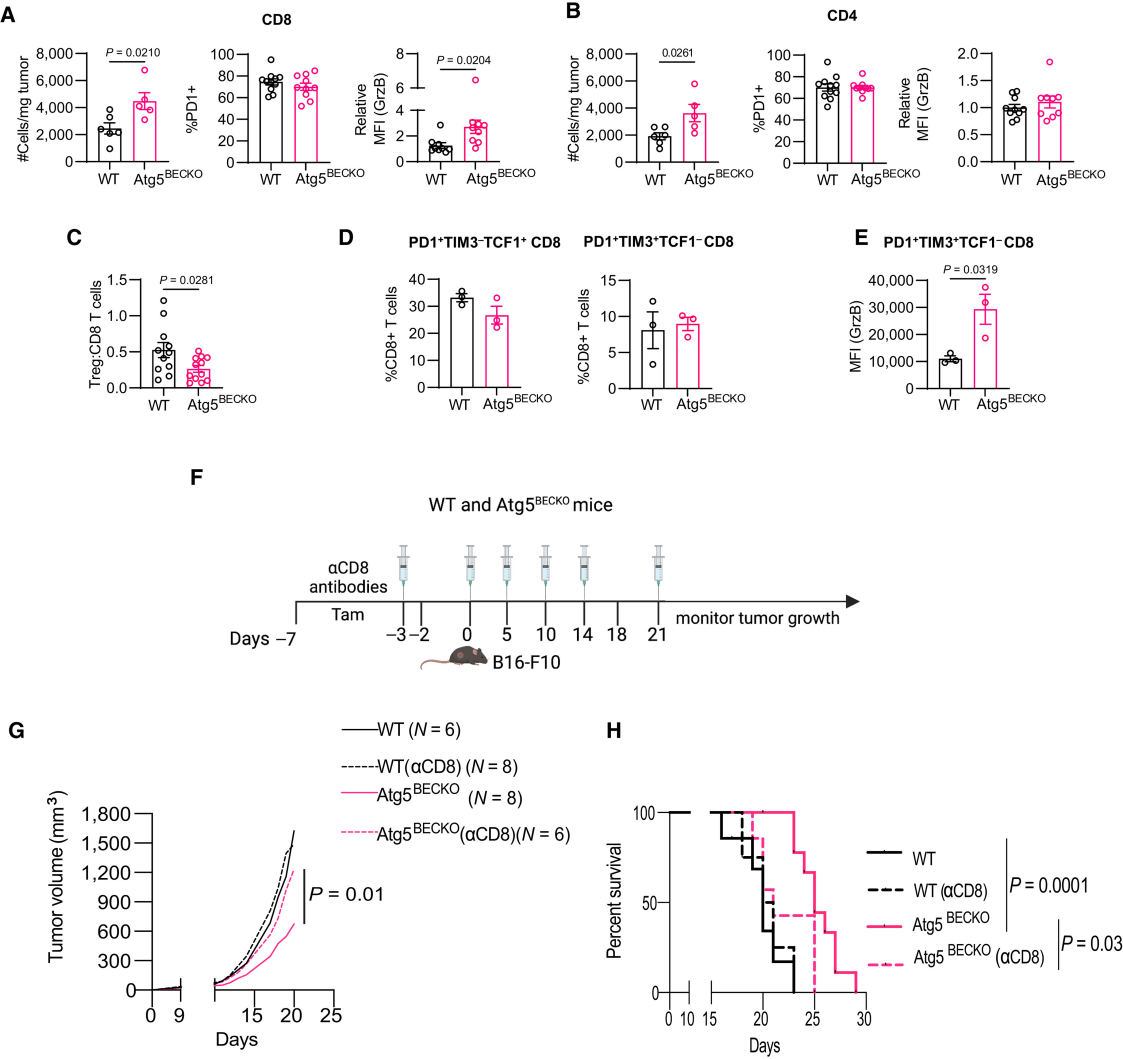
Deletion of *Atg5* in tumor endothelial cells promote CD8^+^ T‐cell mediated antitumor immune responses A–EFlow cytometry analysis of tumor‐infiltrating lymphocytes in subcutaneous B16‐F10 tumors from WT and Atg5^BECKO^ mice. Each point on the graph represents an individual mouse.ACharacterization of tumor‐infiltrating CD8^+^ T‐cells (# cells/mg tumor), PD1^+^ cells (% of CD8^+^ T‐cells) and Granzyme B (GrzB) (mean fluorescence intensity or MFI in CD8^+^ T‐cells).BCharacterization of CD4^+^ T‐cells (# cells/mg tumor), PD1^+^ cells (% of CD4^+^ T‐cells), and Granzyme B (GrzB) (MFI in CD4^+^ T‐cells).CRatio of # of CD4^+^FOXP3^+^ Tregs to CD8^+^ T‐cells.A–CData shown are representative of two independent experiments (A–C).DPercentage of CD8^+^ T‐cells which are PD1^+^TIM3^+^TCF1^−^ and PD1^+^TIM3^−^TCF1^+^ cells.EIntracellular Granzyme B (GrzB) staining of PD1^+^TIM3^+^TCF1^−^ cells.FSchematic representation for depleting CD8^+^ cells in subcutaneous B16‐F10 tumor‐bearing WT and Atg5^BECKO^ mice.G, HEnd‐point tumor volume (G) and survival analysis (H) of B16‐F10 tumor‐bearing WT and Atg5^BECKO^ mice injected with vehicle or αCD8 antibody (100 μg per mouse). Data Information: All data represent mean ± s.e.m. Statistical differences were determined by two‐sided Student's *t*‐test (A–G) or Kaplan–meier survival analysis (H). Source data are available online for this figure.

We then analyzed TILs from B16‐F10 bearing WT and Atg5^BECKO^ mice for the presence of CD8^+^ and CD4^+^ T‐cells by FACs. Melanomas of Atg5^BECKO^ mice, compared to those of WT mice, displayed an increased amount of both CD8^+^ T and CD4^+^ T‐cells (Fig 2A and B) and harbored CD8^+^ T‐cells with higher expression of the key effector molecule Granzyme B (GrzB) (Fig 2A). Melanoma‐bearing Atg5^BECKO^ mice also exhibited a decreased ratio of immunosuppressive T regulatory cells (Tregs) to CD8^+^ T‐cells, an indicator of a diminished immunosuppressive status of the TME (Fig 2C) (Chen *et al*, 2005).

**Figure EV2:**
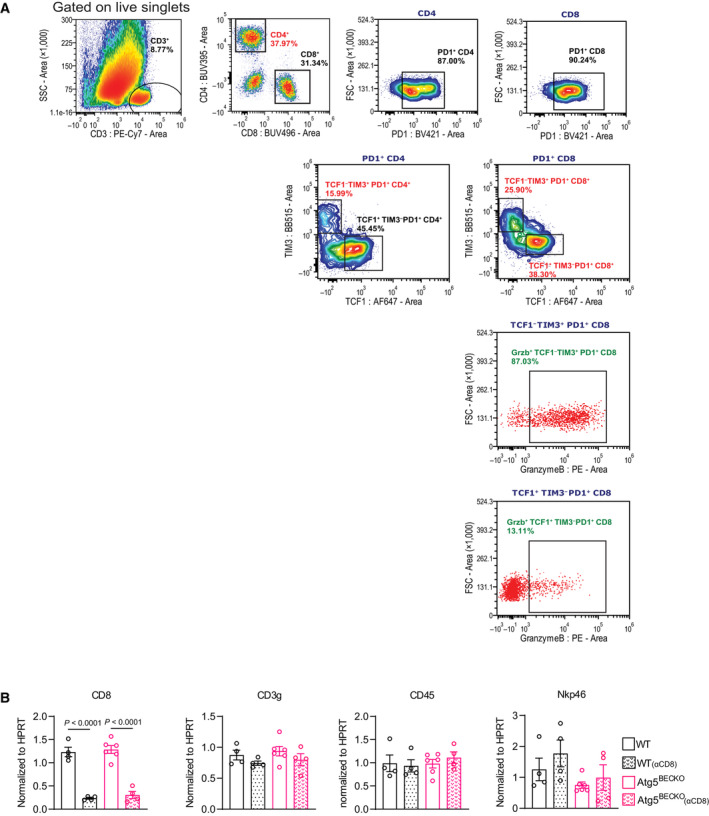
Immunophenotyping of WT and Atg5^BECKO^ mice with subcutaneous B16‐F10 melanoma AGating strategy for analyzing immune cell subsets in subcutaneous B16‐F10 melanoma from WT and Atg5^BECKO^ mice.BGene expression of *CD8*, *CD3g*, *CD45*, and *Nkp46* in blood collected from WT and Atg5^BECKO^ mice with subcutaneous B16‐F10 tumors and injected with αCD8 antibody. Each point represents 1 mouse; with at least *n* = 4 mice per group. Data Information: All data represent mean ± s.e.m. Statistical differences were determined using one‐way Anova with Tukey corrections for multiple comparisons (B).

To gain further insight into the activated TIL populations, we profiled precursor‐exhausted CD8^+^ T‐cells (or T_PEX_) hallmarked by the combined expression of TCF1 (a stem−/memory‐like marker) and PD1, and lack of TIM3 expression (as a terminally differentiated marker), and their progeny pool of terminally exhausted effector PD1^+^ CD8^+^ T‐cells (or T_EX_), defined by increased GrzB and high TIM3 levels, but lack of TCF1 expression (Fig EV2A) (Jansen *et al*, 2019). Both populations remained similar in WT and Atg5^BECKO^ mice (Fig 2D). However, terminally exhausted effector CD8^+^ T‐Cells from Atg5^BECKO^ produced higher amounts of GrzB as compared to WT mice (Fig 2E).

We then asked whether there was a causal relationship between increased CD8^+^ T‐cells and reduced tumor growth in Atg5^BECKO^ mice. We depleted CD8^+^ T‐cells by injecting αCD8^+^ antibodies and examined tumor growth in WT and Atg5^BECKO^ mice (Fig 2F). Depletion of CD8^+^ T‐cells (Fig EV2B) did not significantly affect B16‐F10 growth in WT mice, but reversed the beneficial effect on tumor burden and survival in Atg5^BECKO^ mice (Fig 2G and H), thus functionally implicating CD8^+^ T‐cells in the delayed tumor growth phenotype.

Together, these data suggest that loss of canonical autophagy genes in TECs restrains melanoma growth and the immunosuppressive TME by increasing the frequency of GrzB‐expressing effector CD8^+^ T‐cells.

### Autophagy fosters an immunotolerant status of TECs

**Figure 3:**
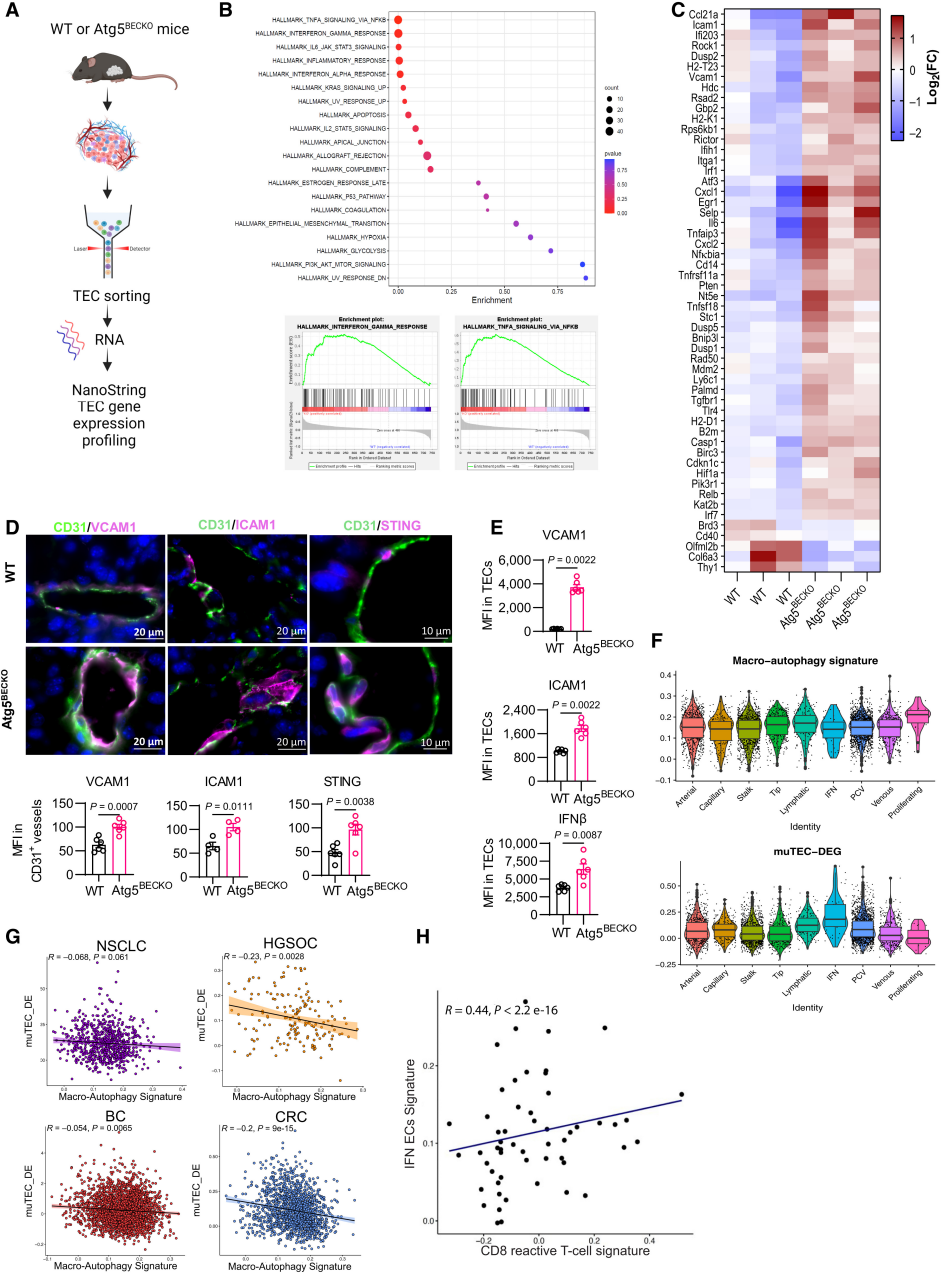
Autophagy represses inflammatory phenotype of tumor endothelial cells AExperimental design for end‐point investigation of tumor‐derived ECs.B, CNanoString analysis from TECs in subcutaneous B16‐F10 tumors from WT and Atg5^BECKO^ mice. (B) Gene set enrichment analysis (GSEA) analysis. (C) Heap map showing differentially expressed genes (Log_2_FC ≥ 0.7, *P* < 0.05).DRepresentative images and quantification (MFI) of immunofluorescence staining for VCAM1, ICAM1, and STING in CD31^+^ tumor endothelial cells from tumor sections of subcutaneous B16‐F10 tumors from WT and Atg5^BECKO^ mice. Scale bar represents 20 μm.EFlow cytometry analysis of surface expression of VCAM1, ICAM1, and intracellular IFNβ in TECs in subcutaneous B16‐F10 tumors from WT and Atg5^BECKO^ mice. Each point represents an individual tumor with *n* = 6 tumors per group.FExpression of macro‐autophagy signature and muTEC‐DEG across the 9 ECs subtypes (*n* Ecs = 4.921). Median of the data is displayed as solid black lines. Boxplots embedded in the violin plots were made by ggplot2 package in R. The lower and upper hinges correspond to the first and third quartiles. The upper whisker extends from the hinge to the largest value no further than 1.5 * IQR from the hinge (where IQR is the interquartile range). The lower whisker extends from the hinge to the smallest value (at most 1.5 * IQR of the hinge).GSpearman correlation between the expression of macro‐autophagy signature and mu‐TEC‐DE in ECs of primary tumors.HSpearman correlation between the expression of CD8^+^ reactive T‐cells signature (calculated on T‐cells) and IFN ECs signature (calculated on ECs) in each primary tumor sample. For immunofluorescence staining, at least 3 WT and 3 Atg5^BECKO^ mice were used for the quantification. (F–H) Samples from 48 cancer patients were used in the analysis. In particular, 31 from BC_early, 7 from CRC, 2 from HGSOC, and 8 from NSCLC_early. Data Information: All data represent mean ± s.e.m. Statistical differences were determined by two‐sided Student's *t*‐test (B–E) or (F–I), exact *P* value calculated by two‐sided Mann–Whitney test or two‐sided Wilcoxon matched‐pairs signed rank test. Only primary tumor samples of HGSOC, CRC, BC, and NSCLC are included in the analysis. Source data are available online for this figure.

**Figure EV3:**
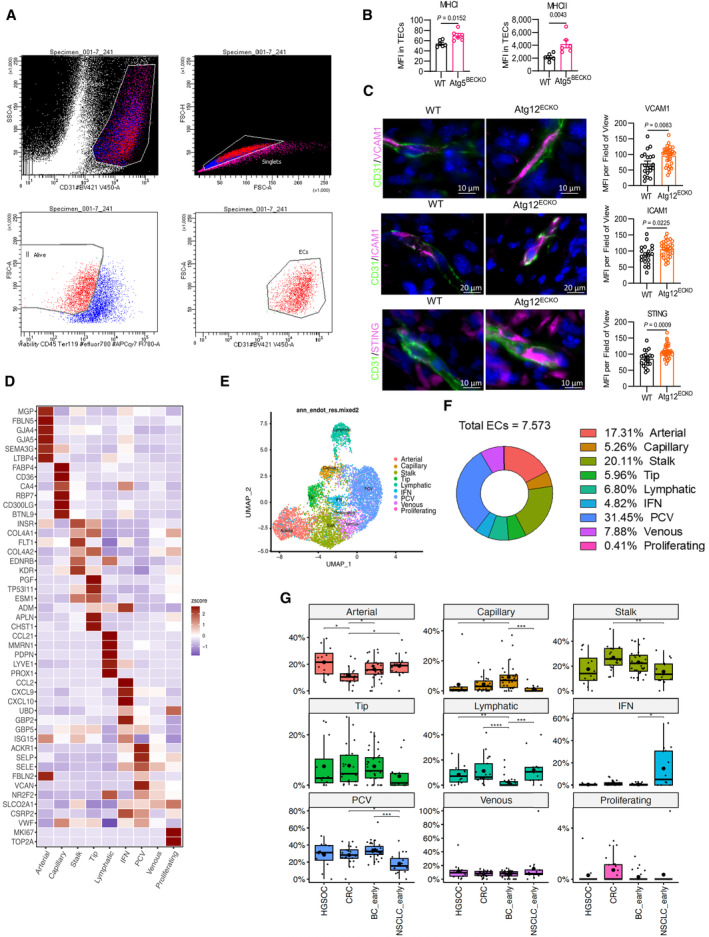
Phenotype of TEC from WT and Atg5^BECKO^ mice and human TEC subclusters from single‐cell RNA‐seq atlases AGating strategy for sorting of tumor endothelial cells from subcutaneous B16‐F10 melanoma from WT and Atg5^BECKO^ mice.BFlow cytometry analysis for the surface expression of MHCI and MHCII in CD31^+^ tumor endothelial cells derived in subcutaneous B16‐F10 tumor from WT and Atg5^BECKO^ mice. Each point represents an individual mouse, with *n* = 6 mice per group.CRepresentative images and quantification (MFI) of immunofluorescence staining for VCAM1, ICAM1, and STING in CD31^+^ tumor endothelial cells from tumor sections of subcutaneous YUMMER 1.7 tumors from WT and Atg12^ECKO^ mice. Scale bars represent 10 μm for VCAM1 and STING and 20 μm for IcAM1.D–GPreliminary analysis of publicly available single‐cell RNA‐seq atlases from primary tumors of treatment‐naïve patients. Genetic markers used to cluster EC subsets and their corresponding expression shown in a heatmap. BC, breast cancer; CRC, colorectal cancer; HGSOC, high‐grade serous ovarian carcinoma; NSCLC, non‐small cell lung cancer; PCV, post‐capillary venules.DHeatmap showing expression of representative marker genes across 9 ECs subtypes.EUMAP map of Ecs (*n* = 7.573) color coded for the indicated cell type.FPie chart showing the pan‐cancer relative abundance of the 9 Ecs subtypes.GRelative abundance of the 9 Ecs subtypes across tumor types included in the study. Boxplots embedded were made by ggplot2 package in R. The lower and upper hinges correspond to the first and third quartiles. The upper whisker extends from the hinge to the largest value no further than 1.5 * IQR from the hinge (where IQR is the interquartile range). The lower whisker extends from the hinge to the smallest value (at most 1.5 * IQR of the hinge). (D–G) Samples from 48 cancer patients were used in the analysis. In particular, 31 from BC_early, 7 from CRC, 2 from HGSOC and 8 from NSCLC_early. Data Information: For immunofluorescence staining, 2 WT and 3 Atg12^ECKO^ mice were used for the analysis. All data represent mean ± s.e.m. Statistical differences were determined using two‐sided Student's *t*‐test (B, C) or in (G), exact *P* values by two‐sided Mann–Whitney test or two‐sided Wilcoxon matched‐pairs signed rank test: *P < 0.05, **P < 0.01, ***P < 0.001.

Next, we asked how autophagy regulates lymphocyte influx and activity, focusing on lymphocyte‐regulating chemokines, cytokines, and adhesion molecules. As autophagy not only exerts direct effects via protein degradation but also regulates the transcriptional and epigenetic programs in cells, including ECs (Maes *et al*, 2014; Di Malta *et al*, 2019; Meçe *et al*, 2022), we initially conducted transcriptional profiling of FACs‐sorted ATG5‐proficent and ‐deficient CD45^−^Ter119^−^CD31^+^ TECs from B16‐F10 tumors (yielding more than 90% pure TECs) (Fig EV3A), with a panel of 770 genes implicated in cancer immunomodulation using the Nanostring technology (Fig 3A). The top hallmarks in our data set were related to inflammatory responses (Fig 3B). Moreover, analysis of differentially expressed (DE) genes with Log_2_FC ≥ 0.7 and *P* < 0.05, revealed a prominent expression of genes in Atg5^BECKO^ TECs encoding for surface inflammatory proteins with immune cell‐interacting activities and cytokines/chemokines, which are characteristics of an inflamed TEC phenotype (Fig 3C). Congruently, gene expression of factors implicated in the NF‐κB pathway and various EC adhesion molecules were significantly upregulated in TECs from Atg5^BECKO^ mice. The latter included *VCAM1*, *ICAM1*, *SELE*, and *SELP* with an established role in binding T‐cell surface ligands and in increasing T‐cell receptor signaling (Contento *et al*, 2010; Jankowska *et al*, 2018; Nagl *et al*, 2020), as well as cytokines and chemokines with chemoattractant and adhesion function in ECs such as: *IL6*, *CX3CL1*/*fractalkine*, *CXCL2*, *CXCL1*, and *RELB* a mediator of the noncanonical NF‐κB pathway (Fig 3C). Another set of DE genes annotated for the antiviral/ type 1 interferon (IFN) responses and included *IFIH1*, *IRF1*, *IRF7*, *TLR4* and elements of the antigen presentation machinery *H2‐K1*, *H2‐T23* and *H2‐D1*, suggesting that TECs from Atg5^BECKO^ mice have acquired improved immunomodulatory functions, including the capacity to sustain T‐cell function (Fig 3C). Gene set enrichment analysis (GSEA) confirmed that TECs from Atg5^BECKO^ mice compared to TECs from WT mice, exhibited a robust enrichment in gene sets involved in inflammatory response and IFN signaling pathways (Fig 3B). Thus, Atg5 KO in TECs evokes the expression of chemokines and molecules controlling lymphocyte adhesion and function.

We then validated the transcriptome results at the protein level for selected immunomodulatory markers. Compared to WT, TECs from Atg5^BECKO^ mice demonstrated a significant increase in the levels of VCAM1, ICAM1, and STING, a major regulator of the antiviral/ type 1 interferon responses (Zhang *et al*, 2022) (Fig 3D).

Congruently, the surface expression of VCAM1, ICAM1 (Fig 3E), MHC class I, and MHC class II (Fig EV3B) and the intracellular expression of IFNß – a downstream effector of the STING pathway (Zhang *et al*, 2022) (Fig 3E)—were elevated in TECs from Atg5^BECKO^ mice. A similar inflamed TEC phenotype was observed in YUMMER 1.7 melanoma grown in Atg12^ECKO^ mice (Fig EV3C). Thus, autophagy blockade boosts TEC inflammation in various melanoma models.

We next sought to more broadly contextualize our murine mRNA profiling to human TECs (huTECs), by analyzing phenotypic characteristics of ECs from publicly available single‐cell RNA‐seq (scRNA‐seq) cancer atlases. To increase coverage of rare huTECs subtypes, we pooled EC scRNAseq data from nonsmall cell lung cancer (NSCLC), breast cancer (BC), high‐grade serous ovarian carcinoma (HGSOC), and colorectal cancer (CRC), from different biopsy sites (primary tumors, metastasis, and adjacent non‐neoplastic tissue samples). Starting from a total of 7.573 high‐quality TECs, we performed cluster analysis across these tumors (Fig EV3D–G). HuTECs were clustered into nine distinct subtypes and validated using markers previously identified in human and murine ECs^11^ (Fig EV3D–F). These clusters included arterial (*MGP*, *FBLN5*, *GJA4*) and capillary ECs (*FABP4*, *CD36*, *CA4*), stalk cells *(INSR*, *COL4A1*, *FLT1*), tip cells (*PGF*, *TP53I11*, *ESM1*), lymphatic ECs (*PROX1*, *PDPN*, *LYVE1*), and ECs with an IFN response signature (*ISG15*, *CCL2*, *CXCL9*), suggesting their involvement in immune cell recruitment (Fig EV3D and E). Additionally, we distinguished venous from post‐capillary venules (PCV), the latter being the most abundant ECs cluster (Fig EV3F) and found a minor subcluster of proliferating ECs (proliferating; Fig EV3F). We observed a variation in different huTECs subtypes frequency across tumor types, for example, IFN huTECs were predominantly present in NSCLC (Fig EV3G). We validated that the “inflammatory” gene signature derived from the nanostring analysis contained genes conserved between mouse and human (Table EV1) and used this in‐house generated signature, dubbed muTEC‐DE, and the reactome‐macroautophagy signature (including *ATG5*, *ATG12*, and *ATG9* among various genes involved in canonical autophagy) (Jupe, 2015) for further analysis. Notably, the IFN huTEC subset, hallmarked by the highest expression of the muTEC‐DE signature, had the lowest enrichment in the autophagy signature (Fig 3F), suggesting that, particularly the subtype with more pronounced immunomodulatory function, concomitantly display low expression level of autophagy genes.

We further tested the association between the muTEC‐DE and autophagy signatures within huTECs of primary tumors, across cancer types. Remarkably, except for NSCLC, we observed a significant inverse correlation between these signatures in all other cancer types analyzed, albeit to a somewhat reduced extent in breast cancer (Fig 3G). Furthermore, enrichment in the muTEC‐DE signature in huTECs showed a positive correlation with the expression of a CD8^+^ reactive T‐cells signature, measured within T‐cells (Fig 3H).

Together these observations suggest that irrespective of the species (mouse or human) or tumor types, TECs with a low autophagic capacity have enhanced immunosupporting functions.

### Autophagy blunts the NF‐κB and cGAS‐sting inflammatory axis in ECs

**Figure 4:**
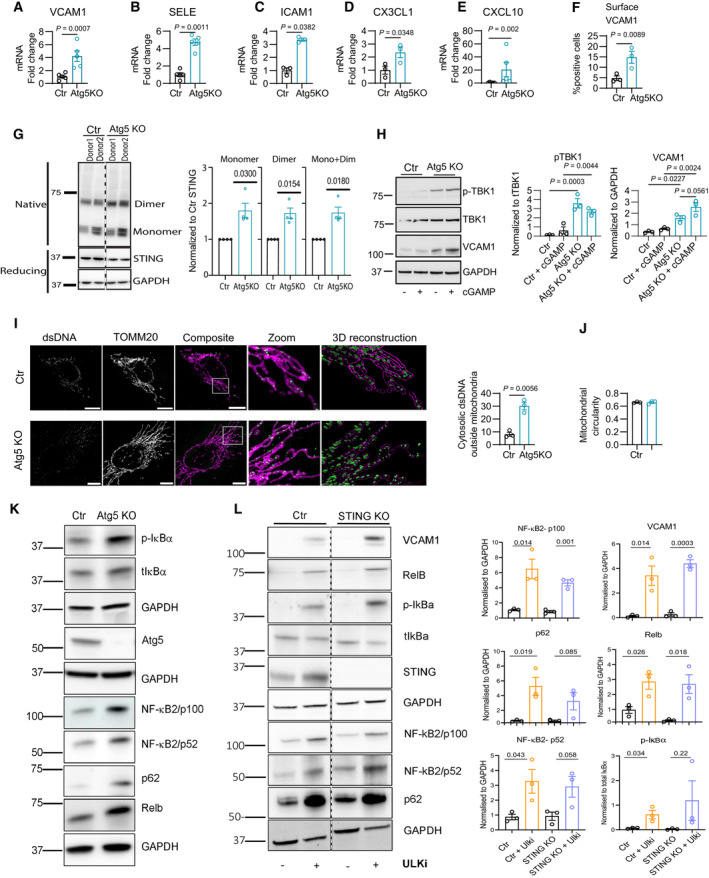
In cultured ECs autophagy curbs inflammation by the CGAS‐STING‐mediated NF‐κB pathway through the clearance of cytosolic dsDNA A–EmRNA expression of *VCAM1* (A), *SELE* (B), *ICAM1* (C), *CX3CL1* (D), and CXCL10 (E) in HUVECs after deletion of *Atg5*, each point represents a biological replicate, *n* = at least 3 biological replicates.FFlow cytometry analysis for surface expression of VCAM1 (% of live cells) in Ctr and Atg5KO HUVECs upon stimulation with IFNγ for 4 h, *n* = 3 biological replicates.GRepresentative western blot of STING in native and reducing conditions and quantification (monomers^+^ dimers) in Ctr and Atg5KO HUVECs, *n* = 4 biological replicates.HRepresentative western blot and relative quantification of pTBK1, tTBK1, and VCAM1 in Ctr and Atg5KO HUVECs treated with vehicle or 2.5 μg/ml STING agonist (2′3′‐cGAMP) for 24 h, *n* = 3 biological replicates.ISuperresolution Airyscan immunofluorescence images for TOMM20 and dsDNA (green—outside mitochondria, magenta—inside mitochondria) in Ctr and Atg5KO HUVEC. Scale bar represents 10 μm.JQuantification of mitochondrial circularity per cell in Ctr and Atg5KO HUVECs by super resolution Airyscan immunofluorescence images. Data from HUVECs was generated from at least three independent donors. In all images, scale bars represent 10 μm and at least 30 cells (10 per donor) were imaged per condition.KRepresentative western blot of p‐IκBα, total IκBα, Atg5, NF‐κB2 (p100 and p52), p62, and RelB in Ctr and Atg5KO HUVECs.LRepresentative western blot and relative quantification of NF‐κB2 (p100), VCAM1, p62, RelB, NF‐κB2 (p52), and p‐IκBα, in Ctr and STINGKO HUVECs treated or not with ULK1/2 chemical inhibitor (ULK1/2 inhi). Quantifications are from three biological replicates. All proteins are from the same set of lysates. When proteins were run on the same blot, cropping is denoted by a dashed line). Data information: All data show mean ± s.e.m. Statistical difference was determined using a two‐sided Student's *t*‐test (A–F, I, L) or one‐way Anova with Tukey corrections for multiple comparisons (G, J). Source data are available online for this figure.

**Figure EV4:**
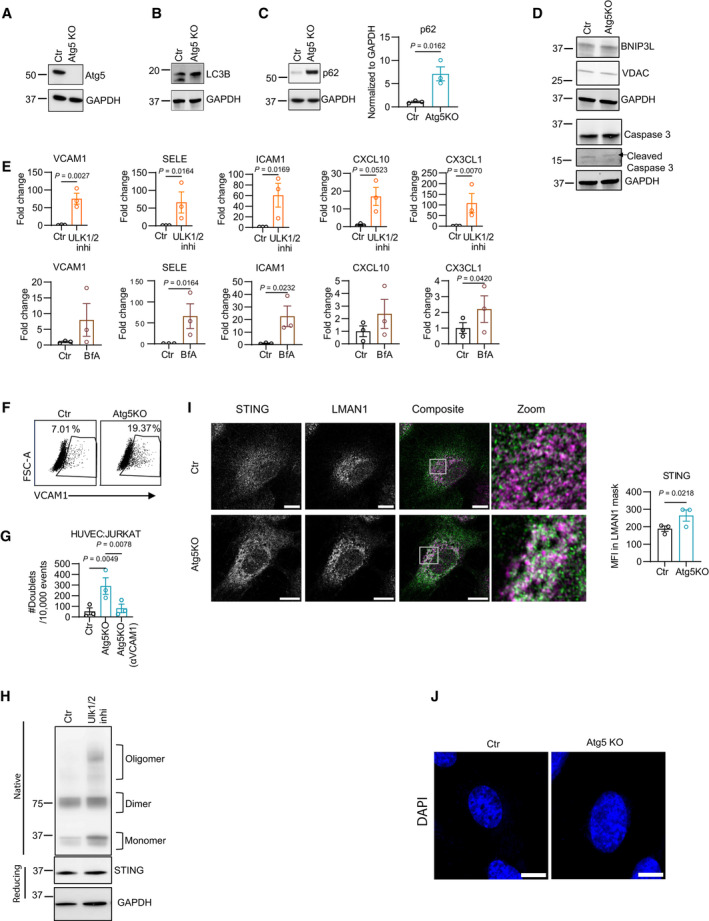
Autophagy blockade in HUVECs promotes formation of STING dimers and oligomers and accumulation in ERGIC A–CRepresentative western blot for Atg5 (A), LC3B (B), and p62 (C) and quantification for p62 in HUVECs nucleofected with scrambled (Ctr) or Atg5 specific guide RNA (Atg5KO) conjugated with cas9 protein. The quantification in C comes from 3 biological replicates.DWestern blot of BNIP3L, VDAC, caspase‐3, and cleaved caspase 3 in Ctr and Atg5KO HUvECs.EGene expression analysis of *VCAM1*, *SELE*, *ICAM1*, *CXCL10*, and *C3CXL1* in HUVECs treated with vehicle (Ctr) or ULK1/2 inhibitor (top) and vehicle (Ctr) or bafilomycin A (BfA) (bottom); *n* = 3 biological replicates.FGating strategy for flow cytometric analysis of surface expression of VCAM1 (% of live cells) in Ctr and Atg5KO HUVECs upon stimulation with IFNγ for 4 h.GImaging flow cytometry analysis for doublet formation (# doublets/10,000 events) in Ctr and Atg5KO HUVECs (stimulated with IFNγ and TNFα for 12 h) with JURKAT‐cells.HRepresentative western blot of Native and reducing conditions for STING in HUVECs treated with vehicle or ULK1/2 inhibitor.ISuper‐resolution Airyscan immunofluorescence images for STING (green) and LMAN1 (magenta) proteins in Ctr and Atg5KO HUVEC. Scale bars represent 10 μm and at least 30 cells (10 per donor) were imaged per condition.JSuper‐resolution Airyscan immunofluorescence images for DAPI in Ctr and Atg5 KO HUVECs. Scale bars represent 10 μm. Representative images from three independent experiments. Data information: Statistical differences were determined using two‐sided Student's *t*‐test.

We next aimed to identify the underlying mechanisms by which autophagy inhibition endorses the EC‐inflamed phenotype. We evaluated whether cultured human umbilical ECs (HUVECs) could recapitulate the effects of genetic loss of autophagy observed in mice. Deletion of *Atg5* by CRISPR‐Cas9 in ECs blunted the conversion of LC3BI to LC3BII and led to the accumulation of p62, indicating blockade of constitutive autophagic flux (Fig EV4A–C). No sign of caspase activation was observed under baseline/replete conditions (Fig EV4D) as observed in our previous study (Maes *et al*, 2014). Compared to control ECs (Ctr), ATG5Ko ECs displayed enhanced expression of a largely similar set of inflammatory genes (e.g., *SELE*, *VCAM1*, and *ICAM1)* and chemokines (e.g., *CX3CL1*, *CXCL10*) as observed in the nanostring analysis of muTECs isolated from Atg5^BECKO^ mice (Fig 4A–E). Similar results were obtained with a ULK1/2 kinase inhibitor or by increasing the lysosomal pH through bafilomycinA (Fig EV4E), suggesting that under baseline conditions, inflammation in ECs is downregulated at the transcriptional level via the canonical lysosomal‐autophagy pathway. Differences in the extent of inflammatory gene expression profiles (Figs 4A–E and EV4E) are likely explained by the acute inhibition of ULK1/2 versus the chronic deletion of *Atg5* in these cells. Increased surface expression of VCAM1 in Atg5KO cells was further validated by FACs (Figs 4F and EV4F). To gain more functional evidence, we then tested the EC adhering ability of αCD3/CD28‐activated JURKAT‐cells in co‐culture with WT or ATG5Ko ECs. In this setting, cytokines (i.e., IFNγ/TNFα) were supplemented in the EC milieu to further promote surface translocation of adhesion molecules (Xia *et al*, 1998; Ozaki *et al*, 1999). When co‐cultured with JURKAT‐cells, *Atg5*‐deficient ECs displayed an increased formation of higher JURKAT/EC doublets in a VCAM1‐dependent manner indicating that *A*tg5 KO promotes a more proficient interaction of EC with T‐cells (Fig EV4G).

Given the similarity of the *in vivo* and *in vitro* phenotypes, we used cultured autophagy‐depleted ECs to investigate the molecular pathways that exacerbate inflammatory signaling. We focused on the STING and NF‐κB signaling pathways because STING activation is known to foster antitumor immunity by co‐regulating type I IFN and NF‐κB activation (Demaria *et al*, 2015; Yum *et al*, 2021; Zhang *et al*, 2022), which are enhanced in muTECs of melanoma‐bearing Atg5^BECKO^ mice (Fig 3C and D). Moreover, while autophagy is known to regulate both STING and NF‐κB pathways (Deretic, 2021), in ECs, depletion of STING improved the recruitment (however, not the adhesion) of T‐cells in response to TNF‐induced inflammation (Anastasiou *et al*, 2021).

In the canonical STING activation pathway, cGAS binds to cytosolic dsDNA and catalyzes the synthesis of cGAMP. The binding of cGAMP to ER‐associated STING triggers its oligomerization and exit/release from the endoplasmic reticulum (ER) to the ER‐Golgi intermediate compartment (ERGIC) (Hiller & Hornung, 2015), where oligomerized STING mediates the activation/phosphorylation of TBK1. This, in turn, leads to the transcription of interferon‐stimulated genes and activation of NF‐κB (Yum *et al*, 2021). Signal cessation is orchestrated by STING trafficking from the post‐Golgi to lysosomes, where STING is degraded (Gonugunta *et al*, 2017).

We first measured the effects of perturbations of the autophagy/lysosomal degradation pathway on STING trafficking and signaling. In resting conditions, blockade of autophagy either by *A*TG5KO (Fig 4G) or pharmacological inhibition of both ULK1/2 kinase activity (Fig EV4H) led to an incremental increase in the monomeric and dimeric/oligomeric forms of STING, revealed by immunoblotting in non‐denaturing conditions. Compared to control (Ctr) cells, *A*TG5 KO in ECs increased granular STING staining in the perinuclear regions, a hallmark of STING activation and signaling (Fig EV4I) (Parkes *et al*, 2021). Congruently, in ATG5KO ECs, STING displayed an increased co‐localization with LMAN1 (a marker of the ERGIC) (Fig EV4I), which was associated with TBK1 phosphorylation (Fig 4H), suggesting its activation. In line with this, addition of cGAMP, the product of the cGAS enzymatic activity, to ECs slightly but not significantly elevated p‐TBK1 and VCAM1 in Ctr ECs, whereas it had no additional effects on ATG5KO ECs (Fig 4H).

We further explored the possibility that autophagy could stimulate the clearance of cGAS activating factors, such as cytosolic dsDNA originating from mitochondria, which is an established endogenous trigger of STING signaling (Liu *et al*, 2016; Maekawa *et al*, 2019). Autophagy can inhibit type I IFN in cells undergoing mitochondrial outer membrane permeabilization (MOMP) driven by bona fide apoptotic signals (Lindqvist *et al*, 2018; Riley *et al*, 2018; Yamazaki *et al*, 2020). Super‐resolution microscopy using TOMM20 to stain the mitochondrial network and an antibody against dsDNA revealed that, compared to Ctr ECs that displayed dsDNA encapsulated within the mitochondrial network (Fig 4I), dsDNA in ATG5‐KO ECs was mostly found in close proximity to the mitochondrial network and partly in the cytosol (Fig 4I), suggesting its efflux through mitochondrial‐derived vesicles (McArthur *et al*, 2018). Since we did not observe presence of micronuclei in autophagy‐deficient cells (Fig EV4J), this result suggests that cystolic dsDNA may be of mitochondrial origin. Although ATG5 KO in ECs compared to Ctr ECs did not induce evident changes in key mitochondrial morphometric parameters (Fig 4J) or alterations in mitochondrial protein amount (Fig EV4D) as a measurement of mitochondrial clearance; we cannot exclude that a minority of mitochondria undergo MOMP‐mediated mitochondrial dsDNA release (Yamazaki *et al*, 2020; Kim *et al*, 2023), or other forms of mitochondrial stress, when autophagy is dysfunctional. Also, given that STING harbors an LC3‐interacting region (LIR) and can be targeted for autophagic degradation (Prabakaran *et al*, 2018; Pan *et al*, 2023), we cannot exclude that this mechanism contributes to the STING signal observed in *Atg5*‐depleted ECs. Notwithstanding, these results suggest that loss of autophagy in ECs mediates cGAS‐STING and NF‐κB activation, resulting in the expression of a repertoire of inflammatory/immunomodulatory target genes. Congruently, *Atg5* ablation in ECs, along with the upregulation of VCAM1, led to the phosphorylation of the inhibitory IκBα protein (RelA) of the canonical NF‐κB pathway, and increased the accumulation of mediators of alternative NF‐κB signaling (Yu *et al*, 2020); the precursor protein p100, its degradation product p52, and RelB, suggesting the activation of both NF‐κB pathways (Fig 4K) (Ghosh & Wang, 2021). This is in line with the upregulation of multiple regulators of the NF‐κB pathways, including *RELB*, upon deletion of *Atg5* in murine TEC (Fig 3C).

We then assessed whether cGAS‐STING was required for NF‐κB activation in autophagy‐depleted ECs. To this end, we CRISPR‐Cas9 deleted STING in Ctr and in Atg5KO cells. However, the effects of the concomitant and chronic suppression of Atg5 and STING varied across human EC donors making results unreliable. We thus tested the effects of ULK1/2 inhibition in STING‐depleted cells. In analogy with ATG5KO, ULK1/2 inhibition elicited the upregulation of VCAM1, IκBα‐phosphorylation, the accumulation of p100, RelB and p52, and autophagic receptor p62 (Fig 4L). The effects of ULK1/2 inhibition on these effectors were still maintained in STINGKO ECs (Fig 4L), suggesting that STING is dispensable for the activation of NF‐κB pathways in autophagy‐depleted ECs.

In summary, endothelial autophagy blockade enhances the immunosupportive/inflammatory TEC phenotype by the activation of both STING‐dependant and STING‐independent NF‐κB pathways.

### Loss of Atg5 in TECs sustains NF‐κB‐driven inflammation independent of sting

Previous studies, using conditional whole‐body STING KO mice or STING activation by intratumoral injection of agonists, proposed that vascular STING expression and Type I IFNs promote anti‐tumor immunity (Demaria *et al*, 2015; Yang *et al*, 2019). Considering that species‐specific mechanisms control the strength and specificity of STING downstream signaling responses (Mann *et al*, 2019), we sought to delineate the contribution of STING signaling to melanoma growth control in WT and Agt5^BECKO^ mice.

**Figure EV5:**
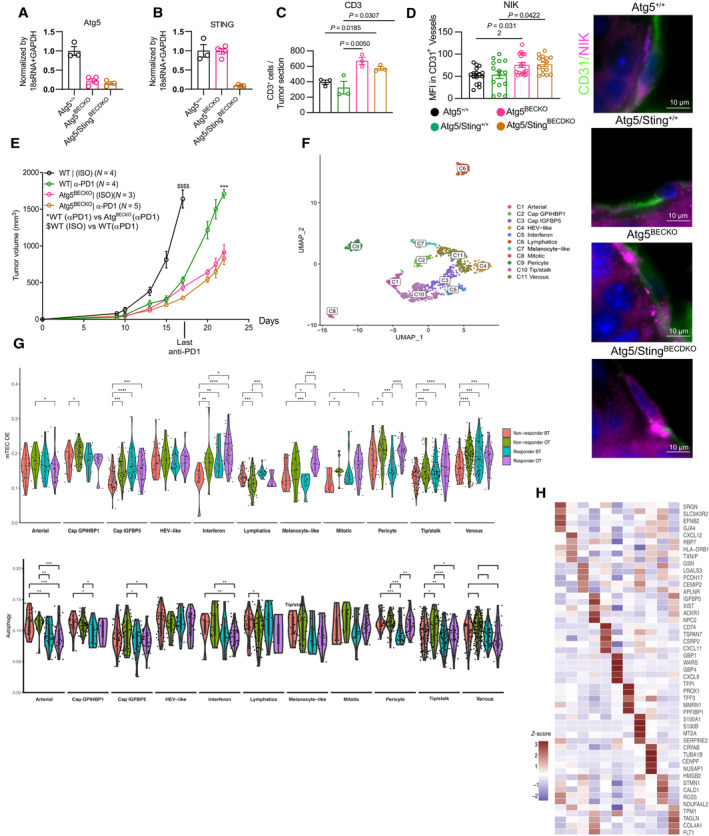
Analysis of genetic deletion of *Sting* and *Atg5* in TECs and interferon subtype of ECs from patients responding to αPD1 therapy A, BGene expression for *ATG5* (A) and *STING* (B) in CD31^+^ tumor endothelial cells sorted from subcutaneous B16‐F10 melanoma tumors from WT, Atg5^BECKO^ and Atg5/STING^BECDKO^ mice. For *ATG5*, forward primer was designed to bind in Exon 3 region and reverse primer was designed to bind in Exon 4 region. For STING, forward primer designed to bind in Exon 2 region and reverse primer was designed to bind in Exon 3 region, at least 3 mice per group were used for the analysis.CQuantification of immunofluorescence staining for CD3^+^ T‐cells in the tumor sections of subcutaneous B16‐F10 tumors from Atg5^+/+^, Atg5/Sting^+/+^, Atg5^BECKO^, and Atg5/Sting^BECDKO^ mice at least 3 mice per group were used for the analysis.DRepresentative images and quantification (MFI) of immunofluorescence staining for CD31 and NIK in the tumor sections of subcutaneous B16‐F10 tumors from Atg5^+/+^, Atg5/Sting^+/+^, Atg5^BECKO^ and Atg5/Sting^BECDKO^ mice, at least 3 mice per group were used for the analysis.EGrouped tumor volume showing Mean and s.e.m. of B16‐F10 subcutaneous tumor bearing WT and Atg5^BECKO^ mice injected with isotype (ISO) or αPD1 antibody. Data is from 1 representative experiment with at least *N* = 3 mice per group.FUMAP map of ECs (*n* = 7.573) color‐coded for the indicated cell type.GGene enrichment score of muTEC‐DE geneset (top) and autophagy geneset (bottom) across different subsets of huTECs from treatment naïve stage III/IV melanoma patients receiving anti‐PD1 based therapy monotherapy (nivolumab). Each point is 1 EC. ECs come from *n* = 10–11 patients per group. Boxplots embedded in the violin plots were made by ggplot2 package in R. The lower and upper hinges correspond to the first and third quartiles. The upper whisker extends from the hinge to the largest value no further than 1.5 * IQR from the hinge (where IQR is the inter‐quartile range). The lower whisker extends from the hinge to the smallest value (at most 1.5 * IQR of the hinge).HHeatmap showing expression of representative marker genes across 11 ECs subtypes. Data information: All data show mean ± s.e.m. Statistics were done using one‐way Anova with Tukey corrections for multiple comparisons (A–E). All statistical analyses on single cell were performed ggpubr package in R. Wilcoxon test was used for (G), *P < 0.05; **P < 0.01; ***P < 0.001; ****P < 0.0001.

To this end, we crossed *Sting* KO mice (Jin *et al*, 2011) with Atg5^BECKO^ mice to generate EC specific conditional *Atg5*/*Sting* double knockout (Atg5/STING^BECDKO^) mice (Fig EV5A and B).

We then injected B16‐F10 melanoma in Atg5^+/+^ mice, Atg5/STING^+/+^, Atg5^BECKO^, and Atg5/STING^BECDKO^ mice, monitored tumor growth and inspected the TEC phenotype by immunofluorescence for the presence of proinflammatory/immunomodulatory factors.

**Figure 5:**
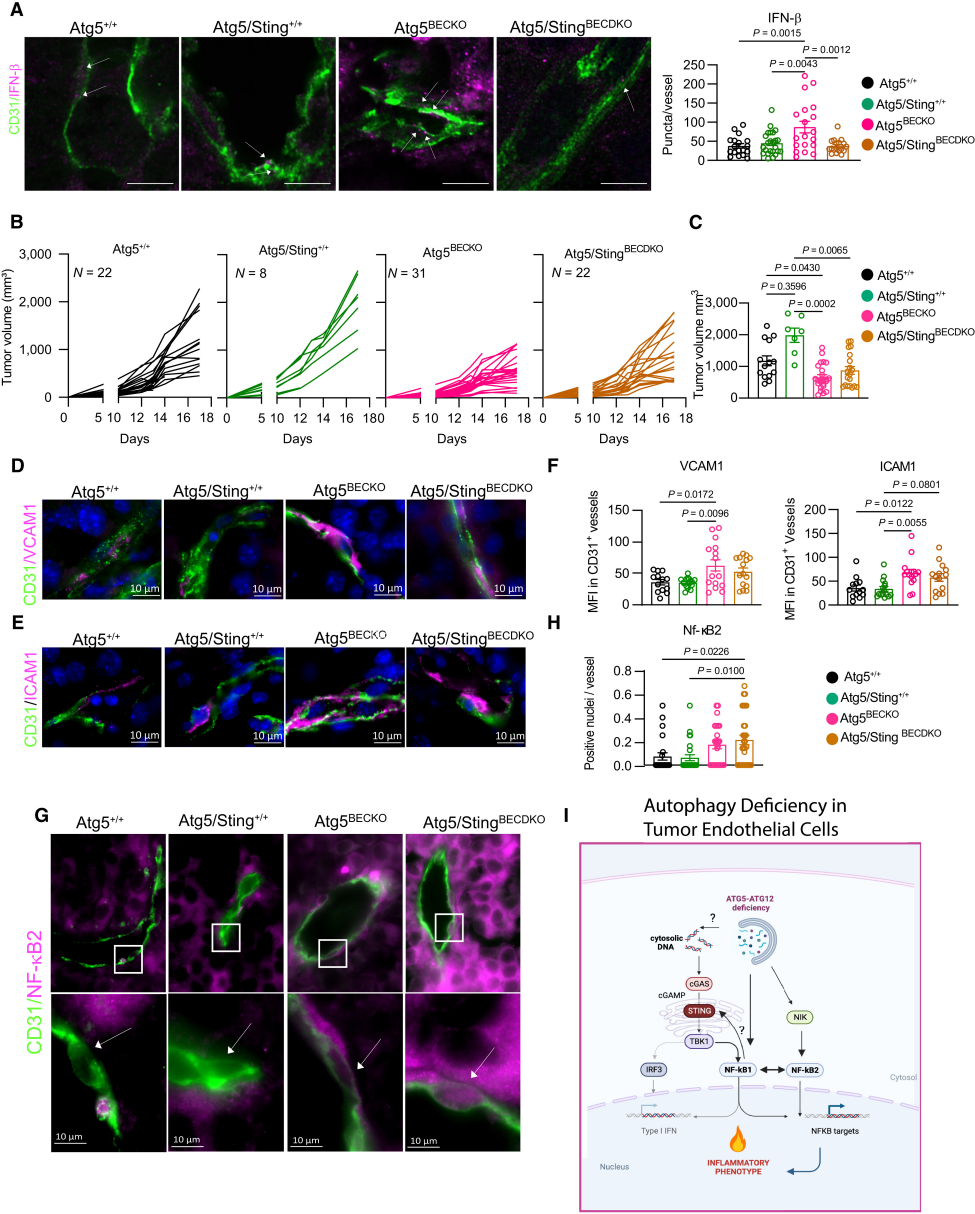
Dual genetic deletion of *Sting* and *Atg5* in TECs promotes the activation of the alternative NF‐κB pathway ARepresentative images and quantification (puncta per vessel) of immunofluorescence staining for IFNβ in the tumor sections of subcutaneous B16‐F10 tumors from Atg5^+/+^, Atg5/Sting^+/+^, Atg5^BECKO^, and Atg5/Sting double KO (Atg5/Sting^BECDKO^) mice. Scale bar represents 10 μm. Each point represents an individual vessel, *n* = at least 3 mice per group.B, CIndividual tumor volume (B) and end‐point tumor volume (C) of Atg5^+/+^, Atg5/Sting^+/+^, Atg5^BECKO^, and Atg5/Sting^BECDKO^ mice subcutanesouly injected with B16‐F10 melanoma cells. Each point represents an individual mouse, *n* = at least 7 mice per group. Figures are pooled from two independent experiments (B–C).D–FRepresentative images and quantification (MFI) of immunofluorescence staining for CD31 (D, E), VCAM1 (D), ICAM1 (E) in the tumor sections of subcutaneous B16‐F10 tumors from Atg5^+/+^, Atg5/Sting^+/+^, Atg5^BECKO^ and Atg5/Sting^BECDKO^ mice.G, HRepresentative images and quantification (positive nuclei) of immunofluorescence staining for CD31 and NF‐𝛋B2 in the tumor sections of subcutaneous B16‐F10 tumors from Atg5^+/+^, Atg5/Sting^+/+^, Atg5^BECKO^, and Atg5/Sting^BECDKO^ mice. Scale bar represents 10 μm.ISchematic of proposed model for inflammatory phenotype downstream of autophagy inhibition. Data Information: For immunofluorescence staining, at least 3 mice per group were used for the quantification. All data show mean ± s.e.m. Statistical differences were determined by one‐way Anova with Tukey corrections for multiple comparisons. Source data are available online for this figure.

The granular expression of IFNß, a major downstream target of STING, was elevated in TECs from melanoma‐bearing Atg5^BECKO^ mice, whereas its expression was blunted in TECs of Atg5/STING^BECDKO^ mice and comparable to WT TECs (Fig 5A), functionally validating the TEC phenotype of the DKO mice.

Melanoma growth (Fig 5B and C) was significantly reduced in both Atg5^BECKO^ and Atg5/STING^BECDKO^ mice and did not differ in the recruitment of intratumoral CD3^+^ T‐cells (Fig EV5C). Moreover, both Atg5^BECKO^ and Atg5/STING^BECDKO^ TECs displayed elevated levels of VCAM1 and ICAM1 adhesion molecules compared to their respective WTs (Fig 5D–F). Since VCAM1 and ICAM1 are shared downstream targets of the canonical and alternative NIK‐mediated RelB/p52 pathways (Yu *et al*, 2020) (Figs 3C and 4K and L), we analyzed the levels of NIK and of the nuclear localized/active p52 transcription factor in TECs. Compared to their respective WT counterparts, TECs from both Atg5^BECKO^ and Atg5/STING^BECDKO^ displayed increased levels of NIK (Fig EV5D). Interestingly, concomitant EC‐specific deletion of both *Atg5* and *Sting* (Atg5/STING^BECDKO^ mice) (Fig 5G and H) exhibited a trend toward further increase in vessels with p52‐positive nuclei when compared to Atg5^BECKO^ mice, suggesting that the alternative NF‐κB pathway was heightened in the absence of STING, in sync with the *in vitro* data (Fig 5I).

Together, these data posit that STING contributes to the exacerbating NF‐κB‐dependent immunosupportive Atg5^BECKO^ phenotype, but is not essential.

### Tumor endothelial autophagy limits melanoma responses to anti‐PD1 therapy

**Figure 6:**
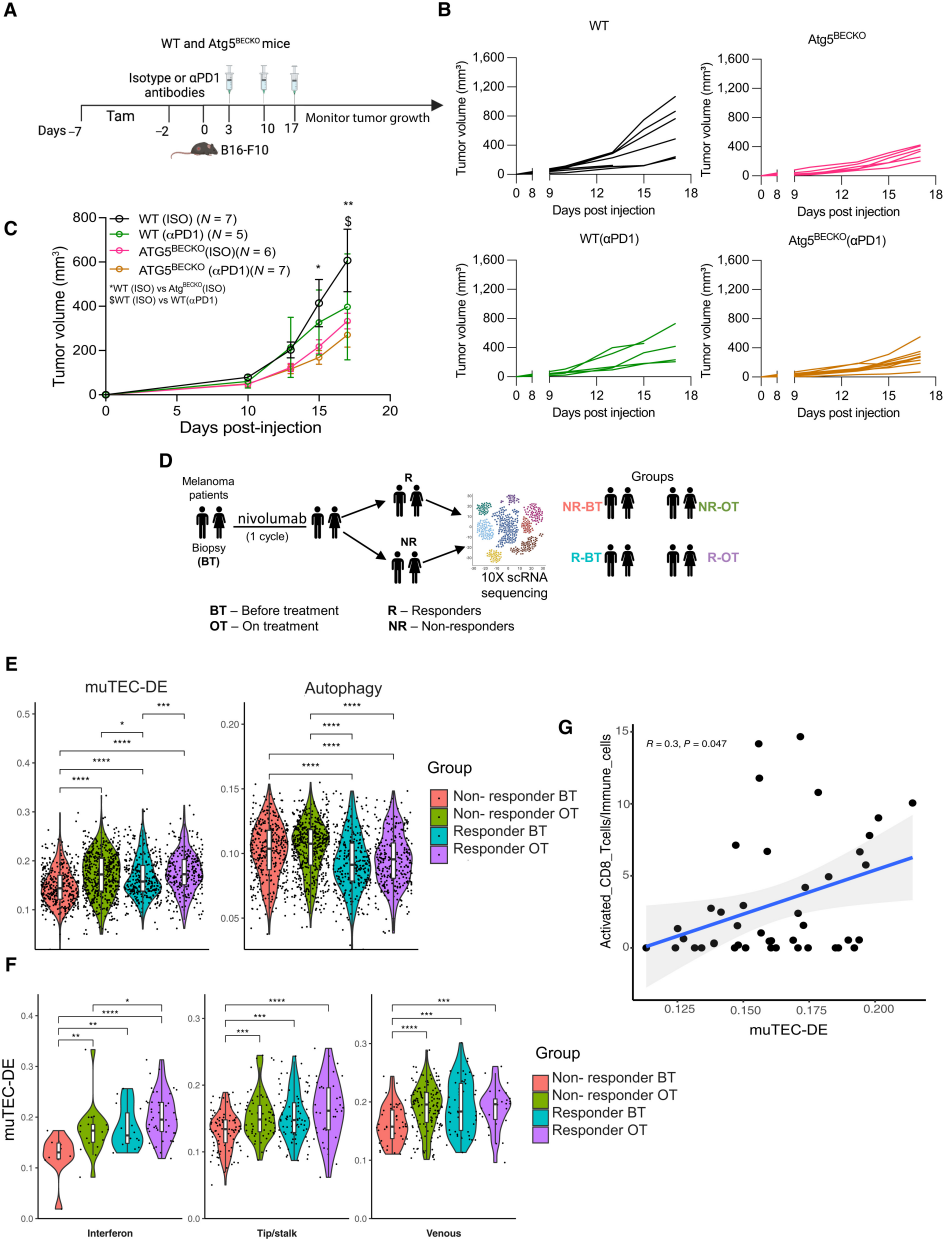
TEC‐autophagy negatively correlates to the response to anti‐PD1 therapy in melanoma ASchematic representation for treating subcutaneous B16‐F10 tumor‐bearing WT and Atg5^BECKO^ mice with αPD1 therapy.B, CIndividual tumor volume (B), grouped tumor volume (C) of B16‐F10 subcutaneous tumor bearing WT and Atg5^BECKO^ mice injected with isotype (ISO) or αPD1 antibody. Data shown in (B, C) are from 1 representative experiment.DStudy design for single‐cell transcriptome analysis of huTEC from treatment naïve stage III/IV melanoma patients receiving anti‐PD‐1‐based therapy monotherapy (nivolumab).E, FscRNA‐seq data analysis from huTECs in melanoma. Each point is 1 Cell. ECs and T‐cells come from *n* = at least 10 patients per group.EAUCell enrichment score of muTEC‐DE (Table EV1) and autophagy (Reactome database, R‐has‐1632852: Macroautophagy) genesets in all ECs.FAUCell enrichment score of mTEC DE geneset in 3 EC subtypes.E–FBoxplots embedded in the violin plots were made by ggplot2 package in R. The lower and upper hinges correspond to the first and third quartiles. The upper whisker extends from the hinge to the largest value no further than 1.5 * IQR from the hinge (where IQR is the inter‐quartile range). The lower whisker extends from the hinge to the smallest value (at most 1.5 * IQR of the hinge.).GCorrelation between AUCell score of mTEC DE geneset in TECs and percentage of activated CD8 T‐cells in each sample. *N* = 43 patients. Data Information: All data represent mean ± s.e.m. (B, C). Statistical differences were determined by two‐way Anova with multiple comparisons *$*P* < 0.05, **P < 0.01. Wilcoxon test was used for (E–G), *P < 0.05; **P < 0.01; ***P < 0.001; ****P < 0.0001. Source data are available online for this figure.

Given the immune‐stimulating role of autophagy blockade concomitant with enhanced CD8 T‐cell infiltration and activity, we then inquired whether impairing autophagy in TECs can boost immunotherapy in the form of immune‐checkpoint blockade (ICB) (Fig 6A).

While anti‐PD1 monotherapy significantly reduced B16‐F10 tumor growth in WT mice (Fig 6B and C), the effect of immunotherapy in B16‐F10 bearing Atg5^BECKO^ mice was incremental but not significant (Fig 6B and C). After cessation of anti‐PD1 therapy tumor burden in ICB‐treated melanoma‐bearing WT mice rapidly resumed (Fig EV5E), whereas in Atg5^BECKO^ treated with anti‐PD1, or their isotype Atg5^BECKO^ controls, tumor burden was more efficiently contained (Fig EV5E). These results suggest that autophagy blockade in TEC of melanoma‐bearing mice, by favoring recruitment and activity of TILs (Fig 2D–G), does not further enhance the efficacy of anti‐PD1 ICB therapy, but may further sustain its effects.

Our previous analysis indicated that particularly, the huTEC interferon subtype of treatment‐naïve cancer patients, exhibited a divergent association between the expression of muTEC‐DE^High^ and autophagy^Low^ gene signatures (Fig 3F). To further portray the significance of these TEC‐specific signatures and their association with clinical responses of melanoma patients to ICBs, we then interrogated the single‐cell transcriptome of huTECs using scRNAseq data from a unique prospective longitudinal study, including treatment naïve stage III/IV melanoma patients receiving anti‐PD1‐based therapy. Tumor biopsies were collected before (BT) treatment and right after the first cycle of treatment (OT) and processed for scRNAseq analysis.

Data consisted of TECs from 22 samples, *N* = 12 responders (R), and *N* = 10 non‐responders (NR) (Fig 6D). Overall, we annotated 1,541 ECs using established methods for unbiased EC identification (Goveia *et al*, 2020). Of note, the melanoma EC dataset included several EC cell types which were absent from the other cancer types (Fig EV3D–F). Melanocyte‐like cells are unique to melanoma and may exist due to vascular mimicry. In addition, pericytes were included in this dataset (Fig EV5F and H). Compared to nonresponders (NR), huTEC from responders (R) showed a significant enrichment of the core inflamed gene sets (muTEC‐DE) before (BT) treatment (Fig 6E), while showing a lower enrichment in the autophagy gene core signature. Interestingly, nonresponders (NR) also showed an enrichment in the muTEC‐DE signature after one cycle of treatment (Fig 6E). This might suggest that a TEC inflammatory phenotype is associated with an initial (yet not long‐lasting and therapeutically inefficient) change in the TME driven as a first response to anti‐PD1. However, in the same set of patients, the higher autophagy score was unchanged (Fig 6E), suggesting that only the muTEC^High^/autophagy^Low^ status is associated with clinical benefits.

We then interrogated whether muTEC‐DE signature differed between responders (R) and non‐responders (NR) exclusively in huTECs subtypes. To do so, we clustered all huTECs and obtained 11 subtypes reflecting huTECs heterogeneity observed in primary pan‐cancer data (Fig EV5F (UMAP), H (heatmap)). Compared to nonresponders (NR), melanoma patients responding to anti‐PD1 harbored a significant enriched muTEC‐DE signature in the Interferon, Tip/Stalk, and Venous EC clusters, a trend that was stably maintained at the ON treatment time point in the interferon subtype. Of note, these same clusters in responders (R) showed a significant decrease in the expression of autophagy signature at the ON treatment timepoint (Figs 6F and EV5G). Furthermore, using these scRNAseq datasets, we found a positive correlation between the expression level of the muTEC‐DE signature in all TECs and the intratumoral infiltration of CD8^+^ T‐cells (Fig 6G).

Together, these data suggest that the muTEC‐DE^High^/autophagy^Low^ phenotype is clinically associated with a favorable response to anti‐PD1 therapy.

### Spatial proximity between inflamed vessels and CD8^+^ t‐cells correlates to response to anti‐PD1 therapy

**Figure 7:**
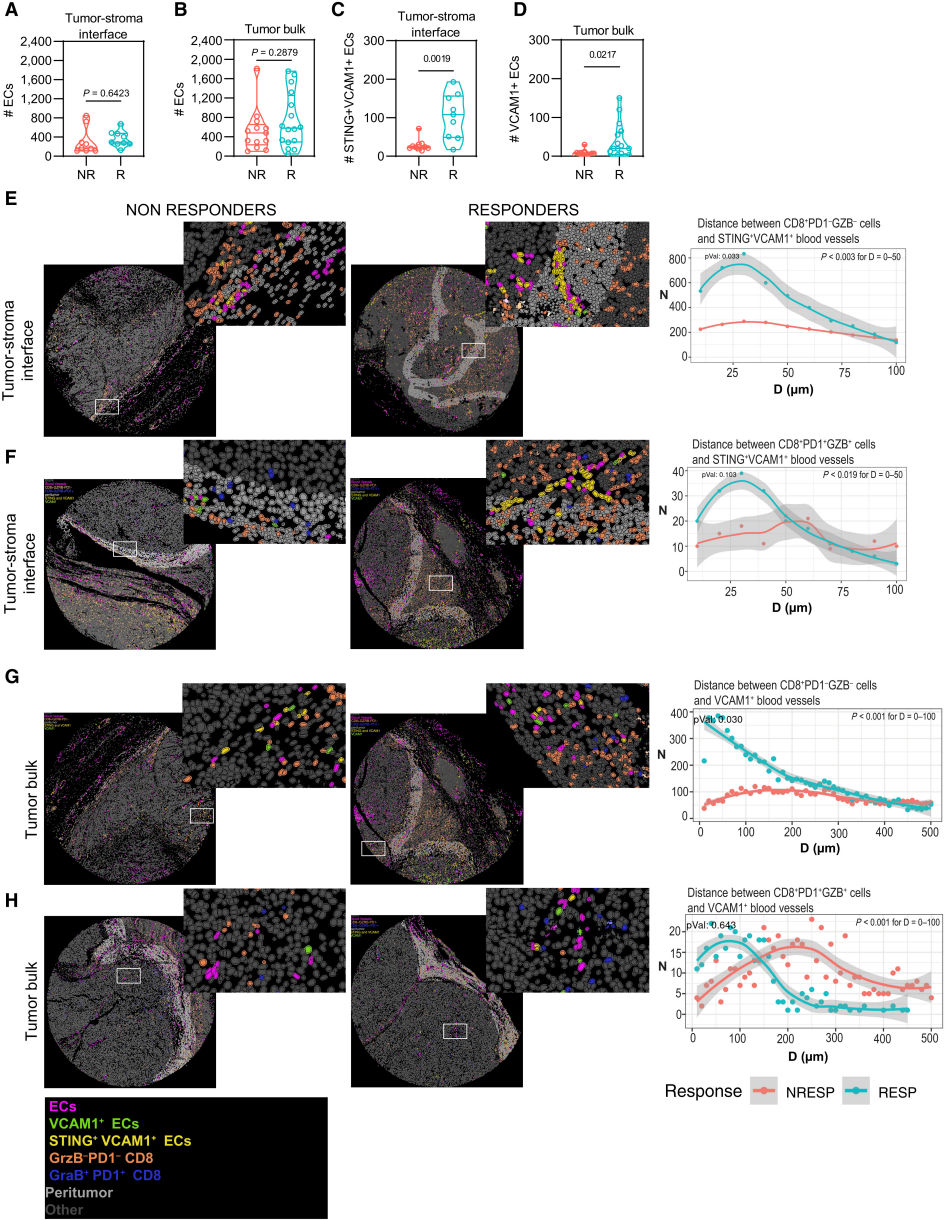
Responders to anti‐PD1 therapy house inflammed TECS with immune cell attracting properties A–DViolin plots comparing nonresponders (NR) and responders (R) in terms of (A) total number of ECs at the tumor–stroma interface, (B) total number of ECs in the tumor bulk, (C) number of VCAM1^+^STING^+^ ECs in the tumor–stroma interface, (D) number of VCAM1^+^ ECs in the tumor bulk. *n* = 6 patients per group.E–HRepresentative digital reconstruction of the tissue based on multiplex staining and cell clustering illustrating EC subtypes and CD8^+^ T‐cells subtypes. The results of the neighborhood analysis between ECs and CD8^+^ T‐cells subtypes are visualized by the line graph on the right. Neighborhood between ECs and CD8^+^ T‐cells subtypes is expressed as number of cells (*y* axis) located at a certain distance (*x* axis) from the ECs. All patient tissue samples are reused and came from a previously published study (Antoranz *et al*, 2022). Data Information: All data represent mean ± s.e.m. *P < 0.05, **P < 0.01, ***P < 0.01, ****P < 0.0001 using a two‐sided Student's *t*‐test (A–F). In neighborhood analysis of CD8^+^ T‐cells with ECs (E–H), two‐sided Student's *t*‐test was used at the particular distance. Source data are available online for this figure.

We then sought to portray the spatial relationship between inflamed huTECs as defined by their enhanced protein expression of VCAM1 or double‐positive VCAM1/STING and CD8^+^ T‐cells in melanoma samples. We performed multiplex immunohistochemistry with the multiple iterative labeling by antibody neodeposition (MILAN) technology (Cattoretti *et al*, 2019; Bolognesi *et al*, 2020) (Appendix Fig [Link], [Link], [Link]A–C) using a set of real‐life biopsies from melanoma patients undergoing anti‐PD1 monotherapy (Table EV2). The melanoma biopsy cohorts consisted of 6 responders (R) and 6 nonresponders (NR). CD31/AQP1 double‐positive (identifying huTECs), CD8^+^ PD1^−^ GrzB^−^ T‐cells (identifying naïve CD8^+^ T‐cells) and CD8^+^ PD1^+^ GrzB^+^ (identifying activated/effector CD8^+^ T‐cells) (Sade‐Feldman *et al*, 2018) were evaluated in three distinct spatial compartments of the biopsy, namely the “tumoral bulk,” the tumor–stroma interface, and the nontumoral areas. There was no difference in EC number between nonresponders (NR) and responders (R) in the tumor bulk and at the tumor–stroma interface (Fig 7A and B). We then investigated the proximity among these cell types in these spatial compartments by neighborhood analysis. In contrast to the nonresponders, responders showed a significant enrichment in VCAM1^+^ and VCAM1^+^STING^+^ TECs in the tumor bulk and at the tumor–stroma interface, respectively (Fig 7C and D). Further neighborhood analysis showed that responders had a significantly higher number of VCAM1^+^STING^+^ TECs in vicinity to both of CD8^+^ PD1‐GrZB^−^ and to CD8^+^ PD1^+^ GrZB^+^ T‐cells (Fig 7E and F). This observation was true for the tumor‐stroma interface and the tumor bulk. Particularly within the tumor bulk, even if in lower number, VCAM1^+^ vessels were still significantly closer to both subsets of CD8^+^ T‐cells, suggesting that an inflamed vessel phenotype maintain a more proficient crosstalk with infiltrated TILs (Fig 7G and H), even in the absence of STING. We also used LC3 antibody in the MILAN analysis, but due to limited resolution of the intracellular granular vs diffuse pattern of LC3 staining in TECs, we could not reliably assess their associated autophagy status.

Together, these results show that the spatial proximity between inflamed vessels with infiltrating CD8^+^ T‐cells is a hallmark of the TME of melanoma patients responding to anti‐PD1 therapy.

## Discussion

Despite the emerging evidence indicating that TECs are not just passive bystander regulators of immune cell trafficking, but play an active role in antagonizing antitumor immunity (Schaaf *et al*, 2018; Nagl *et al*, 2020; Amersfoort *et al*, 2022), the mechanisms controlling the immunosuppressive phenotypes of TECs remain largely unexplored. Gaining mechanistic knowledge of the processes that remodel the tolerogenic/anergic phenotype of TECs into a more proinflammatory/immunostimulatory status is thus key to improve immunosurveillance and immunotherapy responses. The primary focus of this study was to investigate the molecular mechanisms endorsing the tolerogenic status of TECs and to functionally assess the consequences of perturbing these mechanisms in melanoma antitumor immunity.

The data presented advance the concept that autophagy is a crucial vascular‐immune checkpoint that endorses TEC‐mediated immune cell barrier and immunoevasion.

First, through the EC‐specific ablation of assorted autophagy genes (*Atg5*, *Atg12*, and *Atg9)* involved in the early phases of autophagosome formation and expansion, we show that impairing autophagy reduces melanoma growth by promoting expansion of effector CD4^+^ and CD8^+^ T‐cells, thereby reducing the immunosuppressive TME. Second, we unveil that inhibiting TEC‐autophagy potentiates the proinflammatory/proadhesive and immunomodulatory functions of TECs, by exacerbating the canonical and alternative NF‐κB pathways, and by promoting STING signaling. Mechanistic and functional studies further highlight that NF‐κB activation is the major driver of the pro‐inflammatory TEC phenotype induced by autophagy loss. Third, by interrogating scRNAseq EC atlases of melanoma patients undergoing one cycle of anti‐PD1 immunotherapy, we show that TECs, hallmarked by an Inflammatory^High^/autophagy^Low^ phenotype, correlate with clinical responses to anti‐PD1. In line with this, via multiplex immunohistochemistry, we unravel that melanoma patients responding to anti‐PD1 therapy present inflamed vessels in close neighborhood with naïve and activated CD8^+^ T‐cells.

Autophagy has a well‐established role in suppressing homeostatic and pathological inflammation (Jounai *et al*, 2007; Deretic, 2021), due to its innate ability to clear intracellular (principally mitochondria‐derived danger signals) and extracellular (pathogenic) cargos (Deretic & Levine, 2018). It also regulates differentiation and metabolic programs in a highly context and cell/tissue‐specific manner (Deretic & Levine, 2018; Schaaf *et al*, 2018).

In cancer, recent studies disclosed that conditional whole‐body or hepatocyte‐specific knockout of *Atg5* or *Atg7* improved antitumor immunity by stimulating T cell‐mediated IFN‐γ, which led to an increase in MHC class I expression and antigen presentation of murine tumors with high mutational burden, through STING‐mediated immunity (Poillet‐Perez *et al*, 2020).

While these studies implicated the existence of multiple signaling circuitries controlled by host autophagy that regulate antitumor immunity, it remained unclear whether autophagy, specifically in the tumor endothelium, would be a major regulator of these immunosuppressive responses, and if it could affect the efficacy of immunotherapy.

Our transcriptomic profiling of TECs isolated from WT and Atg5^BECKO^ melanoma‐bearing mice, leveraged an inflammatory signature—dubbed muTEC‐DE—which was used to probe publicly available TEC taxonomies from different primary human tumor types. Using this in‐house generated signature across previously annotated TEC subtypes (Goveia *et al*, 2020; Hua *et al*, 2022), the enrichment of the muTEC‐DE was associated with the lowest expression levels of autophagy genes, particularly in the interferon EC subtype. This divergent association, which conforms with our functional data, raises the question of how particularly this subtype of TEC maintains a low transcriptional expression of autophagy genes. Recent scRNAseq studies obtained from different tumor types have highlighted the existence of multiple intermediate phenotypes of ECs in tumors indicating vascular plasticity within the TME (Rohlenova *et al*, 2020; Geldhof *et al*, 2022). One outcome of heterogeneous TECs could be TME niche‐specific responses to proinflammatory cues which are important for immunosurveillance. TEC autophagy, which is stimulated by the metabolically stressed TME, can therefore potentially alter EC specifications and the immunocontexture by niche‐specific interactions.

Furthermore, metabolic plasticity is an emerging trait of the phenotypic adaptation of TECs to their specific TME (Geldhof *et al*, 2022). Supporting a link between metabolism/epigenetic and IFN response, loss of the glycolytic enzyme PKM2 in ECs by altering the TCA cycle, promote DNA hypomethylation, de‐repression of endogenous retroviral elements, with the resulting activation of antiviral innate immune signaling (Stone *et al*, 2018). Hence, it is tempting to suggest that autophagy gene expression may be co‐regulated as a result of different metabolic/epigenetic circuits adopted by a specific EC phenotype. This is an outstanding question that warrants further investigation. Consistent with a potentially different outcome of autophagy deficiency in specific EC subsets, genetic loss of Atg5 in liver sinusoidal ECs stimulated VCAM1 expression and features of endothelial‐to‐mesenchymal transition, which accelerated liver inflammation and fibrosis, only when exposed to TNF (Hammoutene *et al*, 2020). While we cannot rule out that genetic loss of autophagy in non‐tumor ECs contributed to the tumor burden control we observed in melanoma‐bearing mice, it is possible that the presence of melanoma‐derived proinflammatory cytokines in the TME may further accentuate the effects of autophagy deficiency in TECs, as compared with noncancerous ECs of other vascular beds. A hypothesis that needs to be fully addressed in further studies.

In line with the results in murine melanoma and other human tumors, in treatment‐naïve melanoma patients the TEC subtype displaying the muTEC^High^/autophagy^Low^ phenotype was associated with clinical responses and with a higher number of tumor‐infiltrating CD8^+^ T‐cells, further suggesting the functional association between TEC‐autophagy and suppression of immune‐related responses. Furthermore, compared to nonresponders, responders to anti‐PD1 monotherapy had increased VCAM1^+^ STING^+^ vessel counts found in close vicinity to CD8^+^ T‐cells and the fraction of effector PD1^+^/GrzB^+^ CD8^+^ T‐cells, particularly at the tumor–stroma interface. A similar positive correlation was found with VCAM1^+^ vessel counts within the tumor bulk. These findings are congruent with the observation that elevated STING expression in tumor vessels from colorectal cancer patient biopsies correlated with intratumoral CD8^+^ T‐cell infiltration (Yang *et al*, 2019). A recent study across cancer types, including melanoma, indicated that an elevated frequency of CD8^+^ T‐cells within the stroma and invasive margin compartments had a better outcome than those in intra‐tumor compartments (Chen & Mellman, 2017; Li *et al*, 2021). However, more studies are needed to assess how the positioning of inflamed vessels within the tumor parenchyma models their immunomodulatory phenotypes (tolerogenic versus immunostimulatory) and whether the IFN‐EC subtype in responders is accrued at the tumor‐stroma interface to facilitate the infiltration of CD8^+^ T‐cells.

But which signals are critical for conveying the proinflammatory and antitumor effects of autophagy ablation in TECs? Recent studies have largely focused on the crosstalk between autophagy and STING pathways and their effects on cancer cell immunogenicity/antitumor immunity in response to therapy (Zhao *et al*, 2022; Lu *et al*, 2023). However, emerging evidence indicates that the homeostatic and pathological functions of autophagy in ECs are shaped by the cellular and tissue/microenvironment context (Reglero‐Real *et al*, 2021; Amersfoort *et al*, 2022; Meçe *et al*, 2022) and respond to different inflammatory cues. Previous studies specified that within the TME, TECs are early producers of Type I IFN, following intratumoral injection of cGAMP, and contribute via STING to the initiation of spontaneous and ICB‐induced antitumor immunity (Demaria *et al*, 2015). Exogenous, agonist‐mediated STING activation induces vascular reprogramming, likely caused by the reciprocal beneficial effects of tumor‐infiltrating CD8^+^ T‐cells on the tumor vasculature (Tian *et al*, 2017; Yang *et al*, 2019). However, monotherapy with STING agonist, which sustains Type I IFN signaling, may also elicit negative feedback mechanisms of resistance (Jacquelot *et al*, 2019), requiring the concomitant combination of antiangiogenic (anti‐VEGFR2) and ICBs (anti‐PD1 or anti‐CTLA4) to be effective (Yang *et al*, 2019).

While these studies highlight the relevant functions of TECs in antitumor immunity, they did not address the underpinning mechanisms of STING modulation specifically in ECs, nor the role of autophagy as a potential mitigation signal. We report that upon ablation of *Sting* in *Atg5*‐deficient TECs (ATG5/STING^BECDKO^), the stimulation of NF‐κB pathways maintains the proadhesive/inflammatory TEC phenotype. This likely accounts for the reduced tumor burden in Atg5/STING^BECDKO^ mice, to a similar extent to Atg5^BECDKO^ mice. While we cannot exclude that concomitant genetic loss of *Atg5* and *Sting* in muTECs have additional cell non‐autonomous effects, our *in vitro* and *in vivo* data, indicate that STING is not required for the activation of NF‐kB and is dispensable for the major inflammatory effects of autophagy inhibition in TECs, since in the absence of STING, NF‐κB pathways remain active. Interestingly, while STING is known to mediate the activation of NF‐κB by recruiting TBK1 (Yum *et al*, 2021), recent data posited that NF‐κB activation, by inhibiting microtubule‐mediated STING transport to the lysosome, promotes STING signaling in response to different signals (Zhang *et al*, 2023). These studies unravel the existence of a complex positive feedforward loop between these immunostimulatory pathways, which is likely regulated in a cell‐dependent manner. The exact mechanisms linking the possible co‐regulated activation of STING and NF‐κB proinflammatory pathways in response to endothelial autophagy inhibition need further exploration. The finding that the expression of *RELB* is upregulated in *Atg5*‐deficient TECs isolated from melanoma‐bearing mice, suggests a transcriptional mechanism. However, defects in the turnover of adaptors (Fliss *et al*, 2012; Paul *et al*, 2012; Zotti *et al*, 2014; Verzella *et al*, 2020) or elements of canonical and noncanonical NF‐κB pathways, caused by the accumulation of undegraded p62 or other autophagic adapter proteins, could support NF‐κB proinflammatory signals under TEC‐autophagy suppression (Yu *et al*, 2020). Notwithstanding, our results show that autophagy is able to repress two major immunomodulatory pathways, NF‐κB and STING, in TECs thus endorsing their immunosuppressive phenotype, with deleterious consequences for antitumor immunity and efficacy of ICBs. Our data underscore that autophagy is a persuasive TEC‐intrinsic immune‐evasion factor, which could be fostered in the tumor by the metabolically stressed TME to enforce immunosuppression (Debnath *et al*, 2023). This is likely the reason why the sole blockade of TEC‐autophagy reduces but does not prevent melanoma progression. Although a partial reversal of tumor vessel abnormalities was observed in our EC autophagy‐deficient melanoma‐bearing mice, this is possibly insufficient to induce *bona fide* vessel normalization which has been shown to synergize with immunotherapy regimens (Tian *et al*, 2017; Dong *et al*, 2023). Loss of autophagy in ECs may thus potentiate T cell function resulting in an additive—rather than synergistic—antitumor effect. Additional analysis of the effects of anti‐PD1 on tumor vessel normalization in wild‐type and EC autophagy‐deficient mice should help clarify this point. Furthermore, experiments including, e.g., antibody‐based IFNγ neutralization could be also performed to assess the (secondary) contribution of cytotoxic T cell‐derived IFNγ to the antitumor effects observed in Atg5^BECKO^ bearing mice (Zheng *et al*, 2018).

Finally, considering the broader translational relevance of our findings, this study supports the emerging concept that strategies aimed to suppress the anergic status of TECs are decisive in overcoming immunoevasion and improving efficacy of various immunotherapy approaches (Huinen *et al*, 2021). As found in other tumors (Yang *et al*, 2019), preclinical models of melanoma intratumoral STING agonist (i.e., ADU S‐100) enhance tumor‐infiltrating T‐cells and reduced melanoma growth (Demaria *et al*, 2015; Chelvanambi *et al*, 2021), through a process depending on the expression of STING in noncancerous cells. The results of our study advocate that TEC‐autophagy blockade, by the concomitant and independent activation of STING and NF‐κB pathways, offers a more efficient strategy to overcome immunoevasion in melanoma. Therefore, it will be important to scrutinize the still unappreciated effects of clinically used autophagy inhibitors (Mohsen *et al*, 2022) on the tumor vasculature. Our previous studies showed that the clinically used lysosomotropic drug chloroquine (CQ) exerted antimetastatic effects in melanoma by primarily normalizing the tumor vasculature through enhanced Notch signaling (Maes *et al*, 2014), but the effects of CQ on NF‐κB signaling in TECs were not explored. However, systemic administration of CQ could have additional and indirect effects on T‐cells or other stromal cells, making the clinical utilization of this lysosomotropic drug as a vascular autophagy inhibitor problematic.

In summary, this study advances our knowledge of the role of autophagy as a persuasive blood vessel‐intrinsic anti‐inflammatory/immunosuppressive mechanism, restricting antitumor immunity in melanoma. It also provides a theoretical foundation for identifying appropriate combination therapies utilizing vasculature‐homing tools (Lu *et al*, 2017) to target autophagy inhibitors or NF‐κB modulators (Lalle *et al*, 2021) to the tumor vasculature.

## Materials and Methods

### Cell culture

#### Primary cells

Human umbelical vein endothelial cells (HUVECs) from different donors were purchased from PromoCell. Cells were maintained in supplemented Endothelial Cell Growth Medium 2 kit (PromoCell#C‐22111) at 37°C under 5% CO2. Cells were only used at low passage.

#### Murine melanoma cell lines

B16‐F10 were purchased from the ATCC and HcMEL12‐mCherry cells were produced by Prof. Lukas Sommer (University of Zurich, CH) and were maintained in RPMI‐1640 (Sigma#R8758) medium supplemented with 10% FBS, 1 mM sodium pyruvate, and 10 mM HEPES at 37 °C under 5% CO2. Yummer 1.7 were purchased from Sigma‐Aldrich and maintained in DMEM/F12 medium (#DF‐041‐B) supplemented with 10% FBS and 1× non‐essential amino acids (#TMS‐001‐C) and pen/strep. All cell lines are kept at low passage number and checked for mycoplasma before vials are cryofrozen.

#### Inhibitors, agonists, and cytokines

ULK1 and ULK2 Kinase inhibitor MRT68921 (0.5 μM; Sigma#SML1644‐5MG), STING agonist 2′3’‐cGAMP (2.5 μg/ml; Invivogen#tlrl‐nacga23), TNFα (5 ng/m; R&Dsystems#210‐TA‐020), and IFNγ (25 IU/ml; Sigma#SRP3058‐100UG) were used as recommended by the supplier.

### CRISPR‐Cas9 gene knockout by nucleofection

Genes were deleted by nucleofecting (P5 Primary Cell 4D‐Nucleofector kit Lonza#V4XP‐5024). Ribonucleoprotein complexes (RNPs) consisting of pooled sgRNAs conjugated with Cas9. RNPs were prepared by co‐incubating 500 pmoles of sgRNA pools (for Atg5, cGAS, and STING) with 100 pmoles of SpCas9 2NLS (Synthego) and introducing the RNPs into HUVECs using CA‐167 program in Lonza 4D nucleofector. Cells recovered for 48 h before further experiments.

### Mice

All experimental animal procedures were conducted according to the European guidelines (Directive 2010/63/EU) and approved by the Institutional Animal Care and Research Advisory Committee of the KU Leuven (PC57BL6). Mice were housed in conventional ventilated cages in accordance with federal guidelines. Mice with tumors were checked at least 3 times a week to monitor tumor growth. Mice with blood endothelial cell‐specific deletion of *Atg5* were obtained by crossing Pdgfb‐Cre^ERT2^ Rosa26^tdTomato/tdTomato^ mice (Claxton *et al*, 2008) with previously generated *Atg5* ^fl/fl^ mice (Kuma *et al*, 2004). Pdgfb‐Cre+^ERT2^; Atg5^fl/fl^ mice were referred to as Atg5^BECKO^; their Cre‐negative littermates were named WT. Mice with pan endothelial cell‐specific deletion of Atg12 or Atg9a were obtained by crossing mice VeCadh‐cre^ERT2^ (Sörensen *et al*, 2009) with *Atg12* ^*f*l/fl^ (Malhotra *et al*, 2015)^69^ or *Atg9a* ^*fl*/*fl*^ mice (Yamaguchi *et al*, 2018). VeCadh‐Cre+^ERT2^; *Atg9a* ^fl/fl^, or *Atg12* ^fl/fl^ mice were referred to as Atg9a^ECKO^ or Atg12^ECKO^. *Sting* ^fl/fl^ (Jin *et al*, 2011) mice were a gift from Dr. Hamida Hamad and Dr. Jonathan Maelfait and were crossed with Atg5^BECKO^ mice to obtain conditional Atg5/STING double knockout (Pdgfb‐Cre^ERT2^; Atg5^fl/fl^STING^fl/fl^) Atg5/Sting^BECDKO^ mice. Both female and male mice from 7 to 14 weeks of age were used for experiments. Inducible deletion was obtained by intraperitoneal injection with tamoxifen (Sigma#T5648‐5G; 20 mg/ml) dissolved in cornoil (Thermo#405435000), once a day for 5 days. For tumor cells, subcutaneous injection of 250.000 tumor cells (B16‐F10, HCMEL12‐mCherry, YUMMER1.7 at 50–60% confluency) suspended in 100 μl of PBS was performed under isoflurane.

### CD8 ^+^ T‐cell depletion

CD8^+^ T‐cells were depleted by intraperitoneal administration of 100 μg of αCD8 antibody (CloneYTS‐169) 3 days before B16‐F10 injections, the day of B16‐F10 injection, and on day 5, 10, 14, and 21 after B16‐F10 injection.

### Anti‐PD1 treatment

αPD1 antibodies (100 μg; clone–RMP1‐14) were administered intrapertonially 3, 10, and 17 days after B16‐F10 injections. αbetaGAL rat IgG2a isotype control antibody (Clone–GL117) was used as control. Both αCD8 and αPD1 antibodies were a kind gift from Dr. Louis Boon (Polpharma Biologics).

### Dextran straining

Prior to sacrifice dextran (Fluorescein, 70,000 MW, anionic CAT.#D1823) (10 mg/ml) was injected through the tail vein (I.V.) and allowed to circulate for 2 h. Mice were perfused with 2% PFA and PBS, and tumors were excised for analysis.

### FACs

#### muTECs

muTECs, subcutaneous tumors were excised, digested, and sorted using published method (Verhoeven *et al*, 2023). For surface and intracellular staining of muTECs, magnetic enrichment of CD31^+^ cells was used. Cell surface immunostaining used the following antibodies: CD31 (1:400, BV421; BD#562939), CD45 (1:200, APC‐Cy7; BD#561037), VCAM1 (1:200, FITC; Biolegend#105705), ICAM1 (1:200, PE; BD #568539), MHCI (1:100 dilution, APC; Biolegend #107613), and MHCII (1:100 dilution, FITC; Biolegend#107605). eBioscience™ Fixable Viability Dye eFluor™ 780 (1:1,000,Thermo#65‐0865‐14) was used to discriminate dead cells. For intracellular staining of IFNβ, cells were fixed and permebilized following the protocol of eBioscience™Foxp3/Transcription Factor Staining Buffer Set. Cells were incubated overnight at 4°C with anti‐IFNβ antibody (1:50; Thermo#PA5‐20390) conjugated to PE in‐house by PE/R‐Phycoerythrin Conjugation Kit‐Lightning‐Link (abcam#ab102918). Data was acquired in BD symphony A5 flowcytometer.

#### Immunophenotyping

CD45^+^ cells obtained during muTEC enrichment were used for immunophenotyping. After selection, 2 × 10^6^ cells were resuspended in DPBS and incubated with eBioscience™ Fixable Viability Dye eFluor™ 780 for dead cell discrimination. Nonspecific binding of antibodies to cell Fc receptors was blocked using 10 μl FcR blocking reagent (BD Purified Rat Anti‐Mouse CD16/CD32 553142) per 10^7^ cells. Cell surface immunostaining used the following antibodies: CD45 (1:400, BUV805; BD#741957), CD3 (1:100, PE‐Cy7; BD#560591), CD4 (1:100, BUV395; BD#565974), CD8a (1:200, BUV496; BD#750024), TIM3 (1:100, BB515; BD#567810), and PD1 (1:100, BV421; BD#562584). After surface protein staining, cells were fixed and permeabilized as above. Then, cells were immunostained with the following antibodies: TCF‐7/TCF‐1 (1:50, AF647; BD#566693), Granzyme B (1:75, PE; Miltenyi Biotec#130‐116‐486), and Ki67 (1:50, PerCP Cy5.5; BioLegend#652425). Data was acquired in BD Symphony A5 or SONY ID7000 spectral flowcytometer.

#### HUVECs

Cells were stimulated with IFNγ for 4 h, trypsinized, and immunostained with VCAM1 antibody (1:100, BV421; BioLegend#305815). eBioscience™ Fixable Viability Dye eFluor™ 780 was used for discriminating dead cells.

#### Coculture of HUVEC with JURKAT‐cells

HUVECs were stimulated with IFNγ (25 IU/ml) and TNFα (5 ng/ml) for 12 h. After 12 h, HUVECs were incubated with αVCAM1 antibody (R&D#BBA5, 2.5 μg/ml) for 30 min at 37°C. Invitrogen™ CellTrace™ Calcein Green (ThermoFisher Scientific#C34852) was added at 10 nM concentration. JURKAT T‐cells were trypsinized and incubated with 10 nM CellTrace™ Calcein Red‐Orange (Thermo#C34851) for 30 min. Both HUVECs and JURKATs were co‐incubated at 1:1 ratio for 30 min at 37°C. Co‐incubated cells were transferred on ice and Amnis Imagestream was used to acquire data. Ideas Application version 6.0 was used to quantify the number of doulets.

All multicolor FACs experiments included single color compensation/unmixing controls. Single color compensation controls or unmixing controls were prepared and run simultaneously with each experiment. For single color controls, UltraComp eBeads™ Compensation Beads (Invitrogen#01‐2222‐42) were stained with fluorescently tagged antibodies individually using the same protocols as cells. Gates were defined based on FMO controls. Data analysis was perform in FlowJo V10.8.1 or FCS express 7.14 research edition.

### Immunofluorescent imaging

#### Tissues

For frozen sections, excised tumors were fixed in 2% PFA at 4°C overnight, followed by 30% sucrose at 4°C overnight before embedding in OCT (Leica). 10 μm thick tissue sections were stained with anti‐CD3 (1:100 clone17A2; BioLegend#100236), anti‐CD31 (1:200 Abcam#28364), anti‐CD31 (1:200 Millipore#MAB1398Z), anti‐NG2 (1:200 Merck#AB5320), anti‐aSMA (1:200 abcam#ab5694), anti‐P62 (1:100 Sigma#P0067), anti‐VCAM1 (1:200 Invitrogen#14‐1061‐82) anti‐ICAM1 (1:200 BD#550287), anti‐STING (1:100 Proteintech#19851‐1‐AP), anti‐NF‐KB2 (1:100 Novus#87760), anti‐NIK (1:100 CST#4994), and anti‐IFN‐B (1:100 Thermo#PA5‐20390). When primary antibodies were unlabeled, fluorophore‐conjugated secondary antibodies were used (1:500 Invitrogen #A‐11008, #A‐11006, #A78963, #A‐11012, #A32733, #A78967). Images were taken with an Observer Z1 microscope (Zeiss) linked to an AxioCam MRM camera (Zeiss) with 40× or 63× magnification, Zeiss Axioscan (20× magnification, or Zeiss Airyscan for super resolution (63×). Image analysis and quantitation were performed using ImageJ software version2.0.0.

#### Cells

Cells were grown on 1.5 mm coverslips, fixed with 4% PFA for 10 min, followed by permeabilization/blocking in 0.1% saponin/5% normal goat serum for 1 h. Cells were incubated with primary antibodies (1:100 anti‐dsDNA [abcam#ab27156], 1:100 anti‐TOM20 [abcam#ab186735], 1:100 anti‐STING [Proteintech#19851‐1‐AP], 1:100 anti‐LMAN1 [Invitrogen#MA5‐25345]) overnight at 4°C followed by washing before addition of secondary antibodies (1:500 Invitrogen#A21245, #A21236, #A11034, #A11029) for 1 h at RT. Coverslips were mounted with Prolong®Gold (Thermo#P36934). Super‐resolution images were acquired at 63x on an inverted Zeiss LSM880 with Airyscan microscope (Zeiss). Refractive index‐matched immersion oil (Zeiss) was used for all experiments. For z‐stacks collection, software‐recommended optimal slice size was used. For post processing and image analysis, ImageJ (software version2.9.0/1.53t) and Imaris (software version9.1) were used. The authors gratefully acknowledge the VIB Bio Imaging Core for their support and assistance in this work.

### RNA isolation

RNA from HUVECs was extracted using Qiagen RNeasy mini kit (Qiagen #74136). For muTECs, cells were sorted directly into 1 ml Trizol at 4°C. Each sample was immediately frozen on dry ice and kept at −80°C. Recommeded Trizol method was used to extract pure RNA in 12 μl nuclease‐free water. Glycogen (Roche#10901393001) was added with iso‐propanol to facilitate RNA precipitation and visualization of the pellet. RNA was quantified by Agilent RNA 6000 Pico Kit (Agilent#5067‐1513). RNA from blood was extracted using PureLink™ Total RNA Blood Kit (#K156001).

### Quantitative RT‐PCR

Specific primers were designed in Primer3 or adopted from published literature: ATG5 (mouse) FW: AAGTCTGTCCTTCCGCAGTC, RV: TGAAGAAAGTTATCTGGGTAGCTCA; 18 s RNA (mouse): FW: AAACGGCTACCACATCCAAG, RV: CCTCCAATGGATCCTCGTTA; VCAM1 (human) FW: TGTAGTGTCATGGGCTGTGAA, RV: CGCTCAGAGGGCTGTCTATC; SELECTIN E (human): Fw: ACCAGCCCAGGTTGAATG RV: GGTTGGACAAGGCTGTGC; ICAM1 (human) FW:CCTTCCTCACCGTGTACTGG RV: AGCGTAGGGTAAGGTTCTTGC; CXCL10 (human) FW: GAAAGCAGTTAGCAAGGAAAGGT RV: GACATATACTCCATGTAGGGAAGTGA; CX3CL1 (human) FW: AAGCCACCAACATACTCCCA, RV: TGGTAAGGACTGTGAGGCTG.

PPIB (human) FW: CCAACGCAGGCAAAGACACCAA, RV: GCTCTCCACCTTCCGCACCA.

For HUVECs, blood and tumor, RNA was converted to cDNA by QuantiTect Reverse Transcription Kit (Qiagen#205311). For muTECs, RNA was converted to cDNA by SuperScript™ III First‐Strand Synthesis System (Invitrogen#18080051). ORA™ SEE qPCR Green ROX L Mix (highQu# QPD0505) was used to quantify gene expression in Applied Biosystems QuantStudio 5 Real‐Time PCR System. Fold change was calculated by $2^{-\Delta \Delta C_{t}}$ method. *C*_t_ values for each gene was normalized for loading as follows. HUVEC—PPIB, blood—HPRT, muTECs—average of 18 s rRNA + GAPDH. For verification of loss of Exon 3 in Atg5^BECKO^ mice, forward primer was designed to bind in Exon 3 region and reverse primer was designed to bind exon 4 reigon of *ATG5* gene.

### Western blotting

Immunoblotting on whole cell lysates was performed as previously described (Meçe *et al*, 2022). Membranes were incubated overnight with antibodies against VCAM1 (1:1,000; Abcam#ab134047), TBK1 (1:1,000; CST#3504S), pTBK1 (1:1,000; CST#5483S), STING (1:1,000; CST#13647S), cGAS (1:1,000; CST#15102), P100/p52 (1:1,000; CST#4882), p‐IκBα (Ser32/36) (1:500; CST#9246), IκBα (CST#9242 RELB (1:1,000; CST#4922), ATG5 (1:1,000; CST#12994S), GAPDH (1:3,000; CST#5174S), VDAC (1:1,000; CST#4661S), Caspase3 (1:1,000; CST#9662S), cleaved‐caspase3 (1:1,000; CST#9664S), LC3B (1:1,000; CST#3868S), and P62 (1:1,000; CST#39749S). Membranes were then incubated with respective secondaries (1:2,000; CST#7074S, #7076S, Thermo#SA5‐10036, #35568) for 1 h and revealed using Amersham ECL imager (for HRP) or Amersham Typhoon Biomolecular Imager (for DyLight). BIO‐RAD Clarity and Clarity Max ECL Western Blotting Substrates were used with HRP conjugated antibodies. For semi‐native western blotting for STING nondenaturing conditions were used to prepare cell lysates. Quantification was done using ImageJ software version2.0.0.

### Nanostring

Counter PanCancer Mouse Immune Profiling Panel (NanoString #XT‐CSO‐MIP1‐12) was used to screen gene expression in muTEC. Data was analyzed with nSolver software version 4.0 (advanced analysis). 10 ng RNA from muTECs were loaded for analysis.

#### GSEA analysis

Gene set enrichment analysis (GSEA) was carried out using GSEA software with MH: hallmark gene sets in the MSigDB database on the list of 744 genes analyzed as input through nanostring technology. The default gene list was used as background. For visualization, SRplot online tool was used to build Bubble plot, *P* values are represented by colors, and gene counts are represented by bubble size.

### Single‐cell RNA sequencing analysis—metadata analysis

#### Patient population

We collected 79 fresh tissue samples from 53 patients across 4 tumor types: breast cancer (BC; *n* = 31, 39%), colorectal cancer (CRC; *n* = 21, 27%), high‐grade serous ovarian carcinoma (HGSOC; *n* = 12, 15%), and non‐small cell lung cancer (NSCLC; *n* = 15, 19%). All samples were treatment‐naïve tumors that have already been published (Qian *et al*, 2020; Bassez *et al*, 2021). The local ethics committee at the University Hospital Leuven approved the single‐cell study for each cancer type, and all patients provided written informed consent.

### Single‐cell RNA sequencing (scRNA‐SEQ) and expression analysis

Single‐cell suspensions underwent 3′ or 5’ scRNA‐seq using the ChromiumTM Single Cell V(D)J Solution from 10x Genomics as previously described (Bassez *et al*, 2021). Most tissues (CRC, HGSOC and NSCLC samples; 66%) were subjected to 3’ scRNAseq, although in some cases (BC samples; 34%) 5′ were performed instead. Gene expression libraries were generated according to the manufacturer's instructions with the aim to obtain 5,000 cells per sample. All libraries were sequenced on Illumina NextSeq, HiSeq4000, and/or NovaSeq6000 and mapped to the GRCh38 human reference genome using CellRanger (10× Genomics). The latter was also used to generate raw gene expression matrices19, then analyzed using the Seurat v4 R package20. Cells expressing < 200 or > 6,000 genes, < 400 unique molecular identifiers (UMIs), and > 25% mitochondrial counts were removed.

### Clustering ECs subtypes

We first analyzed each cancer type independently and focused on the clusters analysis as previously described (Qian *et al*, 2020; Bassez *et al*, 2021). All cells assigned as ECs per each cancer type were merged, and the same clustering strategy was applied at the subcluster level. In addition, for the identification of cellular subgroups, we integrated the data generated from different technologies (3′ or 5’ scRNA‐seq) with the batch effect correction algorithm harmony (Korsunsky *et al*, 2019). In addition, at the subcluster level, we also regressed out for individual tumor types, interferon response, and stress signature, as previously described (Qian *et al*, 2020). Clusters representing distinct cell types were identified based on the expression of marker genes. Statistical analyses were performed using R (version 4.0.3, R Foundation for Statistical Computing, R Core Team, Vienna, Austria). Spearman's correlation analysis was applied to quantify correlations between levels of signatures gene expression. Statistical analyses were performed with the Mann–Whitney test, using a two‐sided alternative hypothesis at the 5% significance level.

### Melanoma single‐cell EC data

The raw count matrix of the entire Grand Challenge melanoma dataset was obtained and anayzed as previously described (Pozniak *et al*, 2022). TME cells including the CD8^+^ T‐cells were identified as in Landeloos *et al* (unpublished) and the correlations of CD8^+^ T‐cells with muTEC‐DE score were performed using Spearman test. Endothelial cells were identified by calculating the AUCell gene enrichment score of EC signatures and selecting cells with an AUCell score > 0.1 for downstream analysis. High‐variable genes were selected using the FindVariableFeatures function and auto‐scaled with the ScaleData function. Principal component analysis (PCA) was performed using the default RunPCA function in the Seurat package (Stuart *et al*, 2019), and batch effect correction was applied to each sample using the harmony algorithm (Korsunsky *et al*, 2019) based on PCA space. The data were visualized using uniform manifold approximation and projection (UMAP) with the RunUMAP step (dims = 10), and unsupervised clustering was performed using the FindNeighbors followed by FindClusters function (dims = 10, resolution = 0.4) in the Seurat package. EC subtypes were identified primarily based on marker genes reported in the literature (Hua *et al*, 2022). The enrichment of given gene sets for each cell was evaluated using the AUCell package (Aibar *et al*, 2017) for GSEA. Modified violin + box plots were generated using customized R code, and these functions were integrated into the R package “SeuratExtend,” which is available on Github (https://github.com/huayc09/SeuratExtend). Statistical analyses were performed using ggpubr package in R.

### Clinical data for MILAN analysis

A selection of patients included in a previously published dataset (Antoranz *et al*, 2022) was made for analysis with MILAN technology. The protocol was approved for use of human materials by UZ Leuven/KU Leuven (HBM) (approval number S66737). 12 pretreatment, formalin‐fixed, and paraffin‐embedded (FFPE) melanoma metastasis samples from 12 patients were collected from the histopathological archives of the University Hospital of Leuven (Clinical data reported in Table EV2). All patients were treated with anti‐PD‐1 monotherapy (nivolumab or pembrolizumab), and after biopsy was taken. Only biopsies taken < 365 days before the start of anti‐PD‐1 monotherapy were included. Furthermore, only patients with measurable disease were selected, hence enabling tumor response assessment according to RECIST 1.1 (Eisenhauer *et al*, 2009). Patients were classified according to the best objective response to immunotherapy during their time of follow‐up, as defined by RECIST 1.1 (Eisenhauer *et al*, 2009). Complete response and partial response were classified as R for responder, progressive disease, or stable disease as NR for nonresponder. According to these criteria, 6 patients could be classified as R (6 samples) and 6 patients as NR (6 samples). Only metastatic samples were eligible for inclusion. An expert dermatopathologist specialized in melanoma research (FMB) selected the most representative areas of the tumors for tissue microarray (TMA) construction. For each metastasis, one to five representative cores/regions of interest were sampled having at least a size of 1 mm in diameter. The number of samples taken was determined by the specimen and the morphologic heterogeneity of both the melanoma and the inflammatory infiltrate. Therefore, a smaller number of cores were taken from small and homogeneous samples, whereas a larger number was taken from large but heterogeneous specimens. The studies were conducted in accordance with recognized ethical guidelines set out in the WMA Declaration of Helsinki and the Department of Health and Human Services Belmont Report. This project was approved by the Ethical Commission of the University Hospital of Leuven and approved by the review board.

### Multiple iterative labeling by antibody neodeposition (MILAN)

#### Tissue staining

Multiplex immunofluorescent staining was conducted using the MILAN protocol as described previously (Bolognesi *et al*, 2017), and immunofluorescence images were acquired using the Axio scan.Z1 slidescanner (Zeiss, Germany) at 10× objective with a resolution of 0.65 micron/pixel at 16‐bit color depth. Hematoxylin and eosin (H&E) slides were also digitized using the same slidescanner in brightfield modus with a 10X objective at 8‐bit color depth. All samples were stained together, and the quality of the stains was evaluated by an experienced pathologist (FMB). Poor‐quality areas showing artifacts such as tissue folds or antibody aggregation were excluded from downstream analysis.

### Image analysis

Image analysis was conducted using a previously described (Antoranz *et al*, 2022) customized pipeline. Briefly, images were corrected for field of view artifact using a method described in the literature (Kask *et al*, 2016). Then, the overlapping regions of adjacent tiles were stitched together by minimizing the Frobenius distance. Next, images from consecutive rounds were aligned (registered) following an algorithm previously described (Reddy & Chatterji, 1996). To register the images from consecutive rounds, the first round was set as a fixed image while all the following rounds were used as moving images. The DAPI channel was used to calculate transformation matrices, which were then applied to the other channels. The quality of the overlapping regions was visually evaluated and poor registered areas were removed from downstream analyses. Then, tissue autofluorescence was removed by subtracting a baseline image with only secondary antibody. Finally, cell segmentation was applied to the DAPI channel using STARDIST (Schmidt *et al*, 2018; Weigert *et al*, 2020), which delineates a contour for each cell present in the tissue. For each of these cells, the following features were extracted: topological features (X/Y coordinates), morphological features (nuclear size), and molecular features (mean fluorescence Intensity (MFI) of each measured marker).

### Single‐cell analysis

MFI values were normalized using *Z*‐scores within each sample, as recommended in Caicedo *et al* (2017). To avoid a strong influence from outliers in downstream analyses, *Z*‐scores were trimmed within the [−5, 5] range. Three different clustering methods were used to map single cells to known cell phenotypes: PhenoGraph (Levine *et al*, 2015), FlowSom (Quintelier *et al*, 2021), and KMeans, which were implemented in the Rphenograph, FlowSOM, and stats R packages, respectively. While FlowSom and KMeans required the number of clusters as input, PhenoGraph could be executed by exclusively defining the number of nearest neighbors to calculate the Jaccard coefficient 20 (standard value). The number of clusters identified by PhenoGraph was then used as an argument for FlowSom and KMeans. Clustering was performed exclusively on a subset of the identified cells (2,500), which were selected by stratified proportional random sampling and using the following markers: AQP1, CD31, CD4, CD8, MelanA, and SOX10. For each clustering method, clusters were mapped to known cell phenotypes based on manual annotation by domain experts. For every cell, if two or more clustering methods agreed on the assigned phenotype, the cell was annotated accordingly. However, if all three clustering methods disagreed on the assigned phenotype, the cell was annotated as “not otherwise specified” (NOS).

Following consensus clustering, four differenT‐cell types were identified (^+^NOS): Melanoma (SOX10^+^ | MelanA^+^), Blood Vessels (AQP1^+^ | CD31^+^), T‐helpers (CD4^+^), and Cytotoxic T‐cells (CD8^+^). To extrapolate the cell labels to the remaining cells in the dataset, a uMap was built by sampling 500 cells for each identified cell type in the consensus clustering, and the entire dataset was projected into the uMap using the base predict R function. For each cell, the label of the closest 100 neighbors was evaluated in the uMap space, and the most frequent T‐cell type was assigned as the label. Digital reconstructions of the tissue samples were obtained by coloring the segmentation mask by the assigned cell label. An experienced dermatopathologist (FMB) used these reconstructions to annotate different areas of interest: “tumoral bulk” areas, tumor–stroma interface, and non‐tumoral areas. Relative cytometry enrichment was performed for each individual area using Wilcoxon rank‐sum tests. Given the exploratory nature of the analysis, *P*‐values were not adjusted for multiple comparisons. Statistical analysis and data presentation were performed using R Studio (version RStudio 2022.07.2). Relative STING^+^ and VCAM1^+^ blood vessels were identified by applying a cutoff on the respective normalized data. The same analysis was performed for Cytotoxic T‐cells using PD‐1 and GrzB.

### Statistical analysis

Statistical difference between two groups was determined by standard two‐sided Student's *t*‐test, Mann–Whitney *U* test, or to compare paired samples, ratio paired *t*‐test was used. For three or more groups, one‐way ANOVA with Tukey's post hoc test or Holm‐Sidak correction for multiple comparisons were used. Statistical outliers were excluded based on the Grubb's test in Prism software. Additionally, mice where tumors never grew after injection were excluded from analyses. All mouse trials were conducted with at least 4 mice per group. Mice were randomized between treatment allocations. No blinding was performed in our experiments. All TEC/tissue analysis was conducted with at least three mice per group or HUVECs with three independent donors. GP *P* value style was used. */$/ represents a *P*‐value < 0.05, **/$$ < 0.01, ***P < 0.001, and ****P < 0.0001, where a *P*‐value < 0.05 was used to determine statistical difference. All statistical analysis was performed in Prism 9.4.1.

## Author contributions

**Patrizia Agostinis:** Conceptualization; resources; supervision; funding acquisition; writing – original draft; project administration; writing – review and editing. **Jelle Verhoeven:** Conceptualization; formal analysis; investigation; visualization; methodology; writing – original draft. **Kathryn A Jacobs:** Formal analysis; validation; investigation; visualization; writing – review and editing. **Francesca Rizzollo:** Formal analysis; investigation; writing – review and editing. **Francesca Lodi:** Data curation; formal analysis; investigation. **Yichao Hua:** Data curation; formal analysis; investigation. **Joanna Poźniak:** Data curation; formal analysis; investigation. **Adhithya Narayanan Srinivasan:** Formal analysis; investigation. **Diede Houbaert:** Formal analysis; investigation. **Gautam Shankar:** Formal analysis. **Sanket More:** Formal analysis. **Marco B Schaaf:** Conceptualization; formal analysis; investigation. **Nikolina Dubroja Lakic:** Methodology. **Maarten Ganne:** Investigation. **Jochen Lamote:** Investigation. **Johan Van Weyenbergh:** Methodology. **Louis Boon:** Resources. **Oliver Bechter:** Resources. **Francesca Bosisio:** Resources. **Yasuo Uchiyama:** Resources. **Mathieu JM Bertrand:** Resources. **Jean Christophe Marine:** Resources. **Diether Lambrechts:** Resources. **Gabriele Bergers:** Writing – review and editing. **Madhur Agrawal:** Conceptualization; formal analysis; investigation; visualization; writing – original draft.

## Disclosure and competing interests statement

The authors declare that they have no conflict of interest.

## Supporting information



Appendix S1

Expanded View Figures PDF

Table EV1

Table EV2

PDF+

Source Data for Figure 1

Source Data for Figure 2

Source Data for Figure 3

Source Data for Figure 4

Source Data for Figure 5

Source Data for Figure 6

Source Data for Figure 7

## Data Availability

Nanostring data can be found in the Gene Expression Omnibus (GEO) access number GSE244158. All raw images from main figures have been uploaded to BioImage Archive under https://www.ebi.ac.uk/biostudies/studies/S‐BSST1202.
