# Supplementary material for: Tumor endothelial cell autophagy is a key vascular‐immune checkpoint in melanoma
Source: EMBO Mol Med. 2023 Nov 27;15(12):e18028. doi: 10.15252/emmm.202318028 (PMC10701618; doi:10.15252/emmm.202318028)
Supplement: Supplementary file 1 — Appendix S1 [file EMMM-15-e18028-s009.pdf]

Appendix File

Appendix Figure S1..Page 2

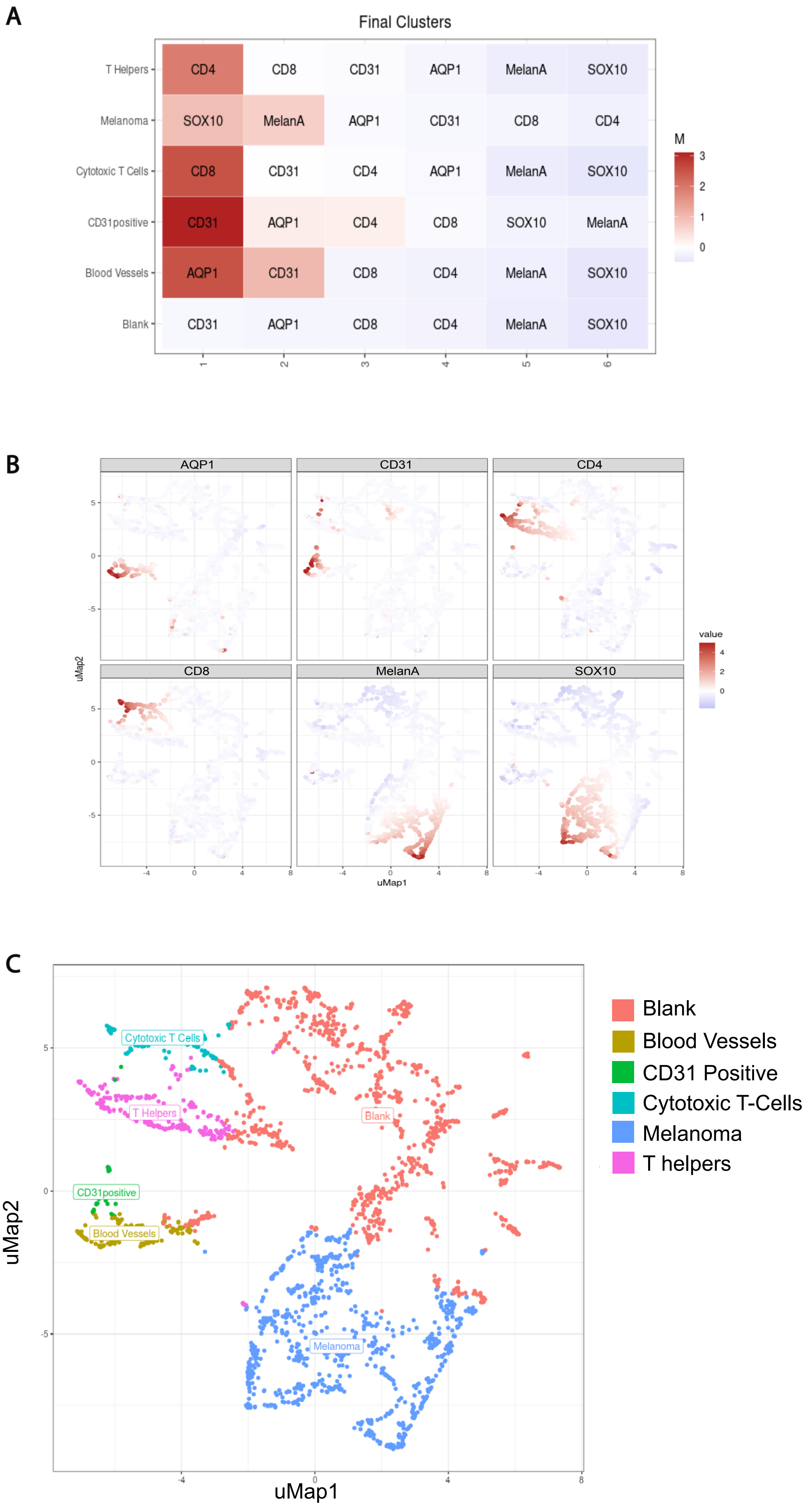
A
Final Clusters
T Helpers
Melanoma
Cytotoxic T Cells
CD31positive
Blood Vessels
Blank
CD4 CD8 CD31 AQP1 MelanA SOX10
SOX10 MelanA AQP1 CD31 CD8 CD4
CD8 CD31 CD4 AQP1 MelanA SOX10
CD31 AQP1 CD4 CD8 SOX10 MelanA
AQP1 CD31 CD8 CD4 MelanA SOX10
CD31 AQP1 CD8 CD4 MelanA SOX10
1 2 3 4 5 6
M
3 2 1 0
B
AQP1
CD31
CD4
CD8
MelanA
SOX10
uMap2
uMap1
value
4 2 0
C
Cytotoxic T Cells
T Helpers
Blank
CD31positive
Blood Vessels
Melanoma
uMap2
uMap1
Blank
Blood Vessels
CD31 Positive
Cytotoxic T-Cells
Melanoma
T helpers

Appendix Figure S1. Protein markers used to annotate cells in the MILAN analysis

A, Table illustrating the markers used for annotating ECs and $CD8^+$ T-cells in tissue sections from melanoma patients undergoing anti-PD1 monotherapy using MiLAN.

B, AQP1, CD31, CD4, CD8, MelanA, and SOX10 expression projected on the UMAP generated built by sampling 500 cells for each identified cell type in the consensus clustering. Entire dataset was projected into the uMap using the base predict R function.

C, UMAP showing identified cell types based on the markers shown in extended figure panel **A**.
