# Supplementary material for: Tumor endothelial cell autophagy is a key vascular‐immune checkpoint in melanoma
Source: EMBO Mol Med. 2023 Nov 27;15(12):e18028. doi: 10.15252/emmm.202318028 (PMC10701618; doi:10.15252/emmm.202318028)
Supplement: Supplementary file 2 — Expanded View Figures PDF [file EMMM-15-e18028-s008.pdf]

Expanded View Figures

Figure EV1. Validation of genetic loss of *Atg5* in tumor endothelial cells.

A Gating strategy for quantifying GFP signal in $CD31^+$ tumor endothelial cells in subcutaneous B16-F10 tumors from WT and $Atg5^{BECKO}$ mice.

B Representative immunofluorescence images showing tdtomato and GFP in $CD31^+$ tumor endothelial cells in subcutaneous B16-F10 tumors from WT and $Atg5^{BECKO}$ mice with subcutaneous B16-F10 tumors.

C Gene expression of *Atg5* normalized to 18 s rRNA + GAPDH in sorted $CD31^+$ tumor endothelial cells in subcutaneous B16-F10 tumors from WT and $Atg5^{BECKO}$ mice. Forward primer was designed to bind in Exon 3 region and reverse primer was designed to bind in Exon 4 region of *Atg5* gene.

D, E Representative immunofluorescence images and quantification for p62 (MFI), in $CD31^+$ tumor endothelial cells from subcutaneous B16-F10 tumors from WT and $Atg5^{BECKO}$, $ATG9a^{ECKO}$, And $ATG12^{ECKO}$ mice.

F Representative immunofluorescence images and quantification for NG2 (% coverage) and αSMA (% coverage) in $CD31^+$ tumor endothelial cells from subcutaneous B16-F10 tumors from WT and $Atg5^{BECKO}$ mice.

G Representative immunofluorescence images and quantification for dextran outside of $CD31^+$ vessels from subcutaneous B16-F10 tumors from WT and $Atg12^{ECKO}$ mice. Scale bars represent 10 μm.

Data Information: For immunofluorescence staining, at least 3 WT and 3 $Atg5^{BECKO}$ mice were used for the quantification. All data represent mean ± s.e.m. Statistical differences were determined using two-sided Student's *t*-test.

▶

© 2023 The Authors

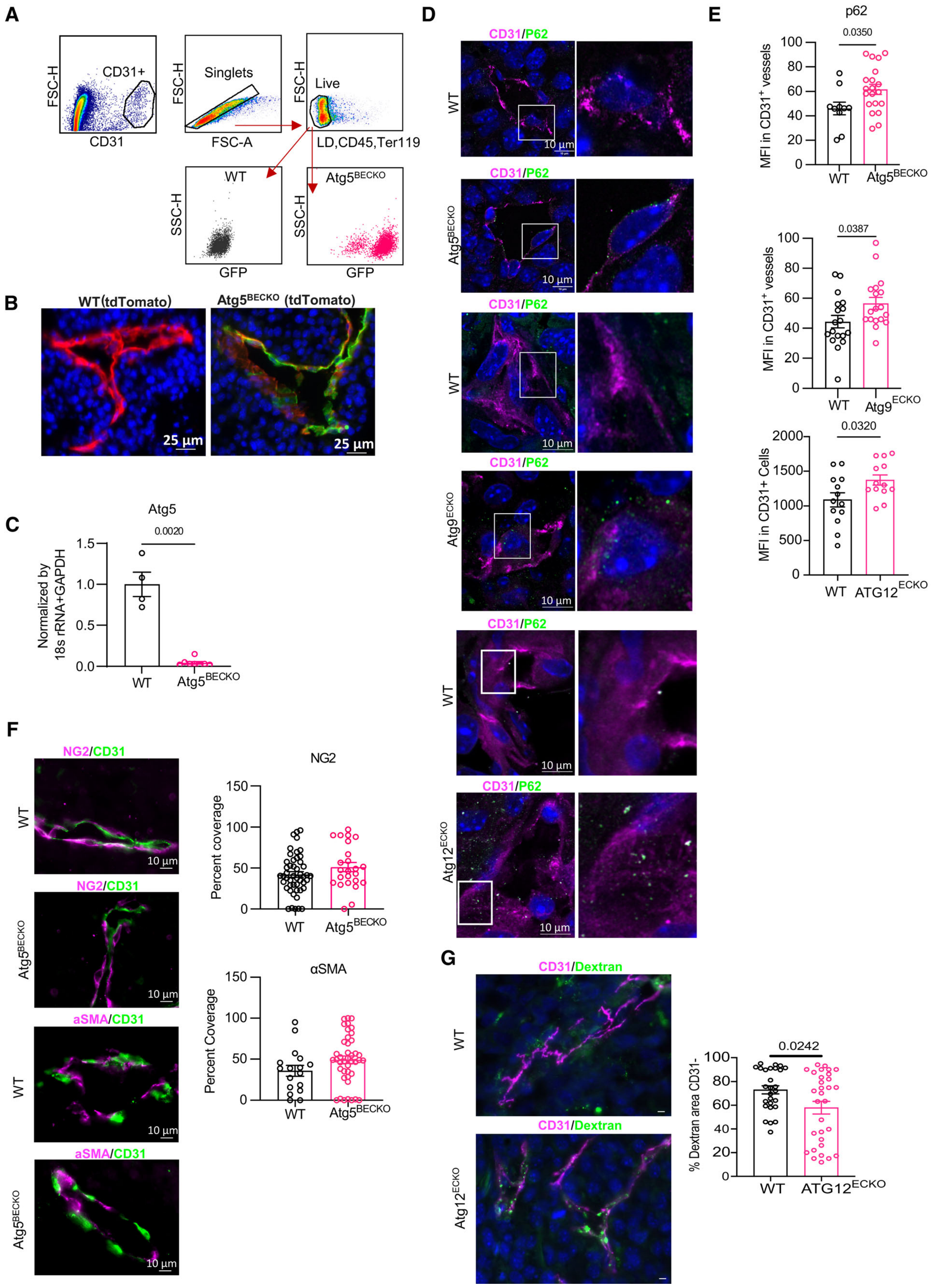
A
FSC-H
CD31+
CD31
FSC-H
Singlets
FSC-A
SSC-H
Live
LD,CD45,Ter119
WT
SSC-H
GFP
Atg5BECKO
SSC-H
GFP
B
WT(tdTomato)
Atg5BECKO (tdTomato)
25 µm
25 µm
C
Atg5
0.0020
Normalized by 18s rRNA+GAPDH
1.5
1.0
0.5
0.0
WT
Atg5BECKO
D
CD31/P62
WT
10 µm
CD31/P62
Atg5BECKO
10 µm
CD31/P62
WT
10 µm
CD31/P62
Atg9ECKO
10 µm
CD31/P62
WT
10 µm
CD31/P62
Atg12ECKO
10 µm
E
p62
0.0350
MFI in CD31+ vessels
100
80
60
40
20
0
WT
Atg5BECKO
0.0387
MFI in CD31+ vessels
100
80
60
40
20
0
WT
Atg9ECKO
0.0320
MFI in CD31+ Cells
2000
1500
1000
500
0
WT
ATG12ECKO
F
NG2/CD31
WT
10 µm
NG2/CD31
Atg5BECKO
10 µm
aSMA/CD31
WT
10 µm
aSMA/CD31
Atg5BECKO
10 µm
NG2
Percent coverage
150
100
50
0
WT
Atg5BECKO
αSMA
Percent Coverage
150
100
50
0
WT
Atg5BECKO
G
CD31/Dextran
WT
CD31/Dextran
Atg12ECKO
0.0242
% Dextran area CD31-
100
80
60
40
20
0
WT
ATG12ECKO

Figure EV1.

Figure EV2. Immunophenotyping of WT and Atg5^{BECKO} mice with subcutaneous B16-F10 melanoma.

A Gating strategy for analyzing immune cell subsets in subcutaneous B16-F10 melanoma from WT and Atg5^{BECKO} mice.

B Gene expression of *CD8*, *CD3g*, *CD45*, and *Nkp46* in blood collected from WT and Atg5^{BECKO} mice with subcutaneous B16-F10 tumors and injected with αCD8 antibody. Each point represents 1 mouse; with at least $n = 4$ mice per group.

Data Information: All data represent mean ± s.e.m. Statistical differences were determined using one-way Anova with Tukey corrections for multiple comparisons (B).

© 2023 The Authors

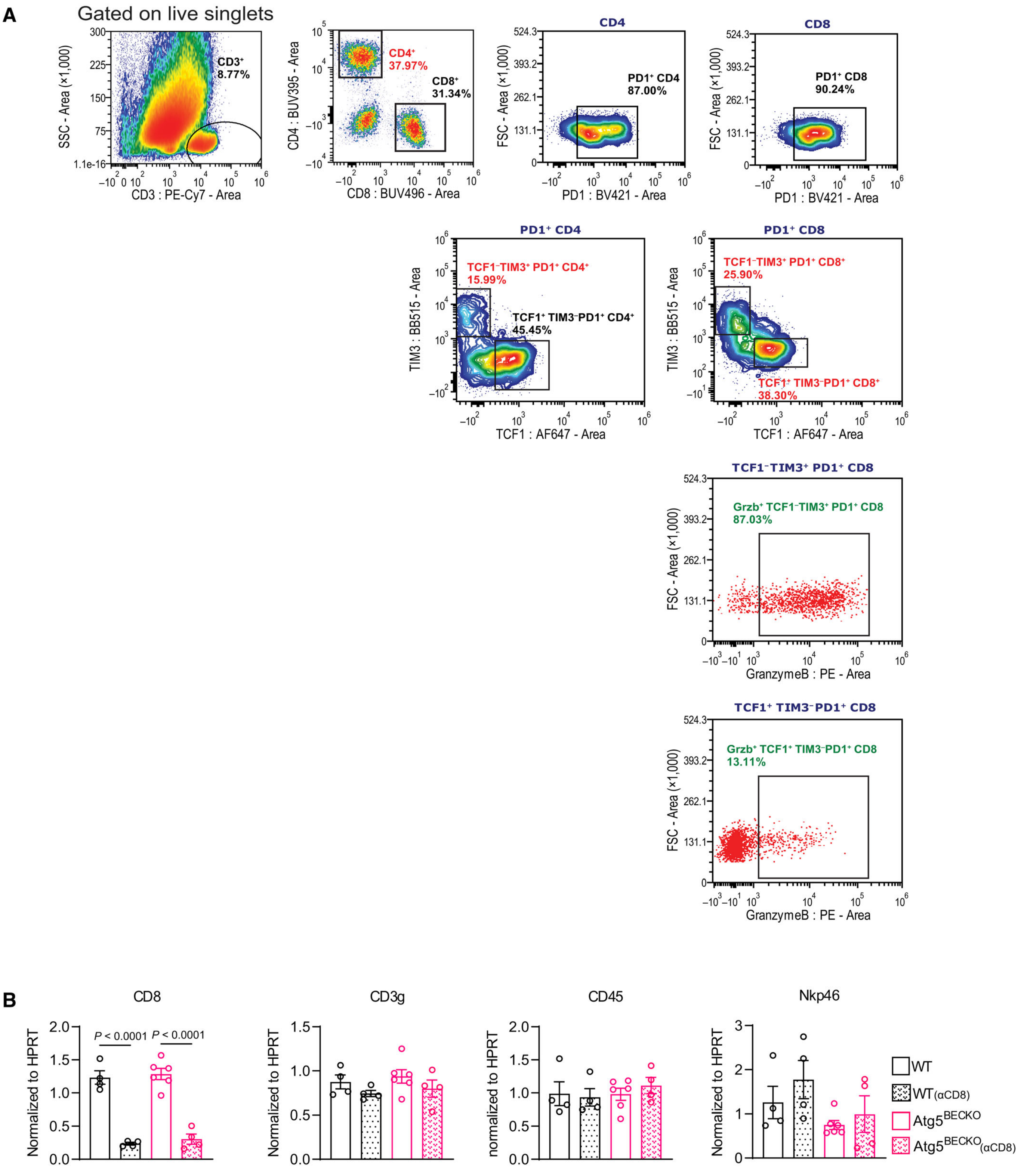
A
Gated on live singlets
CD3+ 8.77%
SSC - Area (×1,000)
CD3 : PE-Cy7 - Area
CD4+ 37.97%
CD8+ 31.34%
CD4 : BUV395 - Area
CD8 : BUV496 - Area
CD4
PD1+ CD4 87.00%
FSC - Area (×1,000)
PD1 : BV421 - Area
CD8
PD1+ CD8 90.24%
PD1+ CD4
TCF1−TIM3+ PD1+ CD4+ 15.99%
TCF1+ TIM3−PD1+ CD4+ 45.45%
TIM3 : BB515 - Area
TCF1 : AF647 - Area
PD1+ CD8
TCF1−TIM3+ PD1+ CD8+ 25.90%
TCF1+ TIM3−PD1+ CD8+ 38.30%
TCF1−TIM3+ PD1+ CD8
Grzb+ TCF1−TIM3+ PD1+ CD8 87.03%
GranzymeB : PE - Area
TCF1+ TIM3−PD1+ CD8
Grzb+ TCF1+ TIM3−PD1+ CD8 13.11%
B
CD8
CD3g
CD45
Nkp46
Normalized to HPRT
normalized to HPRT
P < 0.0001
P < 0.0001
WT
WT(αCD8)
Atg5BECKO
Atg5BECKO(αCD8)

Figure EV2.

Figure EV3. Phenotype of TEC from WT and Atg5^{BECKO} mice and human TEC subclusters from single-cell RNA-seq atlases.

A Gating strategy for sorting of tumor endothelial cells from subcutaneous B16-F10 melanoma from WT and Atg5^{BECKO} mice.

B Flow cytometry analysis for the surface expression of MHCI and MHCII in CD31^{+} tumor endothelial cells derived in subcutaneous B16-F10 tumor from WT and Atg5^{BECKO} mice. Each point represents an individual mouse, with $n = 6$ mice per group.

C Representative images and quantification (MFI) of immunofluorescence staining for VCAM1, ICAM1, and STING in CD31^{+} tumor endothelial cells from tumor sections of subcutaneous YUMMER 1.7 tumors from WT and Atg12^{ECKO} mice. Scale bars represent 10 μm for VCAM1 and STING and 20 μm for IcAM1.

D–G Preliminary analysis of publicly available single-cell RNA-seq atlases from primary tumors of treatment-naïve patients. Genetic markers used to cluster EC subsets and their corresponding expression shown in a heatmap. BC, breast cancer; CRC, colorectal cancer; HGSOC, high-grade serous ovarian carcinoma; NSCLC, non-small cell lung cancer; PCV, post-capillary venules.

D Heatmap showing expression of representative marker genes across 9 ECs subtypes.

E UMAP map of Ecs ($n = 7.573$) color coded for the indicated cell type.

F Pie chart showing the pan-cancer relative abundance of the 9 Ecs subtypes.

G Relative abundance of the 9 Ecs subtypes across tumor types included in the study. Boxplots embedded were made by ggplot2 package in R. The lower and upper hinges correspond to the first and third quartiles. The upper whisker extends from the hinge to the largest value no further than 1.5 * IQR from the hinge (where IQR is the interquartile range). The lower whisker extends from the hinge to the smallest value (at most 1.5 * IQR of the hinge). (D–G) Samples from 48 cancer patients were used in the analysis. In particular, 31 from BC_early, 7 from CRC, 2 from HGSOC and 8 from NSCLC_early.

Data Information: For immunofluorescence staining, 2 WT and 3 Atg12^{ECKO} mice were used for the analysis. All data represent mean ± s.e.m. Statistical differences were determined using two-sided Student's *t*-test (B, C) or in (G), exact *P* values by two-sided Mann–Whitney test or two-sided Wilcoxon matched-pairs signed rank test: $^{*}P < 0.05$, $^{**}P < 0.01$, $^{***}P < 0.001$.

© 2023 The Authors

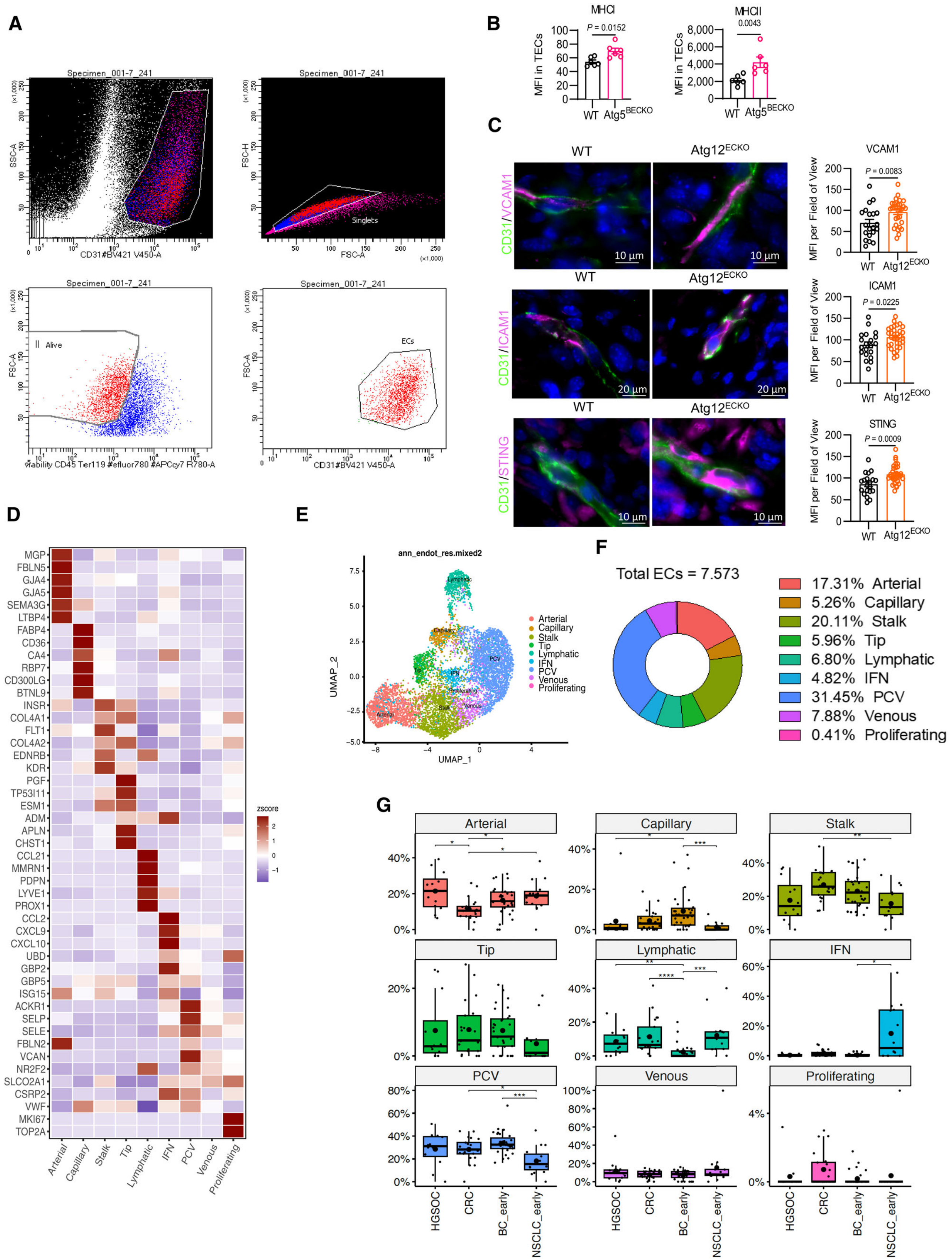
A
B
MHCI
MHCII
MFI in TECs
WT
Atg5BECKO
C
WT
Atg12ECKO
CD31/VCAM1
CD31/ICAM1
CD31/STING
VCAM1
ICAM1
STING
MFI per Field of View
D
E
ann_endot_res.mixed2
UMAP_1
UMAP_2
F
Total ECs = 7.573
17.31% Arterial
5.26% Capillary
20.11% Stalk
5.96% Tip
6.80% Lymphatic
4.82% IFN
31.45% PCV
7.88% Venous
0.41% Proliferating
G
HGSOC
CRC
BC_early
NSCLC_early

Figure EV3.

Figure EV4. Autophagy blockade in HUVECs promotes formation of STING dimers and oligomers and accumulation in ERGIC.

A–C Representative western blot for Atg5 (A), LC3B (B), and p62 (C) and quantification for p62 in HUVECs nucleofected with scrambled (Ctr) or Atg5 specific guide RNA (Atg5KO) conjugated with cas9 protein. The quantification in C comes from 3 biological replicates.

D Western blot of BNIP3L, VDAC, caspase-3, and cleaved caspase 3 in Ctr and Atg5KO HUvECs.

E Gene expression analysis of *VCAM1*, *SELE*, *ICAM1*, *CXCL10*, and *C3CXL1* in HUVECs treated with vehicle (Ctr) or ULK1/2 inhibitor (top) and vehicle (Ctr) or bafilomycin A (BfA) (bottom); $n = 3$ biological replicates.

F Gating strategy for flow cytometric analysis of surface expression of VCAM1 (% of live cells) in Ctr and Atg5KO HUVECs upon stimulation with IFNγ for 4 h.

G Imaging flow cytometry analysis for doublet formation (# doublets/10,000 events) in Ctr and Atg5KO HUVECs (stimulated with IFNγ and TNFα for 12 h) with JURKAT-cells.

H Representative western blot of Native and reducing conditions for STING in HUVECs treated with vehicle or ULK1/2 inhibitor.

I Super-resolution Airyscan immunofluorescence images for STING (green) and LMAN1 (magenta) proteins in Ctr and Atg5KO HUVEC. Scale bars represent 10 μm and at least 30 cells (10 per donor) were imaged per condition.

J Super-resolution Airyscan immunofluorescence images for DAPI in Ctr and Atg5 KO HUVECs. Scale bars represent 10 μm. Representative images from three independent experiments.

Data information: Statistical differences were determined using two-sided Student's *t*-test.

© 2023 The Authors

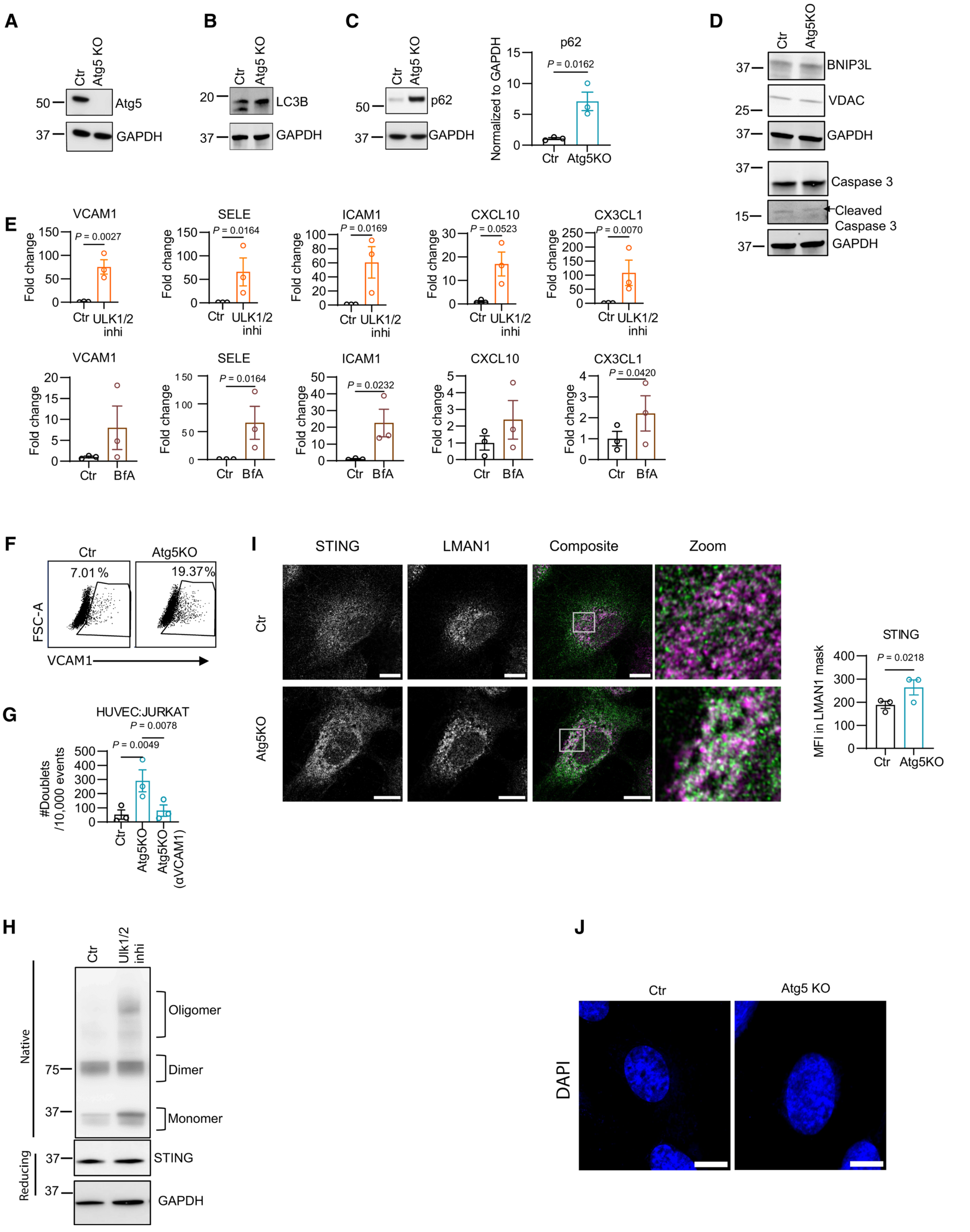
A
Ctr
Atg5 KO
50
Atg5
37
GAPDH
B
Ctr
Atg5 KO
20
LC3B
37
GAPDH
C
Ctr
Atg5 KO
50
p62
37
GAPDH
p62
P = 0.0162
Normalized to GAPDH
Ctr
Atg5KO
D
Ctr
Atg5KO
BNIP3L
VDAC
GAPDH
Caspase 3
Cleaved Caspase 3
E
VCAM1
SELE
ICAM1
CXCL10
CX3CL1
Fold change
P = 0.0027
P = 0.0164
P = 0.0169
P = 0.0523
P = 0.0070
Ctr
ULK1/2 inhi
P = 0.0232
P = 0.0420
BfA
F
Ctr
Atg5KO
7.01 %
19.37 %
FSC-A
VCAM1
G
HUVEC:JURKAT
P = 0.0078
P = 0.0049
#Doublets /10,000 events
Atg5KO (αVCAM1)
H
Ulk1/2 inhi
Native
Reducing
Oligomer
Dimer
Monomer
STING
GAPDH
I
STING
LMAN1
Composite
Zoom
Ctr
Atg5KO
P = 0.0218
MFI in LMAN1 mask
J
Atg5 KO
DAPI

Figure EV4.

Figure EV5. Analysis of genetic deletion of *Sting* and *Atg5* in TECs and interferon subtype of ECs from patients responding to αPD1 therapy.

A, B Gene expression for *ATG5* (A) and *STING* (B) in $CD31^+$ tumor endothelial cells sorted from subcutaneous B16-F10 melanoma tumors from WT, $Atg5^{BECKO}$ and $Atg5/STING^{BECDKO}$ mice. For *ATG5*, forward primer was designed to bind in Exon 3 region and reverse primer was designed to bind in Exon 4 region. For STING, forward primer designed to bind in Exon 2 region and reverse primer was designed to bind in Exon 3 region, at least 3 mice per group were used for the analysis.

C Quantification of immunofluorescence staining for $CD3^+$ T-cells in the tumor sections of subcutaneous B16-F10 tumors from $Atg5^{+/+}$, $Atg5/Sting^{+/+}$, $Atg5^{BECKO}$, and $Atg5/Sting^{BECDKO}$ mice at least 3 mice per group were used for the analysis.

D Representative images and quantification (MFI) of immunofluorescence staining for CD31 and NIK in the tumor sections of subcutaneous B16-F10 tumors from $Atg5^{+/+}$, $Atg5/Sting^{+/+}$, $Atg5^{BECKO}$ and $Atg5/Sting^{BECDKO}$ mice, at least 3 mice per group were used for the analysis.

E Grouped tumor volume showing Mean and s.e.m. of B16-F10 subcutaneous tumor bearing WT and $Atg5^{BECKO}$ mice injected with isotype (ISO) or αPD1 antibody. Data is from 1 representative experiment with at least $N = 3$ mice per group.

F UMAP map of ECs ($n = 7.573$) color-coded for the indicated cell type.

G Gene enrichment score of muTEC-DE geneset (top) and autophagy geneset (bottom) across different subsets of huTECs from treatment naïve stage III/IV melanoma patients receiving anti-PD1 based therapy monotherapy (nivolumab). Each point is 1 EC. ECs come from $n = 10–11$ patients per group. Boxplots embedded in the violin plots were made by ggplot2 package in R. The lower and upper hinges correspond to the first and third quartiles. The upper whisker extends from the hinge to the largest value no further than 1.5 * IQR from the hinge (where IQR is the inter-quartile range). The lower whisker extends from the hinge to the smallest value (at most 1.5 * IQR of the hinge).

H Heatmap showing expression of representative marker genes across 11 ECs subtypes.

Data information: All data show mean ± s.e.m. Statistics were done using one-way Anova with Tukey corrections for multiple comparisons (A–E). All statistical analyses on single cell were performed ggpubr package in R. Wilcoxon test was used for (G), $*P < 0.05$; $**P < 0.01$; $***P < 0.001$; $****P < 0.0001$.

© 2023 The Authors

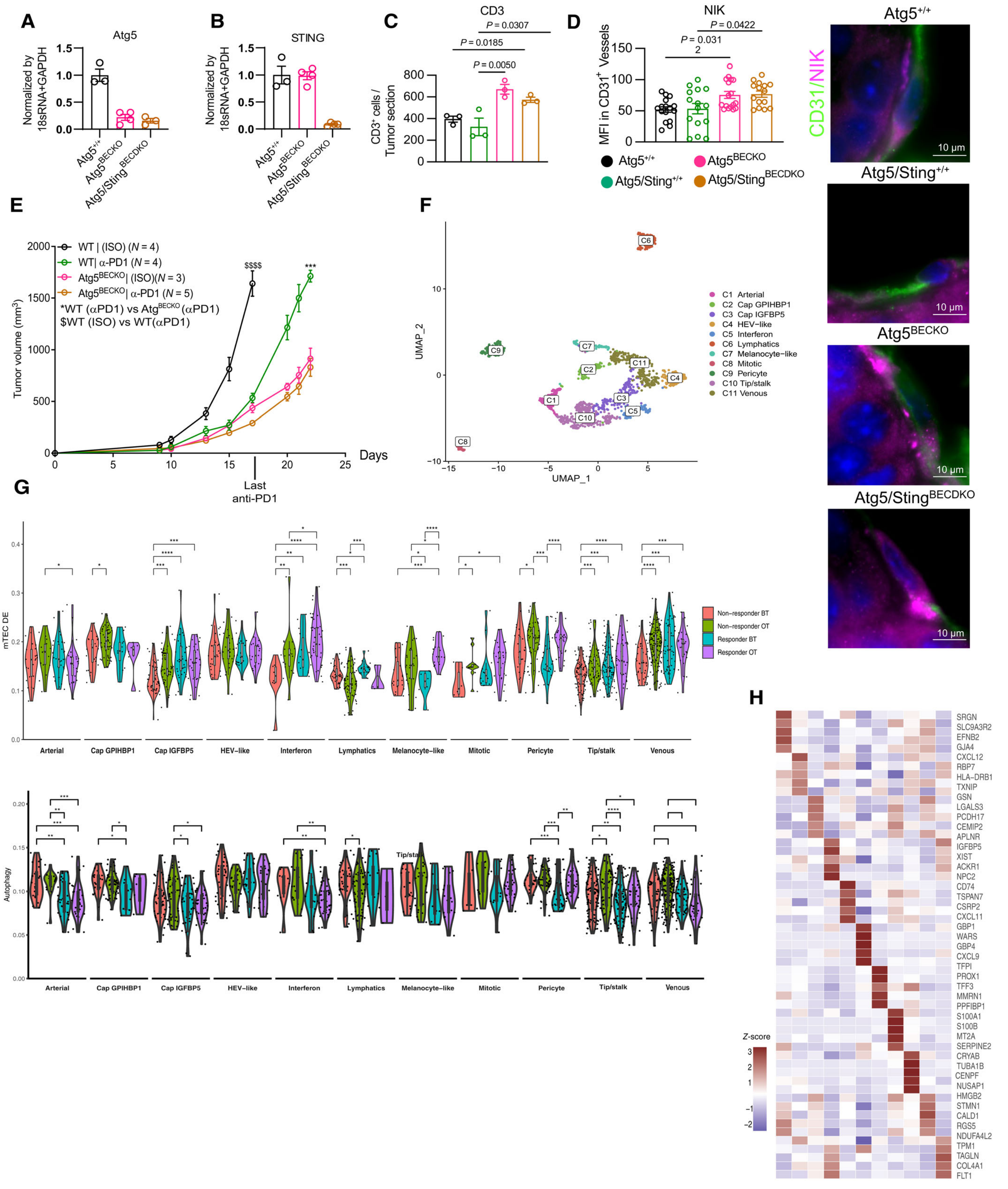
A
Atg5
Normalized by 18sRNA+GAPDH
Atg5+/+
Atg5BECKO
Atg5/StingBECDKO
B
STING
C
CD3
P = 0.0307
P = 0.0185
P = 0.0050
CD3+ cells / Tumor section
D
NIK
P = 0.0422
P = 0.031 2
MFI in CD31+ Vessels
Atg5+/+
Atg5BECKO
Atg5/Sting+/+
Atg5/StingBECDKO
CD31/NIK
10 µm
E
WT | (ISO) (N = 4)
WT| α-PD1 (N = 4)
Atg5BECKO| (ISO)(N = 3)
Atg5BECKO| α-PD1 (N = 5)
*WT (αPD1) vs AtgBECKO (αPD1)
$WT (ISO) vs WT(αPD1)
Tumor volume (mm3)
Days
Last anti-PD1
F
UMAP_1
UMAP_2
C1 Arterial
C2 Cap GPIHBP1
C3 Cap IGFBP5
C4 HEV–like
C5 Interferon
C6 Lymphatics
C7 Melanocyte–like
C8 Mitotic
C9 Pericyte
C10 Tip/stalk
C11 Venous
G
mTEC DE
Autophagy
Arterial
Cap GPIHBP1
Cap IGFBP5
HEV–like
Interferon
Lymphatics
Melanocyte–like
Mitotic
Pericyte
Tip/stalk
Venous
Non-responder BT
Non-responder OT
Responder BT
Responder OT
H
Z-score
SRGN
SLC9A3R2
EFNB2
GJA4
CXCL12
RBP7
HLA-DRB1
TXNIP
GSN
LGALS3
PCDH17
CEMIP2
APLNR
IGFBP5
XIST
ACKR1
NPC2
CD74
TSPAN7
CSRP2
CXCL11
GBP1
WARS
GBP4
CXCL9
TFPI
PROX1
TFF3
MMRN1
PPFIBP1
S100A1
S100B
MT2A
SERPINE2
CRYAB
TUBA1B
CENPF
NUSAP1
HMGB2
STMN1
CALD1
RGS5
NDUFA4L2
TPM1
TAGLN
COL4A1
FLT1

Figure EV5.
