# Supplementary material for: Tumor endothelial cell autophagy is a key vascular‐immune checkpoint in melanoma
Source: EMBO Mol Med. 2023 Nov 27;15(12):e18028. doi: 10.15252/emmm.202318028 (PMC10701618; doi:10.15252/emmm.202318028)
Supplement: Supplementary file 8 — Source Data for Figure 3 [file EMMM-15-e18028-s010.zip › figure_3_raw_data/3f,g/Read_me.docx]

PATIENT POPULATION

We collected 79 fresh tissue samples from 53 patients across 4 tumor types: breast cancer (BC; n=31, 39%), colorectal cancer (CRC; n=21, 27%), high-grade serous ovarian carcinoma (HGSOC; n=12, 15%) and non-small cell lung cancer (NSCLC; n=15, 19%). All samples were treatment-naïve tumors that have already been published (Qian, J. et al, Cell Res. 2020; Bassez, A. *et al. Nat. Med. 2021*). The local ethics committee at the University Hospital Leuven approved the single-cell study for each cancer type, and all patients provided written informed consent.

Data sets:

**Qian et al.:** Raw sequencing reads of the single-cell RNA experiments have been deposited in the ArrayExpress database at EMBL-EBI ([www.ebi.ac.uk/arrayexpress](https://www.ebi.ac.uk/arrayexpress/)) under accession number E-MTAB-8107, E-MTAB-6149 and E-MTAB-6653.

**Bassez, A. et al.:** Raw sequencing reads of all single-cell experiments (scRNA-seq, scTCR-seq and CITE-seq) have been deposited in the European Genome-phenome Archive (EGA) under study no. [EGAS00001004809](https://ega-archive.org/studies/EGAS00001004809) (with a summary of the BioKey study and patient characteristics) and with data accession no. [EGAD00001006608](https://ega-archive.org/datasets/EGAD00001006608) (to access the data itself under restricted access). Requests for accessing raw sequencing reads will be reviewed by the UZLeuven-VIB data access committee. Any data shared will be released via a Data Transfer Agreement that will include the necessary conditions to guarantee protection of personal data (according to European GDPR law).
