# Supplementary figures and images for: Tumor endothelial cell autophagy is a key vascular‐immune checkpoint in melanoma
Source: EMBO Mol Med. 2023 Nov 27;15(12):e18028. doi: 10.15252/emmm.202318028 (PMC10701618; doi:10.15252/emmm.202318028)

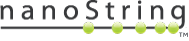

Supplement: Supplementary file 8 — Source Data for Figure 3 [file EMMM-15-e18028-s010.zip › figure_3_raw_data/3b,c/Nanostring_analysis_from_nSolver_4/WT_vs_KO_2022-10-05_07-45/resources/img/logo_nanostring.png]

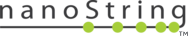

Supplement: Supplementary file 8 — Source Data for Figure 3 [file EMMM-15-e18028-s010.zip › figure_3_raw_data/3b,c/Nanostring_analysis_from_nSolver_4/WT_vs_KO_2022-10-05_07-45/resources/img/logo_nanostring_Flat_189x40.png]

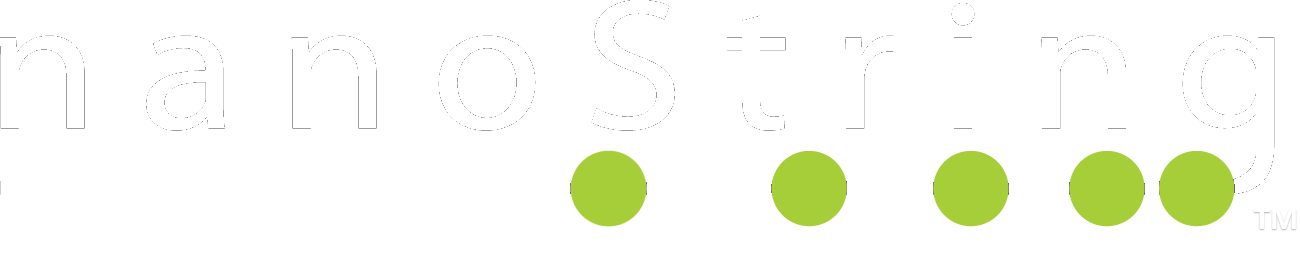

Supplement: Supplementary file 8 — Source Data for Figure 3 [file EMMM-15-e18028-s010.zip › figure_3_raw_data/3b,c/Nanostring_analysis_from_nSolver_4/WT_vs_KO_2022-10-05_07-45/resources/img/logo_nanostring_white_Flat.png]

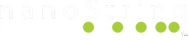

Supplement: Supplementary file 8 — Source Data for Figure 3 [file EMMM-15-e18028-s010.zip › figure_3_raw_data/3b,c/Nanostring_analysis_from_nSolver_4/WT_vs_KO_2022-10-05_07-45/resources/img/logo_nanostring_white_Flat_189x40.png]

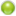

Supplement: Supplementary file 8 — Source Data for Figure 3 [file EMMM-15-e18028-s010.zip › figure_3_raw_data/3b,c/Nanostring_analysis_from_nSolver_4/WT_vs_KO_2022-10-05_07-45/resources/img/nanostring_icon.png]

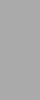

Supplement: Supplementary file 8 — Source Data for Figure 3 [file EMMM-15-e18028-s010.zip › figure_3_raw_data/3b,c/Nanostring_analysis_from_nSolver_4/WT_vs_KO_2022-10-05_07-45/resources/img/ui-bg_flat_0_aaaaaa_40x100.png]

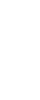

Supplement: Supplementary file 8 — Source Data for Figure 3 [file EMMM-15-e18028-s010.zip › figure_3_raw_data/3b,c/Nanostring_analysis_from_nSolver_4/WT_vs_KO_2022-10-05_07-45/resources/img/ui-bg_flat_75_ffffff_40x100.png]

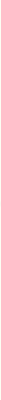

Supplement: Supplementary file 8 — Source Data for Figure 3 [file EMMM-15-e18028-s010.zip › figure_3_raw_data/3b,c/Nanostring_analysis_from_nSolver_4/WT_vs_KO_2022-10-05_07-45/resources/img/ui-bg_glass_55_fbf9ee_1x400.png]

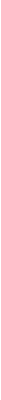

Supplement: Supplementary file 8 — Source Data for Figure 3 [file EMMM-15-e18028-s010.zip › figure_3_raw_data/3b,c/Nanostring_analysis_from_nSolver_4/WT_vs_KO_2022-10-05_07-45/resources/img/ui-bg_glass_65_ffffff_1x400.png]

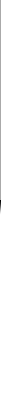

Supplement: Supplementary file 8 — Source Data for Figure 3 [file EMMM-15-e18028-s010.zip › figure_3_raw_data/3b,c/Nanostring_analysis_from_nSolver_4/WT_vs_KO_2022-10-05_07-45/resources/img/ui-bg_glass_75_dadada_1x400.png]

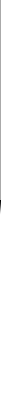

Supplement: Supplementary file 8 — Source Data for Figure 3 [file EMMM-15-e18028-s010.zip › figure_3_raw_data/3b,c/Nanostring_analysis_from_nSolver_4/WT_vs_KO_2022-10-05_07-45/resources/img/ui-bg_glass_75_e6e6e6_1x400.png]

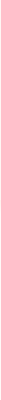

Supplement: Supplementary file 8 — Source Data for Figure 3 [file EMMM-15-e18028-s010.zip › figure_3_raw_data/3b,c/Nanostring_analysis_from_nSolver_4/WT_vs_KO_2022-10-05_07-45/resources/img/ui-bg_glass_95_fef1ec_1x400.png]

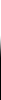

Supplement: Supplementary file 8 — Source Data for Figure 3 [file EMMM-15-e18028-s010.zip › figure_3_raw_data/3b,c/Nanostring_analysis_from_nSolver_4/WT_vs_KO_2022-10-05_07-45/resources/img/ui-bg_highlight-soft_75_cccccc_1x100.png]

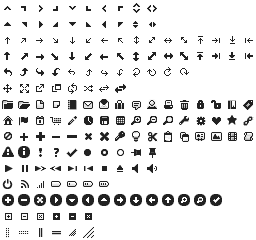

Supplement: Supplementary file 8 — Source Data for Figure 3 [file EMMM-15-e18028-s010.zip › figure_3_raw_data/3b,c/Nanostring_analysis_from_nSolver_4/WT_vs_KO_2022-10-05_07-45/resources/img/ui-icons_222222_256x240.png]

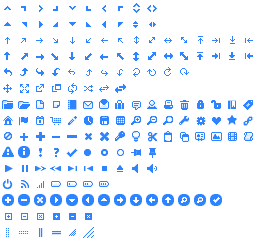

Supplement: Supplementary file 8 — Source Data for Figure 3 [file EMMM-15-e18028-s010.zip › figure_3_raw_data/3b,c/Nanostring_analysis_from_nSolver_4/WT_vs_KO_2022-10-05_07-45/resources/img/ui-icons_2e83ff_256x240.png]

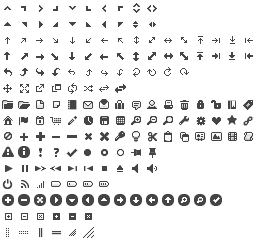

Supplement: Supplementary file 8 — Source Data for Figure 3 [file EMMM-15-e18028-s010.zip › figure_3_raw_data/3b,c/Nanostring_analysis_from_nSolver_4/WT_vs_KO_2022-10-05_07-45/resources/img/ui-icons_454545_256x240.png]

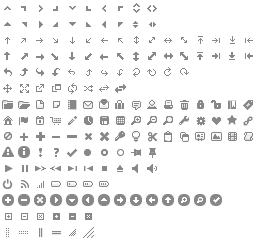

Supplement: Supplementary file 8 — Source Data for Figure 3 [file EMMM-15-e18028-s010.zip › figure_3_raw_data/3b,c/Nanostring_analysis_from_nSolver_4/WT_vs_KO_2022-10-05_07-45/resources/img/ui-icons_888888_256x240.png]

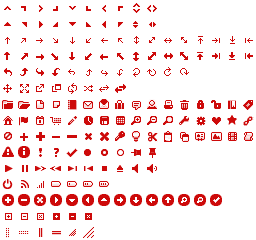

Supplement: Supplementary file 8 — Source Data for Figure 3 [file EMMM-15-e18028-s010.zip › figure_3_raw_data/3b,c/Nanostring_analysis_from_nSolver_4/WT_vs_KO_2022-10-05_07-45/resources/img/ui-icons_cd0a0a_256x240.png]

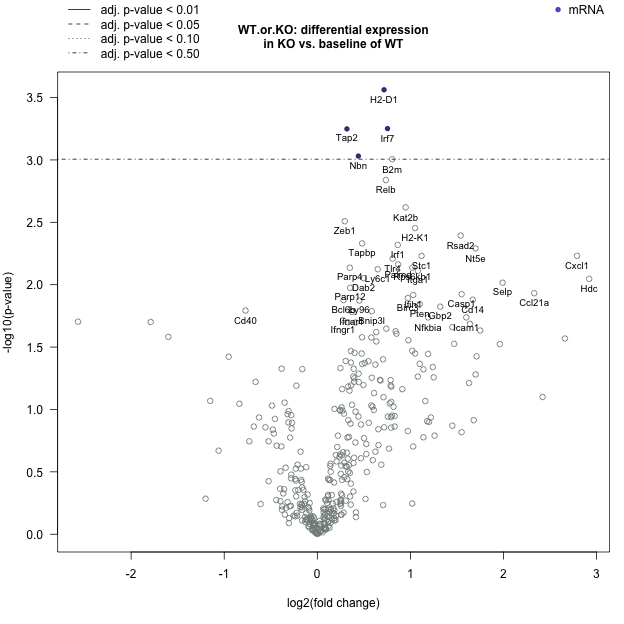

Supplement: Supplementary file 8 — Source Data for Figure 3 [file EMMM-15-e18028-s010.zip › figure_3_raw_data/3b,c/Nanostring_analysis_from_nSolver_4/WT_vs_KO_2022-10-05_07-45/results/DE/volcano_plotWT.or.KOKO.png]

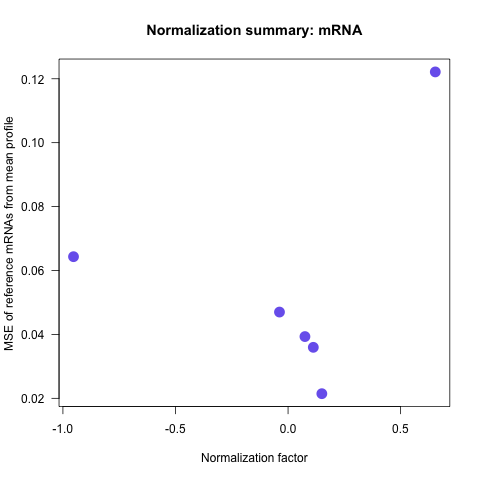

Supplement: Supplementary file 8 — Source Data for Figure 3 [file EMMM-15-e18028-s010.zip › figure_3_raw_data/3b,c/Nanostring_analysis_from_nSolver_4/WT_vs_KO_2022-10-05_07-45/results/Normalization/gx_normalization_results.png]

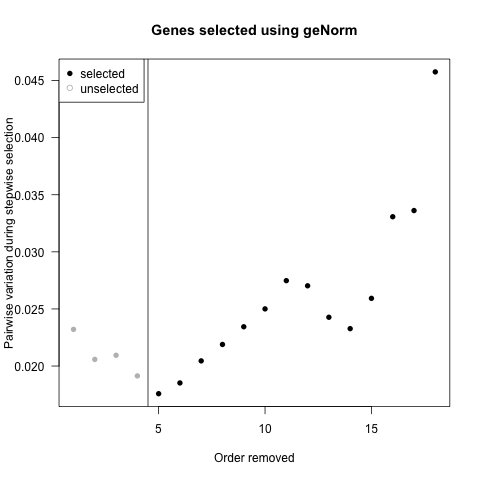

Supplement: Supplementary file 8 — Source Data for Figure 3 [file EMMM-15-e18028-s010.zip › figure_3_raw_data/3b,c/Nanostring_analysis_from_nSolver_4/WT_vs_KO_2022-10-05_07-45/results/Normalization/HK_selection_details_-_pairwise_variance.png]

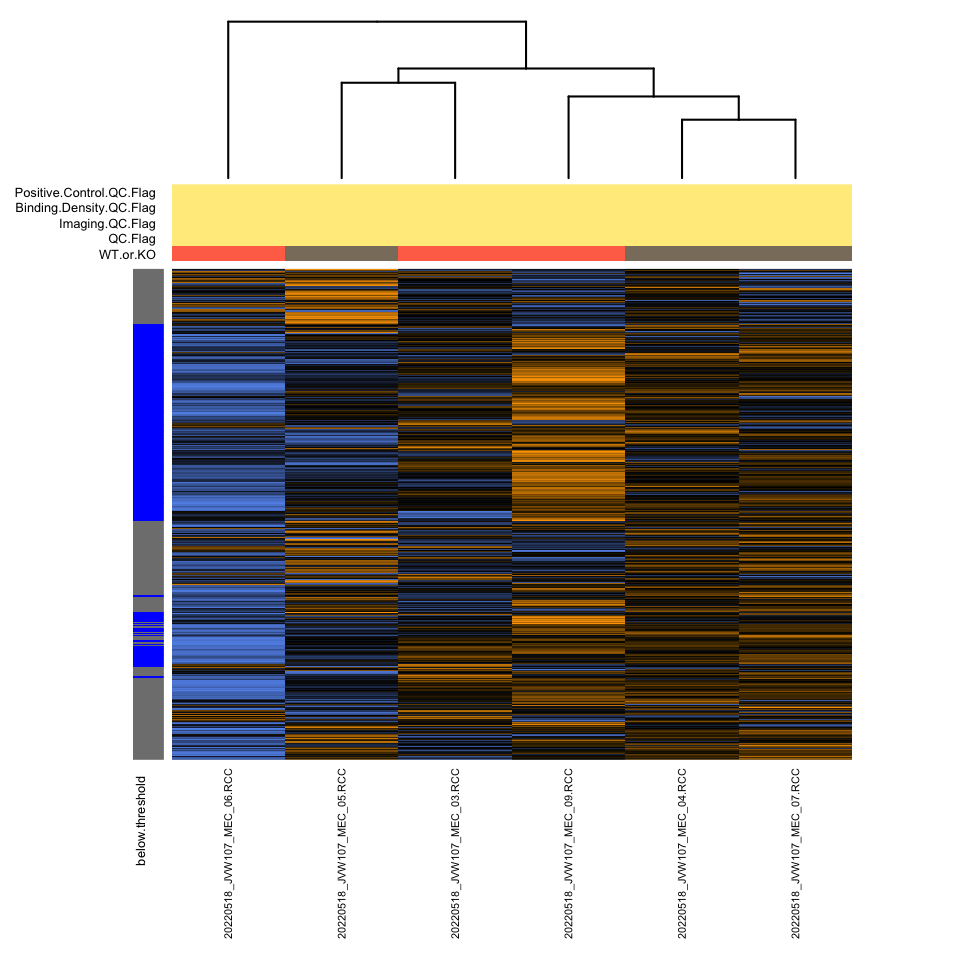

Supplement: Supplementary file 8 — Source Data for Figure 3 [file EMMM-15-e18028-s010.zip › figure_3_raw_data/3b,c/Nanostring_analysis_from_nSolver_4/WT_vs_KO_2022-10-05_07-45/results/QC/heatmap_of_all_genes_X_samples.png]

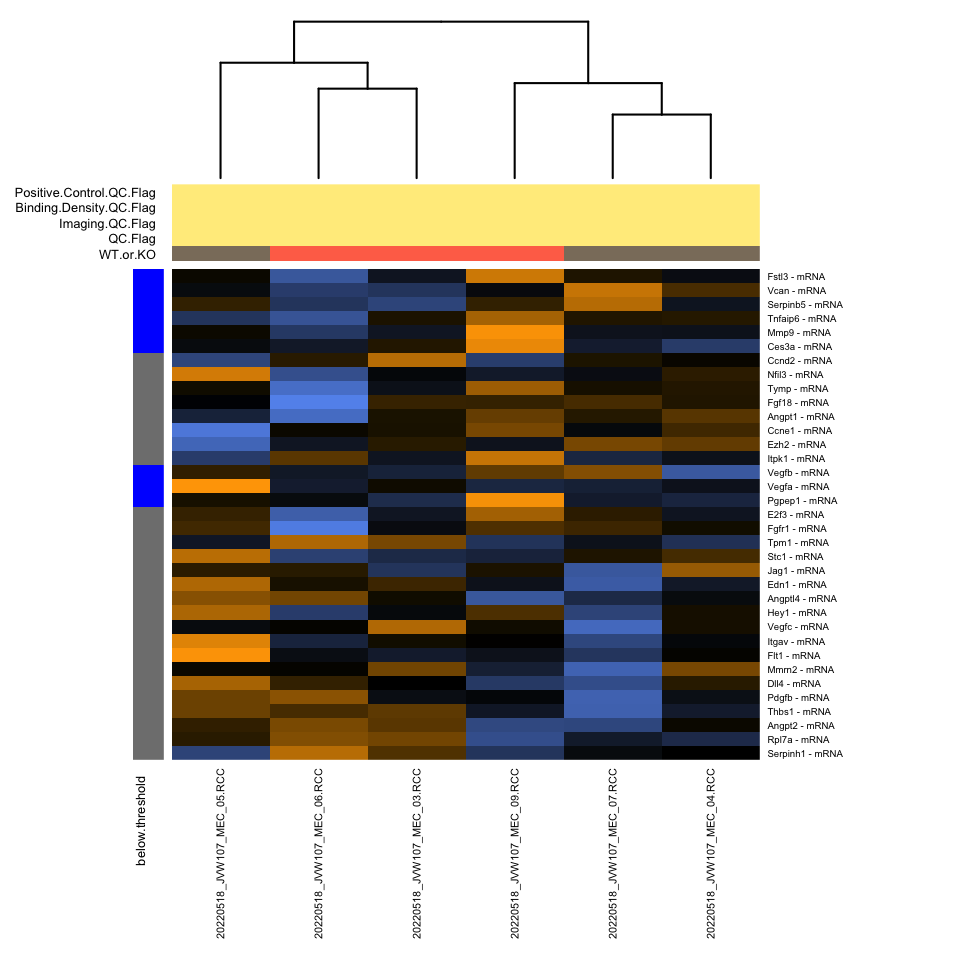

Supplement: Supplementary file 8 — Source Data for Figure 3 [file EMMM-15-e18028-s010.zip › figure_3_raw_data/3b,c/Nanostring_analysis_from_nSolver_4/WT_vs_KO_2022-10-05_07-45/results/QC/heatmap_of_data_-_Angiogenesis.png]

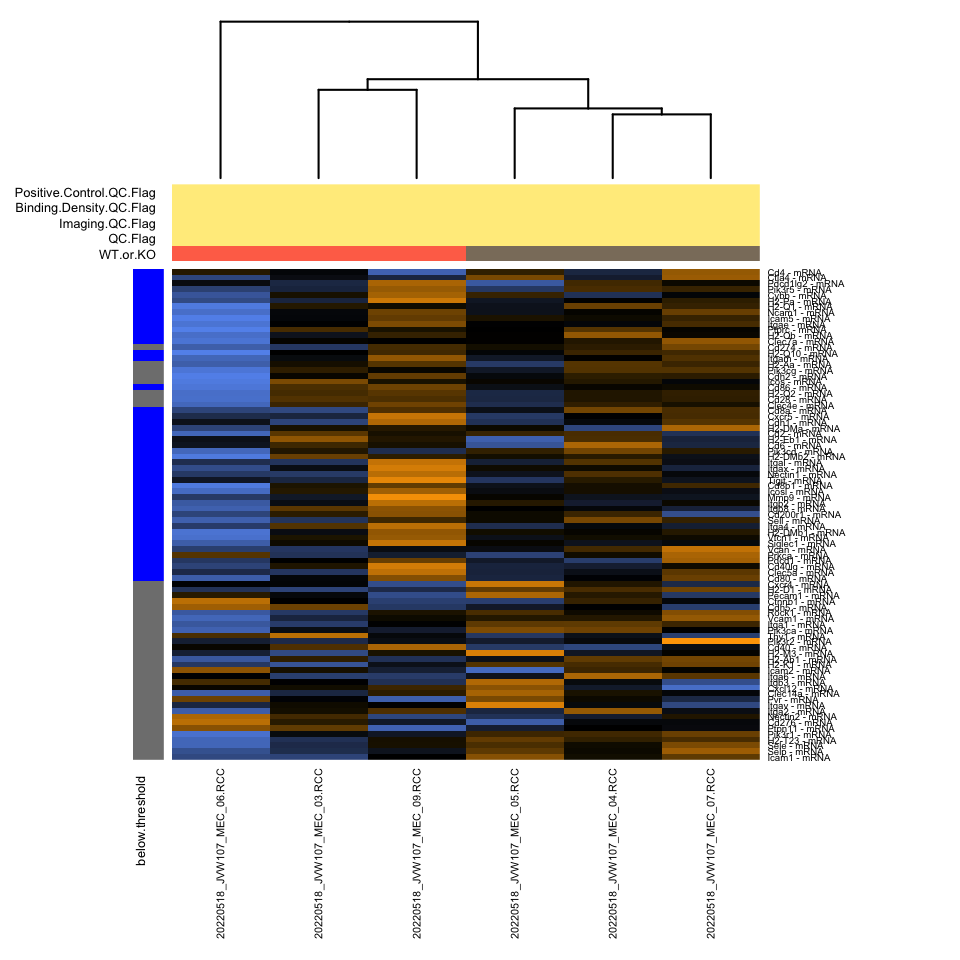

Supplement: Supplementary file 8 — Source Data for Figure 3 [file EMMM-15-e18028-s010.zip › figure_3_raw_data/3b,c/Nanostring_analysis_from_nSolver_4/WT_vs_KO_2022-10-05_07-45/results/QC/heatmap_of_data_-_Immune.Cell.Adhesion.and.Migration.png]

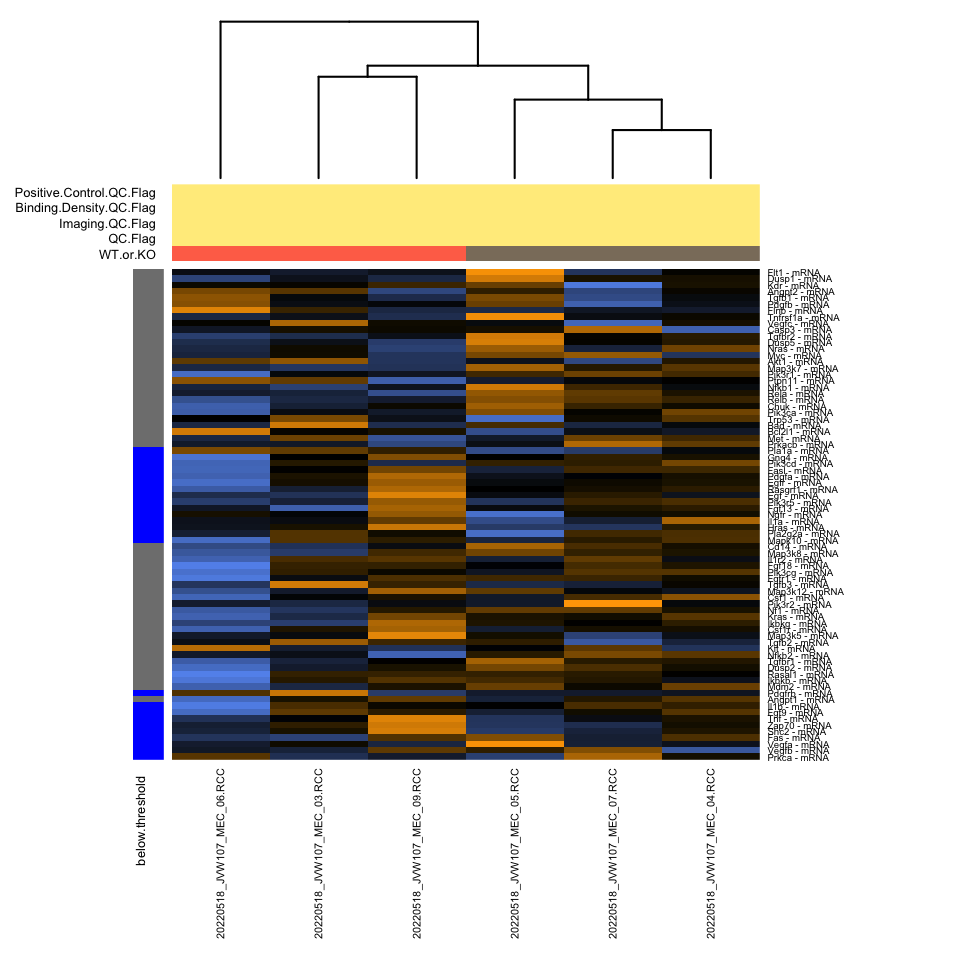

Supplement: Supplementary file 8 — Source Data for Figure 3 [file EMMM-15-e18028-s010.zip › figure_3_raw_data/3b,c/Nanostring_analysis_from_nSolver_4/WT_vs_KO_2022-10-05_07-45/results/QC/heatmap_of_data_-_MAPK.png]

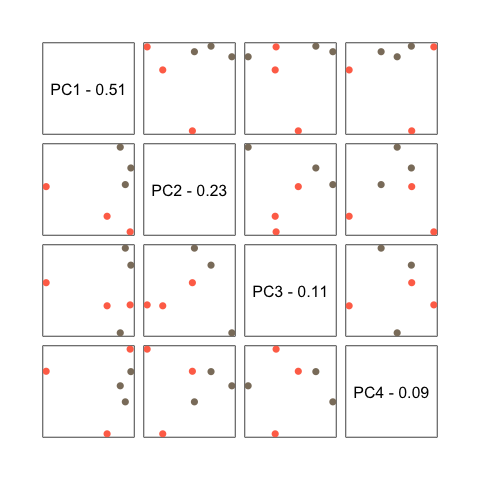

Supplement: Supplementary file 8 — Source Data for Figure 3 [file EMMM-15-e18028-s010.zip › figure_3_raw_data/3b,c/Nanostring_analysis_from_nSolver_4/WT_vs_KO_2022-10-05_07-45/results/QC/PCA_colored_by_WT.or.KO_-_Antigen.Presentation.png]

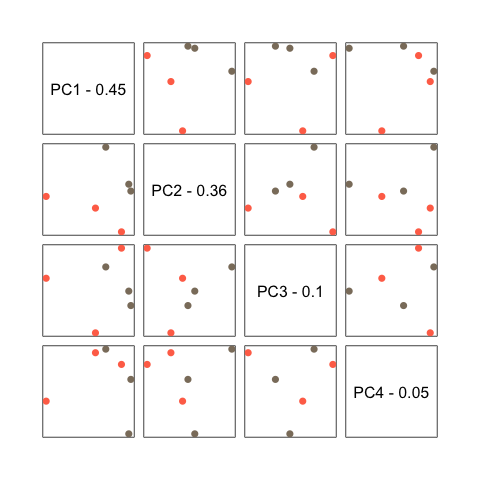

Supplement: Supplementary file 8 — Source Data for Figure 3 [file EMMM-15-e18028-s010.zip › figure_3_raw_data/3b,c/Nanostring_analysis_from_nSolver_4/WT_vs_KO_2022-10-05_07-45/results/QC/PCA_colored_by_WT.or.KO_-_Autophagy.png]

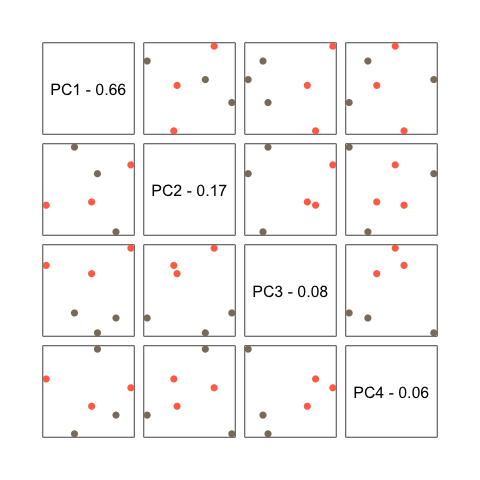

Supplement: Supplementary file 8 — Source Data for Figure 3 [file EMMM-15-e18028-s010.zip › figure_3_raw_data/3b,c/Nanostring_analysis_from_nSolver_4/WT_vs_KO_2022-10-05_07-45/results/QC/PCA_colored_by_WT.or.KO_-_DNA.Damage.Repair.png]

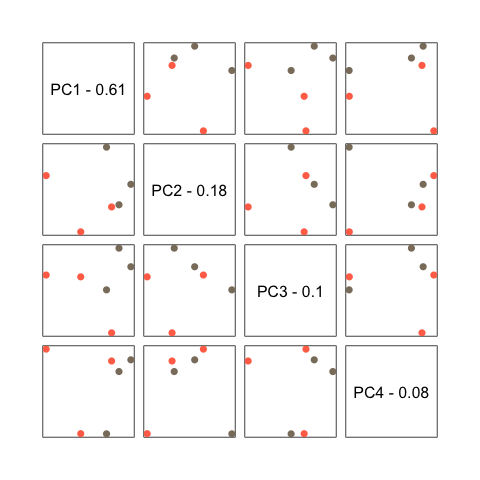

Supplement: Supplementary file 8 — Source Data for Figure 3 [file EMMM-15-e18028-s010.zip › figure_3_raw_data/3b,c/Nanostring_analysis_from_nSolver_4/WT_vs_KO_2022-10-05_07-45/results/QC/PCA_colored_by_WT.or.KO_-_Interferon.Signaling.png]

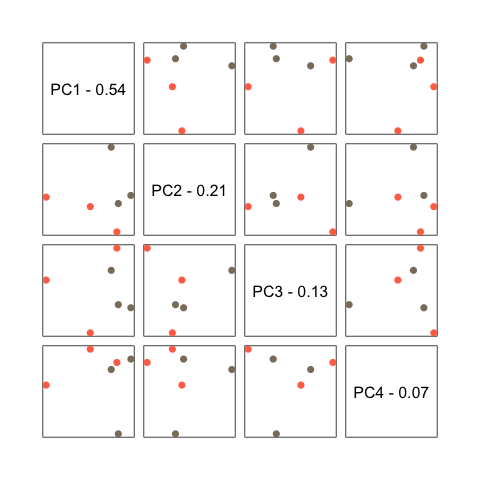

Supplement: Supplementary file 8 — Source Data for Figure 3 [file EMMM-15-e18028-s010.zip › figure_3_raw_data/3b,c/Nanostring_analysis_from_nSolver_4/WT_vs_KO_2022-10-05_07-45/results/QC/PCA_colored_by_WT.or.KO_-_JAK.STAT.Signaling.png]

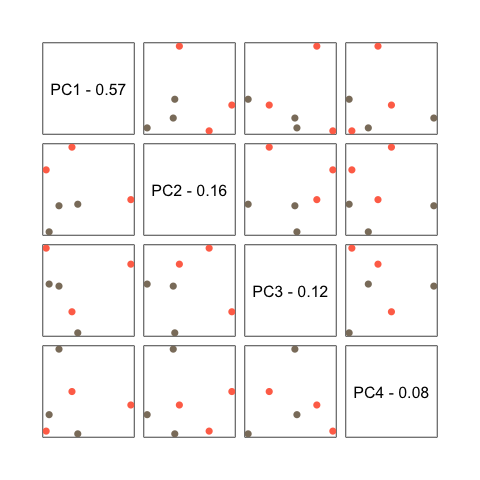

Supplement: Supplementary file 8 — Source Data for Figure 3 [file EMMM-15-e18028-s010.zip › figure_3_raw_data/3b,c/Nanostring_analysis_from_nSolver_4/WT_vs_KO_2022-10-05_07-45/results/QC/PCA_colored_by_WT.or.KO_-_Lymphoid.Compartment.png]

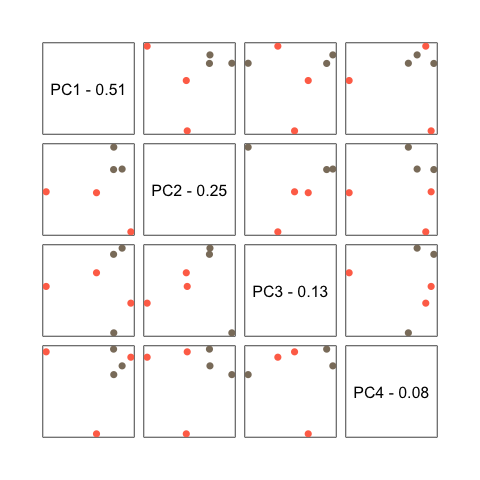

Supplement: Supplementary file 8 — Source Data for Figure 3 [file EMMM-15-e18028-s010.zip › figure_3_raw_data/3b,c/Nanostring_analysis_from_nSolver_4/WT_vs_KO_2022-10-05_07-45/results/QC/PCA_colored_by_WT.or.KO_-_NF.kappaB.Signaling.png]

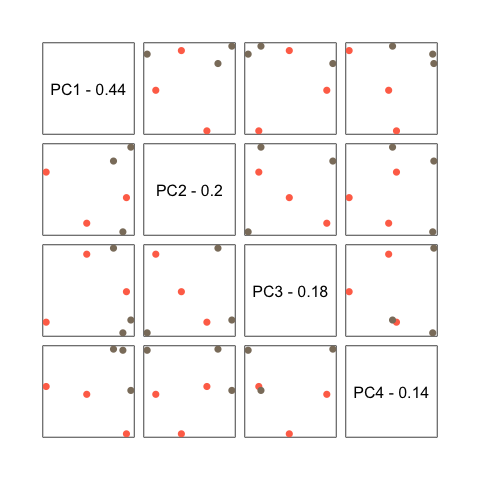

Supplement: Supplementary file 8 — Source Data for Figure 3 [file EMMM-15-e18028-s010.zip › figure_3_raw_data/3b,c/Nanostring_analysis_from_nSolver_4/WT_vs_KO_2022-10-05_07-45/results/QC/PCA_colored_by_WT.or.KO_-_TGF.beta.Signaling.png]

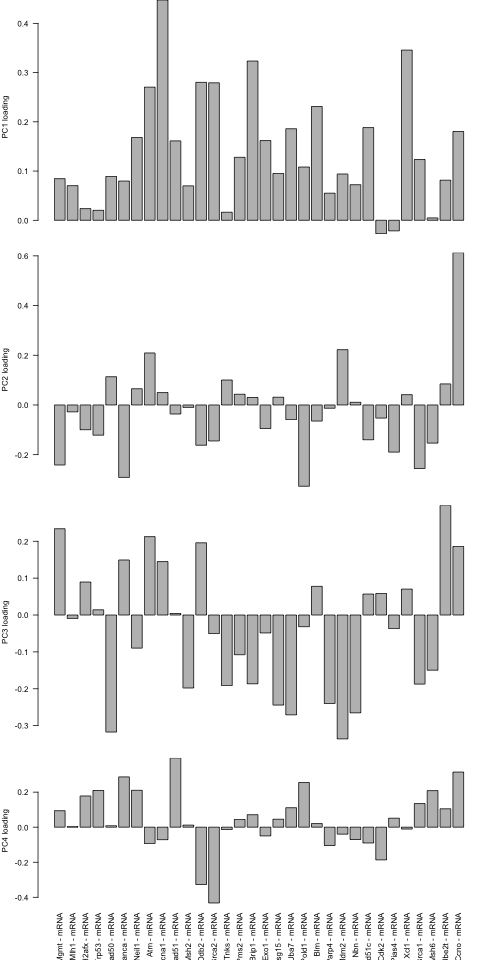

Supplement: Supplementary file 8 — Source Data for Figure 3 [file EMMM-15-e18028-s010.zip › figure_3_raw_data/3b,c/Nanostring_analysis_from_nSolver_4/WT_vs_KO_2022-10-05_07-45/results/QC/PCA_loadings_-_DNA.Damage.Repair.png]

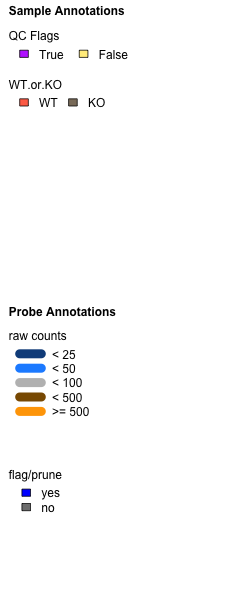

Supplement: Supplementary file 8 — Source Data for Figure 3 [file EMMM-15-e18028-s010.zip › figure_3_raw_data/3b,c/Nanostring_analysis_from_nSolver_4/WT_vs_KO_2022-10-05_07-45/results/QC/raw_data_heatmap_-_color_key.png]

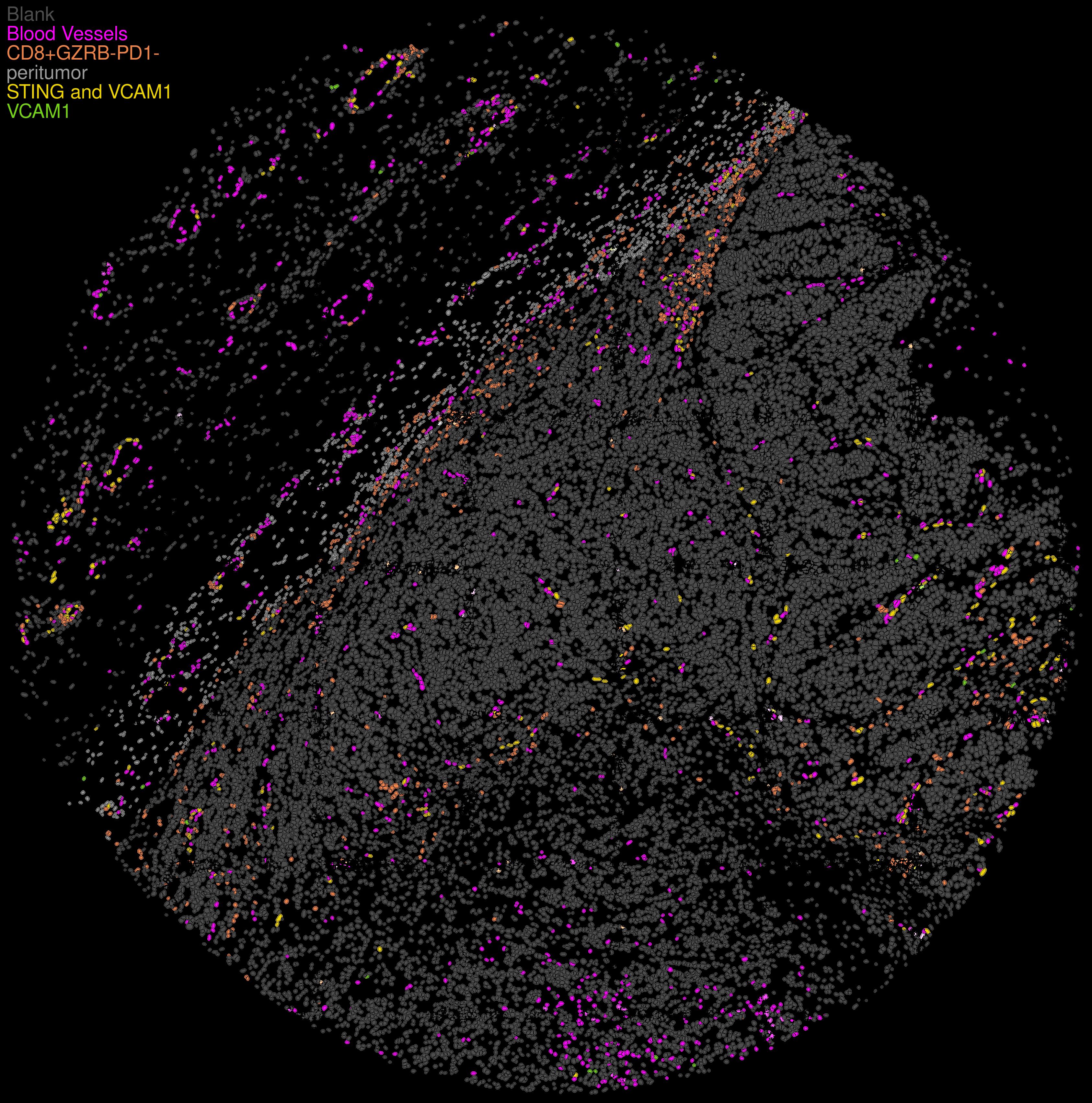

Supplement: Supplementary file 12 — Source Data for Figure 7 [file EMMM-15-e18028-s013.zip › figure_7_raw_data/7e/fig7e_raw_image_nonresponder.jpeg]

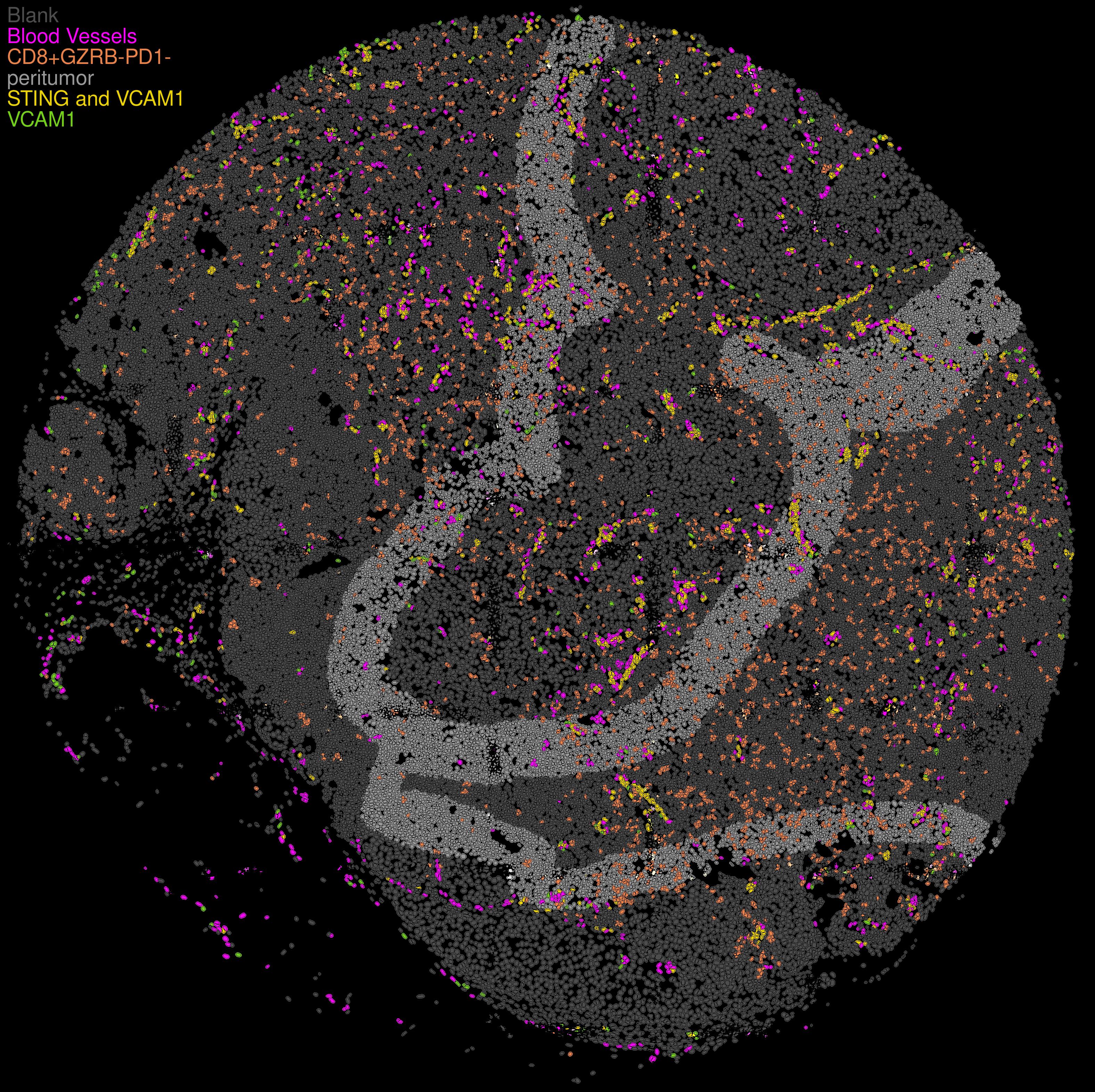

Supplement: Supplementary file 12 — Source Data for Figure 7 [file EMMM-15-e18028-s013.zip › figure_7_raw_data/7e/fig7e_raw_image_responder_.jpeg]

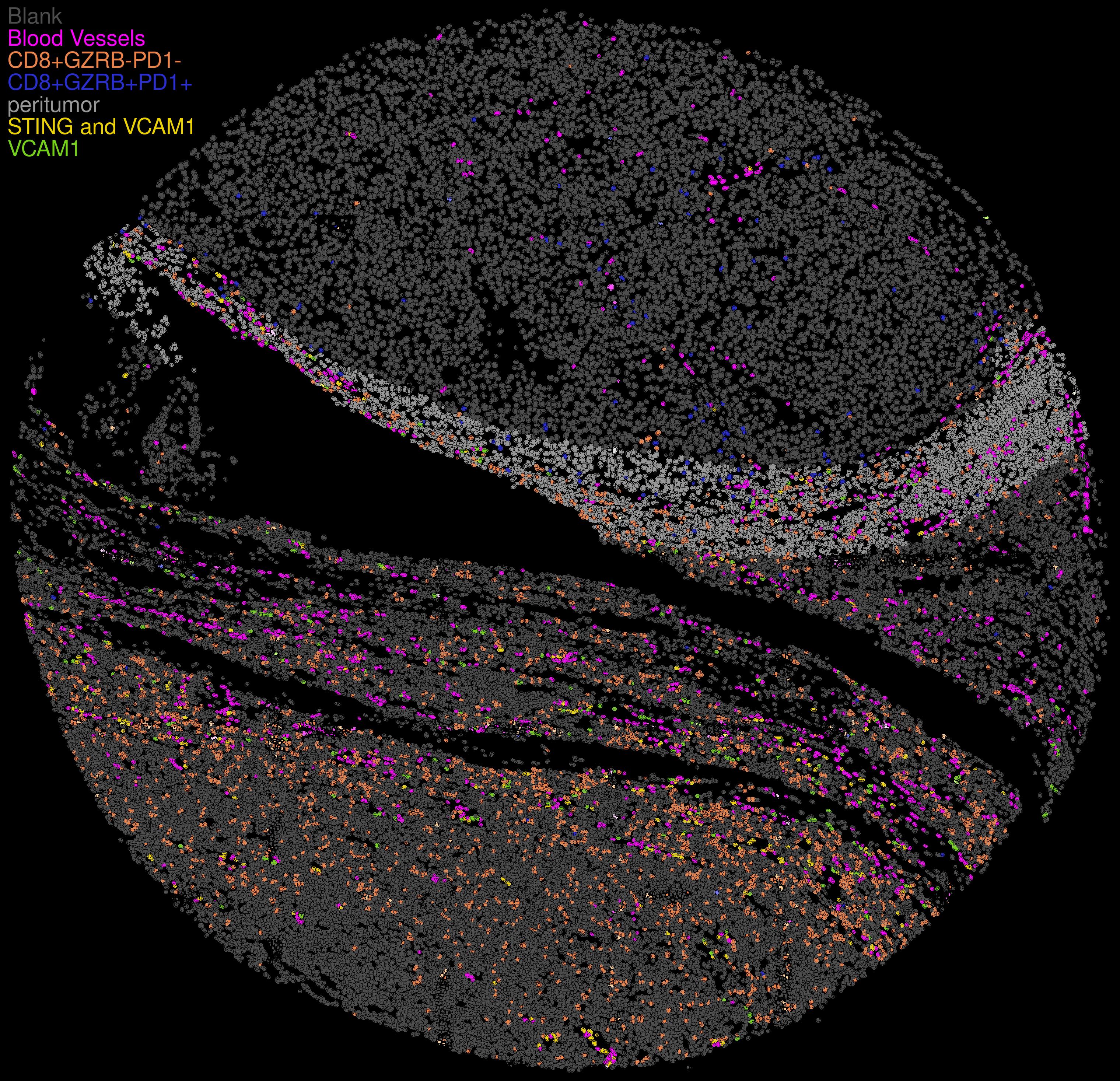

Supplement: Supplementary file 12 — Source Data for Figure 7 [file EMMM-15-e18028-s013.zip › figure_7_raw_data/7f/fig7f_raw_image_nonresponder.jpeg]

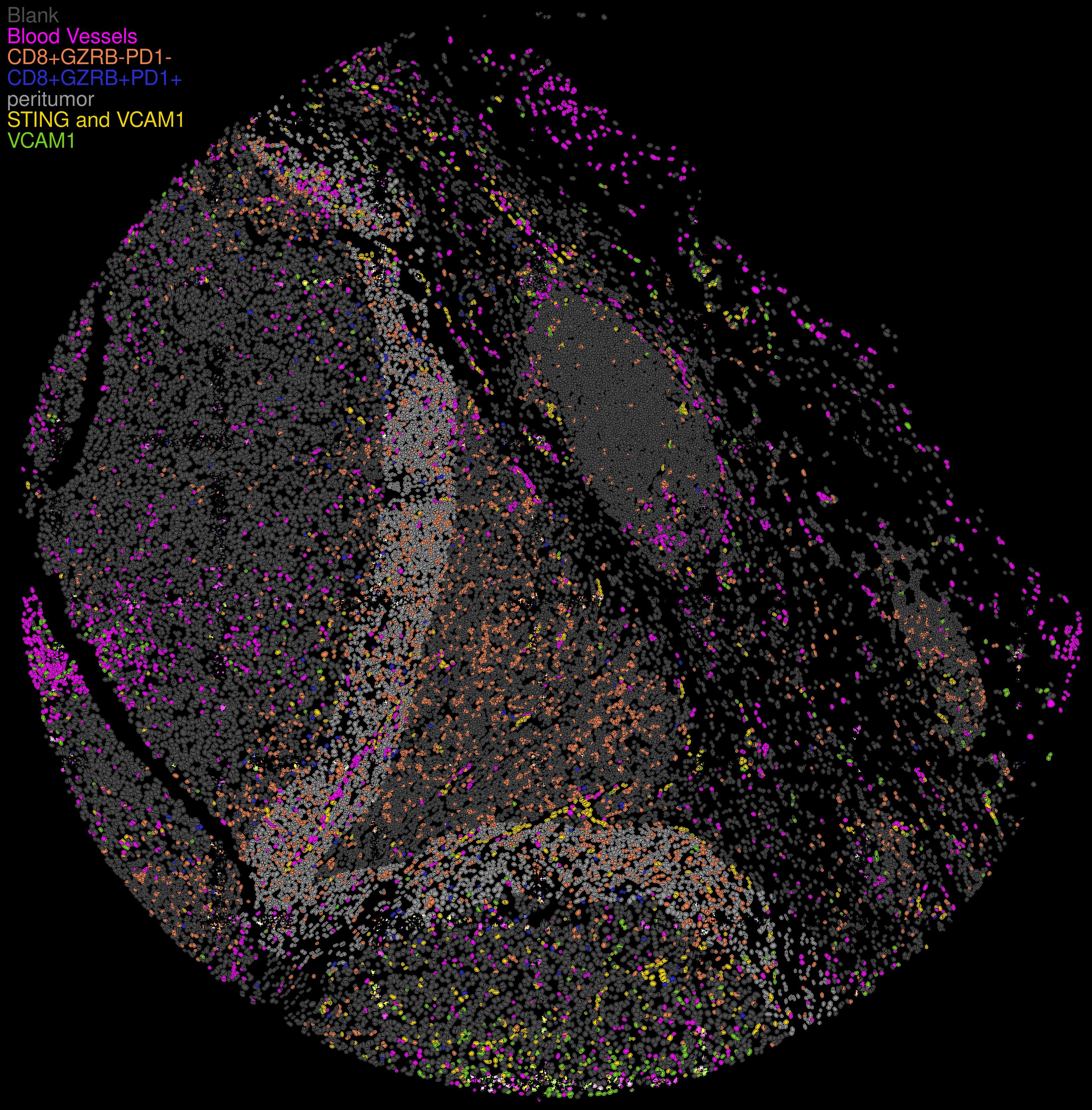

Supplement: Supplementary file 12 — Source Data for Figure 7 [file EMMM-15-e18028-s013.zip › figure_7_raw_data/7f/fig7f_raw_image_responder.jpeg]

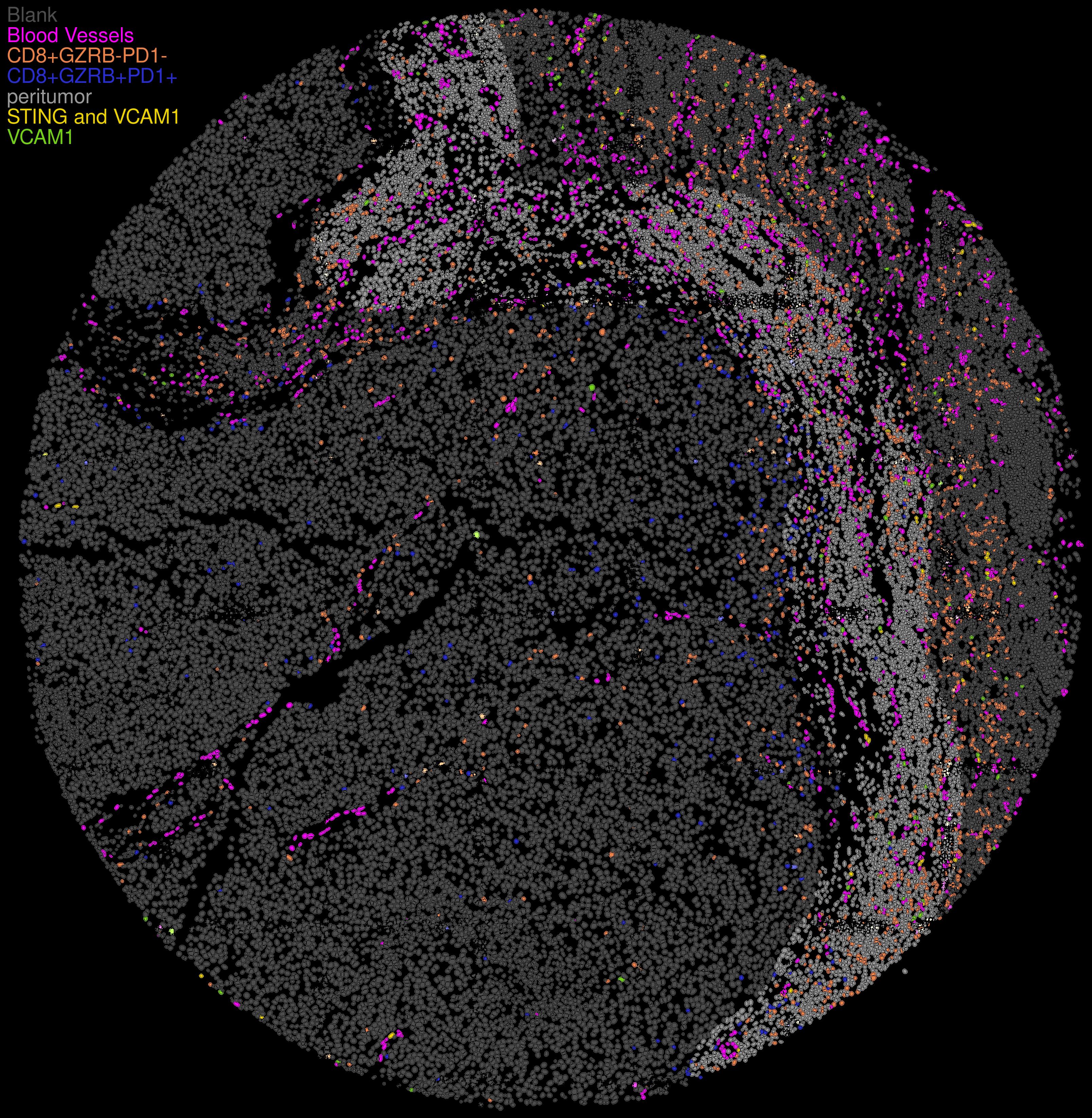

Supplement: Supplementary file 12 — Source Data for Figure 7 [file EMMM-15-e18028-s013.zip › figure_7_raw_data/7h/fig7h_raw_image_nonresponder.jpeg]

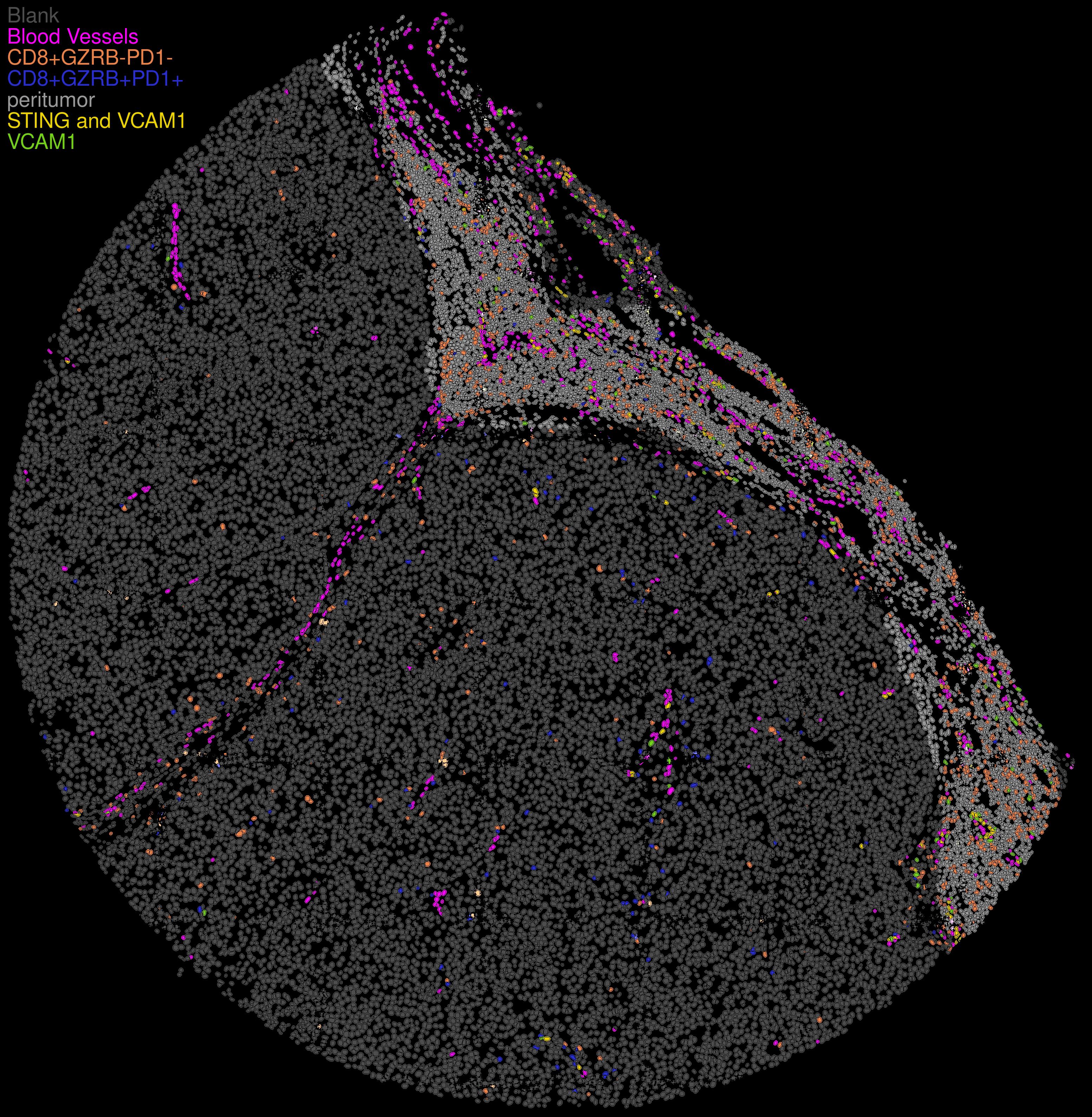

Supplement: Supplementary file 12 — Source Data for Figure 7 [file EMMM-15-e18028-s013.zip › figure_7_raw_data/7h/fig7h_raw_image_responder.jpeg]
