# Supplementary material for: Tumor endothelial cell autophagy is a key vascular‐immune checkpoint in melanoma
Source: EMBO Mol Med. 2023 Nov 27;15(12):e18028. doi: 10.15252/emmm.202318028 (PMC10701618; doi:10.15252/emmm.202318028)
Supplement: Supplementary file 9 — Source Data for Figure 4 [file EMMM-15-e18028-s004.zip › figure_4_raw_data/4g/Uncut_blot_figure_4g.pptx]

## Slide 1
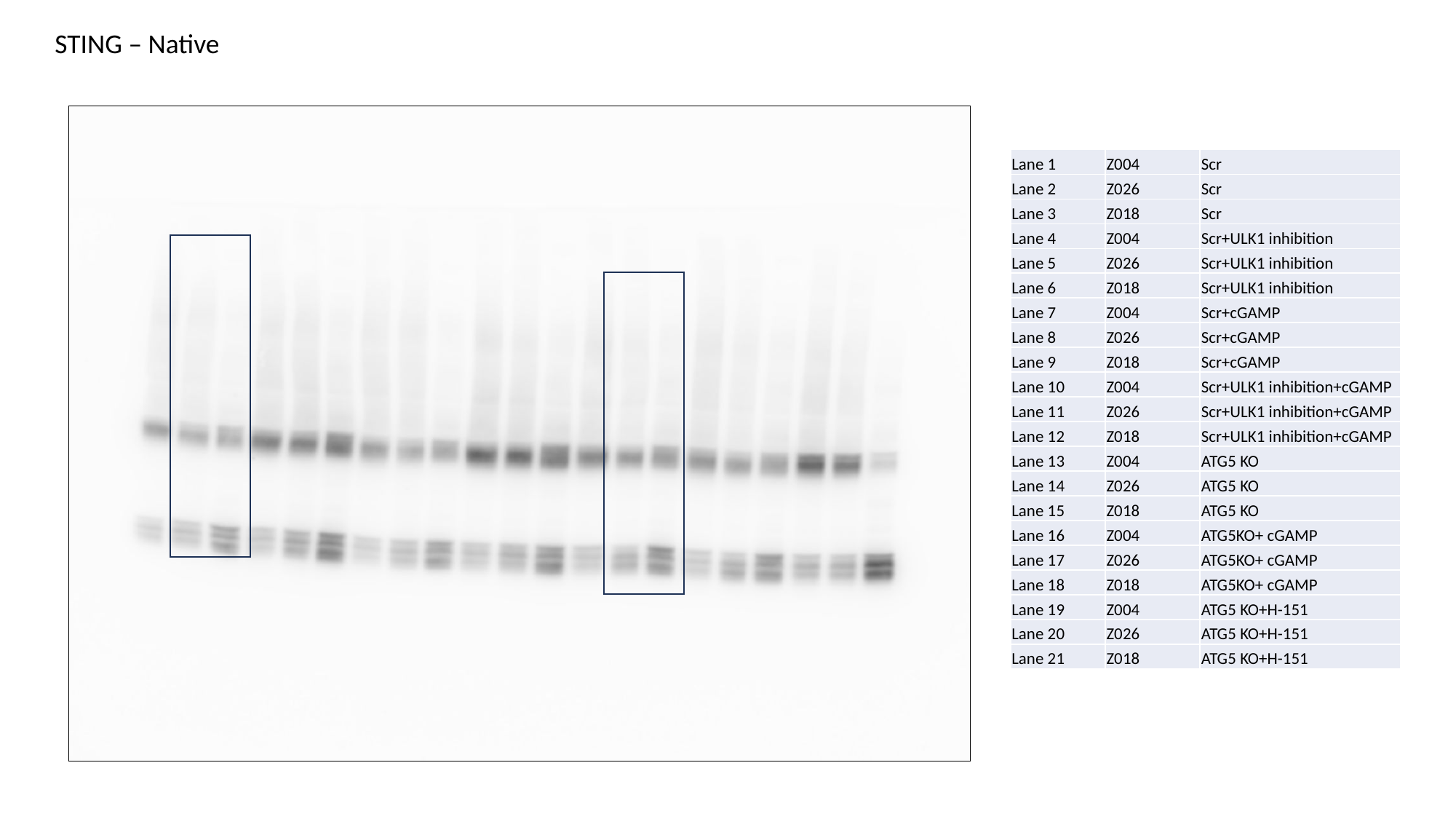

STING – Native
| Lane 1 | Z004 | Scr |
| --- | --- | --- |
| Lane 2 | Z026 | Scr |
| Lane 3 | Z018 | Scr |
| Lane 4 | Z004 | Scr+ULK1 inhibition |
| Lane 5 | Z026 | Scr+ULK1 inhibition |
| Lane 6 | Z018 | Scr+ULK1 inhibition |
| Lane 7 | Z004 | Scr+cGAMP |
| Lane 8 | Z026 | Scr+cGAMP |
| Lane 9 | Z018 | Scr+cGAMP |
| Lane 10 | Z004 | Scr+ULK1 inhibition+cGAMP |
| Lane 11 | Z026 | Scr+ULK1 inhibition+cGAMP |
| Lane 12 | Z018 | Scr+ULK1 inhibition+cGAMP |
| Lane 13 | Z004 | ATG5 KO |
| Lane 14 | Z026 | ATG5 KO |
| Lane 15 | Z018 | ATG5 KO |
| Lane 16 | Z004 | ATG5KO+ cGAMP |
| Lane 17 | Z026 | ATG5KO+ cGAMP |
| Lane 18 | Z018 | ATG5KO+ cGAMP |
| Lane 19 | Z004 | ATG5 KO+H-151 |
| Lane 20 | Z026 | ATG5 KO+H-151 |
| Lane 21 | Z018 | ATG5 KO+H-151 |

## Slide 2
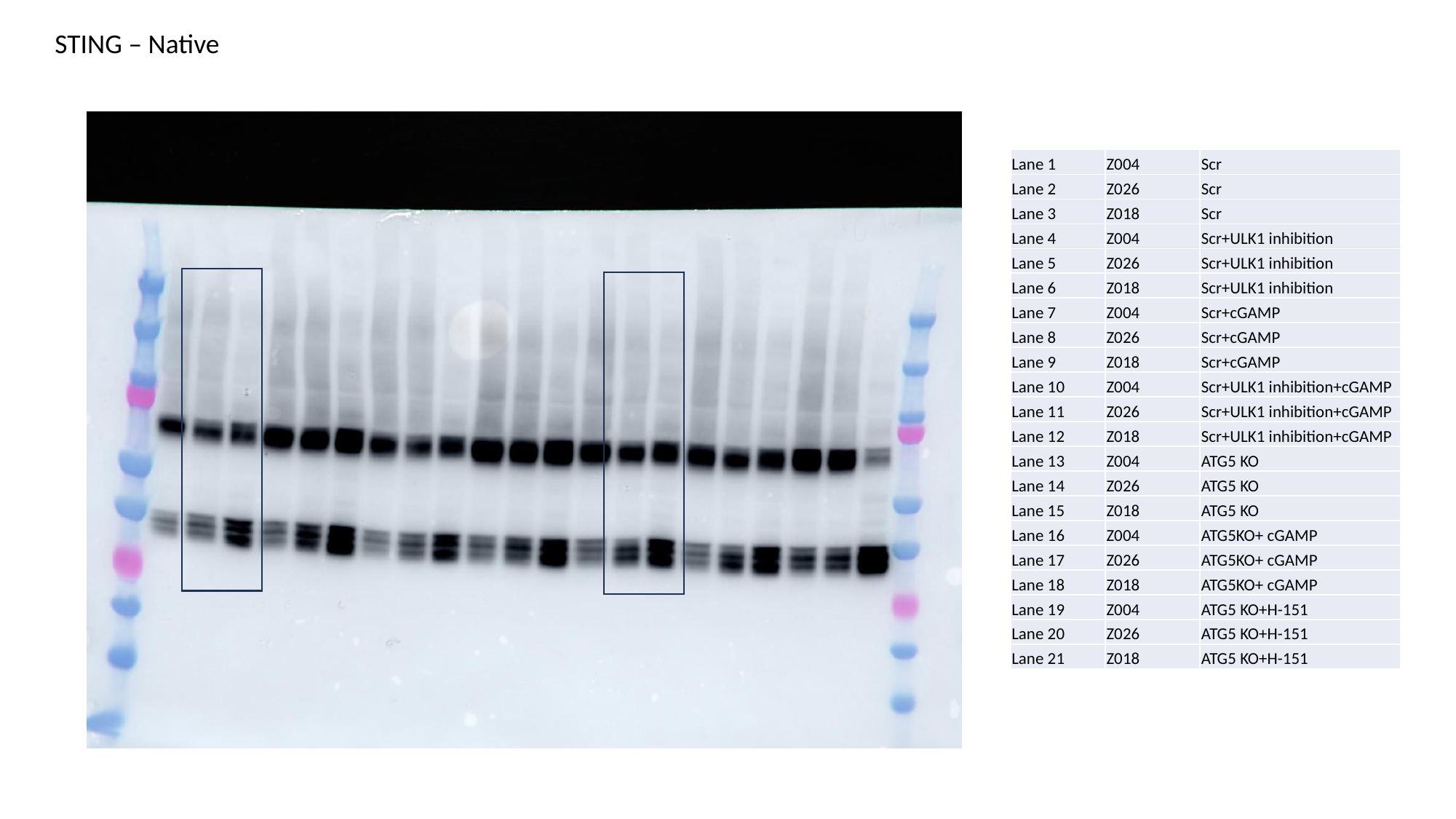

STING – Native
| Lane 1 | Z004 | Scr |
| --- | --- | --- |
| Lane 2 | Z026 | Scr |
| Lane 3 | Z018 | Scr |
| Lane 4 | Z004 | Scr+ULK1 inhibition |
| Lane 5 | Z026 | Scr+ULK1 inhibition |
| Lane 6 | Z018 | Scr+ULK1 inhibition |
| Lane 7 | Z004 | Scr+cGAMP |
| Lane 8 | Z026 | Scr+cGAMP |
| Lane 9 | Z018 | Scr+cGAMP |
| Lane 10 | Z004 | Scr+ULK1 inhibition+cGAMP |
| Lane 11 | Z026 | Scr+ULK1 inhibition+cGAMP |
| Lane 12 | Z018 | Scr+ULK1 inhibition+cGAMP |
| Lane 13 | Z004 | ATG5 KO |
| Lane 14 | Z026 | ATG5 KO |
| Lane 15 | Z018 | ATG5 KO |
| Lane 16 | Z004 | ATG5KO+ cGAMP |
| Lane 17 | Z026 | ATG5KO+ cGAMP |
| Lane 18 | Z018 | ATG5KO+ cGAMP |
| Lane 19 | Z004 | ATG5 KO+H-151 |
| Lane 20 | Z026 | ATG5 KO+H-151 |
| Lane 21 | Z018 | ATG5 KO+H-151 |

## Slide 3
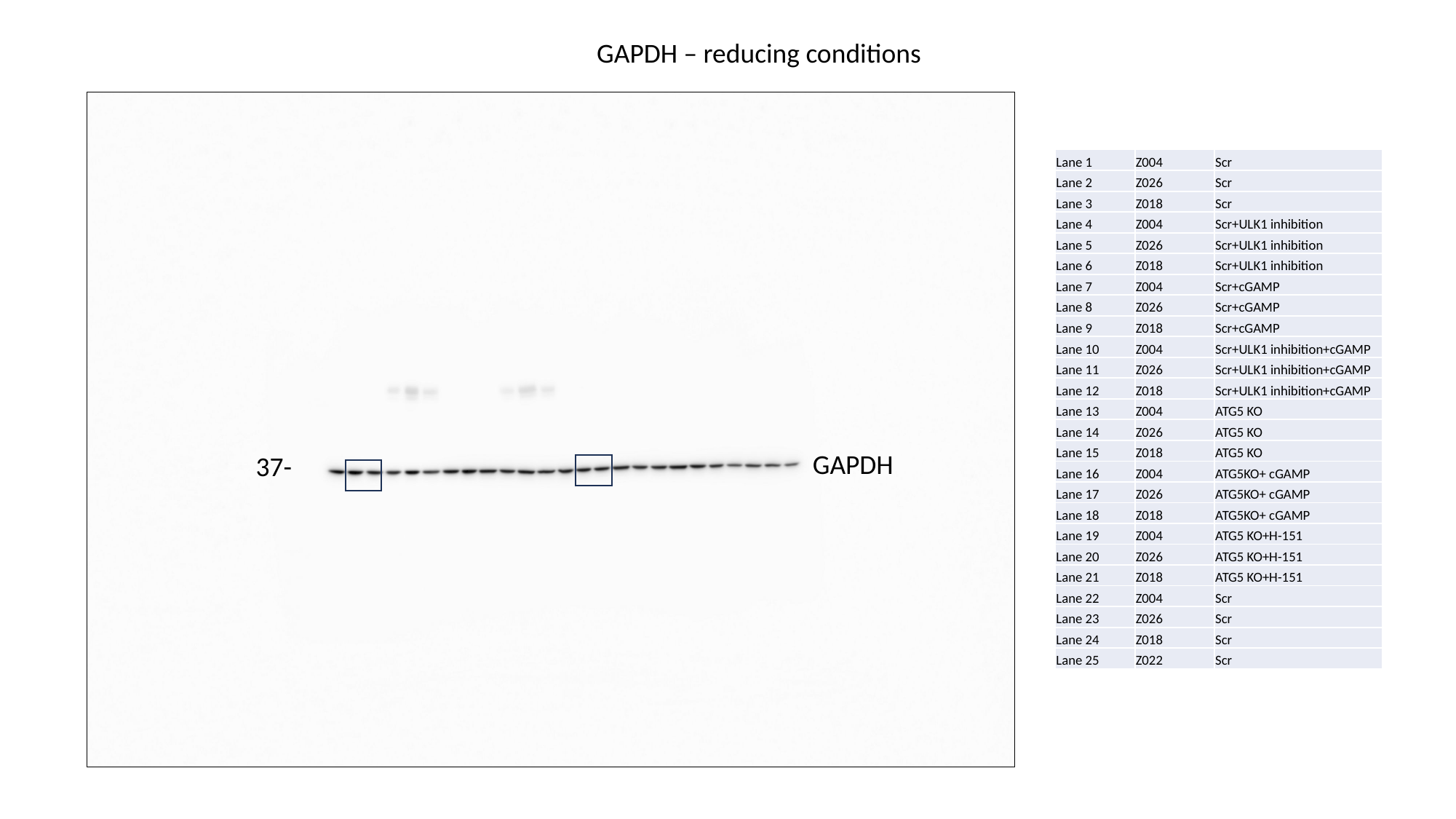

GAPDH – reducing conditions
| Lane 1 | Z004 | Scr |
| --- | --- | --- |
| Lane 2 | Z026 | Scr |
| Lane 3 | Z018 | Scr |
| Lane 4 | Z004 | Scr+ULK1 inhibition |
| Lane 5 | Z026 | Scr+ULK1 inhibition |
| Lane 6 | Z018 | Scr+ULK1 inhibition |
| Lane 7 | Z004 | Scr+cGAMP |
| Lane 8 | Z026 | Scr+cGAMP |
| Lane 9 | Z018 | Scr+cGAMP |
| Lane 10 | Z004 | Scr+ULK1 inhibition+cGAMP |
| Lane 11 | Z026 | Scr+ULK1 inhibition+cGAMP |
| Lane 12 | Z018 | Scr+ULK1 inhibition+cGAMP |
| Lane 13 | Z004 | ATG5 KO |
| Lane 14 | Z026 | ATG5 KO |
| Lane 15 | Z018 | ATG5 KO |
| Lane 16 | Z004 | ATG5KO+ cGAMP |
| Lane 17 | Z026 | ATG5KO+ cGAMP |
| Lane 18 | Z018 | ATG5KO+ cGAMP |
| Lane 19 | Z004 | ATG5 KO+H-151 |
| Lane 20 | Z026 | ATG5 KO+H-151 |
| Lane 21 | Z018 | ATG5 KO+H-151 |
| Lane 22 | Z004 | Scr |
| Lane 23 | Z026 | Scr |
| Lane 24 | Z018 | Scr |
| Lane 25 | Z022 | Scr |
GAPDH
37-

## Slide 4
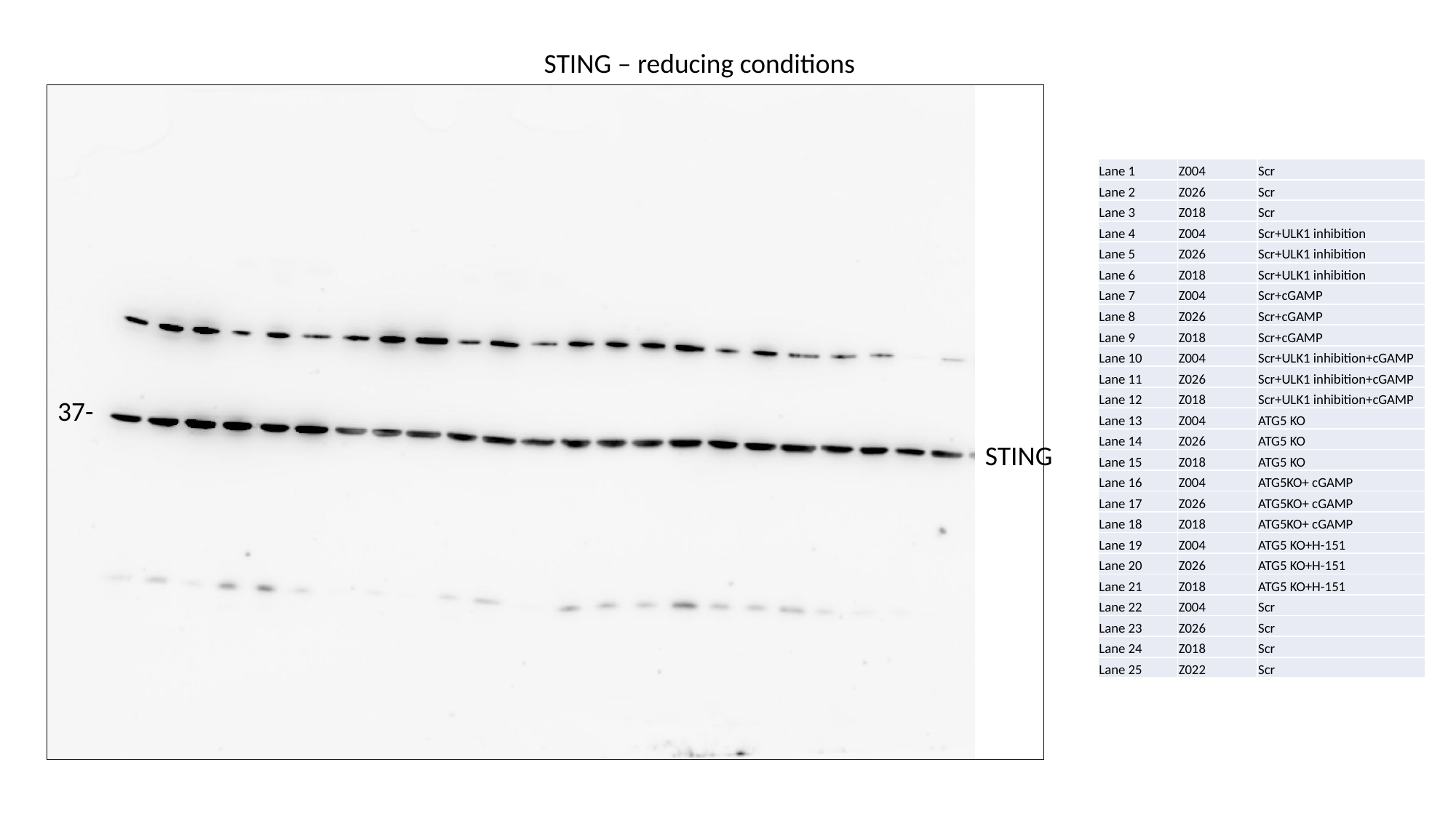

STING – reducing conditions
| Lane 1 | Z004 | Scr |
| --- | --- | --- |
| Lane 2 | Z026 | Scr |
| Lane 3 | Z018 | Scr |
| Lane 4 | Z004 | Scr+ULK1 inhibition |
| Lane 5 | Z026 | Scr+ULK1 inhibition |
| Lane 6 | Z018 | Scr+ULK1 inhibition |
| Lane 7 | Z004 | Scr+cGAMP |
| Lane 8 | Z026 | Scr+cGAMP |
| Lane 9 | Z018 | Scr+cGAMP |
| Lane 10 | Z004 | Scr+ULK1 inhibition+cGAMP |
| Lane 11 | Z026 | Scr+ULK1 inhibition+cGAMP |
| Lane 12 | Z018 | Scr+ULK1 inhibition+cGAMP |
| Lane 13 | Z004 | ATG5 KO |
| Lane 14 | Z026 | ATG5 KO |
| Lane 15 | Z018 | ATG5 KO |
| Lane 16 | Z004 | ATG5KO+ cGAMP |
| Lane 17 | Z026 | ATG5KO+ cGAMP |
| Lane 18 | Z018 | ATG5KO+ cGAMP |
| Lane 19 | Z004 | ATG5 KO+H-151 |
| Lane 20 | Z026 | ATG5 KO+H-151 |
| Lane 21 | Z018 | ATG5 KO+H-151 |
| Lane 22 | Z004 | Scr |
| Lane 23 | Z026 | Scr |
| Lane 24 | Z018 | Scr |
| Lane 25 | Z022 | Scr |
37-
STING
