# Supplementary material for: Tumor endothelial cell autophagy is a key vascular‐immune checkpoint in melanoma
Source: EMBO Mol Med. 2023 Nov 27;15(12):e18028. doi: 10.15252/emmm.202318028 (PMC10701618; doi:10.15252/emmm.202318028)
Supplement: Supplementary file 9 — Source Data for Figure 4 [file EMMM-15-e18028-s004.zip › figure_4_raw_data/4h/4h_uncut_blots.pptx]

## Slide 1
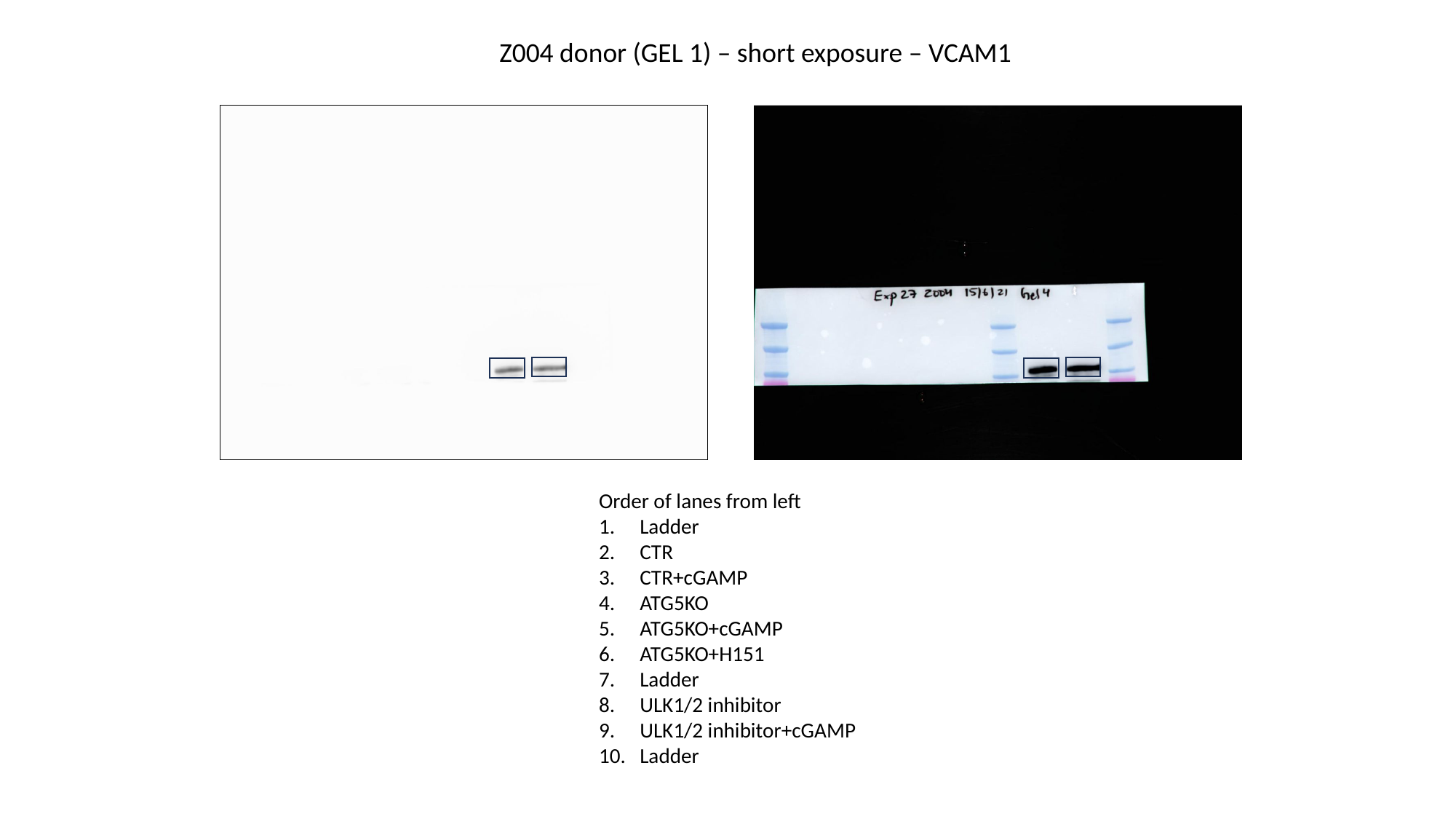

Z004 donor (GEL 1) – short exposure – VCAM1
Order of lanes from left
Ladder
CTR
CTR+cGAMP
ATG5KO
ATG5KO+cGAMP
ATG5KO+H151
Ladder
ULK1/2 inhibitor
ULK1/2 inhibitor+cGAMP
Ladder

## Slide 2
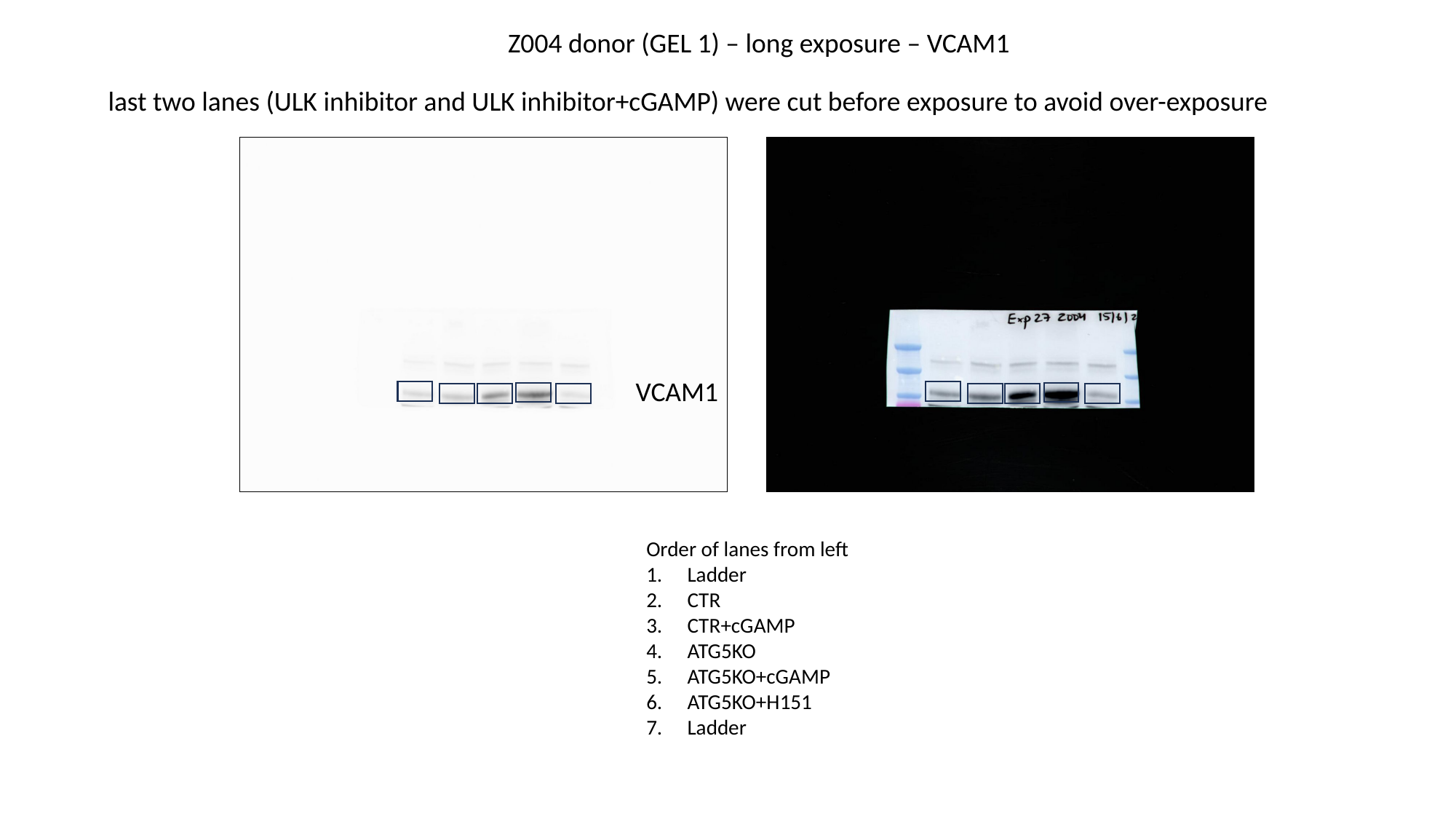

Z004 donor (GEL 1) – long exposure – VCAM1
last two lanes (ULK inhibitor and ULK inhibitor+cGAMP) were cut before exposure to avoid over-exposure
VCAM1
Order of lanes from left
Ladder
CTR
CTR+cGAMP
ATG5KO
ATG5KO+cGAMP
ATG5KO+H151
Ladder

## Slide 3
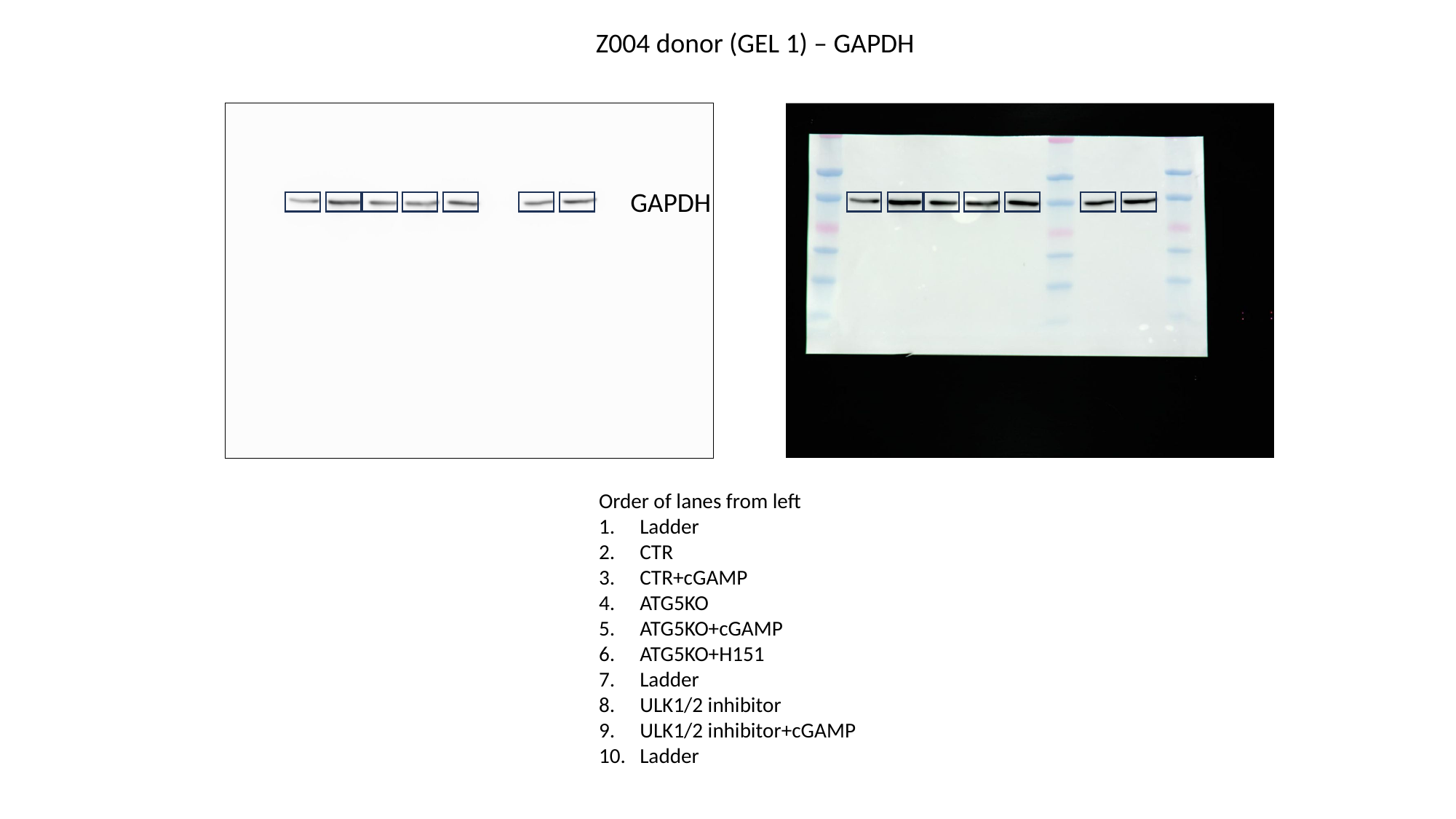

Z004 donor (GEL 1) – GAPDH
GAPDH
Order of lanes from left
Ladder
CTR
CTR+cGAMP
ATG5KO
ATG5KO+cGAMP
ATG5KO+H151
Ladder
ULK1/2 inhibitor
ULK1/2 inhibitor+cGAMP
Ladder

## Slide 4
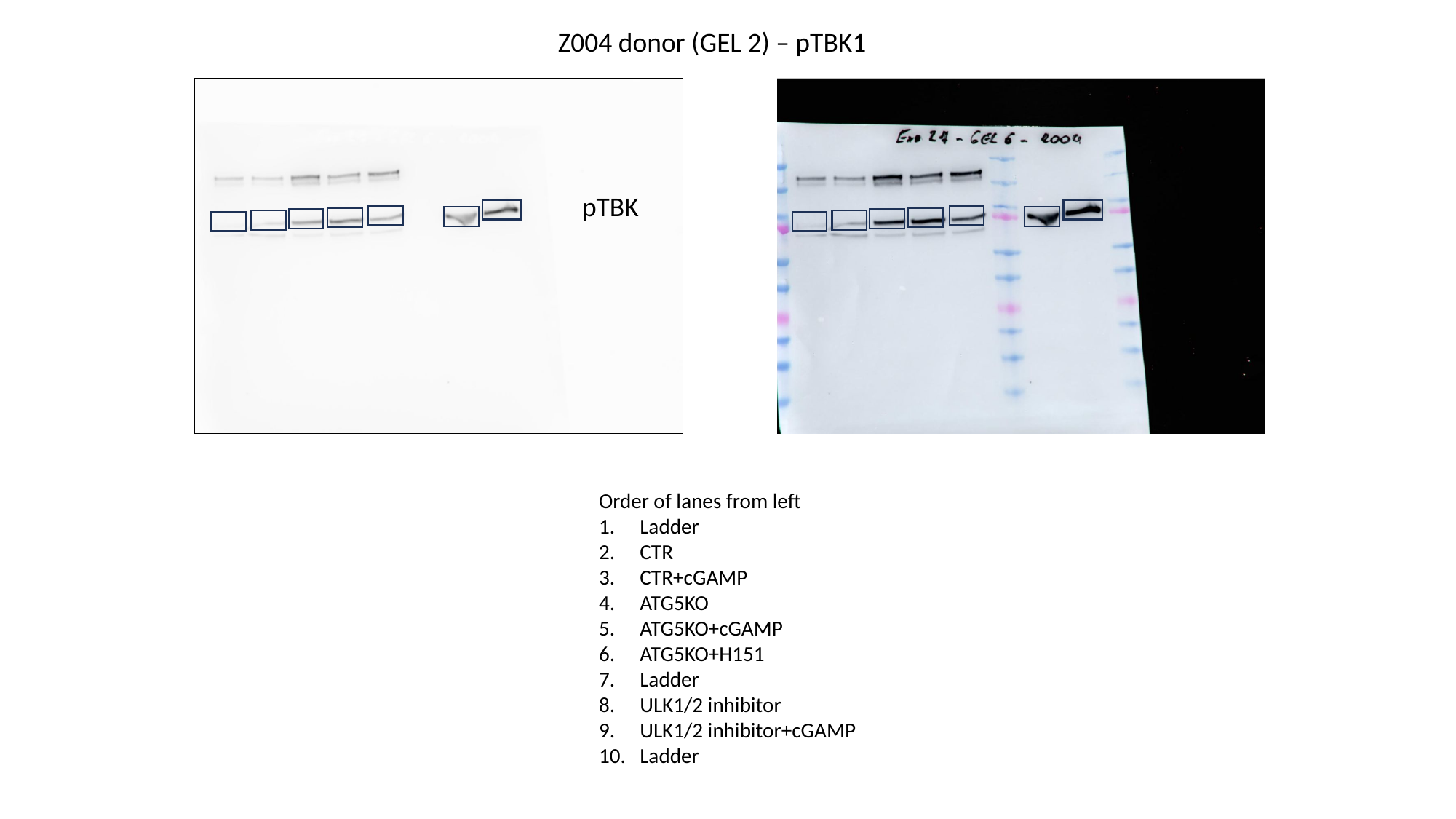

Z004 donor (GEL 2) – pTBK1
pTBK
Order of lanes from left
Ladder
CTR
CTR+cGAMP
ATG5KO
ATG5KO+cGAMP
ATG5KO+H151
Ladder
ULK1/2 inhibitor
ULK1/2 inhibitor+cGAMP
Ladder

## Slide 5
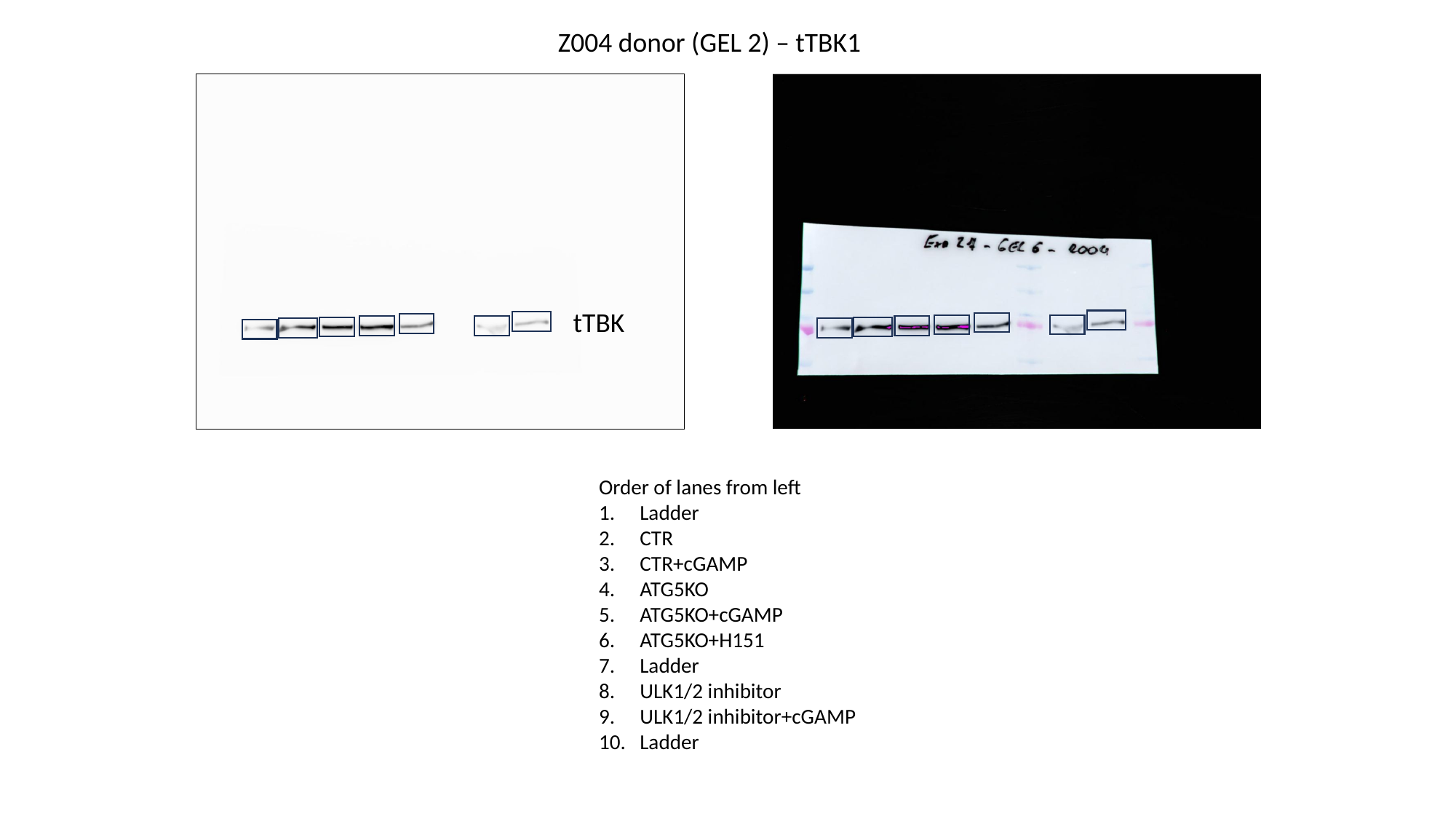

Z004 donor (GEL 2) – tTBK1
tTBK
Order of lanes from left
Ladder
CTR
CTR+cGAMP
ATG5KO
ATG5KO+cGAMP
ATG5KO+H151
Ladder
ULK1/2 inhibitor
ULK1/2 inhibitor+cGAMP
Ladder

## Slide 6
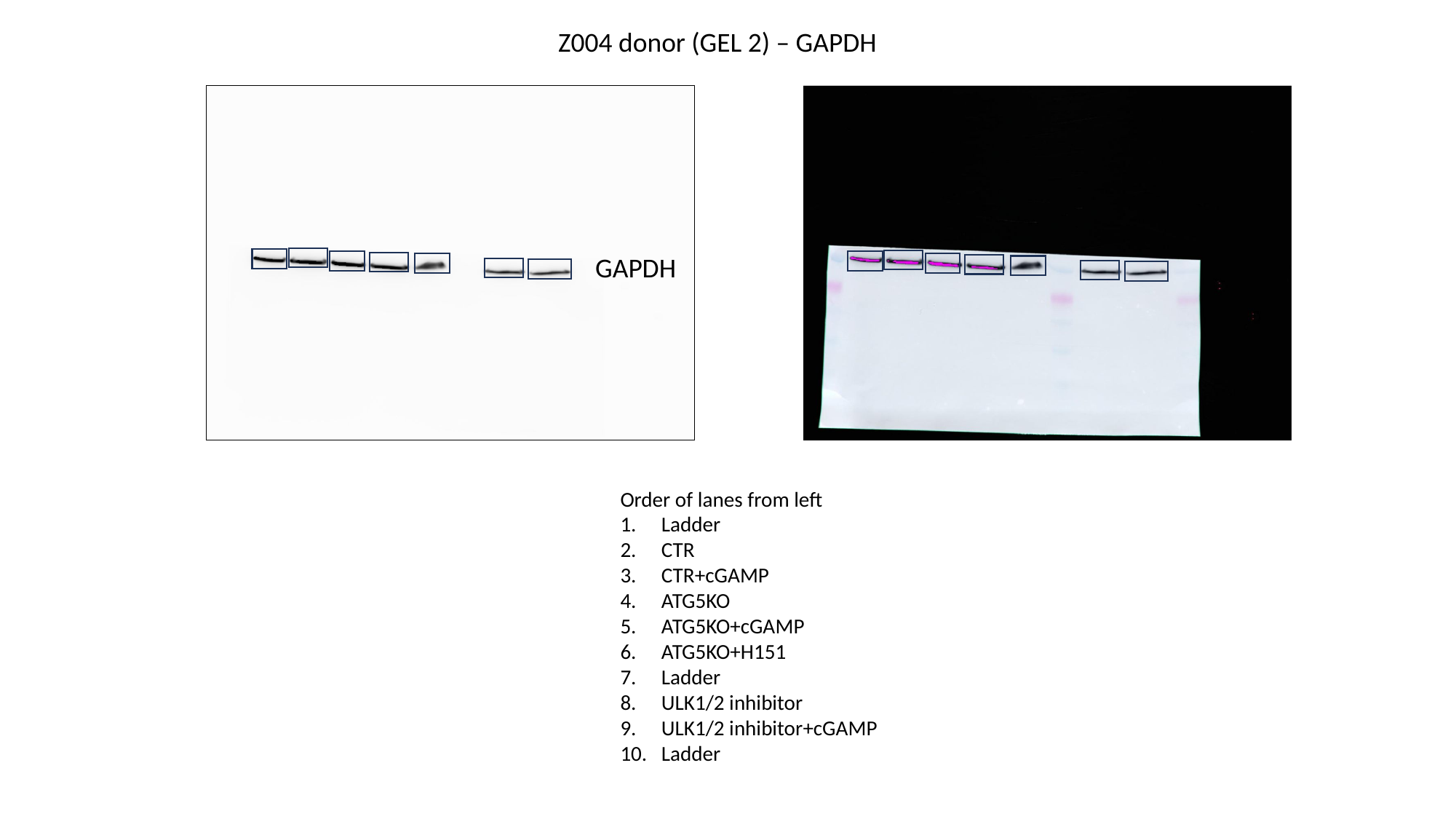

Z004 donor (GEL 2) – GAPDH
GAPDH
Order of lanes from left
Ladder
CTR
CTR+cGAMP
ATG5KO
ATG5KO+cGAMP
ATG5KO+H151
Ladder
ULK1/2 inhibitor
ULK1/2 inhibitor+cGAMP
Ladder

## Slide 7
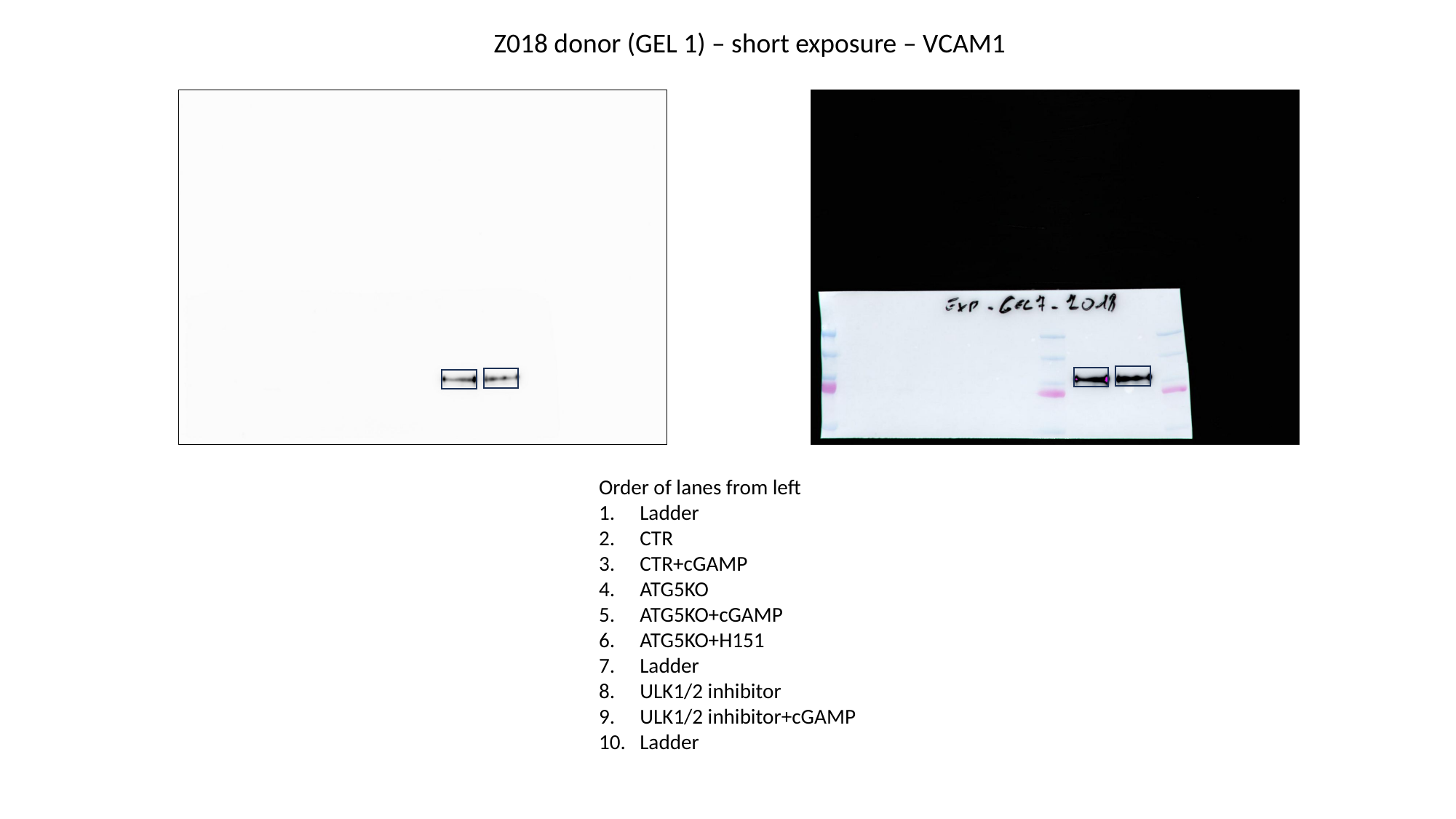

Z018 donor (GEL 1) – short exposure – VCAM1
Order of lanes from left
Ladder
CTR
CTR+cGAMP
ATG5KO
ATG5KO+cGAMP
ATG5KO+H151
Ladder
ULK1/2 inhibitor
ULK1/2 inhibitor+cGAMP
Ladder

## Slide 8
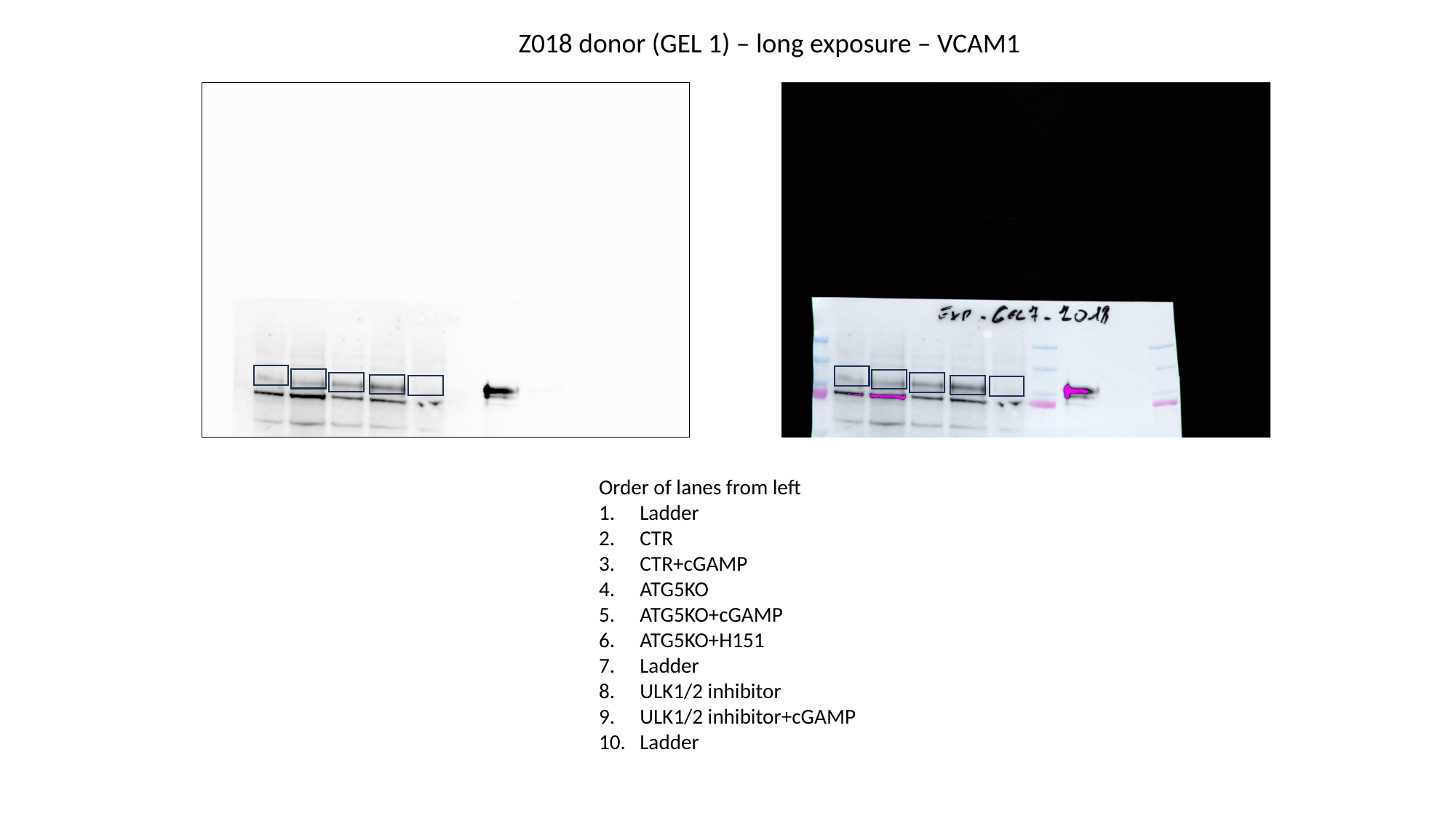

Z018 donor (GEL 1) – long exposure – VCAM1
Order of lanes from left
Ladder
CTR
CTR+cGAMP
ATG5KO
ATG5KO+cGAMP
ATG5KO+H151
Ladder
ULK1/2 inhibitor
ULK1/2 inhibitor+cGAMP
Ladder

## Slide 9
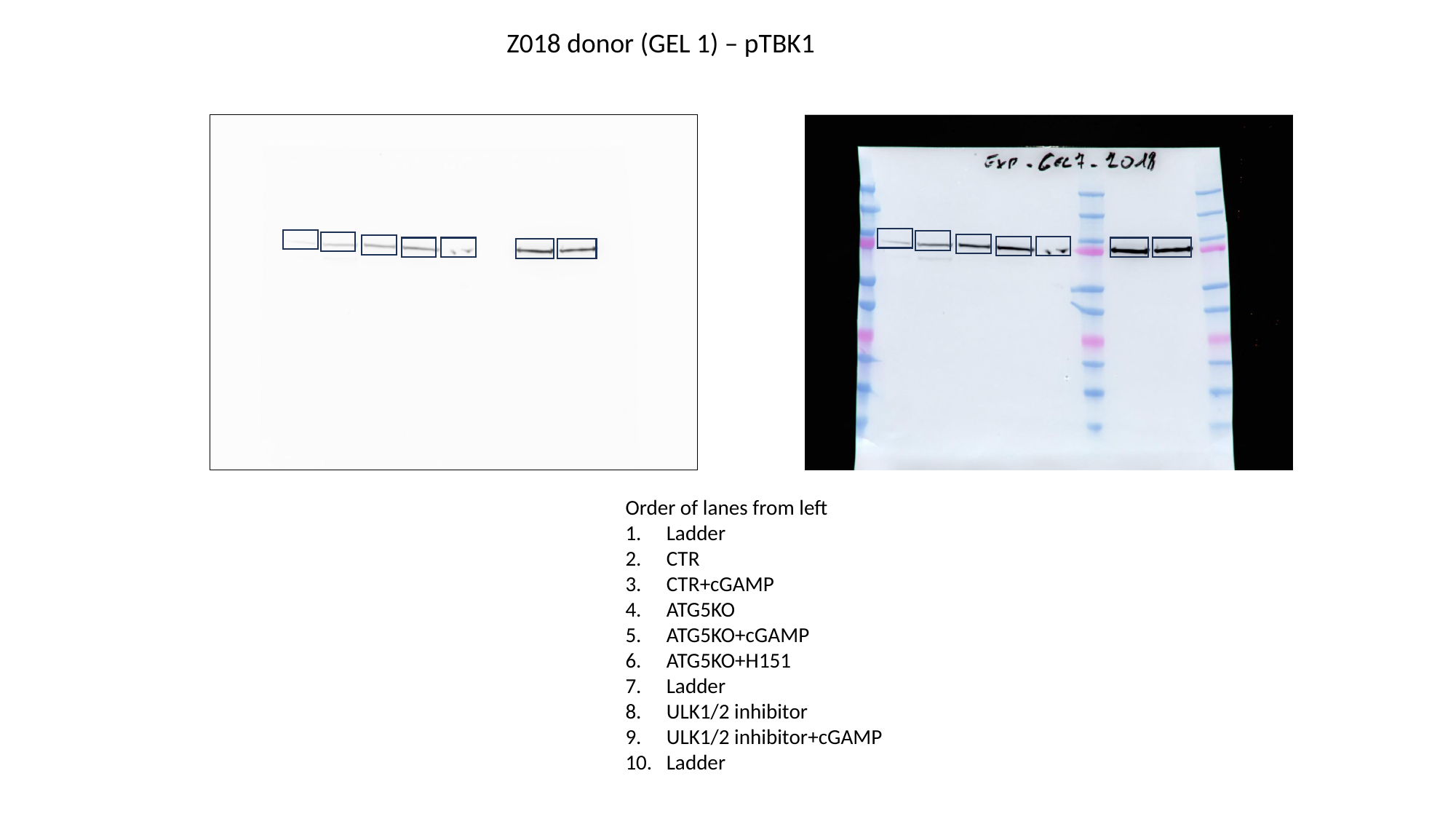

Z018 donor (GEL 1) – pTBK1
Order of lanes from left
Ladder
CTR
CTR+cGAMP
ATG5KO
ATG5KO+cGAMP
ATG5KO+H151
Ladder
ULK1/2 inhibitor
ULK1/2 inhibitor+cGAMP
Ladder

## Slide 10
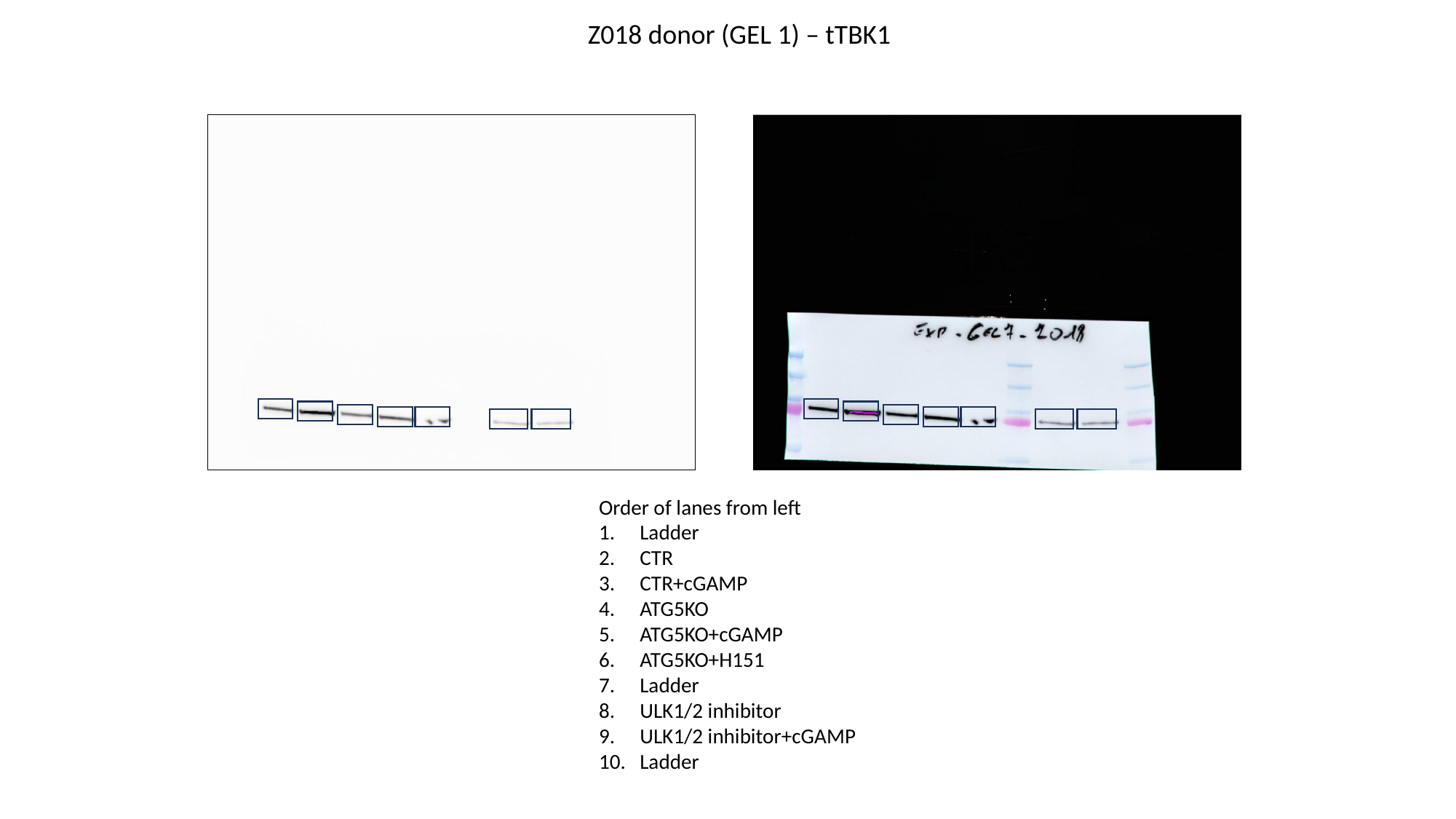

Z018 donor (GEL 1) – tTBK1
Order of lanes from left
Ladder
CTR
CTR+cGAMP
ATG5KO
ATG5KO+cGAMP
ATG5KO+H151
Ladder
ULK1/2 inhibitor
ULK1/2 inhibitor+cGAMP
Ladder

## Slide 11
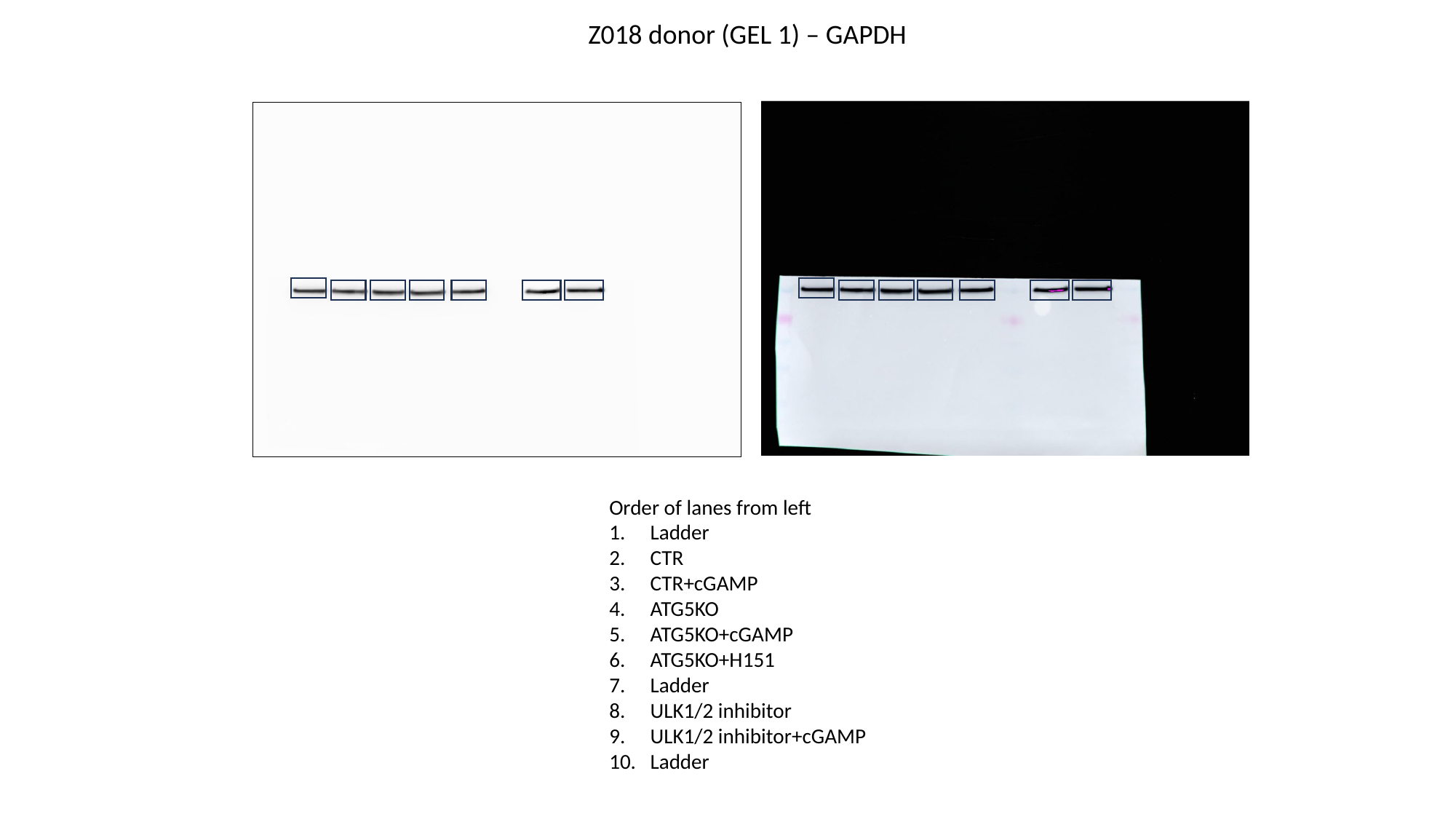

Z018 donor (GEL 1) – GAPDH
Order of lanes from left
Ladder
CTR
CTR+cGAMP
ATG5KO
ATG5KO+cGAMP
ATG5KO+H151
Ladder
ULK1/2 inhibitor
ULK1/2 inhibitor+cGAMP
Ladder

## Slide 12
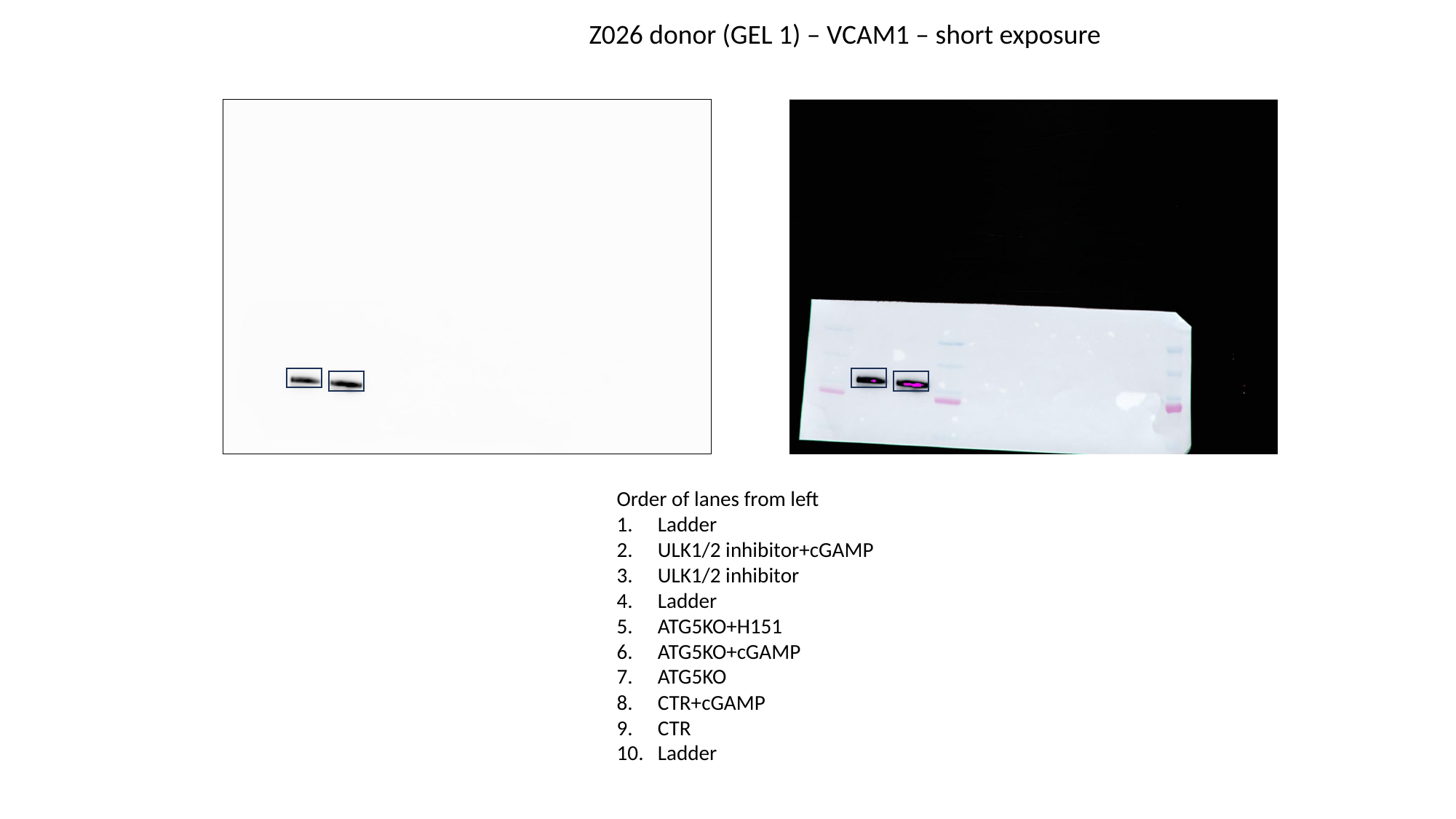

Z026 donor (GEL 1) – VCAM1 – short exposure
Order of lanes from left
Ladder
ULK1/2 inhibitor+cGAMP
ULK1/2 inhibitor
Ladder
ATG5KO+H151
ATG5KO+cGAMP
ATG5KO
CTR+cGAMP
CTR
Ladder

## Slide 13
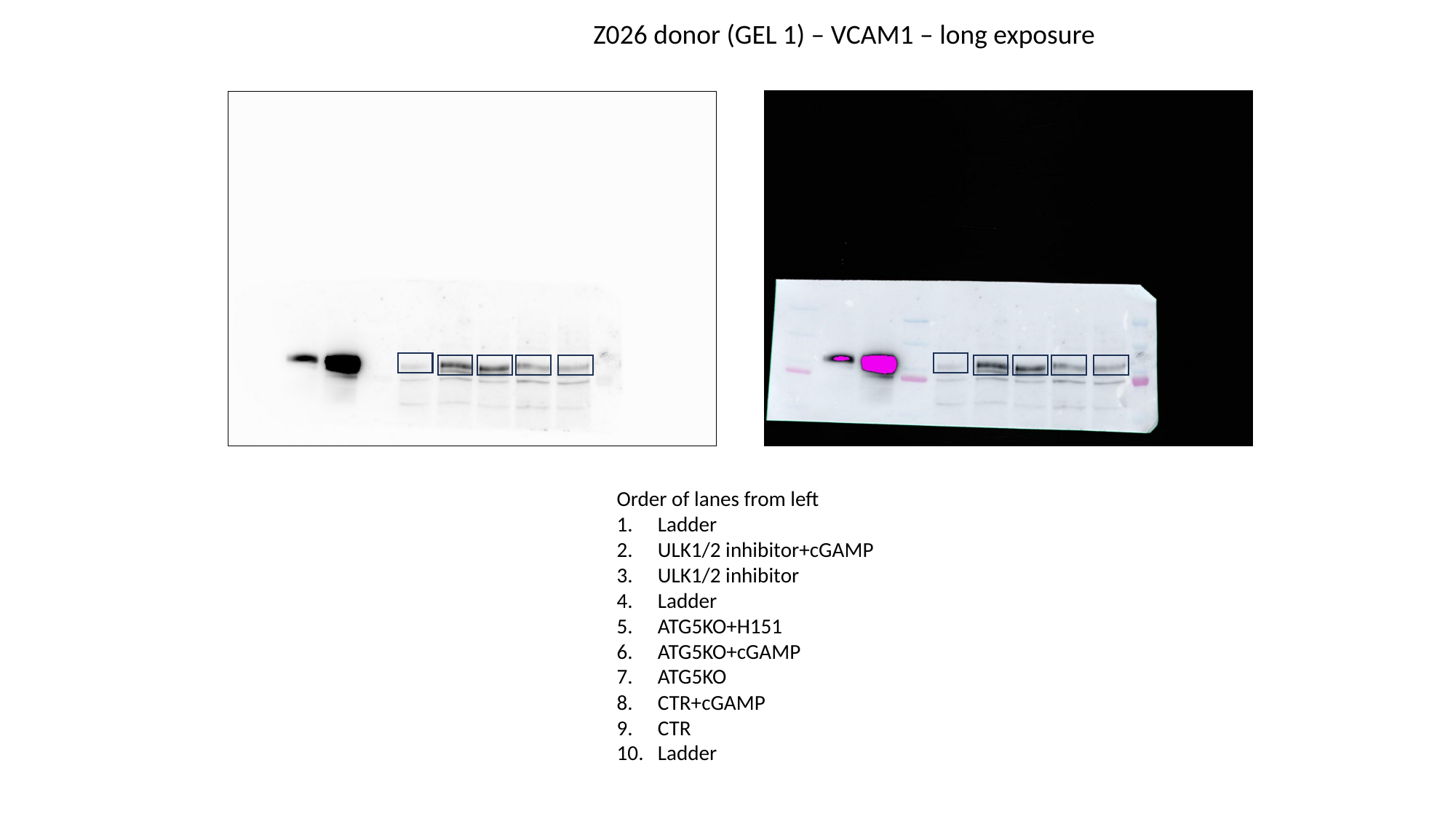

Z026 donor (GEL 1) – VCAM1 – long exposure
Order of lanes from left
Ladder
ULK1/2 inhibitor+cGAMP
ULK1/2 inhibitor
Ladder
ATG5KO+H151
ATG5KO+cGAMP
ATG5KO
CTR+cGAMP
CTR
Ladder

## Slide 14
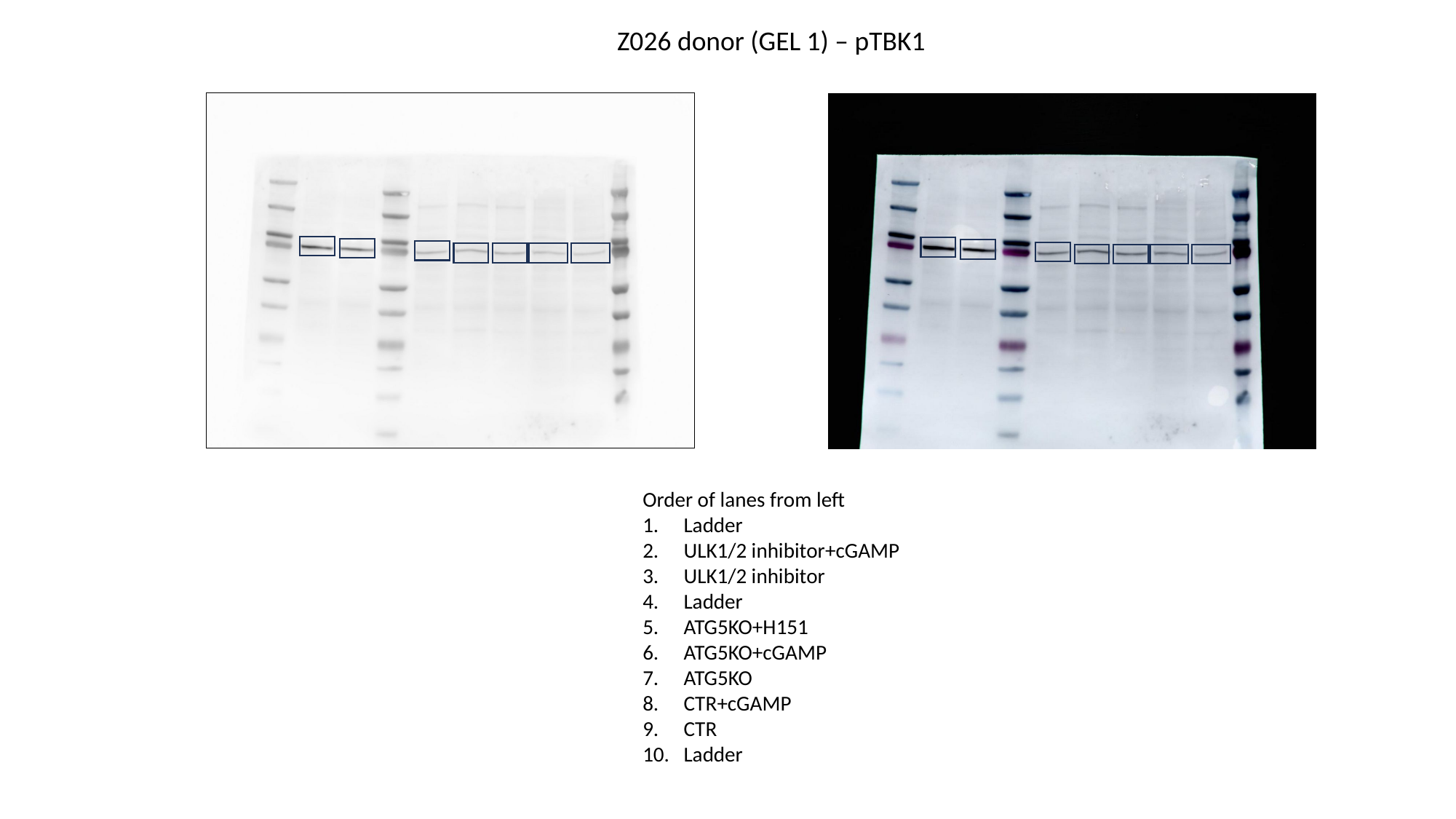

Z026 donor (GEL 1) – pTBK1
Order of lanes from left
Ladder
ULK1/2 inhibitor+cGAMP
ULK1/2 inhibitor
Ladder
ATG5KO+H151
ATG5KO+cGAMP
ATG5KO
CTR+cGAMP
CTR
Ladder

## Slide 15
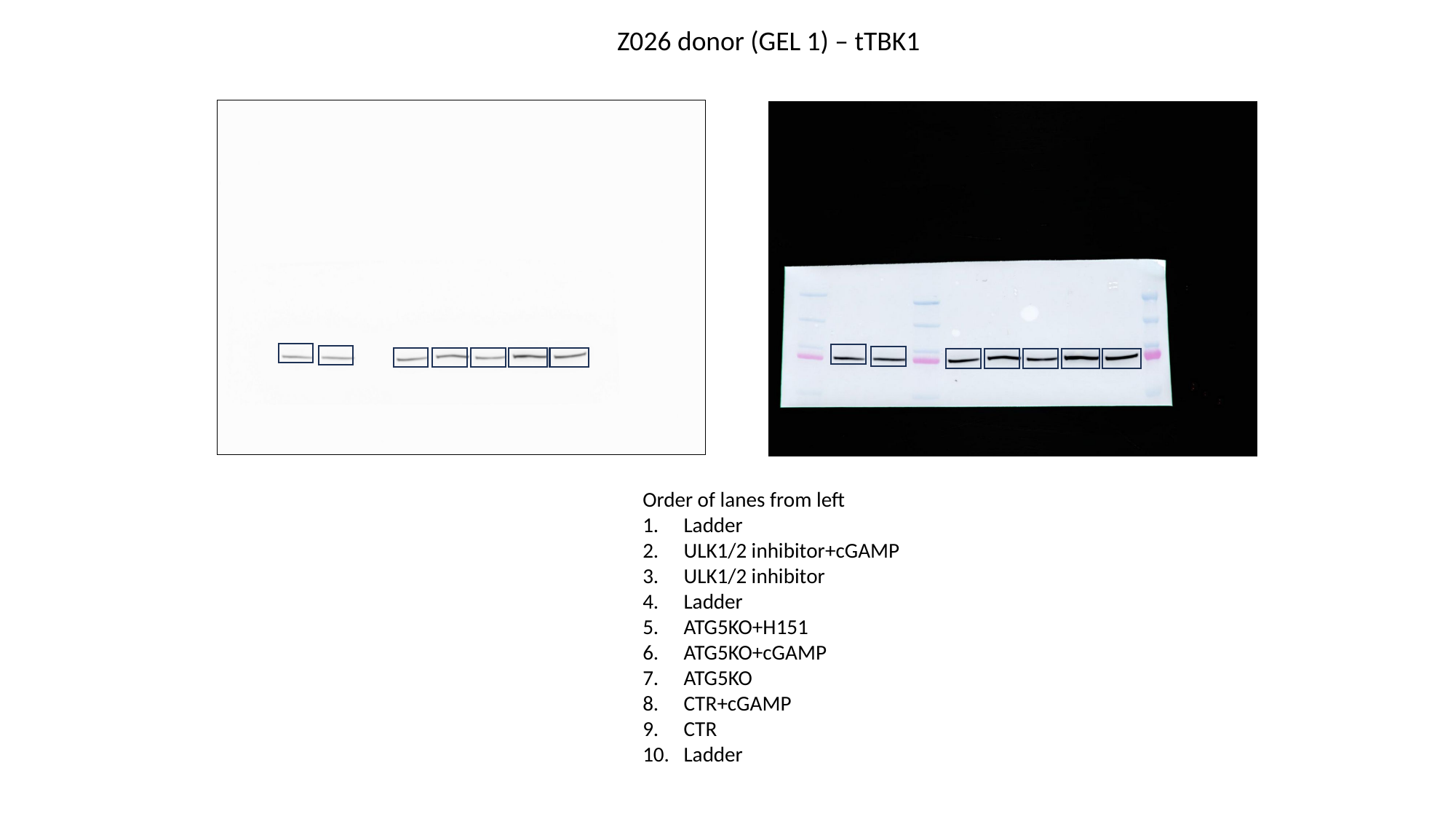

Z026 donor (GEL 1) – tTBK1
Order of lanes from left
Ladder
ULK1/2 inhibitor+cGAMP
ULK1/2 inhibitor
Ladder
ATG5KO+H151
ATG5KO+cGAMP
ATG5KO
CTR+cGAMP
CTR
Ladder

## Slide 16
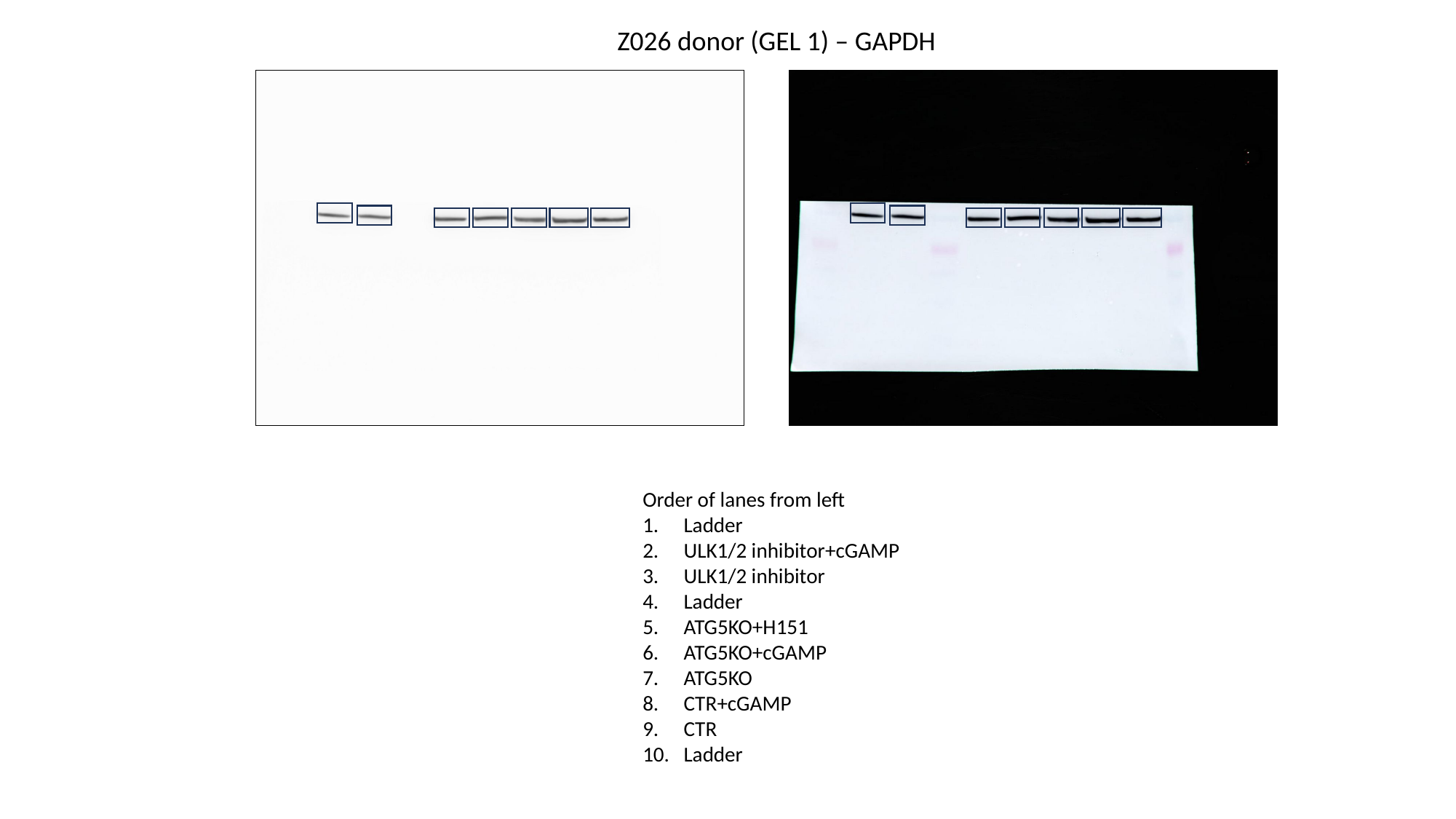

Z026 donor (GEL 1) – GAPDH
Order of lanes from left
Ladder
ULK1/2 inhibitor+cGAMP
ULK1/2 inhibitor
Ladder
ATG5KO+H151
ATG5KO+cGAMP
ATG5KO
CTR+cGAMP
CTR
Ladder
