# Supplementary material for: Tumor endothelial cell autophagy is a key vascular‐immune checkpoint in melanoma
Source: EMBO Mol Med. 2023 Nov 27;15(12):e18028. doi: 10.15252/emmm.202318028 (PMC10701618; doi:10.15252/emmm.202318028)
Supplement: Supplementary file 9 — Source Data for Figure 4 [file EMMM-15-e18028-s004.zip › figure_4_raw_data/4k/figure_4k.pptx]

## Slide 1
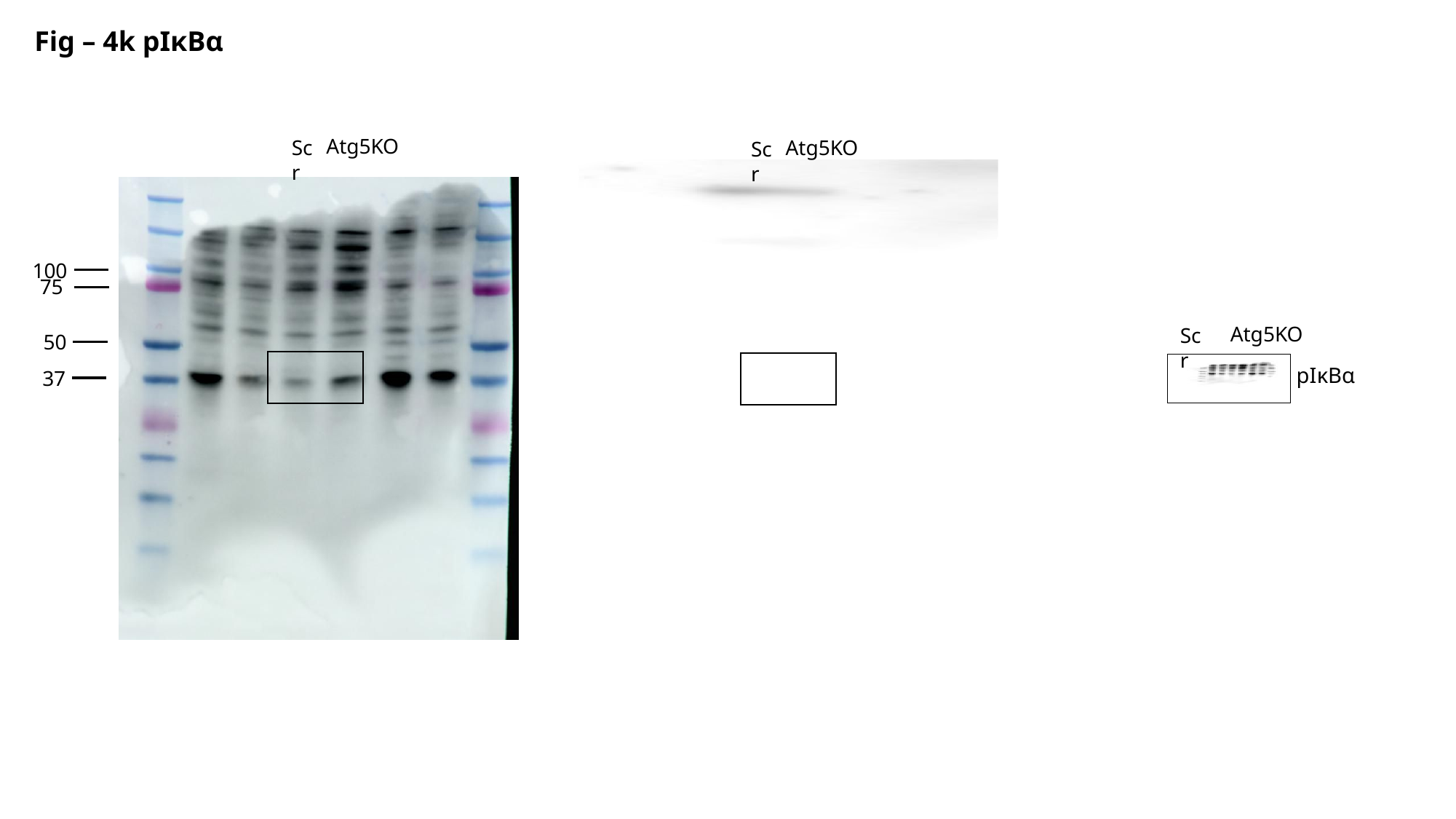

Fig – 4k pIκBα
Atg5KO
Scr
Atg5KO
Scr
100
75
Atg5KO
Scr
50
pIκBα
37

## Slide 2
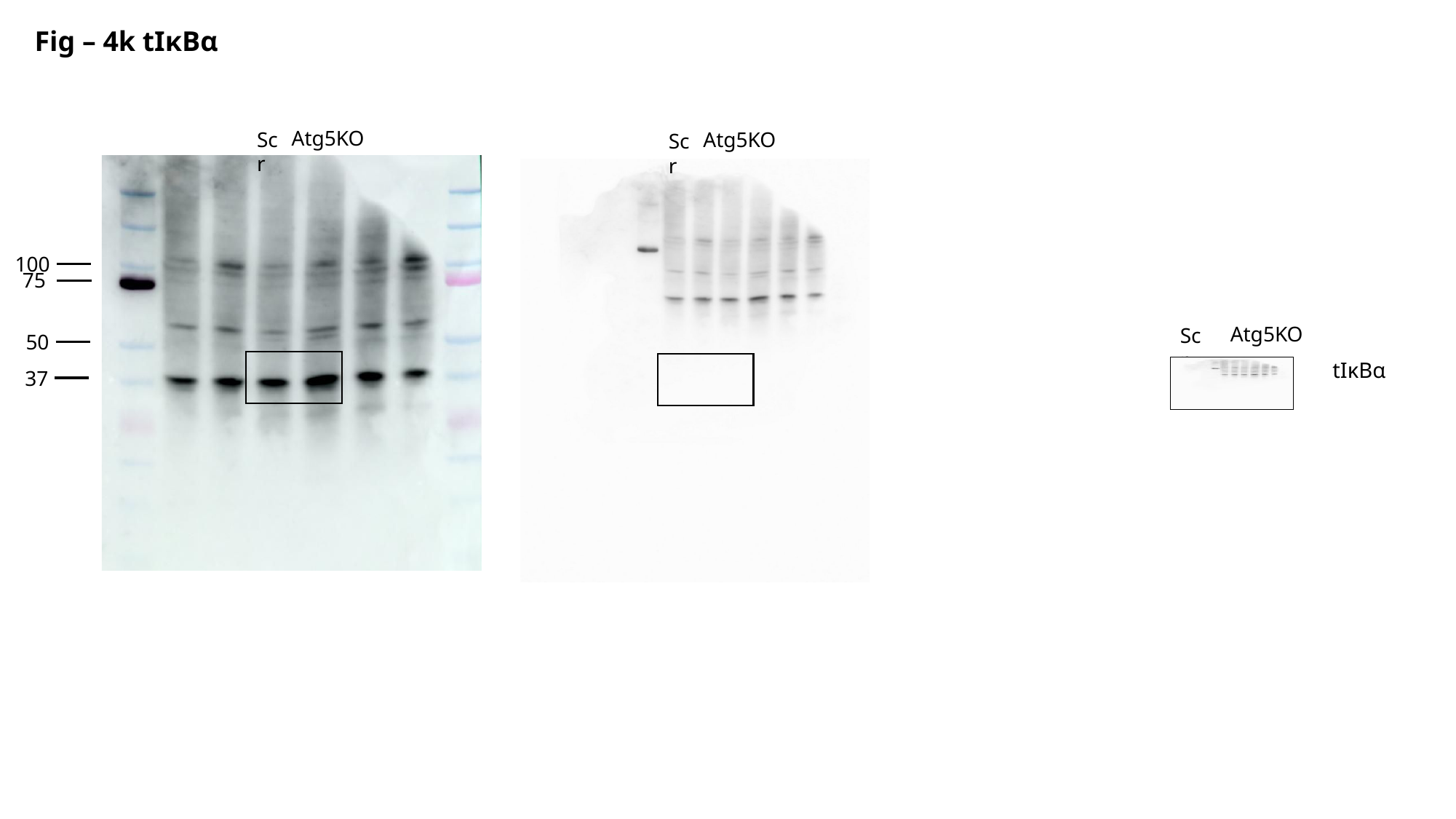

Fig – 4k tIκBα
Atg5KO
Scr
Atg5KO
Scr
100
75
Atg5KO
Scr
50
tIκBα
37

## Slide 3
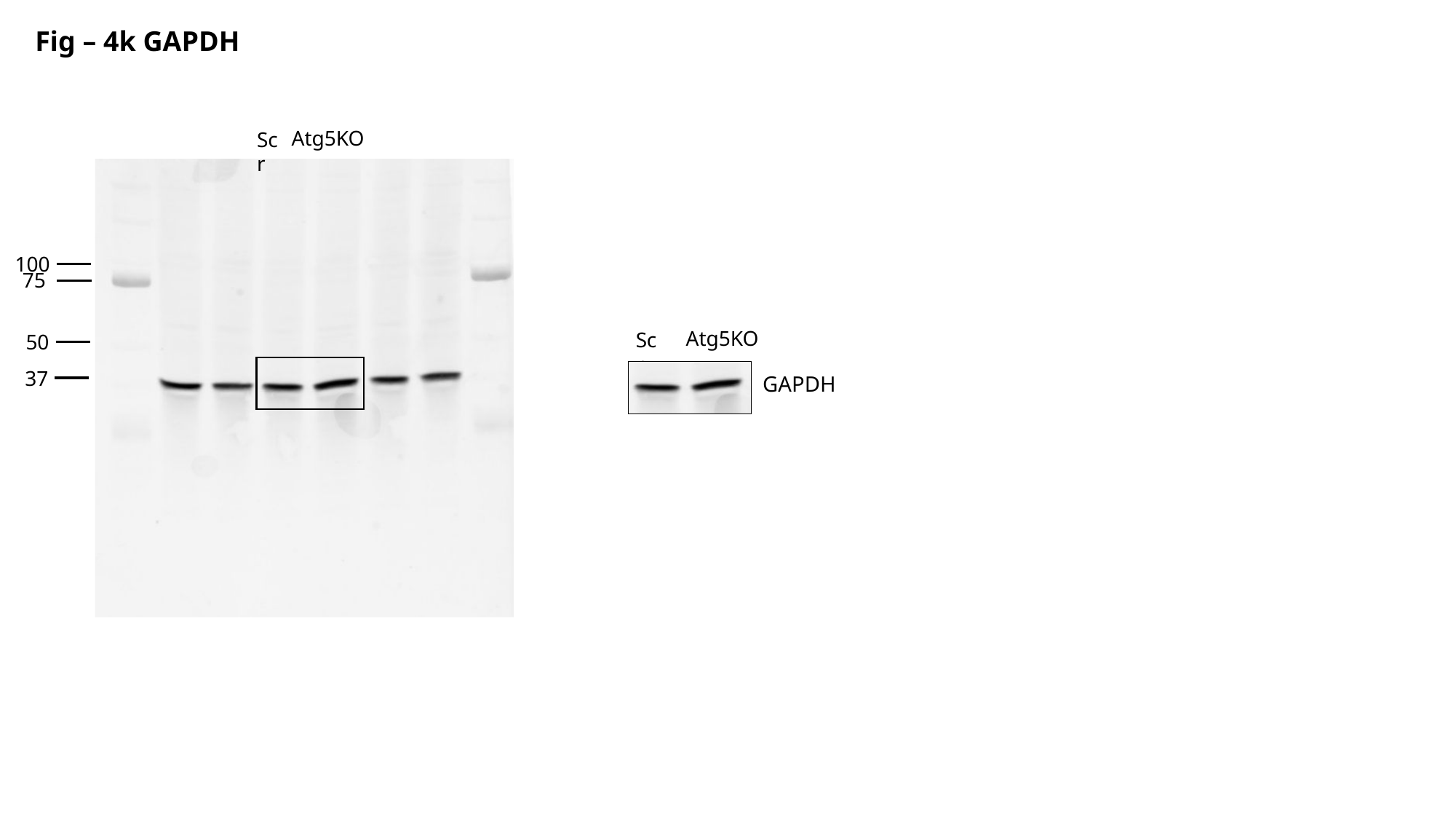

Fig – 4k GAPDH
Atg5KO
Scr
100
75
Atg5KO
Scr
50
37
GAPDH

## Slide 4
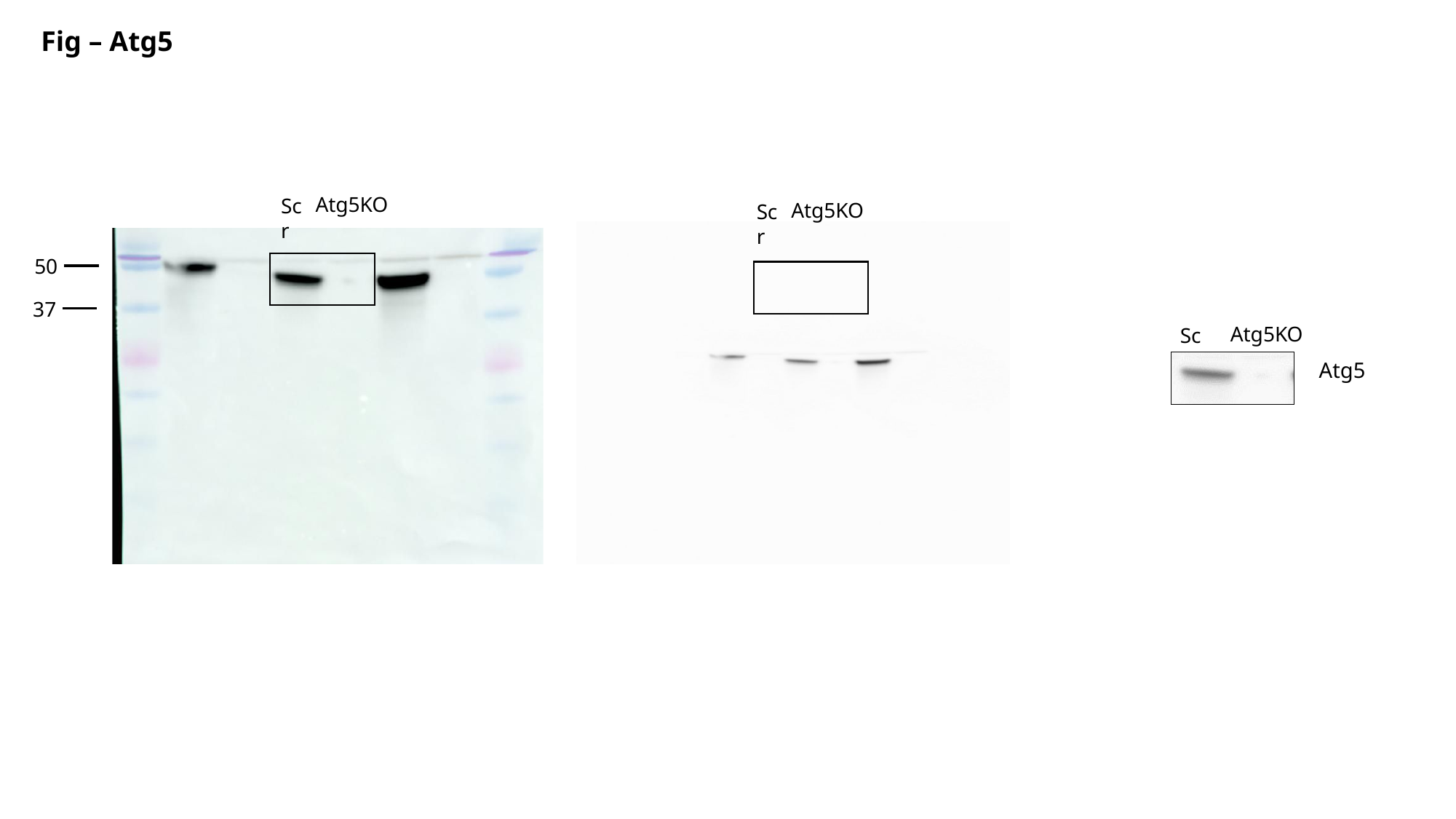

Fig – Atg5
Atg5KO
Scr
Atg5KO
Scr
50
37
Atg5KO
Scr
Atg5

## Slide 5
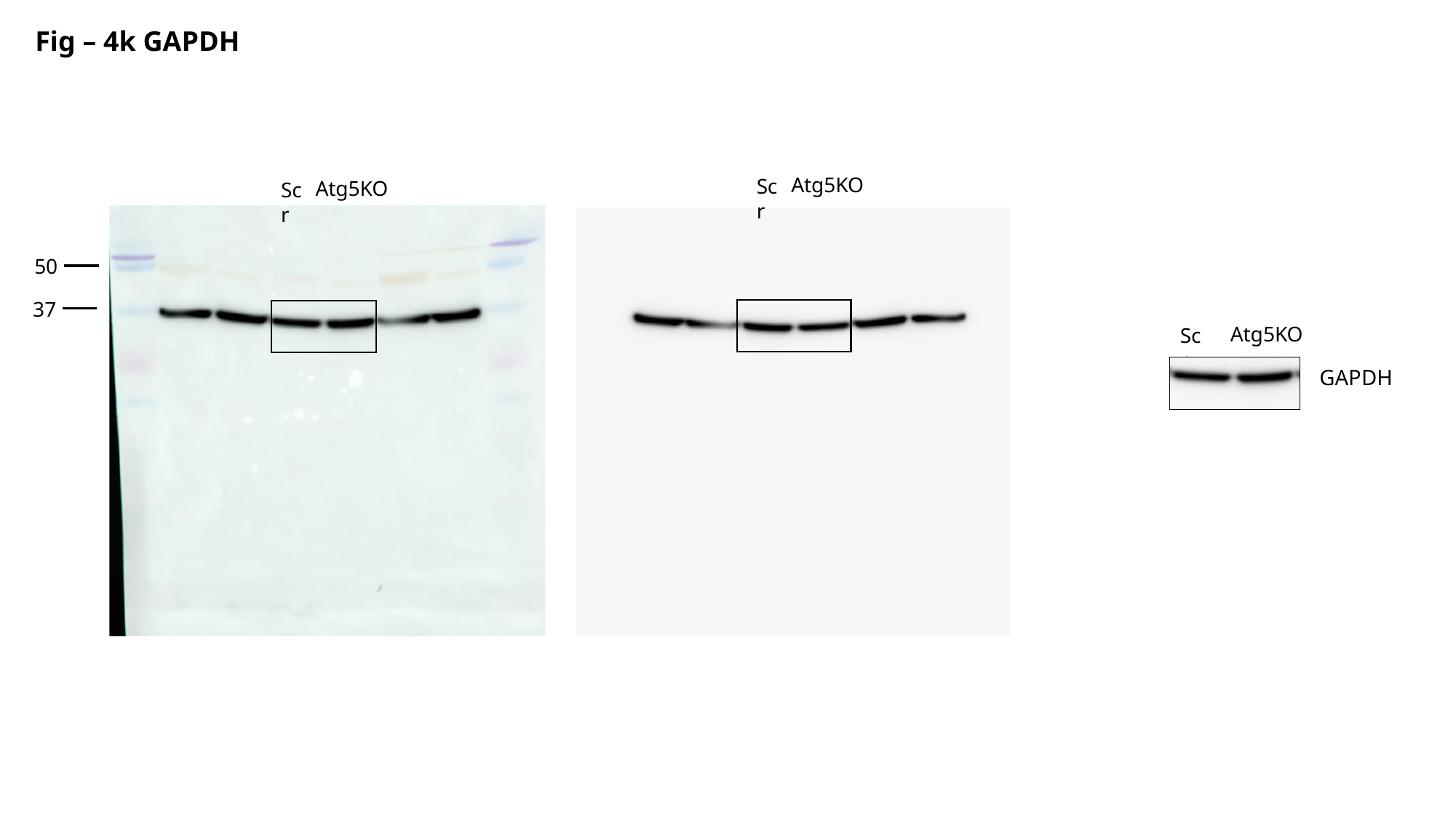

Fig – 4k GAPDH
Atg5KO
Scr
Atg5KO
Scr
50
37
Atg5KO
Scr
GAPDH

## Slide 6
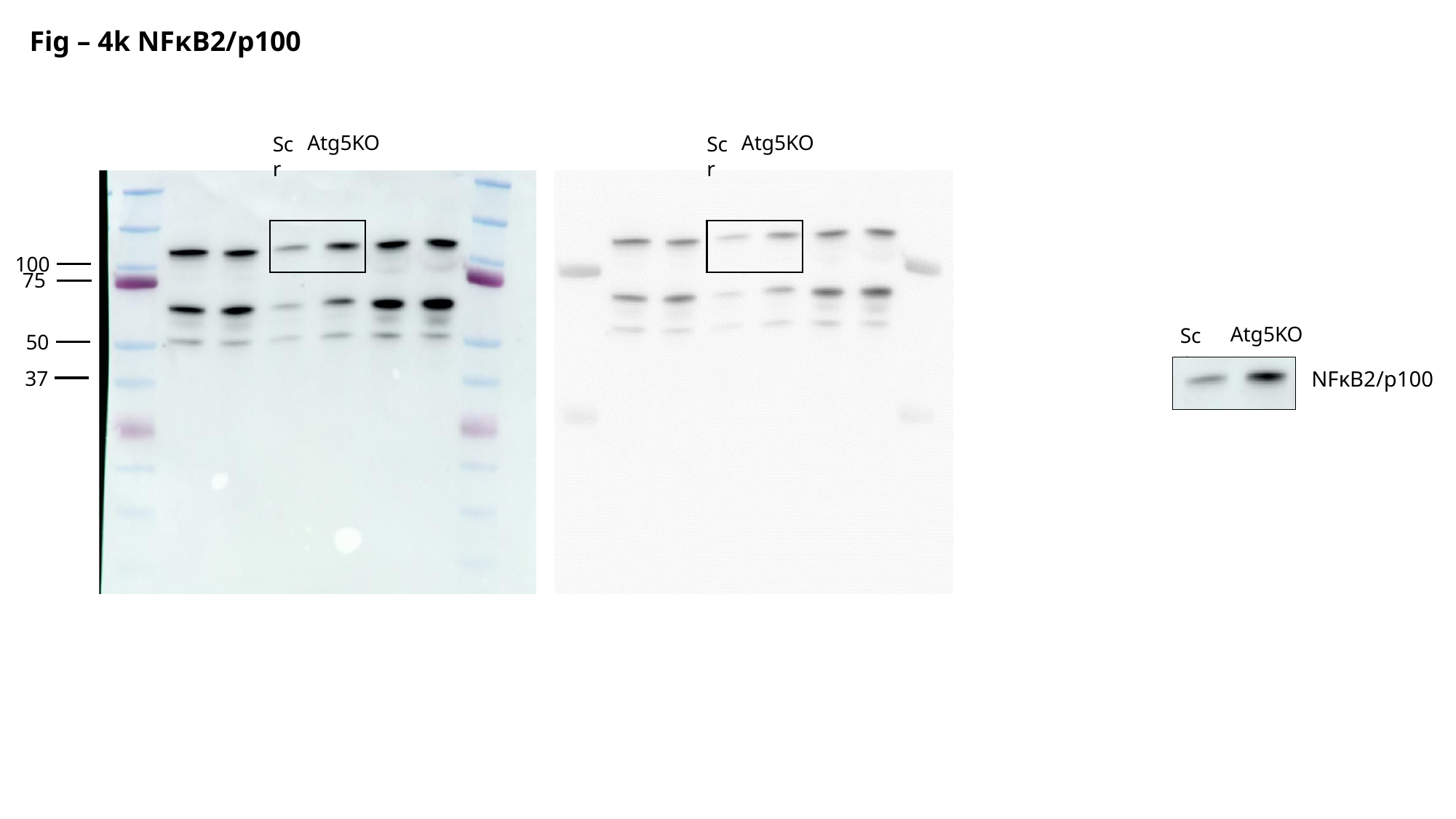

Fig – 4k NFκB2/p100
Atg5KO
Atg5KO
Scr
Scr
100
75
Atg5KO
Scr
50
37
NFκB2/p100

## Slide 7
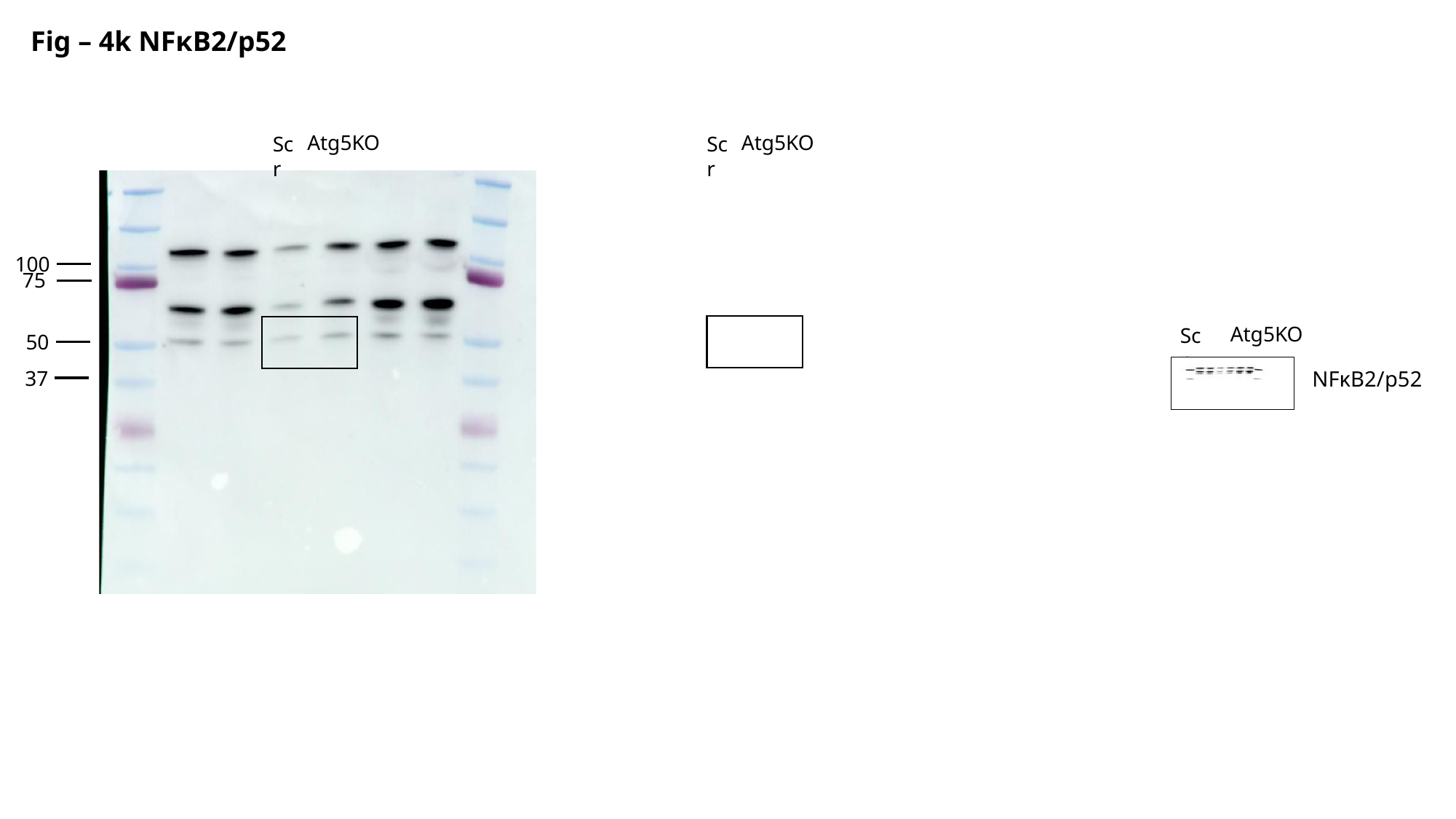

Fig – 4k NFκB2/p52
Atg5KO
Atg5KO
Scr
Scr
100
75
Atg5KO
Scr
50
37
NFκB2/p52

## Slide 8
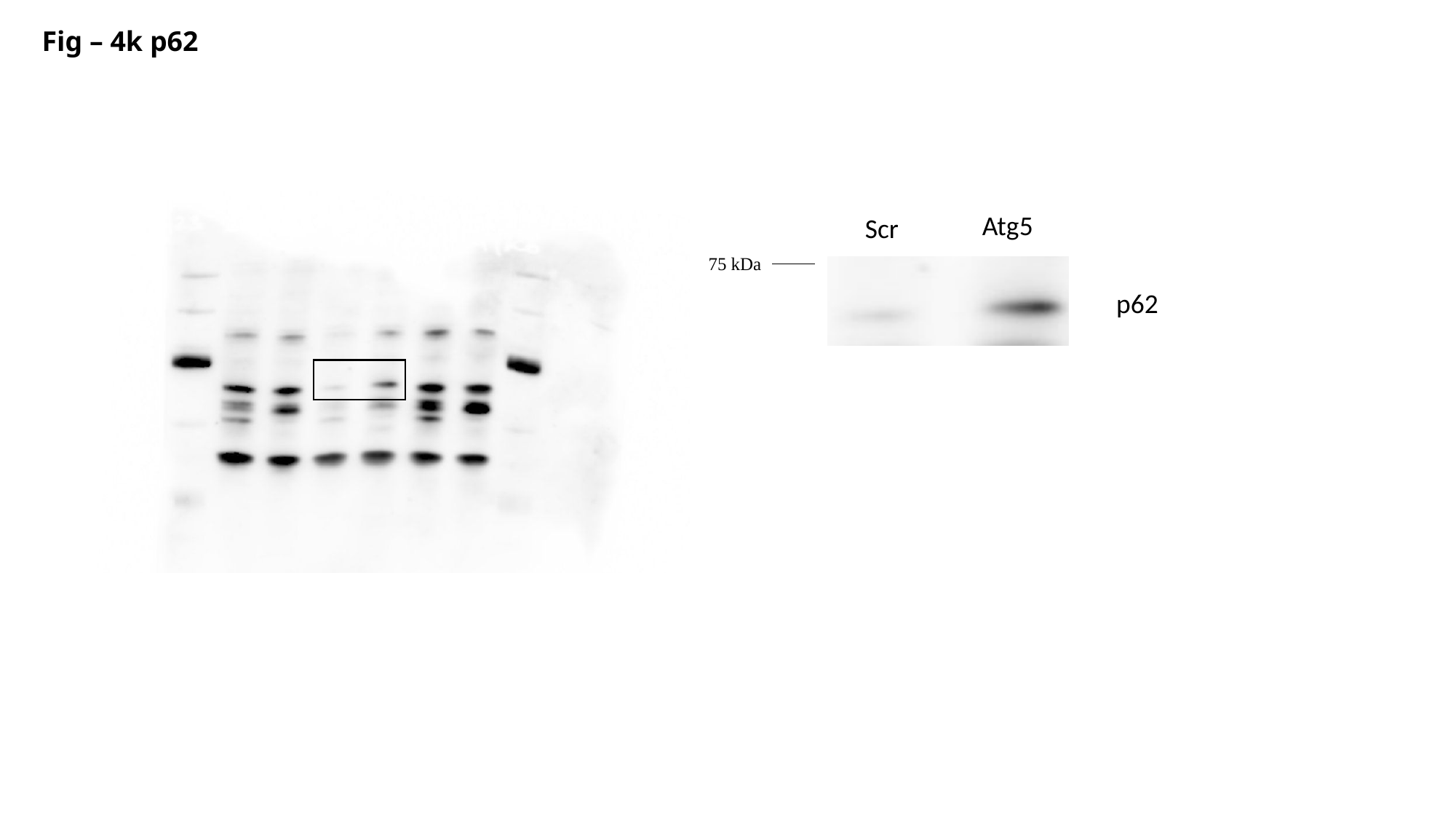

Fig – 4k p62
Atg5
Scr
75 kDa
p62

## Slide 9
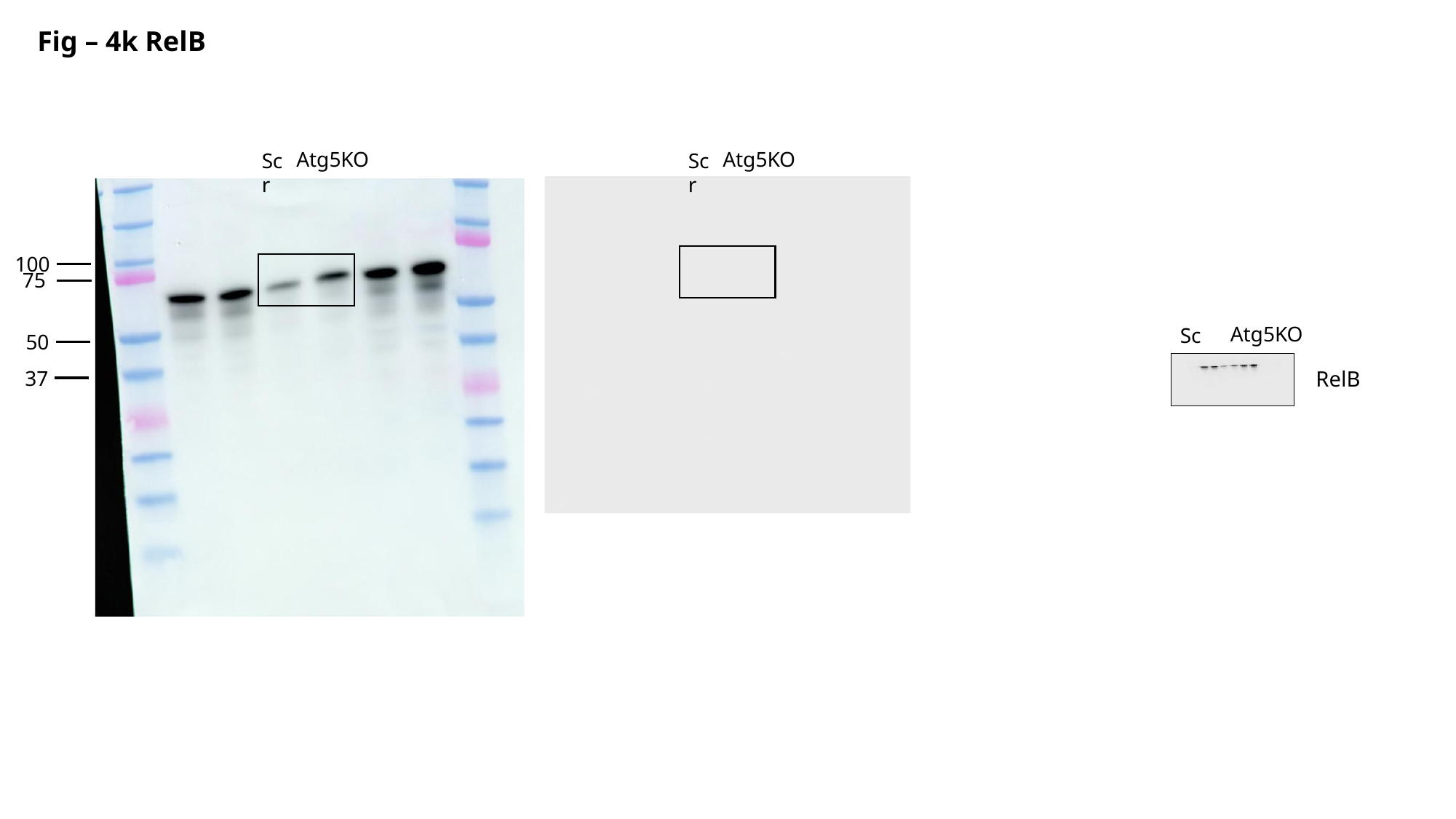

Fig – 4k RelB
Atg5KO
Atg5KO
Scr
Scr
100
75
Atg5KO
Scr
50
37
RelB

## Slide 10
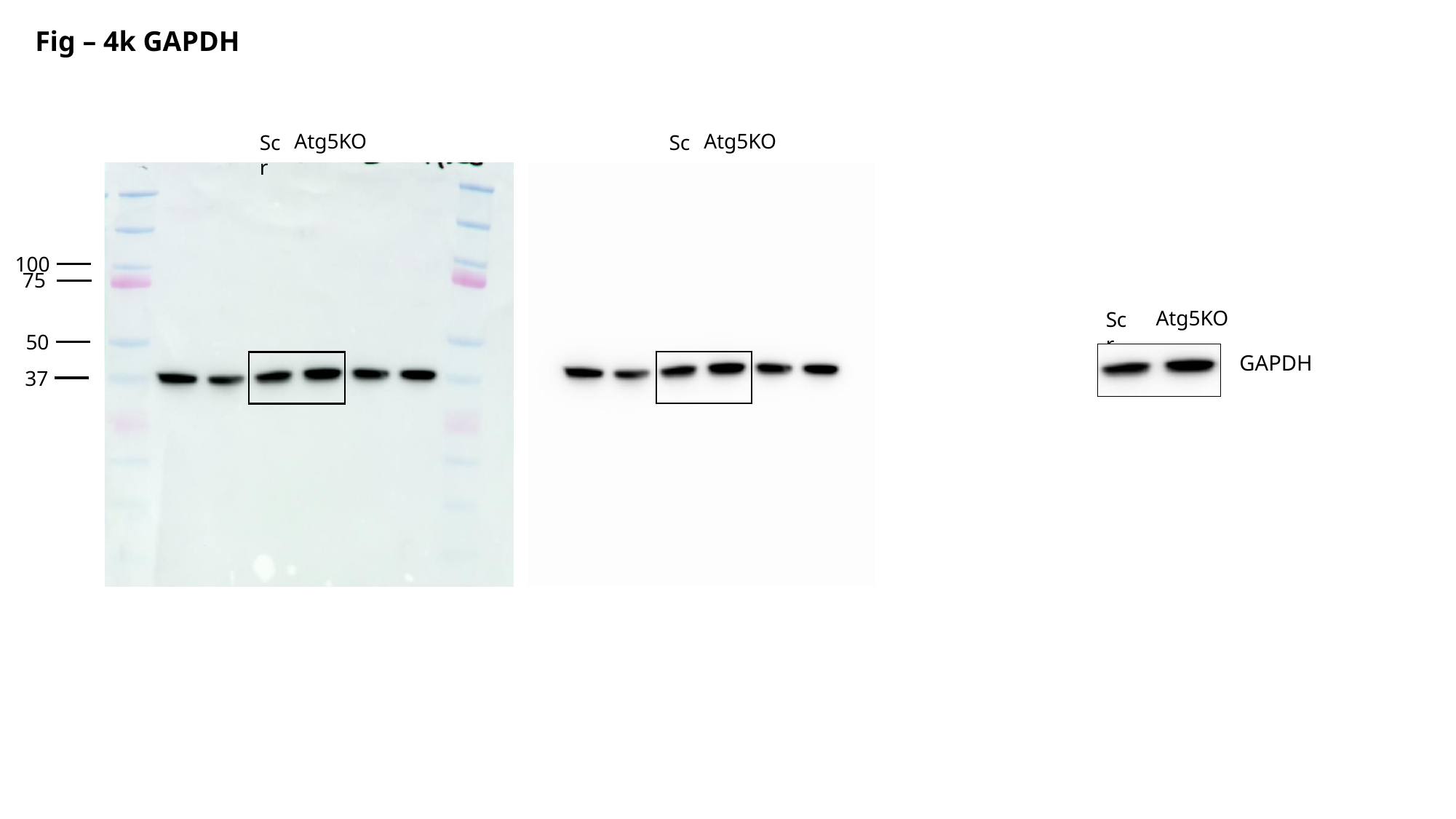

Fig – 4k GAPDH
Atg5KO
Atg5KO
Scr
Scr
100
75
Atg5KO
Scr
50
GAPDH
37
