# Supplementary material for: Tumor endothelial cell autophagy is a key vascular‐immune checkpoint in melanoma
Source: EMBO Mol Med. 2023 Nov 27;15(12):e18028. doi: 10.15252/emmm.202318028 (PMC10701618; doi:10.15252/emmm.202318028)
Supplement: Supplementary file 9 — Source Data for Figure 4 [file EMMM-15-e18028-s004.zip › figure_4_raw_data/4l/figure_4l.pptx]

## Slide 1
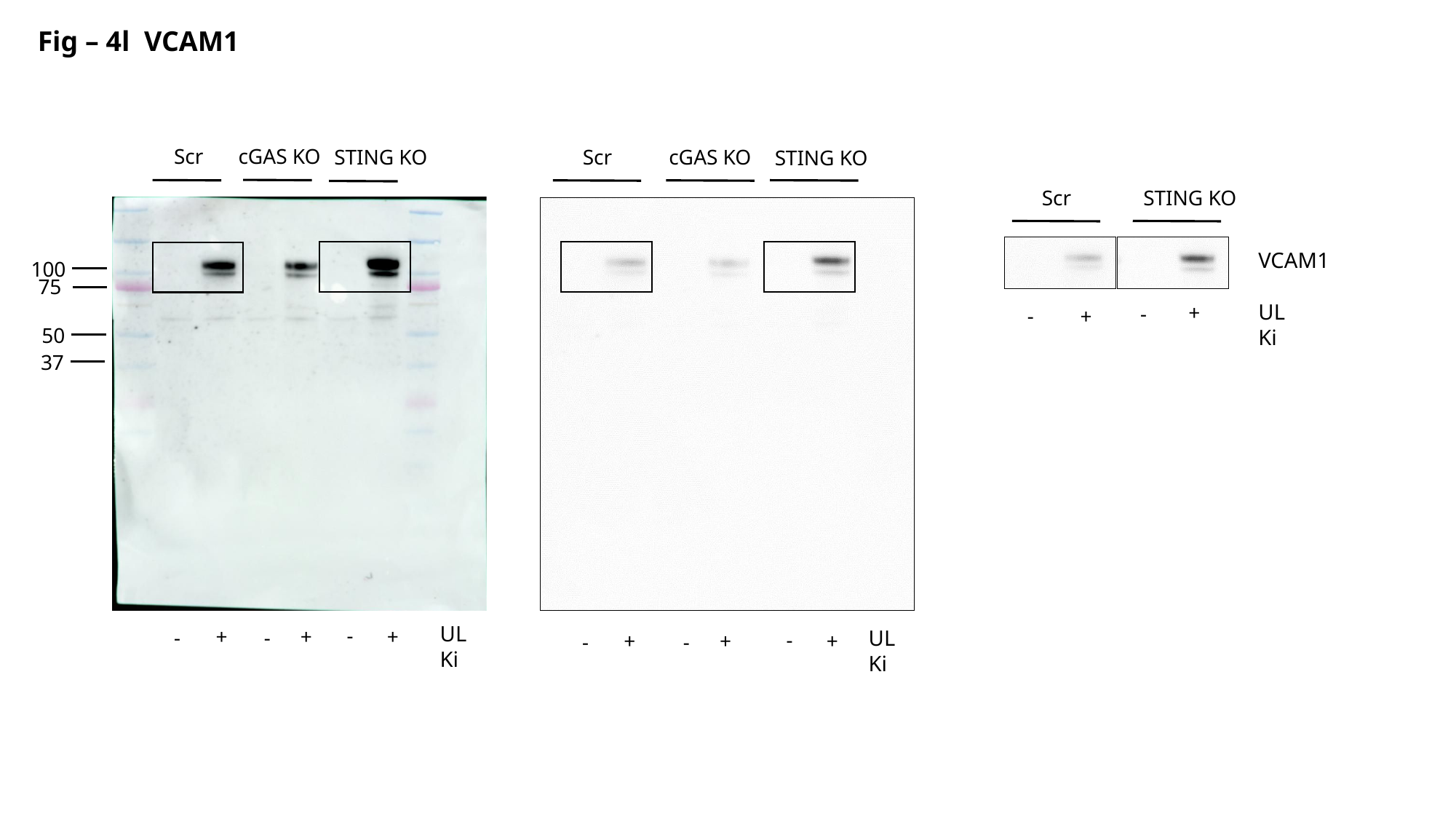

Fig – 4l VCAM1
cGAS KO
Scr
STING KO
cGAS KO
Scr
STING KO
STING KO
Scr
VCAM1
100
75
ULKi
+
-
+
-
50
37
ULKi
+
-
+
+
-
ULKi
-
-
+
+
+
-
-

## Slide 2
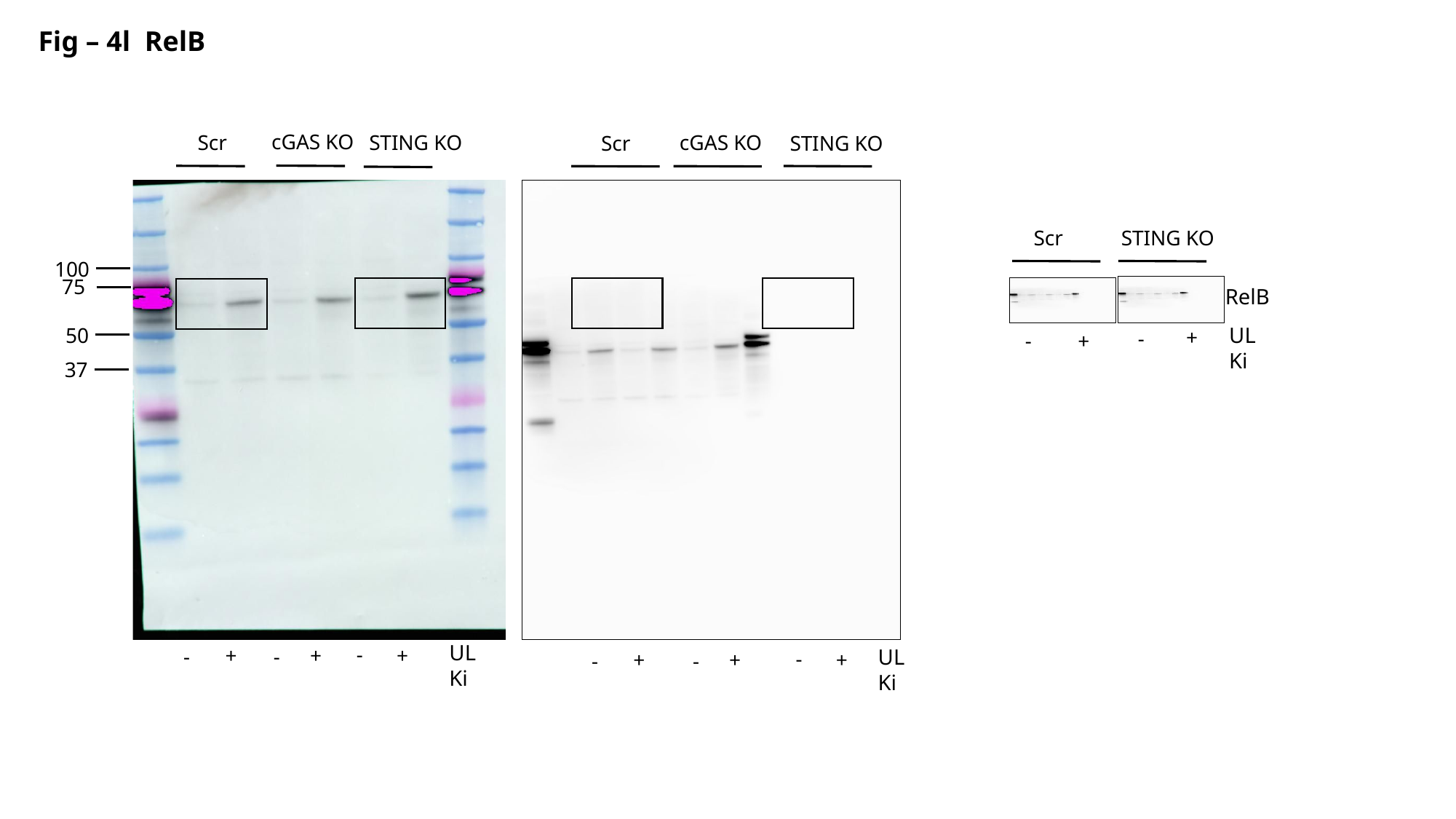

Fig – 4l RelB
cGAS KO
Scr
STING KO
cGAS KO
Scr
STING KO
STING KO
Scr
100
75
RelB
ULKi
50
+
-
+
-
37
ULKi
+
-
+
+
-
ULKi
-
-
+
+
+
-
-

## Slide 3
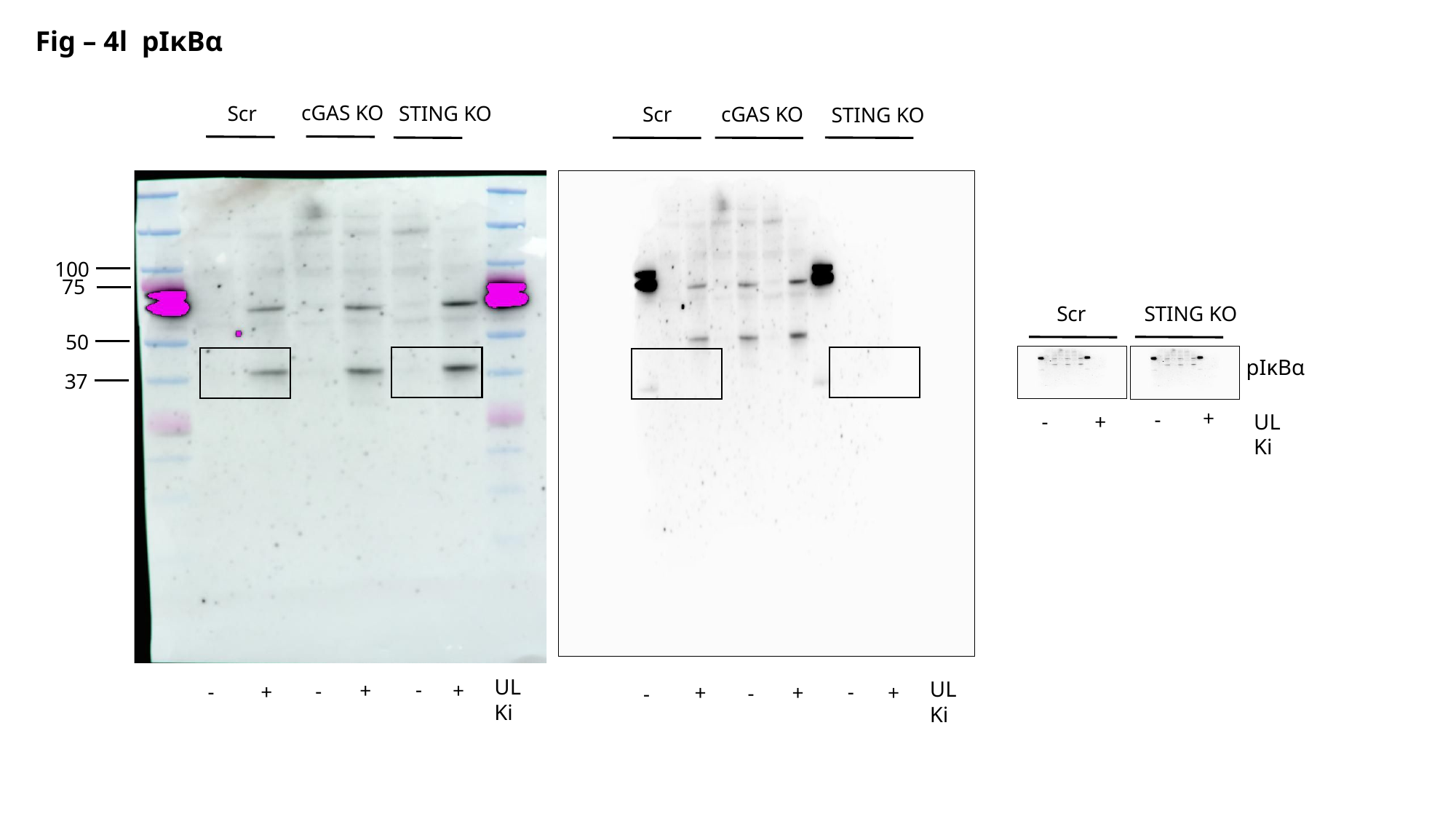

Fig – 4l pIκBα
cGAS KO
Scr
STING KO
cGAS KO
Scr
STING KO
100
75
STING KO
Scr
50
pIκBα
37
+
-
+
ULKi
-
ULKi
ULKi
-
+
+
+
-
-
-
+
+
+
-
-

## Slide 4
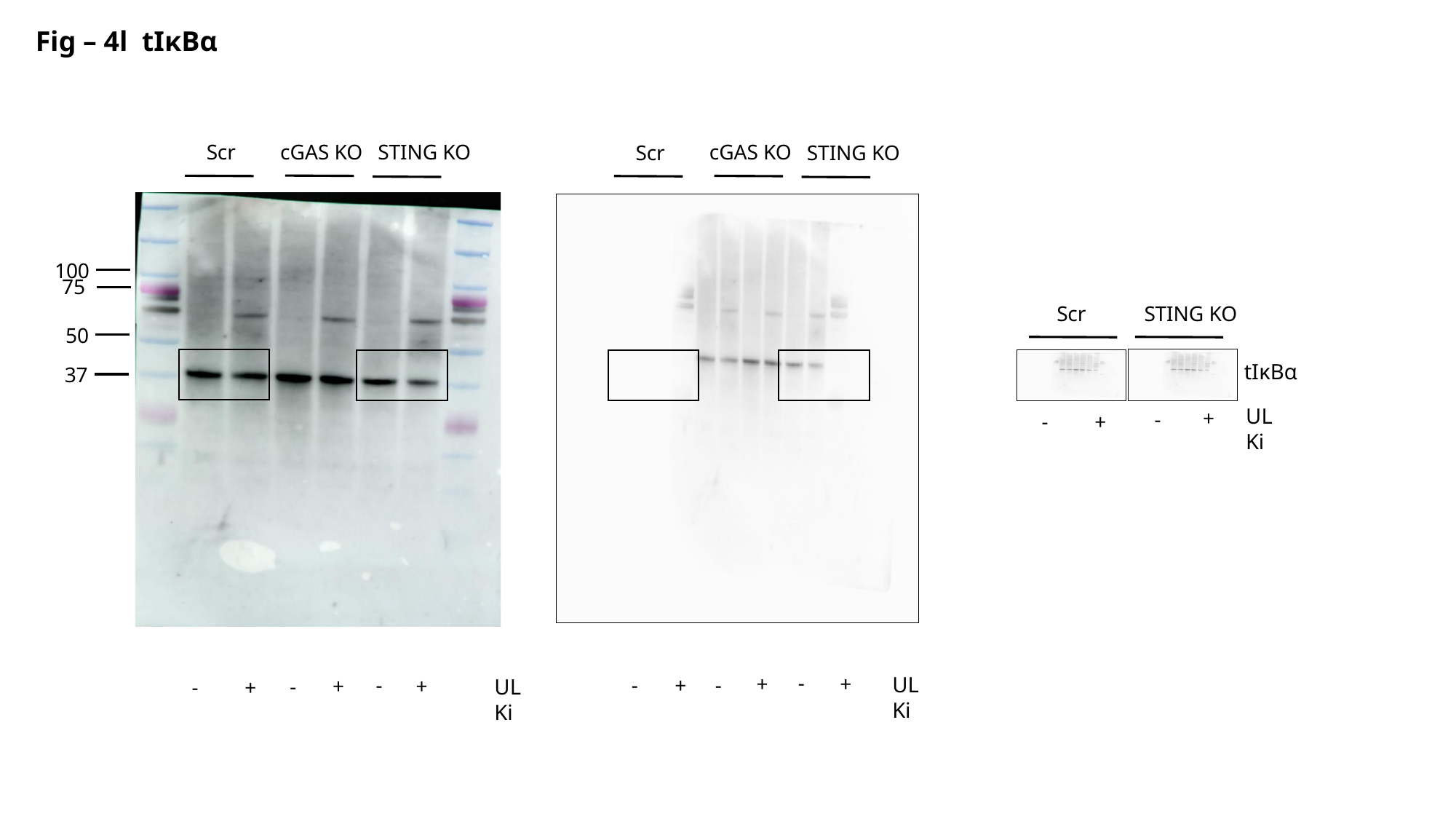

Fig – 4l tIκBα
cGAS KO
cGAS KO
Scr
STING KO
Scr
STING KO
100
75
STING KO
Scr
50
tIκBα
37
ULKi
+
-
+
-
-
+
+
ULKi
+
-
-
+
+
-
+
ULKi
-
-

## Slide 5
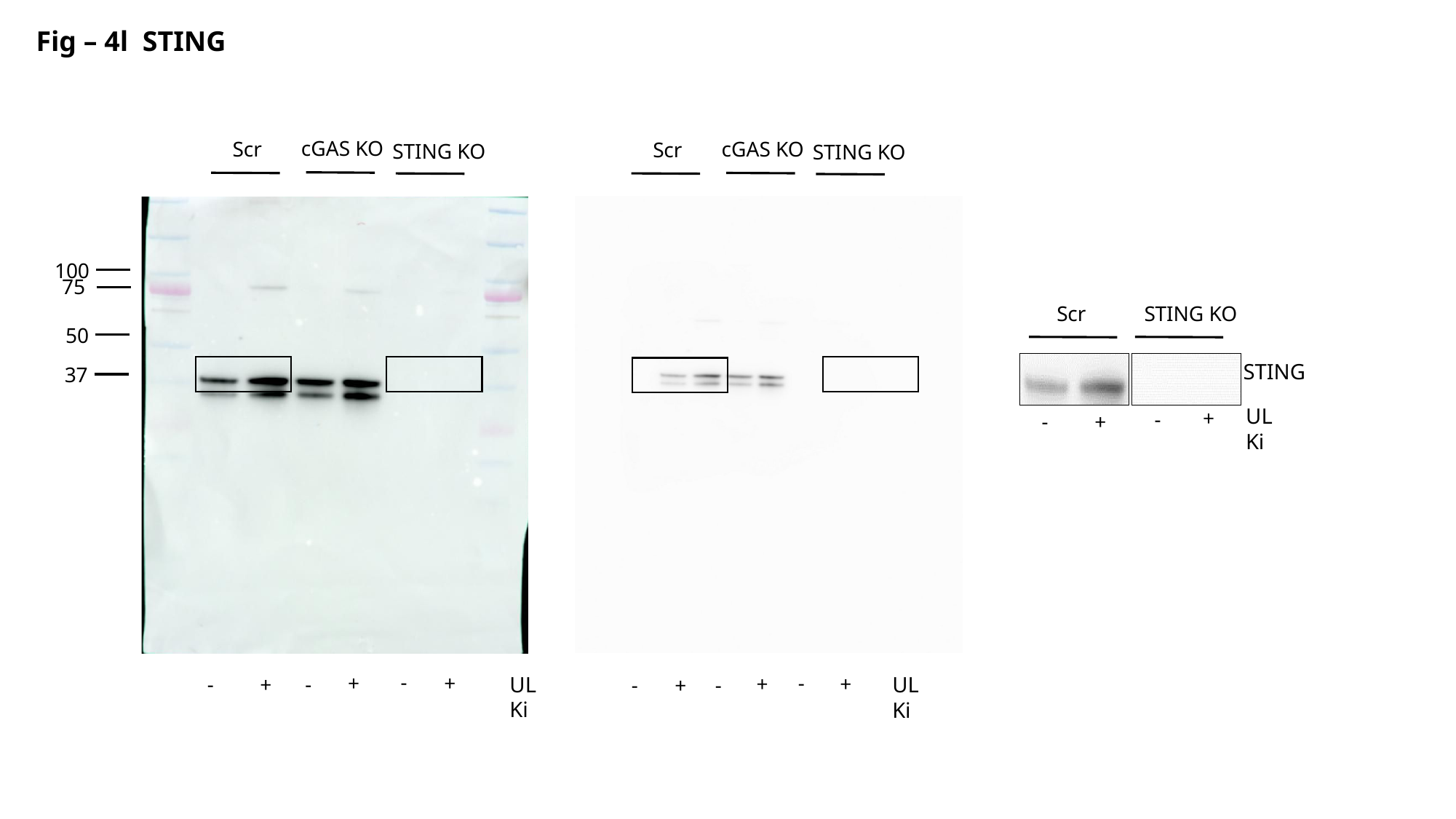

Fig – 4l STING
cGAS KO
cGAS KO
Scr
Scr
STING KO
STING KO
100
75
STING KO
Scr
50
STING
37
ULKi
+
-
+
-
-
+
+
-
+
+
+
ULKi
-
ULKi
-
+
-
-

## Slide 6
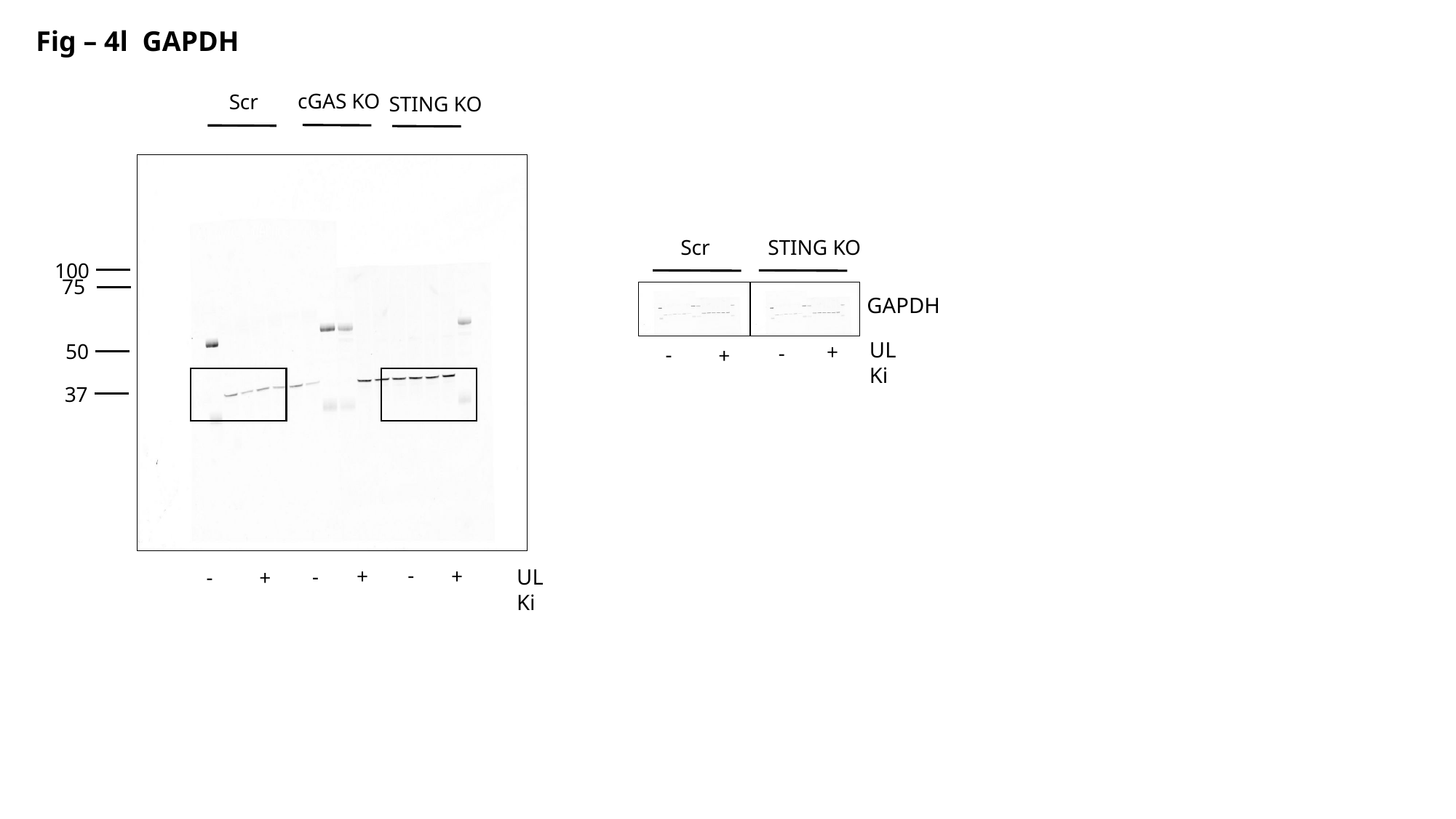

Fig – 4l GAPDH
cGAS KO
Scr
STING KO
STING KO
Scr
100
75
GAPDH
ULKi
+
50
-
+
-
37
-
+
+
+
ULKi
-
-

## Slide 7
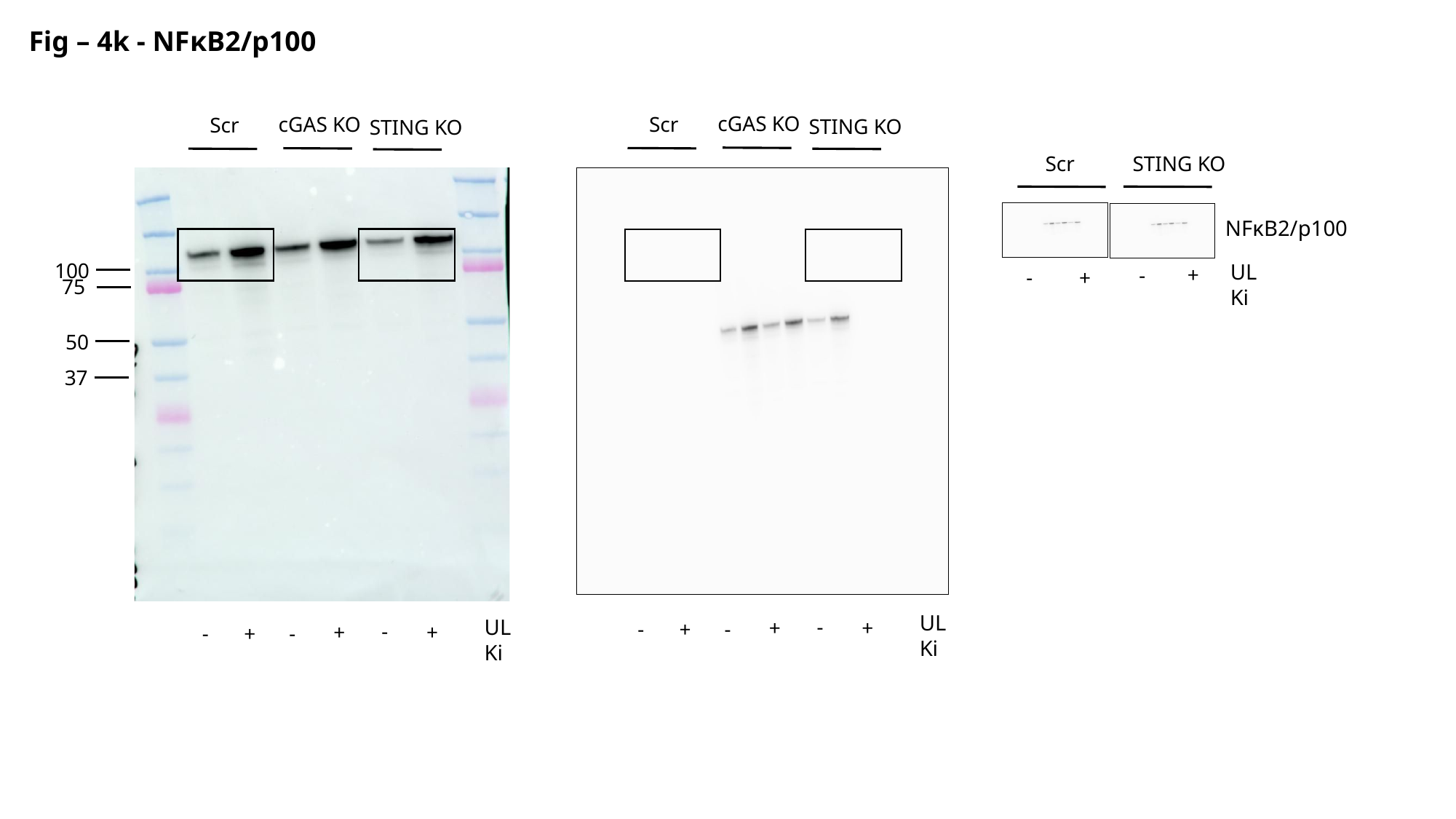

Fig – 4k - NFκB2/p100
cGAS KO
cGAS KO
Scr
Scr
STING KO
STING KO
STING KO
Scr
NFκB2/p100
100
ULKi
+
-
+
-
75
50
37
ULKi
ULKi
-
+
+
+
-
-
-
+
+
+
-
-

## Slide 8
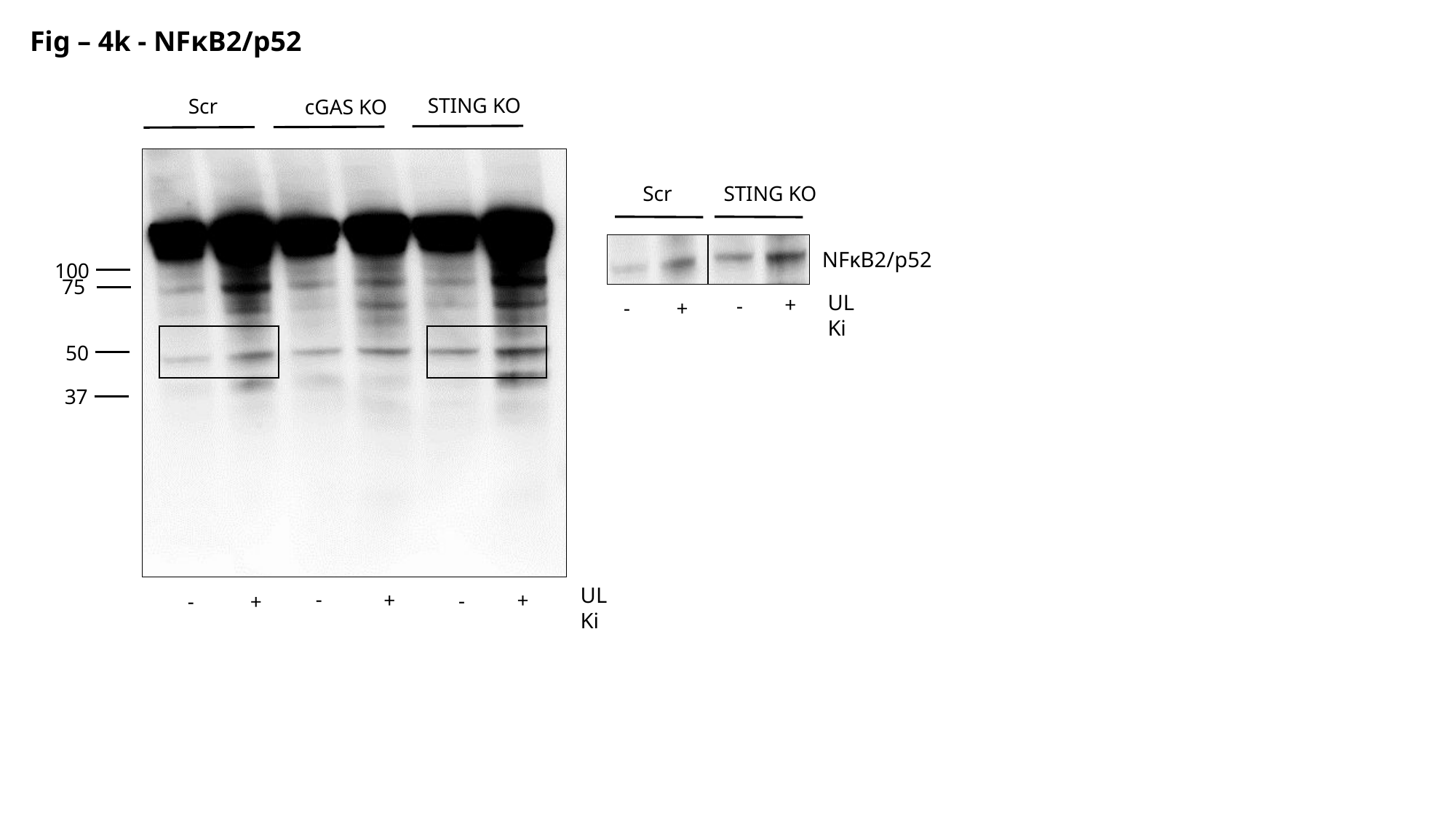

Fig – 4k - NFκB2/p52
STING KO
Scr
cGAS KO
STING KO
Scr
NFκB2/p52
100
75
ULKi
+
-
+
-
50
37
ULKi
-
+
+
+
-
-

## Slide 9
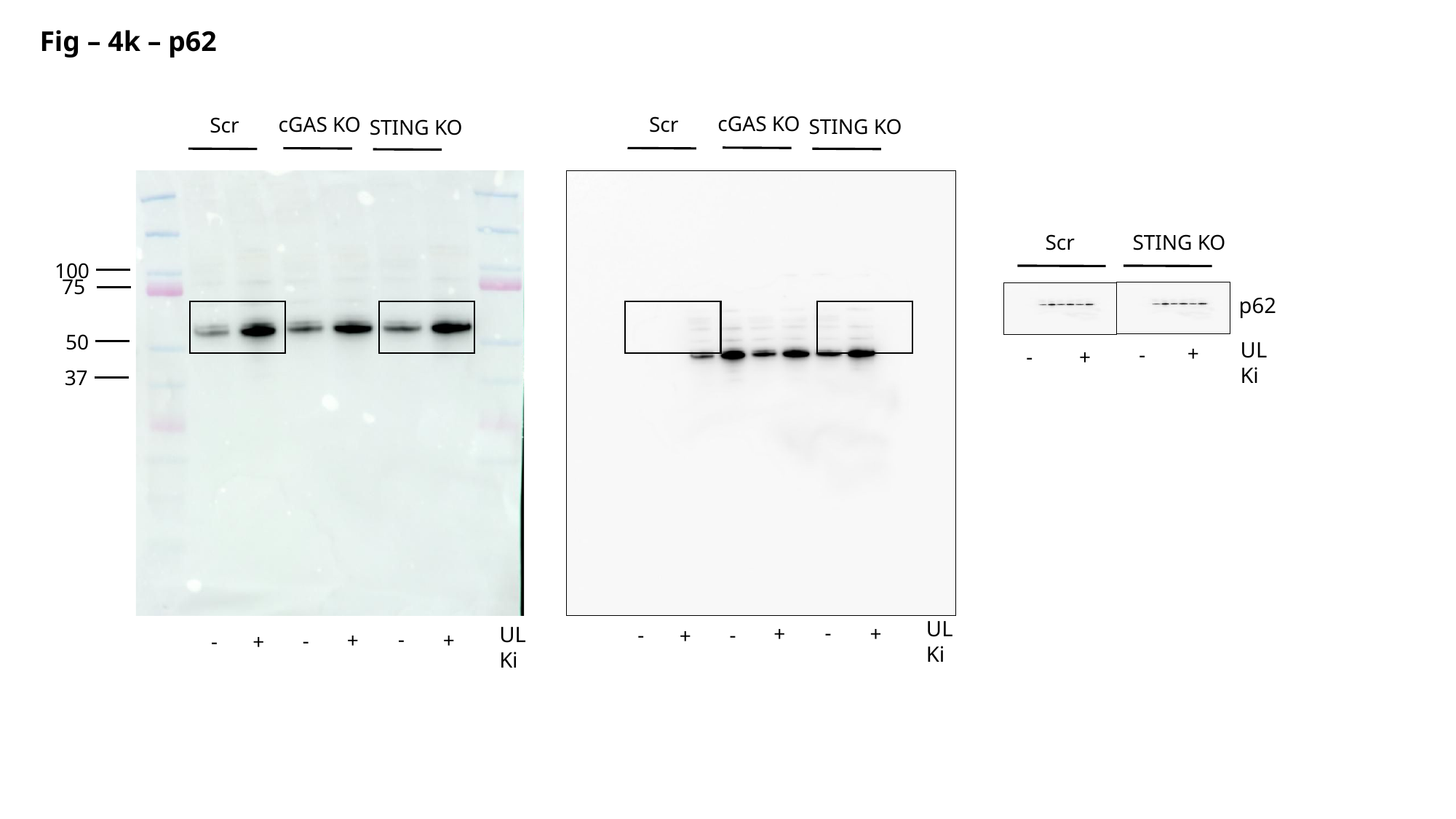

Fig – 4k – p62
cGAS KO
cGAS KO
Scr
Scr
STING KO
STING KO
STING KO
Scr
100
75
p62
50
ULKi
+
-
+
-
37
ULKi
-
+
+
ULKi
+
-
-
-
+
+
+
-
-

## Slide 10
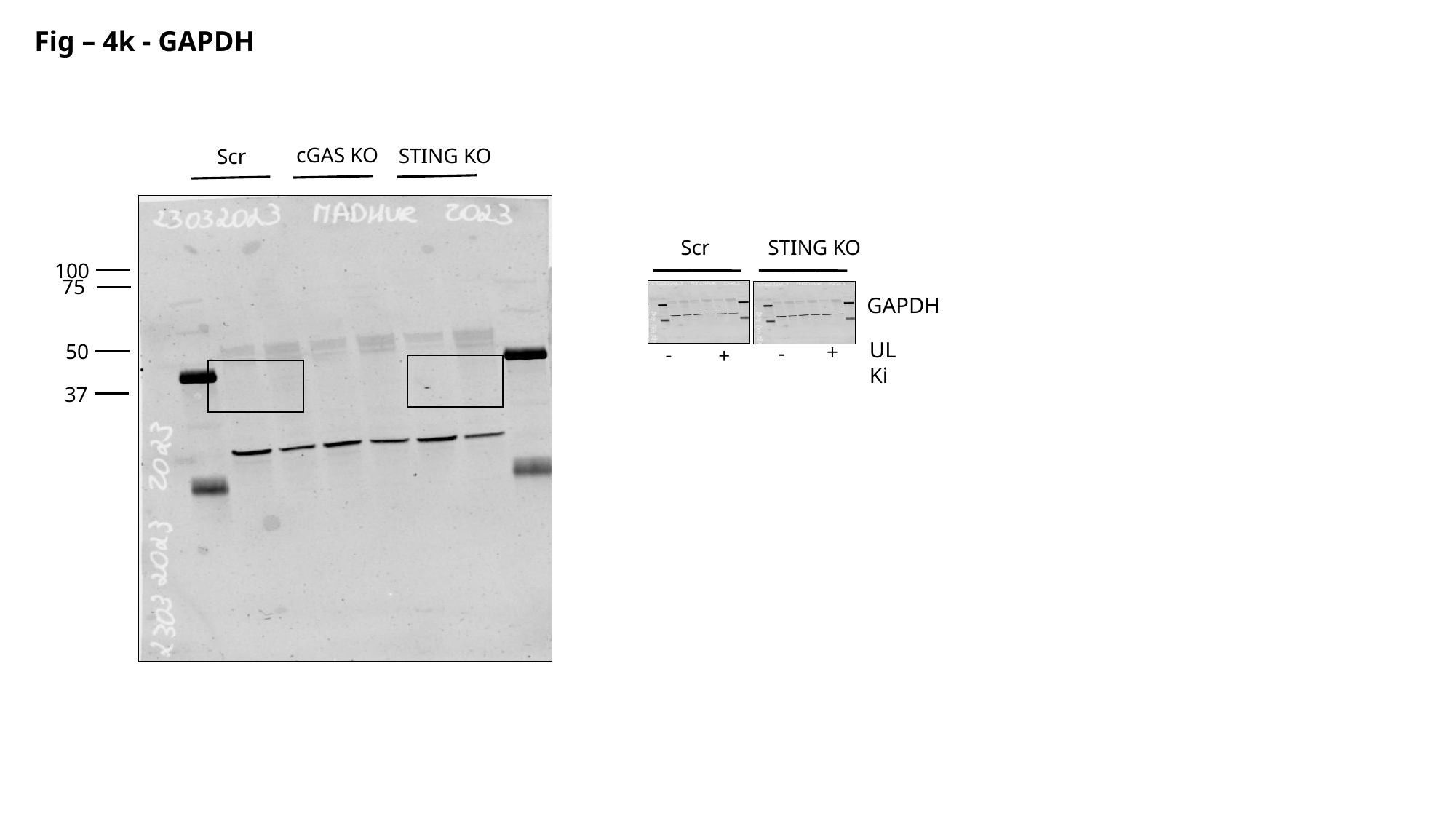

Fig – 4k - GAPDH
cGAS KO
STING KO
Scr
STING KO
Scr
100
75
GAPDH
ULKi
+
50
-
+
-
37
-
+
+
+
ULKi
-
-
