# Supplementary material for: Tumor endothelial cell autophagy is a key vascular‐immune checkpoint in melanoma
Source: EMBO Mol Med. 2023 Nov 27;15(12):e18028. doi: 10.15252/emmm.202318028 (PMC10701618; doi:10.15252/emmm.202318028)
Supplement: Supplementary file 10 — Source Data for Figure 5 [file EMMM-15-e18028-s011.zip › figure_5_raw_data/5d/README.docx]

README:

Source data for Figures 5 D, E and G can be found on Bio Image Archive under access number S-BSST1202.
