# Supplementary material for: Tumor endothelial cell autophagy is a key vascular‐immune checkpoint in melanoma
Source: EMBO Mol Med. 2023 Nov 27;15(12):e18028. doi: 10.15252/emmm.202318028 (PMC10701618; doi:10.15252/emmm.202318028)
Supplement: Supplementary file 11 — Source Data for Figure 6 [file EMMM-15-e18028-s005.zip › figure_6_raw_data/6_e,f,g/READ_ME.docx]

Raw sequencing reads of all scRNA-seq have been deposited in the European Genome-phenome Archive (EGA) under study no. EGAS00001006488. Requests for accessing raw sequencing reads will be reviewed by the UZ Leuven-VIB data access committee.
