# Supplementary material for: Tumor endothelial cell autophagy is a key vascular‐immune checkpoint in melanoma
Source: EMBO Mol Med. 2023 Nov 27;15(12):e18028. doi: 10.15252/emmm.202318028 (PMC10701618; doi:10.15252/emmm.202318028)
Supplement: Supplementary file 12 — Source Data for Figure 7 [file EMMM-15-e18028-s013.zip › figure_7_raw_data/7e/READ_ME.docx]

The following algorithm was used in R to Generate Graphs for figure 7E,F,G,H. :

sqrt[ (X coordinate of BV - X coordinate of Tcy)^2 + (Y coordinate of BV - Y coordinate of Tcy)^2 ]

**The raw data table containing all x, y coordinates can be found under folder figure 7E excel file named Raw data Milan analysis.**

X and Y are coordinates of cells. This is what was used to calculate distances.
Tumor_area column contains information whether you are looking at tumor, peritumor or background regions of the tissue.
CellType column contains the different celltypes.

Cytotoxic T Cells were deemed as being CD8+. 

In addition to this, to generate figures for peritumors, the following were the values for deeming markers as positive:
VCAM1+ = expression > 0.5
STING+ = expression > 0.5
PD1+ = expression > 1
GRB7 (Grzb) = expression > 1

to generate figures for tumors, the following were the values for deeming markers as positive:
VCAM1+ = expression > 2
STING was not positive in tumors
PD1+ = expression > 2
GRB7 (Grzb) = expression > 1.5
