# Supplementary material for: Tumor endothelial cell autophagy is a key vascular‐immune checkpoint in melanoma
Source: EMBO Mol Med. 2023 Nov 27;15(12):e18028. doi: 10.15252/emmm.202318028 (PMC10701618; doi:10.15252/emmm.202318028)
Supplement: Supplementary file 13 [file EMMM-15-e18028-s006.pdf]

EMBO Molecular Medicine

TUMOR ENDOTHELIAL CELL AUTOPHAGY IS A KEY VASCULAR-IMMUNE CHECKPOINT IN MELANOMA

Patrizia Agostinis, Jelle Verhoeven, Kathryn Jacobs, Francesca Rizzollo, Francesca Lodi, Yichao Hua, Joanna Poźniak, Adhithya Narayanan Srinivasan, Diede Houbaert, Gautam Shankar, Sanket More, Marco Schaaf, Nikolina Dubroja Lakic, Maarten Ganne, Jochen Lamote, Johan Van Weyenbergh, Louis Boon, Oliver Bechter, Francesca Maria Bosisio, Yasuo Uchiyama, Mathieu Bertrand, Jean Christophe Marine, Diether Lambrechts, Gabriele Bergers, and Madhur Agrawal
DOI: 10.15252/emmm.202318028

Corresponding authors: Patrizia Agostinis (patrizia.agostinis@kuleuven.be) , Madhur Agrawal (agrawalmadhur@gmail.com)

Review Timeline:

Submission Date:	17th May 23
Editorial Decision:	8th Jun 23
Revision Received:	5th Oct 23
Editorial Decision:	18th Oct 23
Revision Received:	25th Oct 23
Accepted:	30th Oct 23

Editor: Lise Roth

Transaction Report:

(Note: With the exception of the correction of typographical or spelling errors that could be a source of ambiguity, letters and reports are not edited. Depending on transfer agreements, referee reports obtained elsewhere may or may not be included in this compilation. Referee reports are anonymous unless the Referee chooses to sign their reports.)

8th Jun 2023

Dear Patrizia,

Thank you for the submission of your manuscript to EMBO Molecular Medicine. We have now received feedback from the three reviewers who agreed to evaluate your manuscript. As you will see from the reports below, the referees acknowledge the interest of the study and are overall supporting publication of your work pending appropriate revisions.

Addressing the reviewers' concerns in full will be necessary for further considering the manuscript in our journal, and acceptance of the manuscript will entail a second round of review. EMBO Molecular Medicine encourages a single round of revision only and therefore, acceptance or rejection of the manuscript will depend on the completeness of your responses included in the next, final version of the manuscript. For this reason, and to save you from any frustrations in the end, I would strongly advise against returning an incomplete revision.

If you would like to discuss further the points raised by the referees, I am available to do so via email or video. Let me know if you are interested in this option.

Revised manuscripts should be submitted within three months of a request for revision; they will otherwise be treated as new submissions, except under exceptional circumstances in which a short extension is obtained from the editor.

When submitting your revised manuscript, please carefully review the instructions that follow below. We perform an initial quality control of all revised manuscripts before re-review; failure to include requested items will delay the evaluation of your revision.

We require:

1) A .docx formatted version of the manuscript text (including legends for main figures, EV figures and tables). Please make sure that the changes are highlighted to be clearly visible.

2) Individual production quality figure files as .eps, .tif, .jpg (one file per figure). For guidance, download the 'Figure Guide PDF' (https://www.embopress.org/page/journal/17574684/authorguide#figureformat).

3) At EMBO Press we ask authors to provide source data for the main figures. Our source data coordinator will contact you to discuss which figure panels we would need source data for and will also provide you with helpful tips on how to upload and organize the files.

4) A .docx formatted letter INCLUDING the reviewers' reports and your detailed point-by-point responses to their comments. As part of the EMBO Press transparent editorial process, the point-by-point response is part of the Review Process File (RPF), which will be published alongside your paper.

5) A complete author checklist, which you can download from our author guidelines (https://www.embopress.org/page/journal/17574684/authorguide#submissionofrevisions). Please insert information in the checklist that is also reflected in the manuscript. The completed author checklist will also be part of the RPF.

6) Please note that all corresponding authors are required to supply an ORCID ID for their name upon submission of a revised manuscript. An ORCID identifier is currently missing for Dr. Madhur Agrawal.

7) It is mandatory to include a 'Data Availability' section after the Materials and Methods. Before submitting your revision, primary datasets produced in this study need to be deposited in an appropriate public database, and the accession numbers and database listed under 'Data Availability'. Please remember to provide a reviewer password if the datasets are not yet public (see https://www.embopress.org/page/journal/17574684/authorguide#dataavailability).

In case you have no data that requires deposition in a public database, please state so in this section ("This study includes no data deposited in external repositories"). Note that the Data Availability Section is restricted to new primary data that are part of this study.
8) For data quantification: please specify the name of the statistical test used to generate error bars and P values, the number (n) of independent experiments (specify technical or biological replicates) underlying each data point and the test used to calculate p-values in each figure legend. The figure legends should contain a basic description of n, P and the test applied. Graphs must include a description of the bars and the error bars (s.d., s.e.m.). Please provide exact p values.

9) Our journal encourages inclusion of *data citations in the reference list* to directly cite datasets that were re-used and

obtained from public databases. Data citations in the article text are distinct from normal bibliographical citations and should directly link to the database records from which the data can be accessed. In the main text, data citations are formatted as follows: "Data ref: Smith et al, 2001" or "Data ref: NCBI Sequence Read Archive PRJNA342805, 2017". In the Reference list, data citations must be labeled with "[DATASET]". A data reference must provide the database name, accession number/identifiers and a resolvable link to the landing page from which the data can be accessed at the end of the reference. Further instructions are available at .

10) We replaced Supplementary Information with Expanded View (EV) Figures and Tables that are collapsible/expandable online. A maximum of 5 EV Figures can be typeset. EV Figures should be cited as 'Figure EV1, Figure EV2" etc... in the text and their respective legends should be included in the main text after the legends of regular figures.
- For the figures that you do NOT wish to display as Expanded View figures, they should be bundled together with their legends in a single PDF file called *Appendix*, which should start with a short Table of Content. Appendix figures should be referred to in the main text as: "Appendix Figure S1, Appendix Figure S2" etc.
- Additional Tables/Datasets should be labeled and referred to as Table EV1, Dataset EV1, etc. Legends have to be provided in a separate tab in case of .xls files. Alternatively, the legend can be supplied as a separate text file (README) and zipped together with the Table/Dataset file.
See detailed instructions here:

11) The paper explained: EMBO Molecular Medicine articles are accompanied by a summary of the articles to emphasize the major findings in the paper and their medical implications for the non-specialist reader. Please provide a draft summary of your article highlighting
- the medical issue you are addressing,
- the results obtained and
- their clinical impact.
This may be edited to ensure that readers understand the significance and context of the research. Please refer to any of our published articles for an example.

12) For more information: There is space at the end of each article to list relevant web links for further consultation by our readers. Could you identify some relevant ones and provide such information as well? Some examples are patient associations, relevant databases, OMIM/proteins/genes links, author's websites, etc...

13) Author contributions: CRediT has replaced the traditional author contributions section because it offers a systematic machine readable author contributions format that allows for more effective research assessment. Please remove the Authors Contributions from the manuscript and use the free text boxes beneath each contributing author's name in our system to add specific details on the author's contribution. More information is available in our guide to authors.

14) Disclosure statement and competing interests: We updated our journal's competing interests policy in January 2022 and request authors to consider both actual and perceived competing interests. Please review the policy https://www.embopress.org/competing-interests and update your competing interests if necessary.

15) Every published paper now includes a 'Synopsis' to further enhance discoverability. Synopses are displayed on the journal webpage and are freely accessible to all readers. They include a short stand first (maximum of 300 characters, including space) as well as 2-5 one-sentences bullet points that summarizes the paper. Please write the bullet points to summarize the key NEW findings. They should be designed to be complementary to the abstract - i.e. not repeat the same text. We encourage inclusion of key acronyms and quantitative information (maximum of 30 words / bullet point). Please use the passive voice. Please attach these in a separate file or send them by email, we will incorporate them accordingly.

Please also suggest a striking image or visual abstract to illustrate your article as a PNG file 550 px wide x 300-600 px high.

16) As part of the EMBO Publications transparent editorial process initiative (see our Editorial at http://embomolmed.embopress.org/content/2/9/329), EMBO Molecular Medicine will publish online a Review Process File (RPF) to accompany accepted manuscripts.
In the event of acceptance, this file will be published in conjunction with your paper and will include the anonymous referee reports, your point-by-point response and all pertinent correspondence relating to the manuscript. Let us know whether you agree with the publication of the RPF and as here, if you want to remove or not any figures from it prior to publication.
Please note that the Authors checklist will be published at the end of the RPF.

EMBO Molecular Medicine has a "scooping protection" policy, whereby similar findings that are published by others during review or revision are not a criterion for rejection. Should you decide to submit a revised version, I do ask that you get in touch after three months if you have not completed it, to update us on the status.

I look forward to receiving your revised manuscript.

With kind regards,

Lise

Lise Roth, PhD
Senior Editor
EMBO Molecular Medicine

EMBO Press participates in many Publish and Read agreements that allow authors to publish Open Access with reduced/no publication charges. Check your eligibility: https://authorservices.wiley.com/author-resources/Journal-Authors/open-access/affiliation-policies-payments/index.html

***** Reviewer's comments *****

Referee #1 (Remarks for Author):

In their manuscript, by using a series of endothelium-specific in vivo mouse models of autophagic gene deletion in combination with syngeneic cell lines differing in the degree of immunogenicity, Verhoeven et al. characterize the role that tumor endothelial cell autophagy may play in the response to immunotherapy treatment of melanoma in an elegant, detailed, and precise manner. Furthermore, by analyzing pre- and post-treatment tissues from a cohort of human melanoma patients who were either responsive or unresponsive to immunotherapy, the authors substantiate the clinical validity of the observations they obtained in preclinical models by showing how an inflamed endothelial cell signature may predict the response of melanoma patients to the immunostimulatory therapy. The work is innovative and interesting. Yet, some aspects still need to be studied or clarified. In addition, a significant number of typos and errors in referencing the panels of different figures mentioned in the text need to be fixed.

Major issues

1. In the manuscript, ATG5BECKO, ATG9ECKO and ATG12ECKO animals are subcutaneously injected with different murine melanoma cell lines. ATG5 and ATG12 are assumed to be epistatic and thus, the study of two different lines is fine. However, ATG9ECKO is only studied with the poorly immunogenic B16-F10 melanoma cell line. Authors should study ATG9ECKO animals also with the immunogenic YUMMER 1.7 melanoma cell line.

2. Similar to point 1 above and to fully verify in preclinical models what has been observed in patients, the impact of anti-PD1 monotherapy in WT and ATG5BECKO mice should not only be tested using the poorly immunogenic melanoma cell line B16F10, but also the immunogenic cell line YUMMER 1.7.

3. In lines 180-181, the Authors describe the sorting of tumor endothelial cells (TECs) employed for the Nanostring technology, which yielded more than 90% pure TECs. The Authors should provide a figure of this FACS gating strategy, as shown in Extended data Fig.2a.

4. In lines 234 and 235, Authors states "Remarkably, we observed a significant inverse correlations between the signatures in all of these cancer types (Fig 3g)." However, the p-value in non-small-cell lung cancer (NSCLC) is non-significant and this aspect should be mentioned. Moreover, the R determined for the breast cancer (BC) subgroup is quite close to 0, but the p-value of this subgroup is very significant, while I would have anticipated similar results to the NSCLC subgroup: may author verify and comment on this aspect.

5. In lines 315 and 316, the double knock-out of ATG5 and STING led to variations across human EC donors, leading to unreliable results. It could be interesting to have a representation of this variability. Does the knock-down of STING in ATG5-deficient HUVECs lead to the same variability?

6. In Fig.6f and Extended data Fig.6d, the muTEC-DE signature is tested in the different types of TECs. In the discussion (lines 466-468), Authors write: "the enrichment of the muTEC-DE was associated with the lowest expression levels of autophagy genes, particularly in the Interferon EC subtype". However, graphs indicating the decrease in the autophagy signature

corresponding to the EC subtypes are missing, therefore this association are not shown, while it should be

Minor issues

1. Line 58, what TME stands for should be mentioned.

2. Lines 105 and 108, "Recently, in a model of acute... in a model of acute physiological inflammation". "Acute physiological inflammation" is duplicated.

3. Lines 144 to 147, "We observed similar phenotypic differences in tumor burden when comparing WT and Atg12ECKO or Atg9ECKO mice inoculated with the immunogenic YUMMER 1.7 melanoma cells, carrying the BrafV600E/Pten−/−/Cdkn2a−/− mutations, or B16-F10, respectively (Fig. 1e-h)". For a better understanding of the text and the figures, Authors should rephrase. For example, they could write "We observed similar phenotypic differences in tumor burden when comparing WT and Atg9ECKO or Atg12ECKO mice inoculated with B16-F10 or with the immunogenic YUMMER 1.7 melanoma cells, carrying the BrafV600E/Pten−/−/Cdkn2a−/− mutations, respectively".

4. Line 146, "BrafV600E/Pten−/−/Cdkn2a−/−" should be written instead of "BrafV600E,Pten−/−/Cdkn2a−/−"

5. Line 156, Authors write "an indicator of a diminished immunosuppressive status of the TME". They should provide a reference supporting this sentence.

6. Line 165, word "mice" is duplicated.

7. Line 186, it should be Fig.3c instead of Fig.3b.

8. Line 202, Authors write "the levels of VCAM1, ICAM1 and of STING". The preposition "of" before "STING" should be removed.

9. Line 202, the verb "is" is duplicated.

10. Line 263, The Extended data Fig.4f has a FACS gating and a graph that does not correspond to each other. Thus, the Extended data Fig.4f in line 263 should be extended data Fig.4g and all the label of all the following Extended data Fig.4 should be changed:
a. Line 279, should be Extended data Fig.4h
b. Lines 282 and 284, should be Extended data Fig.4i
c. Line 297, Extended data Fig.4j instead of Extended data Fig.4i

11. Line 272 and 273, similarly to what mentioned in point 1, what "ER" and "ERGIC" stand for should be mentioned.

12. Line 287, there is a missing bracket at the end of the sentence.

13. Line 294, Fig.4k should be Fig.4i instead.

14. Line 310, there is a typo on the word "both".

15. Lines 334-335, Authors write "monitored tumor growth and inspected the TEC phenotype by IHC for the presence of proinflammatory/immunomodulatory factors". However, the images shown correspond to immunofluorescence and not immunohistochemistry.

16. Lines 368-370, Authors write "Our previous analysis indicated that particularly, the huTEC interferon subtype, of treatment-naïve cancer patients, exhibited a divergent association between the expression of muTEC-DEHigh and autophagyLow gene signatures". This statement is not clear and Authors should rephrase it. For example, they could write "Our previous analysis indicated that particularly, the huTEC interferon subtype of treatment-naïve cancer patients, exhibited a divergent association between the expression of muTEC-DEHigh and autophagyLow gene signatures".

17. Lines 375 and 394, the abbreviation "ON" (for ON treatment) does not correspond with the abbreviation showed in Fig.6d-f and Extended data Fig.6d.

18. Lines 384 and 387, should refer to Fig.6e and not to Fig.4e.

19. Line 392, should refer to Extended data Fig.6b,c and not to Extended data Fig.6a,b.

20. Line 392, Authors write "Compared to non-responders (NR), melanoma patients, responding to". This statement is not clear,

and Authors should rephrase it. I would write "Compared to non-responders (NR), melanoma patients responding to".

21. Line 395, the figures mentioned are (Fig. 6h; Extended data Fig. 6c), when should be Fig.6f and Extended data Fig.6d.

22. Lines 452-454, in Line 453 there is a clear anacoluthon. I would suggest to rather remove line 453 or connect it by a comma with the line 452.

23. Line 536, Authors write "cell expression, of STING.". The comma should be removed and Authors should write "cell expression of STING."

24. Lines 616-617, Authors should remark that the double stimulation mentioned in these lines is for the co-culture with Jurkat cells.

25. Line 617, it is written that the incubation is for 12h. However, in line 1232 is written 24h incubation. What is the correct timing?

26. Line 724 has a typo. Adhering to the USA English style, the word "analysed" should be "analyzed".

27. Line 764, replace the comma at the end of the sentence by a full stop.

28. Line 807, it is written "frenquenT-cell" instead of "frequent".

29. Lines 1087-1088, should be Kaplan-Meier instead of "Kapelin-meyer".

30. Line 1101 has a typo. Adhering to the USA English style, the word "tumours" should be "tumors".

31. Line 1111, in the sentence "in and HUVECs", remove "and".

32. Line 1123, missing capital letters in HUVECs.

33. Line 1125, should include the reference to the Fig.4l, corresponding to ULK1/2 inhibition.

34. Line 1126, the end of the sentence should be a full stop and not a bracket.

35. Lines 1128-1129, Fig.4m should be Fig.4l.

36. Lines 1160 and 1172, the "track changes" marks of Word should be removed.

37. Line 1184, duplication of "subcutaneous B16-F10 tumors".

38. Lines 1198 and 1206, "mice" instead of "Mice".

39. Line 1209, "10µm VCAM1" should be "10µm for VCAM1".

40. Line 1219, "In" should be "in".

41. Line 1229, typo in "cytometric".

42. Lines 1229 and 1235, "Ctr" is highlighted in grey.

43. Lines 1258-1259, "UMAP of ECs color coded for the indicated cell types" sentence is duplicated.

44. Extended data Fig.3, as in the other figure legends, also write the scale bar µm value.

45. Fig.4i, the title of the top of the graph can be confusing with the Y axes title. I would suggest using the graphs title as Y axes title and remove the title of the top of the graph.

46. Fig.5c, Authors should put tumor volume (mm3) as title of Y axes, as in the other figures representing the same endpoint.

47. Extended data Fig.3b, similarly to what mentioned in points 1 and 11, what "FOV" stands for should be mentioned.

48. Extended data Fig.4e, the ULK1/2 should indicate that Y axes is the fold change, as in the Bafilomycin A graphs

49. Fig.4l, to keep the same nomenclature as in the Western Blot, Authors should write "Ctr" instead of "Scr".

Referee #2 (Comments on Novelty/Model System for Author):

The creation of various Atg EC conditional KO mouse models did provide a unique system to investigate the role of autophagy in ECs in regulation of tumor immune surveillance and tumor growth, as well as to responsiveness to immunotherapy.

Referee #2 (Remarks for Author):

In this study, Verhoeven et al made a great deal of efforts in addressing a key question whether autophagy in tumor endothelial cells (TECs) plays an important role in modulating immune surveillance and tumor growth, mainly by using melanoma-bearing mouse models with defective autophagy, including conditional deletion of Atg5, Atg12 and Atig9 in ECs. The main conclusion of this study is that autophagy in TEC is capable of suppressing immune surveillance of the host against tumor cells and thus promoting tumor growth.
There are several important issues to be addressed to improve the study.
1) The concept of tumor endothelial cells (TECs) is not well described. More importantly, it is not clear how the authors differentiate the TECs from the no tumor endothelial cells in the animal models in vivo, based on the fact that the conditional deletion of Atgs was in all ECs.
2) In this study, the authors did not investigate the effect of autophagy on ECs including TECs, such as the viability and proliferation of TECs. In other words, it is not clear how deletion of Atgs in ECs would affect angiogenesis in the xenografted melanoma. I believe that this is a critical point overlooked by the authors in this study.
3) Figure 1: The authors should present evidence for the defective autophagy in ECs with deletion of Atgs (Atg5, Atg9 and Atg12).
4) Figure 2: The panel g and h appear to be redundant data, both showing the tumor volume changes over time among different groups.
5) Figure 3f - 3h: Data from these 3 panels were from bioinformatics analysis from primary human cancers: Better to move to suppl figures.
6) Figure 4k: The authors should present evidence demonstrating the impaired autophagic flux in ATG5 KO HUVECs.
7) The authors should present a summary illustration to highlight the main findings of this study, either as a separate figure or as the last panel in Figure 7.

Referee #3 (Comments on Novelty/Model System for Author):

The major issue of this study is whether the inflammatory EC is required for immune or therapeutic response against tumor, so the autophagy in EC is a genuine immune checkpoint, or it is the secondary event in the "hot" tumor microenvironment. The data (e.g. Fig. 1i and 1j, 2c and 2e) suggested that inflammatory EC enhanced, but is not required for, the recruitment of activated T cells. In that case, for example, tumor may use dissipating factors (e.g. hypoxic or lactate) to suppress T cell activities and induce autophagy in EC simultaneously. This also explained the marginal effect of Atg5b KO on response of melanoma to anti-PD-1 in the mouse studies (Figure 6a-c). These should be discussed in the text.

Referee #3 (Remarks for Author):

In this study, the author showed that Atg5b/Atg9a/Atg12 KO in Pdgfb+ or Ve-cadh+ cells caused: (1) growth delay of melanoma in mice; (2) increased CD3+ cells accumulated around the blood vessels of the tumors; and (3) infiltration of activated T cells. The depletion antibody study demonstrated that the melanoma growth delay was caused by the increased activated T cells. Atg5b KO resulted in the inflammatory phenotypes of endothelial cells, especially STING and IFNB expression, and recruitment of immune cells. Sting KO in endothelial cells (EC) eliminated IFNB induction by Atg5b KO; however, it did not rescue melanoma growth delay in mice as well as other inflammatory phenotypes of EC caused by Atg5b KO. Finally, Atg5b KO in EC sensitized the melanoma to anti-PD-1 treatment in mouse studies.

The major issue of this study is whether the inflammatory EC is required for immune or therapeutic response against tumor, so the autophagy in EC is a genuine immune checkpoint, or it is the secondary event in the "hot" tumor microenvironment. The data (e.g. Fig. 1i and 1j, 2c and 2e) suggested that inflammatory EC enhanced, but is not required for, the recruitment of activated T cells. In that case, for example, tumor may use dissipating factors (e.g. hypoxic or lactate) to suppress T cell activities and induce autophagy in EC simultaneously. This also explained the marginal effect of Atg5b KO on response of melanoma to anti-PD-1 in the mouse studies (Figure 6a-c). These should be discussed in the text.

Technical question: are there any other cell types targeted by Pdgfb-CreERT2 or Ve-cadh-CreERT2 in tumor microenvironment?

Referee #1 (Remarks for Author):

Major issues

1. In the manuscript, ATG5BECKO, ATG9ECKO and ATG12ECKO animals are subcutaneously injected with different murine melanoma cell lines. ATG5 and ATG12 are assumed to be epistatic and thus, the study of two different lines is fine. However, ATG9ECKO is only studied with the poorly immunogenic B16-F10 melanoma cell line. Authors should study ATG9ECKO animals also with the immunogenic YUMMER 1.7 melanoma cell line.

Answer: To validate the implication of bona fide autophagy in our study we confirmed the effects on melanoma tumor burden control, observed in the ATG5^{ECKO} (Fig 1A,B) and ATG12^{ECKO} mice (Fig.1G,H) in the transgenic ATG9^{ECKO} mice (Fig1E,F). The original experiments of Fig1 E,F, were performed in the lab of our collaborator at the University of Gent, who has since stopped the mouse colony and transferred the mice to us. Currently, after the requested period of quarantine, the ATG9^{ECKO} mice are still breeding and unfortunately, at this stage we do not have enough pups for the trial suggested by the Reviewer, since it will require the use of <u>only male mice</u> for the inoculation of male-derived YUMMER 1.7 melanoma cells.
Waiting for the availability of a sufficient number of male mice to perform this experiment, will push back significantly the publication of our findings. As we do not believe it would alter the main message of our data, we hope the Reviewer will understand why we have not attempted to answer this suggestion.

2. Similar to point 1 above and to fully verify in preclinical models what has been observed in patients, the impact of anti-PD1 monotherapy in WT and ATG5BECKO mice should not only be tested using the poorly immunogenic melanoma cell line B16-F10, but also the immunogenic cell line YUMMER 1.7.

Answer: We thank the Reviewer for this suggestion. Indeed, in our study we used the rather immunogenic YUMMER 1.7 as an additional melanoma model, which conformed to the same observations for the B16-F10 tumors in terms of tumor burden control (Fig. 1g), increased localization of T lymphocytes at the tumor margins (Fig. 1J) and accrued levels of STING, ICAM1 and VCAM1 in the tumor vessels (Expanded View Fig. 3C) when compared with their respective wild type counterparts.
We agree with the Reviewer that the impact of anti-PD1 therapy could be further confirmed in this immunogenic melanoma model, although we purposefully used the 'cold' B16-F10 tumor as a paradigm of melanoma that would only partially respond to anti-PD1 monotherapy, to potentially appreciate the additional effects of the genetic loss of autophagy in an immunosuppressive melanoma.
However, to answer the reviewer's point we then performed the suggested experiment by growing YUMMER 1.7 in WT and ATG5^{BECKO} mice followed by treatment with IgG or anti-PD1, using the same schedule and protocol utilized for the B16-F10 tumor, and the same cell number for injection as in all other settings explored in our study that used the YUMMER 1.7 cells.
As shown in the **Rebuttal Letter (RL) Figure 1**, this proved to be technically challenging. In

our hands, administration of IgG alone or anti-PD1; in YUMMER 1.7 melanoma-bearing mice led to almost complete tumor rejection and thus failure to conduct the trial.

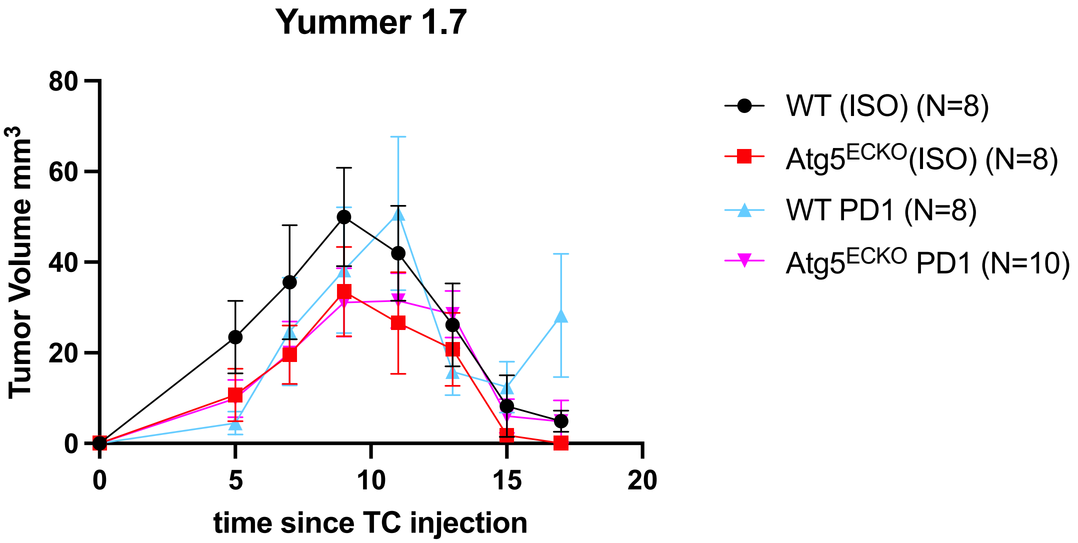

RL Figure 1. Grouped tumor volume of Yummer 1.7 subcutaneous tumor-bearing WT and $Atg5^{BECKO}$ mice injected with isotype (ISO) or αPD1 antibody. Mice were injected with 250,000 cells/tumors.

We were initially very surprised by these results and more specifically by the IgG group. Sporadic evidence exists showing that non-cancer-specific IgG may induce tumor regression. Upon further literature analysis, a recent study suggested that IgG treatment elicits tumor regression(Xu *et al*, 2019), this combined with the hot tumor environment generated by the YUMMER 1.7 cells, in these settings may have been enough to cause tumor rejection.
Please note that other authors using this immunogenic UV-induced melanoma found that unless a large number of these cells is inoculated (basically 4 times higher than those used here), YUMMER 1.7 tumors regress in a T cell-dependent manner (Zhang *et al*, 2022; Wang *et al*, 2017). Also increasing the number of injected cells, as in the paper of Wang et al, who increased YUMMER1.7 cell injection up to a million cells to prevent tumor rejection, would stimulate a complete response to ICB therapy. Hence we haven't pursued further immunotherapy experiments using such an increased number of YUMMER 1.7 cells. It is reasonable to hypothesize that the effects elicited by TEC-autophagy would be overruled by the efficient therapy-induced immunogenicity in this melanoma model.
Despite the limitations of this model in our settings, we surmise that the use of the more immunosuppressive B16-F10 melanoma in our anti-PD1 experiments along with the single cell RNAseq analysis of tumor ECs, of melanoma patients undergoing anti-PD1 therapy, which further unravels the inverse correlation between TEC-autophagy and the response to anti-PD1 monotherapy, together support the relevant conclusions of our study.

3. In lines 180-181, the Authors describe the sorting of tumor endothelial cells (TECs) employed for the Nanostring technology, which yielded more than 90% pure TECs. The Authors should provide a figure of this FACS gating strategy, as shown in Extended data Fig.2a.

Answer: We thank the Reviewer for raising this point. We have thus added the gating strategy to Figure EV3A.

4. In lines 234 and 235, the Authors states "Remarkably, we observed a significant inverse correlation between the signatures in all of these cancer types (Fig 3g)." However, the p-value in non-small-cell lung cancer (NSCLC) is non-significant and this aspect should be mentioned. Moreover, the R determined for the breast cancer (BC) subgroup is quite close to 0, but the p-value of this subgroup is very significant, while I would have anticipated similar results to the NSCLC subgroup: may author verify and comment on this aspect.

Answer: We thank the reviewer for highlighting this inconsistency. We have reanalyzed the scRNAseq data and they were consistent with what was reported. However, we have amended the text to say "*Remarkably, except for NSCLC, we observed a significant inverse correlation between the signatures in all other cancer types analyzed, albeit to a somehow reduced extent in breast cancer"* (Fig 3G)."

5. In lines 315 and 316, the double knock-out of ATG5 and STING led to variations across human EC donors, leading to unreliable results. It could be interesting to have a representation of this variability. Does the knock-down of STING in ATG5-deficient HUVECs lead to the same variability?

Answer: We thank the Reviewer for raising this point. As suggested, we silenced STING in ATG5 KO HUVECs. The knockdown caused a reduction of approximately 40% of the STING level, which was better tolerated by the autophagy-deficient endothelial cells compared to the full DKO of Atg5 and STING obtained by CrisprCas9-based KO. As shown in the **RL Fig. 2** reported below, in line with the data shown in Fig4L using the acute pharmacological inhibition of ULK1/2, the partial reduction of STING in ATG5 KO cells did not alter the upregulation of NF-KB proteins Relb, P52, P100. This further supports our observation that STING is dispensable for NF-kB activation upon ATG5KO, as also observed in the in vivo settings. Because this result just confirms the data shown in the main Figure 4L, we believe that its inclusion in the already dense Fig EV4 is not required. However, we will be happy to add this additional evidence in the Expanded View, if the Reviewer finds it more appropriate.
Regarding the representation of the variability, in the **RL Fig. 3** we provide the Reviewer a few representative WBs indicating that specifically in the DKO conditions VCAM1 upregulation, as a readout of the effects of concomitant autophagy and cGAS or STING ablation, are very inconsistent and vary from donor to donor. This is likely due to the compromised status of the DKO HUVECs, and the reason why we decided to use ULK1/2 inhibition in STING KO conditions.

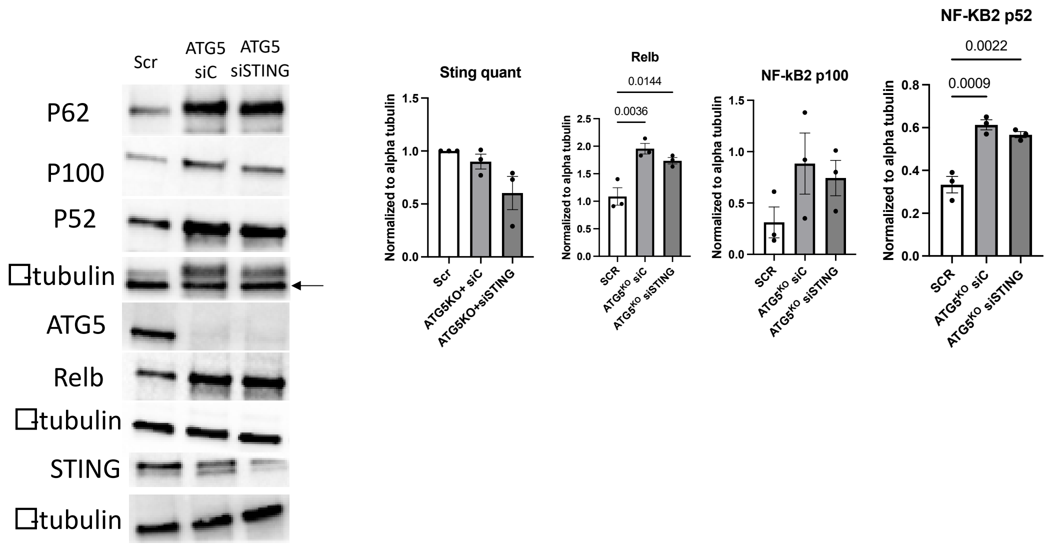

RL Figure 2: (Left) Representative western blot of Atg5, NF-κB2(p100 and p52), p62 and RelB in Scr, Atg5KOsiC, and ATG5KOsiSTING HUVECs. **(Right)**, relative quantification of NF-κB2(p100), STING , RelB, NF-κB2(p52) in Scr, Atg5KOsiC, and ATG5KOsi STING HUVECs

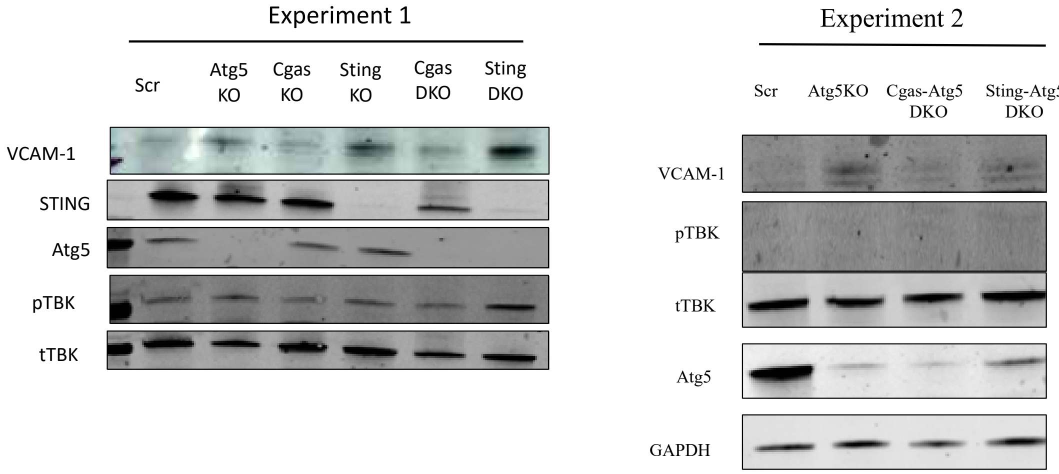

RL Figure 3: (left) representative western blot of VCAM1, STING ATG5 and phospho-TBK1. Total TBK1 serves as loading control. **(Right)** representative western blot of VCAM1, phosphor-TBK1 and ATG5. Total TBK1 and GAPDH serve as loading controls.

6. In Fig.6f and Extended data Fig.6d, the muTEC-DE signature is tested in the different types of TECs. In the discussion (lines 466-468), Authors write: "the enrichment of the muTEC-DE was associated with the lowest expression levels of autophagy genes, particularly in the Interferon EC subtype". However, graphs indicating the decrease in the

autophagy signature corresponding to the EC subtypes are missing, therefore this association are not shown, while it should be.

Answer: We thank the Reviewer for pinpointing this inconsistency. We have now included this association in the FigEV 6D.

Minor issues

Answer: We thank the Reviewer for the careful reading and editing of the Manuscript. Please note that all the minor issues/typo's have been amended according to the Reviewer's suggestions.

1. Line 58, what TME stands for should be mentioned.
2. Lines 105 and 108, "Recently, in a model of acute... in a model of acute physiological inflammation". "Acute physiological inflammation" is duplicated.
3. Lines 144 to 147, "We observed similar phenotypic differences in tumor burden when comparing WT and Atg12ECKO or Atg9ECKO mice inoculated with the immunogenic YUMMER 1.7 melanoma cells, carrying the BrafV600E/Pten−/−/Cdkn2a−/− mutations, or B16-F10, respectively (Fig. 1e-h)". For a better understanding of the text and the figures, Authors should rephrase. For example, they could write "We observed similar phenotypic differences in tumor burden when comparing WT and Atg9ECKO or Atg12ECKO mice inoculated with B16-F10 or with the immunogenic YUMMER 1.7 melanoma cells, carrying the BrafV600E/Pten−/−/Cdkn2a−/− mutations, respectively".
4. Line 146, "BrafV600E/Pten−/−/Cdkn2a−/−" should be written instead of "BrafV600E,Pten−/−/Cdkn2a−/−"
5. Line 156, Authors write "an indicator of a diminished immunosuppressive status of the TME". They should provide a reference supporting this sentence.
6. Line 165, word "mice" is duplicated.
7. Line 186, it should be Fig.3c instead of Fig.3b.
8. Line 202, Authors write "the levels of VCAM1, ICAM1 and of STING". The preposition "of" before "STING" should be removed.
9. Line 202, the verb "is" is duplicated.
10. Line 263, The Extended data Fig.4f has a FACS gating and a graph that does not correspond to each other. Thus, the Extended data Fig.4f in line 263 should be extended data Fig.4g and all the label of all the following Extended data Fig.4 should be changed:
a. Line 279, should be Extended data Fig.4h
b. Lines 282 and 284, should be Extended data Fig.4i
c. Line 297, Extended data Fig.4j instead of Extended data Fig.4i
11. Line 272 and 273, similarly to what mentioned in point 1, what "ER" and "ERGIC" stand for should be mentioned.
12. Line 287, there is a missing bracket at the end of the sentence.
13. Line 294, Fig.4k should be Fig.4i instead.
14. Line 310, there is a typo on the word "both".
15. Lines 334-335, Authors write "monitored tumor growth and inspected the TEC phenotype by IHC for the presence of proinflammatory/immunomodulatory factors". However, the images shown correspond to immunofluorescence and not immunohistochemistry.

16. Lines 368-370, Authors write "Our previous analysis indicated that particularly, the huTEC interferon subtype, of treatment-naïve cancer patients, exhibited a divergent association between the expression of muTEC-DEHigh and autophagyLow gene signatures". This statement is not clear and Authors should rephrase it. For example, they could write "Our previous analysis indicated that particularly, the huTEC interferon subtype of treatment-naïve cancer patients, exhibited a divergent association between the expression of muTEC-DEHigh and autophagyLow gene signatures".
17. Lines 375 and 394, the abbreviation "ON" (for ON treatment) does not correspond with the abbreviation showed in Fig.6d-f and Extended data Fig.6d.
18. Lines 384 and 387, should refer to Fig.6e and not to Fig.4e.
19. Line 392, should refer to Extended data Fig.6b,c and not to Extended data Fig.6a,b.
20. Line 392, Authors write "Compared to non-responders (NR), melanoma patients, responding to". This statement is not clear, and Authors should rephrase it. I would write "Compared to non-responders (NR), melanoma patients responding to".
21. Line 395, the figures mentioned are (Fig. 6h; Extended data Fig. 6c), when should be Fig.6f and Extended data Fig.6d.
22. Lines 452-454, in Line 453 there is a clear anacoluthon. I would suggest to rather remove line 453 or connect it by a comma with the line 452.
23. Line 536, Authors write "cell expression, of STING.". The comma should be removed and Authors should write "cell expression of STING."
24. Lines 616-617, Authors should remark that the double stimulation mentioned in these lines is for the co-culture with Jurkat cells.
25. Line 617, it is written that the incubation is for 12h. However, in line 1232 is written 24h incubation. What is the correct timing?

Answer: This was indeed a typo. We have amended the text to say the correct incubation time of 12 hrs.

26. Line 724 has a typo. Adhering to the USA English style, the word "analysed" should be "analyzed".
27. Line 764, replace the comma at the end of the sentence by a full stop.
28. Line 807, it is written "frenquenT-cell" instead of "frequent".
29. Lines 1087-1088, should be Kaplan-Meier instead of "Kapelin-meyer".
30. Line 1101 has a typo. Adhering to the USA English style, the word "tumours" should be "tumors".
31. Line 1111, in the sentence "in and HUVECs", remove "and".
32. Line 1123, missing capital letters in HUVECs.
33. Line 1125, should include the reference to the Fig.4l, corresponding to ULK1/2 inhibition.
34. Line 1126, the end of the sentence should be a full stop and not a bracket.
35. Lines 1128-1129, Fig.4m should be Fig.4l.
36. Lines 1160 and 1172, the "track changes" marks of Word should be removed.
37. Line 1184, duplication of "subcutaneous B16-F10 tumors".
38. Lines 1198 and 1206, "mice" instead of "Mice".
39. Line 1209, "10µm VCAM1" should be "10µm for VCAM1".
40. Line 1219, "In" should be "in".

41. Line 1229, typo in "cytometric".
42. Lines 1229 and 1235, "Ctr" is highlighted in grey.
43. Lines 1258-1259, "UMAP of ECs color coded for the indicated cell types" sentence is duplicated.
44. Extended data Fig.3, as in the other figure legends, also write the scale bar µm value.
45. Fig.4i, the title of the top of the graph can be confusing with the Y axes title. I would suggest using the graphs title as Y axes title and remove the title of the top of the graph.
46. Fig.5c, Authors should put tumor volume (mm3) as title of Y axes, as in the other figures representing the same endpoint.
47. Extended data Fig.3b, similarly to what mentioned in points 1 and 11, what "FOV" stands for should be mentioned.
48. Extended data Fig.4e, the ULK1/2 should indicate that Y axes is the fold change, as in the Bafilomycin A graphs
49. Fig.4l, to keep the same nomenclature as in the Western Blot, Authors should write "Ctr" instead of "Scr".

Referee #2 (Remarks for Author):

In this study, Verhoeven et al made a great deal of efforts in addressing a key question whether autophagy in tumor endothelial cells (TECs) plays an important role in modulating immune surveillance and tumor growth, mainly by using melanoma-bearing mouse models with defective autophagy, including conditional deletion of Atg5, Atg12 and Atig9 in ECs. The main conclusion of this study is that autophagy in TEC is capable of suppressing immune surveillance of the host against tumor cells and thus promoting tumor growth. There are several important issues to be addressed to improve the study.

1) The concept of tumor endothelial cells (TECs) is not well described.

Answer: We thank the Reviewer for the appraisal of our study. In accordance with the reviewer's suggestion, we have better defined 'tumor endothelial cells' or TECs in the introduction. "*Compared with the mostly quiescent endothelial cells of healthy tissues, tumor endothelial cells (TECs) are constantly exposed to angiogenic cues driving functional and structural abnormalities, including loose intercellular junctions, reduced pericyte coverage, and vascular leakiness"* (Jain, 2014; García-Caballero *et al*, 2022; Schaaf *et al*, 2018).

More importantly, it is not clear how the authors differentiate the TECs from the no tumor endothelial cells in the animal models in vivo, based on the fact that the conditional deletion of Atgs was in all ECs.

Answer: We thank the Reviewer for highlighting this point, indeed conditional deletion of the different Atgs used in our study, will affect not only TECs but also ECs in other compartments/organs. However, in terms of distinguishing TECs from other ECs all the analysis performed in our study, all FACs, sorting, and RNA profiling were done on ECs isolated from the tumor itself, which identify them as TECs. For example, for the Nanostring analysis, mice were sacrificed, and tumors were excised, tissue was digested to extract TECs as specified in the methods. Only TECs were sorted and subjected to RNA

profiling (see new Fig EV3A for gating strategy). For immunostainings, only tumor tissue was taken. Thus, while we can be confident that the structural and functional data presented in our study represent the TEC status of tumor-bearing WT and ATG5/12/9^{ECKO} mice, we cannot rule out that deletion of autophagy in non-tumoral blood vessels may contribute to the observed phenotype.

In a preliminary assay, isolation of lung ECs from B16-F10 bearing mice did not reveal a significant upregulation in the expression of VCAM1 (**RL Figure 4**), suggesting that EC-autophagy within the hostile TME is particularly involved in subverting the TEC inflammatory phenotype. However, further studies, which go beyond the scope of the current manuscript, are required to properly confirm this hypothesis.

Figure for reviewers removed

2) In this study, the authors did not investigate the effect of autophagy on ECs including TECs, such as the viability and proliferation of TECs. In other words, it is not clear how deletion of Atgs in ECs would affect angiogenesis in the xenografted melanoma. I believe that this is a critical point overlooked by the authors in this study.

Answer: We appreciate the Reviewer's comment. To further characterize the TEC phenotypes, we performed stainings for pH3 and cleaved caspase 3 in B16-F10 tumors from WT and ATG5^{BECKO}. We could not detect significant pH3 positive ECs in tumor vessels of our sections. Please note that as a comparison between conditions, all stainings were performed in tissues from end-point tumors, therefore we cannot discount that autophagy may play a role in tumor vessel proliferation at different stages of tumor growth. Double staining for CD31/cleaved caspase 3 in tissues from tumor-bearing WT or ATG5^{BECKO} mice, to assess the effects of autophagy blockade on apoptotic cell death in tumoral EC, did not reveal significant differences either, **Figure RL5**.

In line with this, we also measured tumor vessel density (as a parameter of vessel area/tumor section) and found no difference between tumor-bearing WT and ATG5^{BECKO} mice (**Figure RL5**), suggesting that upon loss of autophagy the overall fraction of tumor vessels is not significantly affected in these conditions.

Figure for reviewers removed

In answer to the Reviewer's comment, to further elucidate the role of autophagy in vessel function, we injected tumor-bearing ATG12^{ECKO} mice with Dextran through the tail vein and allowed it to circulate for 2 hr prior to sacrifice. Mice were perfused with PFA and PBS and tumors (about 300 mm^3) were excised for analysis. Dextran staining, now reported in Fig EV1G, shows that compared with WT mice vessel leakage in tumor-bearing ATG12^{ECKO} mice is partially, but significantly, reduced suggesting improved vessel functionality. This, together with the effects of loss of autophagy in fostering the inflammatory status of TEC, is consistent with the increased immunosurveillance observed in these mice.
We have now placed these new dextran stainings in Fig EV1G along with a comment on the findings in the text Lines 149-155.
We would not integrate the Cleaved caspase 3 and vessel density data in the Extended data of Fig. 1, not to overload this already dense supplementary figure.

3) Figure 1: The authors should present evidence for the defective autophagy in ECs with deletion of Atgs (Atg5, Atg9 and Atg12).

Answer. We thank the Reviewer, this is indeed an important point, which we had not fully addressed. We have now performed additional stainings using p62 accumulation in CD31 positive vessels, as a marker of defective autophagic flux in TECs. Imaging of double CD31+/p62+ vessels in B16-F10-bearing WT, ATG5^{ECKO}, ATG9^{ECKO} and ATG12^{ECKO} mice indicated a significant increase in p62 granularity (MFI) (thus decreased autophagic flux) upon deletion of these autophagy genes in TECs. We have added imaging and quantification of p62 from all transgenic mice models used in this study to Fig EV1D, E.

4) Figure 2: The panel g and h appear to be redundant data, both showing the tumor volume changes over time among different groups.

Answer. As requested by the reviewer, we have removed the previous panel g in Figure 2 to reduce redundancy.

5) Figure 3f - 3h: Data from these 3 panels were from bioinformatics analysis from primary human cancers: Better to move to suppl figures.

Answer. We respectfully disagree with the Reviewer, the data from different primary human cancers shown Figure 3F – 3H demonstrates that our signature does not only apply to melanoma but has pan-cancer applications. Because of the relevance of the human data, and the species (mouse and human) independent validation of the autophagy signature we wish to keep this data in the main Figure. We hope that the Reviewer will acknowledge this point.

6) Figure 4k: The authors should present evidence demonstrating the impaired autophagic flux in ATG5 KO HUVECs.

Answer. We thank the Reviewer for pointing out this omission. As for the in vivo settings, we have now added p62 in the Western Blot of Figure 4k, indicating its accumulation in autophagy-deficient HUVECs as a readout of impaired autophagic flux.

7) The authors should present a summary illustration to highlight the main findings of this study, either as a separate figure or as the last panel in Figure 7.

Answer. As per the Reviewer's request, we have now added a graphical abstract illustrating the main observations and take-home message of the study.

Referee #3 (Remarks for Author):

The major issue of this study is whether the inflammatory EC is required for immune or therapeutic response against tumor, so the autophagy in EC is a genuine immune checkpoint, or it is the secondary event in the "hot" tumor microenvironment. The data (e.g. Fig. 1i and 1j, 2c and 2e) suggested that inflammatory EC enhanced, but is not required for, the recruitment of activated T cells. In that case, for example, tumor may use dissipating factors (e.g. hypoxic or lactate) to suppress T cell activities and induce autophagy in EC simultaneously. This also explained the marginal effect of Atg5b KO on response of melanoma to anti-PD-1 in the mouse studies (Figure 6a-c). These should be discussed in the text.

The Reviewer raises an interesting point. Indeed, several tumor microenvironmental factors, including hypoxia and acidosis, are known stimulants of autophagy in cancer cells and stromal cells. However, how cell-autonomous autophagy is harnessed to support tumor growth, while avoiding anti-tumor immunity, largely depends on the type of cancerous/non-cancerous cells and the pathways that autophagy contextually controls in these cells. Vascular remodeling and immunosuppression are intimately intertwined. We believe that our study provides evidence that a dominant anti-inflammatory function of autophagy in tumor vessels contributes to dampening T-cell recruitment and activation. Autophagy repression of TEC inflammation is exerted by simultaneously hampering STING and NF-kB pathways, immunomodulatory signals that in human melanoma samples correlate with CD8 T cells vicinity to the tumor vessels.

The combined control of both (NF-kB and STING) immunomodulatory signals by TEC-associated autophagy portrays the relevance of this pathway which is *per se* able to reprogram -albeit not completely- as the Reviewer correctly points out, the immunosuppressive microenvironment of the 'cold' B16-F10 melanoma.

In support of the direct role of autophagy in suppressing inflammatory signals and maintaining the anergic status of the tumor vasculature, we provide to the Reviewer in **RL Fig. 6** the data of a preliminary experiment where we used antibody-based neutralization of IFNγ in B16-F10 bearing WT and ATG5BECKO mice. In this experiment, we tested whether the effects of reduced tumor burden observed upon TEC-autophagy depletion were secondary to cytotoxic T cell-derived IFNγ. While neutralizing IFNγ accelerates tumor growth to some extent in both the WT and $ATG5^{BECKO}$ mice, the antitumor effects of $ATG5^{BECKO}$ were not reversed. Although quite preliminary, these data suggest that the effects of autophagy-deletion in TECs are not secondary to the improved antitumor immunity, and further highlight the immunomodulatory role of TEC-autophagy in antitumor immunity. The effect of the genetic deletion of TEC autophagy, seems thus different from the role in antitumor immunity induced by loss of host or liver autophagy reported in previous studies, showing that tumor burden control in autophagy-deficient mice was secondary to cytotoxic T-cell-mediated IFNγ and abolished by its neutralization (Poillet-Perez *et al*, 2020).

Figure for reviewers removed

Together with the human melanoma data presented in this study, unraveling the inverse correlation between, TEC-autophagy status and patient responses to anti-PD1, we believe that TEC-autophagy functions as a bona fide immune-evasion factor. This is in line with the emerging role of tumor endothelial cells in immunomodulation (Nagl *et al*, 2020). The observation that blockade of autophagy in TECs does not significantly enhance anti-PD1 monotherapy, in our opinion, is a reflection of the already favorable environment generated by the genetic loss of TEC-autophagy, causing a reprogramming of the local immunosuppressive B16-F10 TME. Although we did not address in detail the effects of anti-PD1 on the tumor vasculature, our data suggest that loss of autophagy in tumor vessels induces a trend toward better pericyte coverage and reduced vascular leakage (additional Dextran experiment in Fig EV1G), which has been shown to favor immunosurveillance and T cell-mediated responses either alone or in combination with immunotherapy reviewed in

(Khan & Kerbel, 2018). However, it is clear that this reprogramming per se is not able to overcome the immunosuppressive TME generated by a cold tumor. We briefly discuss these points as suggested by the Reviewer in page 16 (555-561) by adding " Our data underscore that autophagy is a persuasive TEC-intrinsic immune-evasion factor, which could be fostered in the tumor by the metabolically stressed TME to enforce immunosuppression(Nagl *et al*, 2020; Fischer *et al*, 2018). This is likely the reason why the sole blockade of TEC-autophagy reduces but does not prevent melanoma progression. Furthermore, additional experiments including antibody-based IFNγ neutralization, should rule out the contribution of cytotoxic T cell-derived IFNγ to the antitumor effects observed in $Atg5^{BECKO}$ bearing mice".

Technical question: are there any other cell types targeted by Pdgfb-CreERT2 or Ve-cadh-CreERT2 in tumor microenvironment?

Answer. Endothelial cells (both blood and lymphatic) are the major cells targeted by Vecadherin-CreERT2 in the tumor microenvironment. Blood endothelial cells, pericytes, smooth muscle and megakaryocytes all express PDGFb.
Both Pdgfb-CreERT2 and Ve-cadh-CreERT2A are amongst the most widely used EC-targeted Cre models (see (Payne *et al*, 2018)).

References

Fischer GM, Vashisht Gopal YN, McQuade JL, Peng W, DeBerardinis RJ & Davies MA (2018) Metabolic strategies of melanoma cells: Mechanisms, interactions with the tumor microenvironment, and therapeutic implications. *Pigment Cell Melanoma Res* 31: 11–30

García-Caballero M, Sokol L, Cuypers A & Carmeliet P (2022) Metabolic Reprogramming in Tumor Endothelial Cells. *Int J Mol Sci* 23: 11052

Jain RK (2014) Antiangiogenesis strategies revisited: from starving tumors to alleviating hypoxia. *Cancer Cell* 26: 605–622

Khan KA & Kerbel RS (2018) Improving immunotherapy outcomes with anti-angiogenic treatments and vice versa. *Nat Rev Clin Oncol* 15: 310–324

Nagl L, Horvath L, Pircher A & Wolf D (2020) Tumor Endothelial Cells (TECs) as Potential Immune Directors of the Tumor Microenvironment – New Findings and Future Perspectives. *Front Cell Dev Biol* 8

Payne S, De Val S & Neal A (2018) Endothelial-Specific Cre Mouse Models: Is Your Cre CREdibile? *Arterioscler Thromb Vasc Biol* 38: 2550–2561

Poillet-Perez L, Sharp DW, Yang Y, Laddha SV, Ibrahim M, Bommareddy PK, Sherrie Hu Z, Vieth J, Haas M, Bosenberg MW, *et al* (2020) Autophagy promotes growth of tumors with high mutational burden by inhibiting a T-cell immune response. *Nat Cancer* 1: 923–934

Schaaf MB, Garg AD & Agostinis P (2018) Defining the role of the tumor vasculature in antitumor immunity and immunotherapy. *Cell Death Dis* 9: 115

Wang J, Perry CJ, Meeth K, Thakral D, Damsky W, Micevic G, Kaech S, Blenman K & Bosenberg M (2017) UV-induced somatic mutations elicit a functional T cell response in the YUMMER1.7 Mouse Melanoma Model. *Pigment Cell Melanoma Res* 30: 428–435

Xu Q, Zhang Z, Chen Z, Zhang B, Zhao C, Zhang Y, Zhao C, Deng X, Zhou Y, Wu Y, *et al* (2019) Nonspecific immunoglobulin G is effective in preventing and treating cancer in mice. *Cancer Manag Res* 11: 2073–2085

Zhang T, Yin C, Fedorov A, Qiao L, Bao H, Beknazarov N, Wang S, Gautam A, Williams RM, Crawford JC, *et al* (2022) ADAR1 masks the cancer immunotherapeutic promise of ZBP1-driven necroptosis. *Nature* 606: 594–602

18th Oct 2023

Dear Patrizia,

Thank you for submitting your revised manuscript. We have now received the reports from the referees, and as you will see below, they are supportive of publication pending minor revisions. I will therefore be able to accept your manuscript once the following points will be addressed:

1/ Referees' comments:
- Referee #2: please discuss the potential role of non-tumor EC in your manuscript. Please note that we DO NOT ask you to modify the title or abstract.
- Referee #3: we'd like you to discuss the referee's point (additive vs. synergistic effect) and if you do have data at hand, we'd be happy for you to include it, however we will NOT ask you to provide any additional experiments at this stage.

2/ Manuscript text:
- Please remove the yellow highlights and only keep in track changes mode any new modification.
- Please rename "Methods" to "Materials and methods"
o Cell culture: please indicate the origin of the cancer cells, and whether they were authenticated.
o Mice: please provide the housing and husbandry conditions.
o Antibodies: please make sure to indicate the used dilutions/concentrations for all antibodies (i.e. including IF experiments).
o Patients' samples: please include the full sentence that the experiments conformed to the principles set out in the WMA Declaration of Helsinki and the Department of Health and Human Services Belmont Report.
- Data Availability section: This section should directly follow the Materials and Methods and include URL links to the deposited datasets.
Please note that the Data Availability Section is restricted to new primary data that are part of this study. To reference previously published datasets, our journal supports *formal data citations in the Reference list*. In addition to the citation to the original research papers that reported the data, we encourage you to also cite the relevant datasets directly in the Reference list, to provide credit to the authors and to help readers accessing the datasets. In the text of the article, references to datasets are simply included as "Data ref: Smith et al, 2001" or "Data ref: NCBI Sequence Read Archive PRJNA342805, 2017". In the Reference list, data citations are very similar to normal literature reference and must be labeled with "[DATASET]" at the end of the reference.
- Acknowledgements: Please make sure that the information provided in the manuscript matches the information provided in the submission system (ATLANTIS consortium and grant number 11L7622N are currently missing from the submission system).
- Please rename "Disclosure of competing interests" to "Disclosure Statement and Competing Interests".

3/ Figures and Appendix:
- Figure 7E-H: please mention in the figure legend any tissue re-use.
- As we usually accommodate a maximum of 5 EV figures, we would encourage you to either merge Figure EV6 and EV7 to the other EV figures, or to provide them in an Appendix pdf file, with page numbers and table of content (the nomenclature should be Appendix Figure S1, etc).
- The table EV legends need to be provided in the table files; the correct nomenclature and callouts are Table EV1 and Table EV2.
- Please rename "Extended data Fig 1", etc. to "Expanded View Figure 1" etc.
- Figure legends:
1. Please note that the figure legend style does not comply with the journal guidelines i.e. all the figure legends are in a run-on style.
2. Please note that a separate 'Data Information' section is required in the legends of all figures.
3. Please note that information related to n is missing in the legends of figures 1b, d, f, h; 3e-h; 4a-h, l; 5a, c; 6e-g; 7a-d; extended data figures 2b; 3b, g; 4c, e; 5a-c; 6a, d
4. Please note that the box plots need to be defined in terms of minima, maxima, bounds of box and whiskers, and percentile in the legends of figures 3f; 6e-f; extended data figures 3g; 6d
5. Please note that the error bars are not defined in the legend of extended data figure 6a.

4/ Thank you for providing the Source Data. Please make sure they are provided for all requested items (for instance files 5d, 5g are empty).

5/ Checklist: please make sure that both left and right columns are filled when applicable (i.e. statistical tests).

6/ Synopsis:
- Please resize the synopsis image to 550 pixels wide x 200-400 pixels high and make sure the text remains legible.
- I slightly edited your text to match our style and format, in particular the first paragraph must be 300 characters maximum.

Please let me know if you agree with the following or amend as you see fit:

Tumor endothelial cell (EC)-autophagy was identified as a major barrier to anti-tumor immune responses against melanoma.
- Endothelial cell-specific knockout of autophagy promoted the concomitant activation of the STING-Type I Interferon and NF- B signaling.
- An "inflamed TEC" signature, identified in EC-specific autophagy-deficient mice, correlated with low vascular autophagy levels across several human cancer types.
- An InflamedHigh/AutophagyLow status in TECs of melanoma patients was associated with better response to anti-PD1 therapy.

7/ As part of the EMBO Publications transparent editorial process initiative (see our Editorial at http://embomolmed.embopress.org/content/2/9/329), EMBO Molecular Medicine will publish online a Review Process File (RPF) to accompany accepted manuscripts.
This file will be published in conjunction with your paper and will include the anonymous referee reports, your point-by-point response and all pertinent correspondence relating to the manuscript. Let us know whether you agree with the publication of the RPF and as here, if you want to remove or not any figures from it prior to publication.
Please note that the Authors checklist will be published at the end of the RPF.

I look forward to receiving your revised manuscript.

With kind regards,

Lise

Lise Roth, PhD
Senior Editor
EMBO Molecular Medicine

***** Reviewer's comments *****

Referee #1 (Remarks for Author):

Suitable for publication

Referee #2 (Comments on Novelty/Model System for Author):

The model system used in this study is appropriate.

Referee #2 (Remarks for Author):

In this revised MS, the authors have made a great deal of efforts in addressing the comments from the reviewers, including those from mine, with addition of new data and revision of the text.

Here I still have one point that remains to be further addressed: since the conditional deletion of Atgs was in all ECs, and the authors did not provide any direct evidence to exclude the potential role and contribution from no tumor endothelial cells in the animal models in vivo (despite the fact that they did many experiments using ECs isolated from the tumor), the authors should tone down their conclusions on TECs and revise the title, abstract and the text (esp the discussion and conclusion) accordingly.

Referee #3 (Comments on Novelty/Model System for Author):

The "M1" and "M2" represent ICB-resistant melanoma models that exhibit T cell exhaustion and exclusion phenotypes (https://www.nature.com/articles/s41591-020-0818-3). They should be better models to test the author's hypothesis. However, considering the logistics of mouse studies, that using multiple conventional models to represent non-immunogenic and immunogenic tumors should be able to address most of the major questions. Using the better immunologically characterized M1

and M2 models can improve their following up studies in the future.

Referee #3 (Remarks for Author):

The authors assumed that IFNG is the major effector of T cells. They blocked IFNG and found the KO of an autophagy gene still delayed tumor growth. Therefore, they concluded that EC-mediated immunosuppressive effect is not a secondary event following T cell response.
The preliminary results could be better interpreted as that KO of an autophagy gene in EC additively enhanced T cell-mediated anti-tumor effect.
Importantly, to distinguish if this is an additive or synergistic effect, the T cells from WT and Atg5/Atg9-KO mice (tumor-free) should be analyzed if the KO itself could enhance overall T cell activity.
Additional comments:
1. Each figure needs to be marked with figure number. The figure legends need to be self-sufficient to explain the content, including (1) fully spelling the terminology instead of using only acronym; (2) explaining the procedure instead of referring to the text.
2. The tumor growth kinetics in the WT and Atg5-KO mice in Fig. 1A and Fig. 2G are not consistent.

Point-by point answer

Referee #2 (Comments on Novelty/Model System for Author):

The model system used in this study is appropriate.

Referee #2 (Remarks for Author):

In this revised MS, the authors have made a great deal of efforts in addressing the comments from the reviewers, including those from mine, with addition of new data and revision of the text.
Here I still have one point that remains to be further addressed: since the conditional deletion of Atgs was in all ECs, and the authors did not provide any direct evidence to exclude the potential role and contribution from no tumor endothelial cells in the animal models in vivo (despite the fact that they did many experiments using ECs isolated from the tumor), the authors should tone down their conclusions on TECs and revise the title, abstract and the text (esp the discussion and conclusion) accordingly.

Answer. We thank the Reviewer for the appraisal of our work. We agree with the Reviewer that, based on our model of conditional Atg5 KO in all ECs we cannot exclude that beyond the major effects of TEC-autophagy deletion reported in our study, additional effects caused by the loss of autophagy in non-tumor ECs contribute to support immunosurveillance.
As per the suggestions of the Senior Editor, we haven't revised the title and abstract but elaborated further on the potential role of non-TEC-autophagy in the discussion section, page 15, by citing also additional studies in liver sinusoidal ECs.
"*Consistent with a potentially different outcome of autophagy deficiency in specific EC subsets, genetic loss of Atg5 in liver sinusoidal ECs stimulated VCAM1 expression and features of endothelial-to-mesenchymal transition, which accelerated liver inflammation and fibrosis, only when exposed to TNF* (Hammoutene et al, 2020)*. While we cannot rule out that genetic loss of autophagy in non-tumor ECs contributed to the tumor burden control we observed in melanoma-bearing mice, it is possible that the presence of melanoma-derived proinflammatory cytokines in the TME may further accentuate the effects of autophagy deficiency in TECs, as compared with non-cancerous ECs of other vascular beds. A hypothesis that needs to be fully addressed in further studies*".

Referee #3 (Comments on Novelty/Model System for Author):

The "M1" and "M2" represent ICB-resistant melanoma models that exhibit T cell exhaustion and exclusion phenotypes (https://www.nature.com/articles/s41591-020-0818-3). They should be better models to test the author's hypothesis. However, considering the logistics of mouse studies, that using multiple conventional models to represent non-immunogenic and immunogenic tumors should be able to address most of the major questions. Using the better immunologically characterized M1 and M2 models can improve their following up studies in the future.

Answer. We concur with the suggestions of the Reviewer using additional ICB-resistant melanoma models in future studies.

Referee #3 (Remarks for Author):

The authors assumed that IFNG is the major effector of T cells. They blocked IFNG and found the KO of an autophagy gene still delayed tumor growth. Therefore, they concluded that EC-mediated immunosuppressive effect is not a secondary event following T cell response.
The preliminary results could be better interpreted as that KO of an autophagy gene in EC additively enhanced T cell-mediated anti-tumor effect.
Importantly, to distinguish if this is an additive or synergistic effect, the T cells from WT and Atg5/Atg9-KO mice (tumor-free) should be analyzed if the KO itself could enhance overall T cell activity.

Answer. We agree that our preliminary results using IFNg depletion are not sufficient to draw a conclusive statement on the complex interface between tumor endothelial cells and anti-tumor immunity, for which further studies are required.
We have discussed the potential role of TEC-autophagy in T cell mediated effects further on page 17, by highlighting the possibility that loss of TEC-autophagy may elicit additive rather than synergistic effects on T cells functions, partially because loss of autophagy does not fully evoke vessel normalization, which has been reported in previous studies to synergize with anti-PD1 therapy.
"*Our data underscore that autophagy is a persuasive TEC-intrinsic immune-evasion factor, which could be fostered in the tumor by the metabolically stressed TME to enforce immunosuppression (Debnath et al, 2023). This is likely the reason why the sole blockade of TEC-autophagy reduces but does not prevent melanoma progression. Although a partial reversal of tumor vessel abnormalities was observed in our EC autophagy-deficient melanoma-bearing mice, this is possibly insufficient to induce bona fide vessel normalization which has been shown to synergize with immunotherapy regimens (Dong et al, 2023; Tian et al, 2017). Loss of autophagy in ECs may thus potentiate T cell function resulting in an additive -rather than synergistic- anti-tumor effect. Additional analysis of the effects of anti-PD1 on tumor vessel normalization in wild-type and EC autophagy-deficient mice should help clarify this point. Furthermore, experiments including e.g. antibody-based IFNγ neutralization could be also performed to assess the (secondary) contribution of cytotoxic T cell-derived IFNγ to the anti-tumor effects observed in Atg5^{BECKO} bearing mice (Zheng et al, 2018)*".

We hope that the Reviewer is satisfied with the additional explanation we have provided.

Additional comments:
1. Each figure needs to be marked with figure number. The figure legends need to be self-sufficient to explain the content, including (1) fully spelling the terminology instead of using only acronym; (2) explaining the procedure instead of referring to the text.

Answer. Figures have been marked and legends have been adapted and extended according to the EMBO MM Journal standard.

2. The tumor growth kinetics in the WT and Atg5-KO mice in Fig. 1A and Fig. 2G are not consistent.

Answer. We respectfully disagree with the Reviewer's comment. In Fig. 2G tumor growth was extended up to 20 days, considering the 15 day's time points the tumor growth kinetics are rather consistent between independent experiments.

30th Oct 2023

Dear Patrizia,

Thank you for bearing with the last editorial concerns and sending the revised files. Please note that we have added a table of content to your Appendix file.

I am pleased to inform you that your manuscript is accepted for publication and is now being sent to our publisher to be included in the next available issue of EMBO Molecular Medicine!

Please read below for additional IMPORTANT information regarding your article, its publication and the production process.

Congratulations on your interesting work!

With kind regards,

Lise

Lise Roth, Ph.D
Senior Editor
EMBO Molecular Medicine

Follow us on Twitter @EmboMolMed
Sign up for eTOCs at embopress.org/alertsfeeds

*** *** *** IMPORTANT INFORMATION *** *** ***

SPEED OF PUBLICATION
The journal aims for rapid publication of papers, using using the advance online publication "Early View" to expedite the process: A properly copy-edited and formatted version will be published as "Early View" after the proofs have been corrected. Please help the Editors and publisher avoid delays by providing e-mail address(es), telephone and fax numbers at which author(s) can be contacted.

Should you be planning a Press Release on your article, please get in contact with embomolmed@wiley.com as early as possible, in order to coordinate publication and release dates.

LICENSE AND PAYMENT:

All articles published in EMBO Molecular Medicine are fully open access: immediately and freely available to read, download and share.

EMBO Molecular Medicine charges an article processing charge (APC) to cover the publication costs. You, as the corresponding author for this manuscript, should have already received a quote with the article processing fee separately. Please let us know in case this quote has not been received.

Once your article is at Wiley for editorial production you will receive an email from Wiley's Author Services system, which will ask you to log in and will present you with the publication license form for completion. Within the same system the publication fee can be paid by credit card, an invoice, pro forma invoice or purchase order can be requested.

Payment of the publication charge and the signed Open Access Agreement form must be received before the article can be published online.

PROOFS

You will receive the proofs by e-mail approximately 2 weeks after all relevant files have been sent o our Production Office. Please return them within 48 hours and if there should be any problems, please contact the production office at embopressproduction@wiley.com.

Please inform us if there is likely to be any difficulty in reaching you at the above address at that time. Failure to meet our deadlines may result in a delay of publication.

All further communications concerning your paper proofs should quote reference number EMM-2023-18028-V3 and be directed to the production office at embopressproduction@wiley.com.

EMBO Press Author Checklist

Patrizia Agostinis
EMBO Molecular Medicine
EMM-2023-18028

USEFUL LINKS FOR COMPLETING THIS FORM
[The EMBO Journal - Author Guidelines]
[EMBO Reports - Author Guidelines]
[Molecular Systems Biology - Author Guidelines]
[EMBO Molecular Medicine - Author Guidelines]

Reporting Checklist for Life Science Articles (updated January
This checklist is adapted from Materials Design Analysis Reporting (MDAR) Checklist for Authors. MDAR establishes a minimum set of requirements in transparent reporting in the life sciences (see Statement of Task: 10.31222/osf.io/9sm4x). Please follow the journal's guidelines in preparing your
Please note that a copy of this checklist will be published alongside your article.

Abridged guidelines for figures
1. Data
The data shown in figures should satisfy the following conditions:

- the data were obtained and processed according to the field's best practice and are presented to reflect the results of the experiments in an accurate and unbiased manner.
- ideally, figure panels should include only measurements that are directly comparable to each other and obtained with the same assay.
- plots include clearly labeled error bars for independent experiments and sample sizes. Unless justified, error bars should not be shown for technical
- if n<5, the individual data points from each experiment should be plotted. Any statistical test employed should be justified.
- Source Data should be included to report the data underlying figures according to the guidelines set out in the authorship guidelines on Data

2. Captions
Each figure caption should contain the following information, for each panel where they are relevant:

- a specification of the experimental system investigated (eg cell line, species name).
- the assay(s) and method(s) used to carry out the reported observations and measurements.
- an explicit mention of the biological and chemical entity(ies) that are being measured.
- an explicit mention of the biological and chemical entity(ies) that are altered/varied/perturbed in a controlled manner.
- the exact sample size (n) for each experimental group/condition, given as a number, not a range;
- a description of the sample collection allowing the reader to understand whether the samples represent technical or biological replicates (including how many animals, litters, cultures, etc.).
- a statement of how many times the experiment shown was independently replicated in the laboratory.
- definitions of statistical methods and measures:
  - common tests, such as t-test (please specify whether paired vs. unpaired), simple χ2 tests, Wilcoxon and Mann-Whitney tests, can be unambiguously identified by name only, but more complex techniques should be described in the methods section;
  - are tests one-sided or two-sided?
  - are there adjustments for multiple comparisons?
  - exact statistical test results, e.g., P values = x but not P values < x;
  - definition of 'center values' as median or average;
  - definition of error bars as s.d. or s.e.m.

Please complete ALL of the questions below.
Select "Not Applicable" only when the requested information is not relevant for your study.

Materials

Newly Created Materials	Information included in the manuscript?	In which section is the information available? (Reagents and Tools Table, Materials and Methods, Figures, Data Availability Section)
New materials and reagents need to be available; do any restrictions apply?	Not Applicable	

Antibodies	Information included in the manuscript?	In which section is the information available? (Reagents and Tools Table, Materials and Methods, Figures, Data Availability Section)
For **antibodies** provide the following information:<br>- Commercial antibodies: RRID (if possible) or supplier name, catalogue number and or/clone number<br>- Non-commercial: RRID or citation	Yes	methods

DNA and RNA sequences	Information included in the manuscript?	In which section is the information available? (Reagents and Tools Table, Materials and Methods, Figures, Data Availability Section)
**Short novel DNA or RNA including primers, probes**: provide the sequences.	Yes	Methods

Cell materials	Information included in the manuscript?	In which section is the information available? (Reagents and Tools Table, Materials and Methods, Figures, Data Availability Section)
**Cell lines:** Provide species information, strain. Provide accession number in repository **OR** supplier name, catalog number, clone number, and/**OR** RRID.	Yes	Methods
**Primary cultures:** Provide species, strain, sex of origin, genetic modification status.	Yes	methods
Report if the cell lines were recently **authenticated** (e.g., by STR profiling) and tested for mycoplasma contamination.	Yes	Methods

Experimental animals	Information included in the manuscript?	In which section is the information available? (Reagents and Tools Table, Materials and Methods, Figures, Data Availability Section)
**Laboratory animals or Model organisms:** Provide species, strain, sex, age, genetic modification status. Provide accession number in repository **OR** supplier name, catalog number, clone number, **OR** RRID.	Yes	Methods
**Animal observed in or captured from the field:** Provide species, sex, and age where possible**.**	Not Applicable	
Please detail **housing and husbandry conditions**.	Yes	methods

Plants and microbes	Information included in the manuscript?	In which section is the information available? (Reagents and Tools Table, Materials and Methods, Figures, Data Availability Section)
**Plants:** provide species and strain, ecotype and cultivar where relevant, unique accession number if available, and source (including location for collected wild specimens).	Not Applicable	
**Microbes:** provide species and strain, unique accession number if available, and source.	Not Applicable	

Human research participants	Information included in the manuscript?	In which section is the information available? (Reagents and Tools Table, Materials and Methods, Figures, Data Availability Section)
If collected and within the bounds of privacy constraints report on age, sex and gender or ethnicity for all study participants.	Not Applicable	

Core facilities	Information included in the manuscript?	In which section is the information available? (Reagents and Tools Table, Materials and Methods, Figures, Data Availability Section)
If your work benefited from core facilities, was their service mentioned in the acknowledgments section?	Yes	acknowledgement

Design

Study protocol	Information included in the manuscript?	In which section is the information available? (Reagents and Tools Table, Materials and Methods, Figures, Data Availability Section)
If study protocol has been **pre-registered, provide DOI in the manuscript**. For clinical trials, provide the trial registration number **OR** cite DOI.	Not Applicable	
Report the **clinical trial registration number** (at ClinicalTrials.gov or equivalent), where applicable.	Not Applicable	

Laboratory protocol	Information included in the manuscript?	In which section is the information available? (Reagents and Tools Table, Materials and Methods, Figures, Data Availability Section)
Provide DOI OR other citation details if **external detailed step-by-step protocols** are available.	Not Applicable	

Experimental study design and statistics	Information included in the manuscript?	In which section is the information available? (Reagents and Tools Table, Materials and Methods, Figures, Data Availability Section)
Include a statement about **sample size** estimate even if no statistical methods were used.	Yes	Methods (statistics)
Were any steps taken to minimize the effects of subjective bias when allocating animals/samples to treatment (e.g. **randomization procedure**)? If yes, have they been described?	Yes	Yes methods statistics
Include a statement about **blinding** even if no blinding was done.	Yes	yes methods (statistics)
Describe **inclusion/exclusion criteria** if samples or animals were excluded from the analysis. Were the criteria pre-established?<br><br>If sample or data points were omitted from analysis, report if this was due to attrition or intentional exclusion and provide justification.	Yes	yes methods statistics
For every figure, are **statistical tests** justified as appropriate? Do the data meet the assumptions of the tests (e.g., normal distribution)? Describe any methods used to assess it. Is there an estimate of variation within each group of data? Is the variance similar between the groups that are being statistically compared?	Yes	Legends, methods, statistics

Sample definition and in-laboratory replication	Information included in the manuscript?	In which section is the information available? (Reagents and Tools Table, Materials and Methods, Figures, Data Availability Section)
In the figure legends: state number of times the experiment was **replicated** in laboratory.	Yes	Legends
In the figure legends: define whether data describe **technical or biological replicates**.	Yes	Legends

Ethics

Ethics	Information included in the manuscript?	In which section is the information available? (Reagents and Tools Table, Materials and Methods, Figures, Data Availability Section)
Studies involving **human participants**: State details of **authority granting ethics approval** (IRB or equivalent committee(s), provide reference number for approval.	Yes	Methods
Studies involving **human participants**: Include a statement confirming that **informed consent** was obtained from all subjects and that the experiments conformed to the principles set out in the WMA Declaration of Helsinki and the Department of Health and Human Services Belmont Report.	Yes	Methods
Studies involving **human participants: For publication of patient photos**, include a statement confirming that consent to publish was obtained.	Not Applicable	
Studies involving experimental **animals**: State details of **authority granting ethics approval** (IRB or equivalent committee(s), provide reference number for approval. Include a statement of compliance with ethical regulations.	Yes	methods
Studies involving **specimen and field samples:** State if relevant **permits** obtained, provide details of authority approving study; if none were required, explain why.	Not Applicable	

Dual Use Research of Concern (DURC)	Information included in the manuscript?	In which section is the information available? (Reagents and Tools Table, Materials and Methods, Figures, Data Availability Section)
Could your study fall under dual use research restrictions? Please check biosecurity documents and list of **select agents and toxins** (CDC): https://www.selectagents.gov/sat/list.htm	Not Applicable	
If you used a select agent, is the security level of the lab appropriate and reported in the manuscript?	Not Applicable	
If a study is subject to dual use research of concern regulations, is the name of the **authority granting approval and reference number** for the regulatory approval provided in the manuscript?	Not Applicable	

Reporting

The MDAR framework recommends adoption of discipline-specific guidelines, established and endorsed through community initiatives. Journals have their own policy about requiring specific guidelines and recommendations to complement MDAR.

Adherence to community standards	Information included in the manuscript?	In which section is the information available? (Reagents and Tools Table, Materials and Methods, Figures, Data Availability Section)
State if relevant guidelines or checklists (e.g., **ICMJE, MIBBI, ARRIVE, PRISMA**) have been followed or provided.	Not Applicable	
For **tumor marker prognostic studies,** we recommend that you follow the **REMARK** reporting guidelines (see link list at top right). See author guidelines, under 'Reporting Guidelines'. Please confirm you have followed these guidelines.	Not Applicable	
For **phase II and III randomized controlled trials**, please refer to the **CONSORT** flow diagram (see link list at top right) and submit the CONSORT checklist (see link list at top right) with your submission. See author guidelines, under 'Reporting Guidelines'. Please confirm you have submitted this list.	Not Applicable	

Data Availability

Data availability	Information included in the manuscript?	In which section is the information available? (Reagents and Tools Table, Materials and Methods, Figures, Data Availability Section)
Have **primary datasets** been deposited according to the journal's guidelines (see 'Data Deposition' section) and the respective accession numbers provided in the Data Availability Section?	Yes	Data availibility
Were **human clinical and genomic datasets** deposited in a public access-controlled repository in accordance to ethical obligations to the patients and to the applicable consent agreement?	Not Applicable	
Are **computational models** that are central and integral to a study available without restrictions in a machine-readable form? Were the relevant accession numbers or links provided?	Yes	materials and methods
If publicly available data were reused, provide the respective **data citations in the reference list**.	Yes	reference list
